# Supplementary material for: DNA Double-Strand Break-Related Competitive Endogenous RNA Network of Noncoding RNA in Bovine Cumulus Cells
Source: Genes (Basel). 2023 Jan 22;14(2):290. doi: 10.3390/genes14020290 (PMC9956238; doi:10.3390/genes14020290)
Supplement: Supplementary file 1 [file genes-14-00290-s001.zip › Table S1.pdf]

Supplementary Table S1.Primers used for qRT-PCR

primers name	sequence(5'-3')
ABHD8-F	TTGTGCCAGTGGAGGAAGAC
ABHD8-R	AGAGCAGGAATTCGTGGAGC
PSPH-F	GTCTCCTGGGAATCCAGCAC
PSPH-R	CTGACACGGCATCCTCAACT
PLK2-F	CTCAGCAACCCAGCAAACAC
PLK2-R	CTTGTTTTCCACTGCGGGTG
ZFP36L1-F	CTCGTGTCTGCCACCATCTT
ZFP36L1-R	CTGAGGAGCTGGTTCTGGTG
SYPL1-F	ACTCCTCTTCTGCGCAGTTC
SYPL1-R	TGAAGTGCTCACCAACCACA
SMC4-F	TCCAGAACGGCCTACAGAGA
SMC4-R	CATCGTGTTCAGTCTGGCCT
GAPDH-F	ACATACTCAGCACCAGCATCAC
GAPDH-R	ATTCTGGCAAAGTGGACATCG
circAC_000159.1:127039209|127042698-F	CATTTGGGTCTCAGGCATCC
circAC_000159.1:127039209|127042698-R	CCATCTGATCCATTGGACTGG
circAC_000163.1:46430752|46431533-F	AAAGAATCAGGTGGCACGAAG
circAC_000163.1:46430752|46431533-R	TCTCCTCATCATCCGGCAG
circAC_000167.1:10418278|10437326-F	AACACCAGAAAGCATCAACGG
circAC_000167.1:10418278|10437326-R	AAGATGGGTTGTTCCCTATGTG
circAC_000158.1:67422933|67458942-F	TATTCAGTGCAGGGCTTTTCA
circAC_000158.1:67422933|67458942-R	GACTTTCCTGGGGTTTGCTG
circAC_000164.1:18971362|18980712-F	ATCTGTGAGTGCCCCTTTCTG
circAC_000164.1:18971362|18980712-R	ATGACAATGTAGGCCACGTTG
circAC_000180.1:2936016|2951681-F	AATAGCTGAGAATCCAGAATCA ATG
circAC_000180.1:2936016|2951681-R	CACTTCCATATCTTCCTCCAACA TT
MSTRG.93118.1-F	TAGAGCGAAGGCAATGGCAA
MSTRG.93118.1-R	TCACAAGCTCAGTCGTGTCC
MSTRG.177599.1-F	GCTCACCAGGTTCCTCTGTC
MSTRG.177599.1-R	CTAAAGGGCTTCCCTGGTGG
MSTRG.185353.3-F	AGGCACCACATTGAACGCAGAG
MSTRG.185353.3-R	GCCAGCACCAACAGCCAGAG
MSTRG.31915.1-F	GTTCCCCTTCCTTGTCTGGG
MSTRG.31915.1-R	GTCCAGGTCCCTTCACTTGG
MSTRG.59961.1-F	AATCCTGCTCCCAAACCCTG
MSTRG.59961.1-R	CTGGGGCCAGATGGAAGAAA
MSTRG.25896.1-F	GGCAGCAGCATGAAATCTCG
MSTRG.25896.1-R	ATCCACATCTCTCCCCACCA
bta-miR-2368-3p-RT	CTCAACTGGTGTCGTGGAGTCGGCAATT CAGTTGAGGGCAGA

bta-miR-2368-3p-F	ACACTCCAGCTGGGACTGTCAGACCACCTC
bta-miR-10a-RT	CTCAACTGGTGTCGTGGAGTCGGCAATTCAGTTGAGCACAAA
bta-miR-10a-F	ACACTCCAGCTGGGTACCCTGTAGATCCGAATT
unconservative_AC_000158.1_13387-RT	CTCAACTGGTGTCGTGGAGTCGGCAATTCAGTTGAGAAAAGT
unconservative_AC_000158.1_13387-F	ACACTCCAGCTGGGAGTTGCTGTTGGTTTAC
unconservative_AC_000171.1_312038-RT	CTCAACTGGTGTCGTGGAGTCGGCAATTCAGTTGAGACTGAG
unconservative_AC_000171.1_312038-F	ACACTCCAGCTGGGGTGGACTTCCCTGGTAGCT
unconservative_AC_000170.1_301545-RT	CTCAACTGGTGTCGTGGAGTCGGCAATTCAGTTGAGAGTGCC
unconservative_AC_000170.1_301545-F	ACACTCCAGCTGGGCACACTCAGATAAAGTAGG
unconservative_AC_000159.1_32276-RT	CTCAACTGGTGTCGTGGAGTCGGCAATTCAGTTGAGGCTGAG
unconservative_AC_000159.1_32276-F	ACACTCCAGCTGGGGTGGACTTCCCTGGTAGCT
miR	CTCAAGTGTCGTGGAGTCGGCAA
U6 RT	CGCTTCACGAATTTGCGTGTCAT
U6 F	GCTTCGGCAGCACATATACTAAAAT
U6 R	CGCTTCACGAATTTGCGTGTCAT
