# Supplementary material for: DNA Double-Strand Break-Related Competitive Endogenous RNA Network of Noncoding RNA in Bovine Cumulus Cells
Source: Genes (Basel). 2023 Jan 22;14(2):290. doi: 10.3390/genes14020290 (PMC9956238; doi:10.3390/genes14020290)
Supplement: Supplementary file 1 [file genes-14-00290-s001.zip › Table S2.pdf]

**Supplementary Table S2. Sequences of ncRNAs**

| ID# | sequence |
| --- | --- |
| MSTRG.93118.1 | TTTGGAATTCGTCGTTTCCACCTTAGGAAG GCAACCGTCTTGGACGAGGGGTGGCAGG AAACTAGAAGGTGGGGTGAACCAAGGAA AGCTGATGTTGTTGAGTTTGAATTCCAACT CAGACCCTTCACAACTGCTAGATGTAATTC ATCCCTTGGCGCGGCCCTTTTGGTTAGATA AGAGACTGAATCTTGTCACCTTTCTTCACA TACGGACCAAGGTTGGACTTTACGAACcag ggttgattttttctcagctgCCACGTGAGTGTTATAATT GGTACACTGCTTTCCACGGGATTGACCCTT GATTAAGCGTTCCTTTTTACAGTTGTTGAG ACGGTTTGGTAACATGAACAGAGTTTGGA AAATTCTAGGCGGATCTTAATCTCTTATTTA ATTCTCTGTTATAGACTCCTAACTGCCTTTG ATCATTCAAAGGAGGTCGAAGTCAGAGTG TGATCTTGGATGGTTTGAGTCTTAAACTTT GTATAAGATGGGCTGAGAGGAACTGTTAC ATGGCGTTACAATATAgagcgaaggcaatggcaatgg caccccactccagtactgttgcctggaaaatcccatggacggagggg cctggtaggctgcagtccatggggtcgctaggagtcggacacgact gagcttgtgacttcacttttctccaattgtccTAGTAGACCATA TATCTGAAGGAAGTGCCCTAAAGGTCTAA ACAGTTAAAGTTATAGAAAACACAAACAG ATGAACTTTGGCTCTCTCATGGATAGATCA TCAGATAGATGGTGTTAAAGAAAGCCATT CTTTAATAGAAATTGCAAAACTAACACTCT TTTTGCC |
| MSTRG.177599.1 | CAATAAAAGCCTTTATATAGCACCTTCTgtgtt ttggtgtgttttttgttgttgtttaatttctaagtcatgtccaactcatttgtg accccaatggactatagctcaccaggttcctctgtccatgagatttccca ggcaagaatactggggtgggttgccatttccttctccaaagggattcttt accactgaaccaccagggaagccctttagtgcTTTTACATAG ATGGTAAACCTCCTTGTTGCCTGAACATGC TGTacaagCTTACTCTCATCCAGACCAGGCCC TGAGGTCTCACTCTCCTGTATCCGCAGGCT GAGGTTACTTGTGGGTGACGTGTTCAGAG GCACTGCCACACGGTAAACCTCAGTGGAT TGTTAGTCCCCAGTGAGACTGTGATTGTGC TCAATGGCGAAGCACGGGCCGGGGTTCCT GCCTCCGGGAACGTCCACAGGCAACATTA GCCTCAGCAATCACCTTCTCTCAGTACAAA GGGATGTAAAACTGGCTCCGACTCTACGG |

| | |
|---|---|
| | GTGGGTGAGCGGGGTCCTCGCCCCACATC<br>TTTCACGATCCA |
| MSTRG.185353.3 | AAACCCTTTCAGCCGAACCCGCCTTGGGC<br>TGGGAAGGCACAGATCTTCAAGAGAGAG<br>GAGGCAGAAATTCGCCCCGGGCTGCAGCT<br>TAGCACAGATGGAAGCACCCCGAGACCG<br>GGCACCCCGAGGCCGGGCACAGGGCCCT<br>GGAGTCGCTTAGTCGCAGGTCTTTAGATC<br>CCTGCGGCTGGGAGCCCCCGGACTGTGCT<br>CGTGAGTTTCCAGGCCGCACCGGCCACAC<br>AGGATGCTGCAGGTACCCTGTGACTCGGA<br>GCTGCTGCCTGTGTCTGGTGTCTGTAGTC<br>AGCACGGAGAGGTTCCCAGGATCAAGAC<br>ACTGTCCTCTGGCTGACACTCGTTTAGGA<br>ATCTGAGCGAGGCCTTAGCAGTCCCGTCT<br>CTGGGAGGCACATGGTGGGTGCCGCGGG<br>TTCTTGGTGCCTGCTGGGGAGGCTTCCAC<br>GGCAGGGGCTCAGGGCAGCCACAGATAG<br>CCTCGCGGGCCCCCACCCCCTCCGCCCAC<br>GGCGACAGGCAGGGATGCCCACGGTGGG<br>TAGCGGGCGGTTAGGAGTGGCGGCTCGG<br>CGACCAGGGGCTGCAGGTCACAGGCCCT<br>GCGGGCGATCACGACCCTTAACCCAGTCC<br>CAGGTCTTCCAAACGGACACCAGGTCCTG<br>GGCTGCACAGCGCCGGGGCCCGGCCCTC<br>GCTGCCTGGGGCATTCCTTCCGCTCAGAA<br>ACCCAGGCCCTGGCCCAGCCTGGGGAGA<br>TGGTCTGGCCTAGCTGAGGGAGCGGGAG<br>TAGCGGGGAGGCTGGCGCCGACAGGAGG<br>CCCGAGGCCGCACAGGCCCCAGCCAGAC<br>CCCTCAGGAGGACATCCCGACCTTTGTTT<br>ACAACGCTCTGTTAGGAAAAGTAGCCAAT<br>GAATAAAGGACGTTCGGCGCGCAGAGCG<br>CGTAAGTCTGCGCCCTCTGTCTCGCTCCTC<br>ACACTT |
| MSTRG.31915.1 | gcggattctttagcactgagccacgagggaagcatCAAAAC<br>AGACTTttaacCATGGTTCCCCTTCCTTGTC<br>TGGGGAGTAGACACACTGCAGACAATGT<br>GGCTATAGCAGATGGTCATTGCAATCAGT<br>GGCAGCAGAAGATGAAGAGGTTGTAGAT<br>GATCTCTTGCCATTGAGTCTTGAAGCTGC<br>CTTTGGTGACACACCAAGTGAAGGGACC<br>TGGACCAGCGCGTCAGACGGTATGGAAC<br>AGGAACAGCTAGATTGGAGTCAAGGACT |

| | |
|---|---|
| | ATTAGAACTGGT |
| MSTRG.59961.1 | CAGCCTTTTCTGTCCTCTGATTGACCCCT TCTGAAGAATTGGAGTCACGCAGTTGTC ACTCATGAGAGAGGTAACACACTCAGGC AAATAACAGatctttCATTTAAGTGACGGTAC TTCAAAGTGAATATGGGTATTCTCAACTC CTcatatcttaaaaaagaaaaaaaaaggaagaattaatgggactg gaaattttaaaacaacagatGTACTAAAAGTGTGATT AGTGCCTGAGTTAGACATACGGGGGAGT GCCACCACTTTATGTAGCTTTGGCTGAGT GCTGATTGTATCATAGAGGAACTGGAGCC CCAGAGTCACGCTGAGACCATCTCTAGG AAATGACTGCTGATTTCCCTTGGAGTGCA AGAGATAAGGAGCTGTTTCTCTCTAAAC ATAGCTCTCTGACTGCTTTGTGGTTAACT AGATGCACTGCTAAGAATTGTGGGTAATT CATTGAAGGATGAATATTGAAACGTGCTG TGTGGTGAGGGAGGAAATCCTGCTCCCA AACCCTGGCTGCATTACTCTGAAGGAGC AGAGAAGTAGAGAGGGACCAGCTCTCTA CATCCTCCACTAAGGGGCCGACACACGT AAGCTGAACTCCAGAATGCAAGGTCAGA AAGAGACTCCCAGTAGTACCACTTGGGC TAAAGTGACATTTCTTCCATCTGGCCCcagt tattgtttttttttttttgtcctccaCAGAGCTACATACTT CAGTCATGACGCCTTAAGCAAGAAAACA ACTTCTTCAGACAGTGGCTGAGATAAGA AAGACAAGGCAAAGCTTAGTCCCGAGTA CAATGGTGTGCATGTTGATATCACTGAGT TATGAACTATTTCCTTTTAATCCAGTTTCA AAAGATAACTTCCTCCATATGCAattaattccac atataactaCAAGGAACTGCATATTTTCGAAC AGTTGTGTCATCATTATGAATGTTTAAAC AATCTCTGTGGTCTCTTTCCCACAAACTG ATTTAACATGTATATAAAGAGGCTTTTGA AATGAGCACTTTACATTTGCTTCTCAAAC TTCAGCGTCTGTTTTTAGTAAATATTGAA GATGAAACTTCCCCCATTCATGCCTGGAG CACTGGCCAATTGCAACTCAGGAATGCA GTTCTCGGGCTTTTCATGAATAAAATTCT CTGGTTGTGAACCTAGTCTTTCTCAAGCT GTGTCAACAGGACCAAAACTGAATGGAT GTGGAATAAAGCACTCATGGCAGTTGGC TGAGCTGGCCACAGATTATCCTAATTAAT |

| | |
|---|---|
| | ATCAAGAGAGTATGAGC |
| MSTRG.25896.1 | GAAATAATAAACTCATTACAAATAAAcaagt<br>cacatttttaaaaatgaaaaatggtatTGTTTCAAAAATG<br>TTAGAAGAGTAGCGTTATTTTTGAGAATC<br>TTAATGTCTGGTTTAATTGGAGACAGCTgg<br>attttcctactttttttctgttgtttcagtTGGTTCAGTGTAC<br>TGTTTCGTTGAATTATGTGGATTATCTAGC<br>CTCATATAGAGGATTAGTTGCAAGAGGGA<br>GGAATGTttgtgtagccttttcagatagtGGGTATtctttg<br>gtgctgctgctgctgctaagtcgcttcagtcatgtccgactctgtgc<br>gaccccatagacggaagcccaccagggtcccctgtccctgggat<br>tctccaggcaagaacagtgaagtgggctgccaatgccttctccgat<br>TCTTTGGTACTACACAGACGTTAACAAG<br>GGGTGGTGTTATGAAGGTTAGCTGCTATG<br>GAATCTGAAATGTTATCAGGGAGGTTTTC<br>ATACTTCGTTACATTAGAATCCATTGGTCT<br>ATCTCTGACCAGATCTTTTACCCATGTCT<br>GATTTTTTGACATTGTGTGATAGATAATTT<br>GGGAAATACTGGTTCATTGAGTTGTTGCC<br>ACATTCTATATGacagtataaaaaaataatattaccaCA<br>GTCATCAGAAAAATGGCTAAGTTTTGGG<br>AAGCTGTCAAACTCATGGTGGCAGATAC<br>AATTTTGCCcaaattctcattttctcttggAAGCTCAA<br>ATTTTATCATTGGCAACAAACACTGTCAG<br>TTGTTTCCCTTGAAGTGACAGGCGTCCTC<br>TGTTTCATTTTCTAGAGAACGCCTGCCAA<br>GTGTCAAAGTCTGAATAACCATCATTTGT<br>GTATCCTTCATTCTTTCAAGTAAACATGAT<br>GTTCCACAAAAATTTTGCAATTCAAATAA<br>GTGCTTTACTTACAGATAACCGTTGTCTT<br>CTGTTTTATATGTACTTCTTATTTTATCACA<br>CAGAATGTTAAAAAGATATACTTGAGGAT<br>TGggatttcatttttcactgtgtCATCAAGGACATTCT<br>TAAGTAAACCTGGCActtcttcctttgctgtgcagtgT<br>AGAAGTGTGTGGTGGTGAAAAATCTCAC<br>GTGGTACCTTTGGCTTGAATCCTACTGGG<br>GAAACAGCAGTTTGACCCACCGTTGCTC<br>TTGCACCATCAGTGGAAAGGCTGGCACA<br>GTTAAAAAGGCAAATCACATCTTAGTTTT<br>GTTATGGAAATAGTTTCACCTAGTGGAAC<br>CCCTGAAAGGGCCCACACTTTAAGGACC<br>ATTGCCTTCAAGTTCACCCAAGGTTTCTT<br>TCTGAAGCTGCTGAAGATCAGGGCTTCA<br>GGAGAGCTAGTCACTTAATTATGTTAGAA |

| | |
|---|---|
| | Tgtacttaattgctcagtcatgtccaactctttgcgaccccatggact<br>gtagcccgccaggctcctctgtccatgggattctccaggcaagaat<br>actggagtggcttgctattcccttctccagaggatcttcccaatcca<br>gggattgaatgcgggtctcctccattgcaggcagatttttactgtct<br>gagccaccagggaagcccctaattacGTTAAGCCTtcaac<br>tgtttccacttttcagtAATTTGCAGATAGACCTCCT<br>TTAGCTTTAGCTGCTataagctgttttttttccctgaggt<br>tAGCTAACTTCTTTTGCAGTAACTGTGTTC<br>TACAAGAGCTGCCCCCTCAACTTCTATTG<br>GCCCTCCCTTCTCATTAGGTTGGCGTGTG<br>GACCTTTCTTTCTCAATAGGGCTTTACAG<br>AAGTTCCATGCTCCTCCAACTTTCTTTAG<br>GGAGACAGTTTGGGATTTATCTGTTAAGA<br>GAAAATGTGTCTGATTCCTACACTCCTCA<br>TTCTGTTTGATTCCAGCCAAGTCACTTCT<br>ATCTTCTTATATAACTATTGGGCAACTAAA<br>GTGATCTCATCAGAGATCTTGTGACTCTA<br>GACTCTGAACTGTTTCTGATGTTCTGAGT<br>TCCTCTGGTGACTGAGTGAAACTCTCAgat<br>ggaagaggaaaaggaaactgCAGATCCCAAGAGCC<br>ACACACTCCTGTAATATAGGAGAGTGAC<br>ACAGTCAATGTGTAATTAATAATTACTTTC<br>TGTAGAAATAGATACCTTGTCCAAGTTGG<br>TTTGGTCTGGCCTGGCCTCTGGAGACCT<br>GGTGGTTTGAGACAACAGTCATAGTTTTT<br>CTAGCATAGGCTGCTCATCATTCACTCTC<br>ACTTAAGGAAGAAACTTGAGGCAAAACG<br>CCCCATAAAGAGTGGAAGGCAGAATGAG<br>ATAGACAGCTTCTGAATGTTGTTTAAAGA<br>TGGCGGGGCAGGACGTTGAGATTCCTGA<br>GACTGGTTGCTTTAGAAGAAGAGAGGTT<br>GGATCCAGGGAAGGCAGGCAGGATGCTA<br>CTACAAATGCAGGCCTGCATTTGGTGAG<br>GCAGGTGAGGGACTAGGAATTGGACAGA<br>GCTAGCCACACAAATAGAGATGTTTGAGg<br>ccattttgtttatcttcagAATGCTCTGCAGTTCTTTA<br>CTTTTCTTATcccctcaggggaaaaaaaaattgtttcattttt<br>aacagcTTTGTGGAGGTATAACTGACCTAGA<br>AAATATTCATTACCCTCaagtcaattatttttaaacttt<br>attctagGAACCCTTAACATCTTATGATATTTT<br>AGGGGGAGGAGACTTGGGCAGCGTTGCT<br>GAGAAGAAGGCCAGACTAGGGCAGTGAT<br>CTTCCAACTTCTACTAACATAGCTGTGCA<br>TTTATCTTTTACAGATACCCAAGATTTCCC |

| | |
|---|---|
| | TATAAAAATCATCAGGGGCTTATGAAGTA<br>TATTCATATGAAAAGAGAACAGTGGTAA<br>AAGATAGCCTTTGCAAAACATTAAATTAG<br>AGGAATAAATACACAGCAGATCTTCATGG<br>GTGGGATGGGGCTTGAGTTGGGCCTGGA<br>AAAGGTAAATGCCGGGCCACTTGCGTCC<br>AGTGTTCTGGCACTGAGGATTCATGCGG<br>AGGGATGCAGAGCAGTGTGTGCGGGAG<br>GTGAGTGAAGACAACTGTCCCCTTTACC<br>CGCTGCACACACACTAGGGAGTGACACT<br>TGTAGAGACCTGAAGGTCTGAGATGGCA<br>GGATGCACACGAGAAGCTACACGTCGGG<br>TGACATTCAGGGGGCAGCAGCATGAAAT<br>CTCGTTTCCTTATCCCTTTCTGCCATCACT<br>TTGGTGAACCTGTTGTGCCTCATTGGTGG<br>GGAGAGATGTGGATTCCCATAAGATGACT<br>ACGTAAGTTGGAACGTGAGCCCATTGTG<br>TGTACATGACACACACTCAGGTGTGTGG<br>TCTTATAAAATCACTGGCAGGGTCTGACC<br>CCTCCCTTTGTGCTTGTTTCCCATAAGTG<br>CGCAGAGTGTAGCTGTGCCCTGCACAGT<br>ATTTCCATAGTTTGGACTTGGTGGAGGTC<br>CATTTTCTGTTACCTTGAGTTTGCATTTAC<br>TTGCATCAAATCTGGTTTTAGCTGTGATG<br>CTGTTTTTCACCCACTATCATTTTGAATAG<br>TGGCAGTGATTTTTTGGTACTCTTCTTGA<br>GAAGGACTCCCACACAGGTCAGTCTTTG<br>CAGGAAAAGAGGGCTATATCACGTGGCT<br>TTTGCAGGCTTTTTGTTGGTGGAAAACA<br>GCAGCAGTTCCTGTAACTATAGGCAGGA<br>ATGAGAATCTTCCTTGGCCATTGTTCTCC<br>TATCAAAATACAAAGCCCTCTCCCCATTG<br>TCATTTGTGTCTTAAATAAAACTTGAGCA<br>AGAAAATTCTCTGCTATTTCCTTCCCTTTA<br>GCCACTGTGAAAATACTTGATTAAGATGC<br>CAGTATTTCATTCAGAATTATCCCAGATGT<br>ATGACTTCTTATAGAAGAGTCTTGTGTTT<br>CTCCCCTGGATGCTGTCATAAAAAGCAG |
| MSTRG.177599.1 | CAATAAAAGCCTTTATATAGCACCTTCTgtg<br>ttttggtgtgtttttttgttgttgttgtttaatttctaagtcatgtccaactca<br>tttgtgaccccaatggactatagctcaccaggttcctctgtccatga<br>gatttcccaggcaagaatactggggtgggttgccatttccttctcca<br>aagggattctttaccactgaaccaccagggaagccctttagtgcT<br>TTTACATAGATGGTAAACCTCCTTGTTGC |

| | |
|---|---|
| | CTGAACATGCTGTacaagCTTACTCTCATCC AGACCAGGCCCTGAGGTCTCACTCTCCT GTATCCGCAGGCTGAGGTTACTTGTGGGT GACGTGTTCAGAGGCACTGCCACACGGT AAACCTCAGTGGATTGTTAGTCCCCAGTG AGACTGTGATTGTGCTCAATGGCGAAGC ACGGGCCGGGGTTCCTGCCTCCGGGAAC GTCCACAGGCAACATTAGCCTCAGCAAT CACCTTCTCTCAGTACAAAGGGATGTAA AACTGGCTCCGACTCTACGGGTGGGTGA GCGGGGTCCTCGCCCCACATCTTTCACG ATCCA |
| MSTRG.155681.4 | ATCTTGGGATAATCACCTACTAGCTGCAC AGATGTTTCATATTCTGCTGAAGGAAGCA AGTTTCCTTGGAATACTTGAGAATTCCCT CATCATTTATTCTTCAGAAATCGAGACAC AGTGTGGAAGAGAGGATGCATGCCTTTG CTTTCTGGCCTCCCTGGGATCACAAGAG GAAAGAGAACACAGACCGCTATCAGTAC AGAAAGTATTTTTATAGAAGGGAGGAGG AGCTTTCCACCCCGTTTGTAAGACCTGGT CTGAAGGAGAGGGGCCAGACCCCAGGG TCCTCTTGAAGgactaatattttaaataagGTATTTT ACCCACGCATATGCACATATGAACGTGTA CTTGGATTGTTTTGTAGTATGTAAAGTTA GCACCAAAGACTAGCAGCGAATTCAATC AGGAAGATACTGTGAAGTAAACTACTgtta atctataaaatggaatactacacagcttTGACAAAGATG ACAATgaacagtatgtgtgtgtatctgaaatatgtatattttgaaa tgtgtgtgtgaaatatgTATGGTCTTGAGATTCAGT TAAATTAATTATGTCCTTTTATACCCATCTT TAACCTTTCTTTGTTGCAAGTGTGCTACA TATGTAAAATTGCACAAAGCAGAATACAC AGCTCAATGAATGAGAACAAAATGAATAT CATGTAACTGCTAATCTGATGAAGAAAGA GAACTCTGTCtataccttttctttttgtctacaAGCTCAT GGATAATTTCCGTCATTCAAAGCTTCTGT TTCCTTACCTATGATTGGGAACACTGAAA ATCGTTAACACCACTGTTCTCTCCTTTCT GACAATGTCTTTGTGAGAATCACATGACT TAATGTTAATGCTGTTCTTCTAAGTGACA GAGAGCTCTGCAAATGAGGATGAAGATG GCCATTCAGTCTGATAGATGTGTGAAGCC TGCAAGGACATCATGCTTGCAGTTGTCCT |

| | |
|---|---|
| | TAAGCCTTCCCTTCCTTGGGAAGACAGA<br>ATGTAAGGAAAAGAGTGGTATTCTGAAC<br>GAGACAATCTGTTGGTCATCATCCTTCCA<br>GTAAATTTGACTTGAAGCCATATAGTCCA<br>ACCTGTGAATGAGAATGCACAAACTTCC<br>TAATTGTTCTGAATCTCTGACTTTTGGCA<br>GGACACTGCCGAACTGGAGCCATGCAAG<br>CTTTTGGGAGACTTTCCCAGAGCCGTGA<br>CCTTGTAGGAACAGTCTTCAGTGACTAG<br>GTGAGGGGATCCCTTACCAGTAATTGCTA<br>GGGATGTGAAATAATACATGTGCCCTGCC<br>AGGTTCCATTCTAGCTATGTGACCTCTGA<br>CGAGTCATTTAACCTCACTGAGCCTGAAT<br>TACCTACCTTCTTGAGGATATACATAACCC<br>CAACCTACCTAACCCTAACCTCACAAGG<br>TGCTGTGAGTGTCCCTGTGAGATGGTTCA<br>TATAAACTTCTCACTGAGGTTCCTCACTT<br>GACCAGTACTCAGAACTGTTTGCTGTGTA<br>AACTGTCTCGGAATATCTAACTGGGTCAT<br>CACGGTGAGTGAGACTGCAGCACGTGGG<br>CCTATATGTTTCTTACACATGAACACAGG<br>CAGCCAGGTATGGACGTGGAGAGTGGGG<br>TGATGGTTTTCTGACTCAGAAAACCACA<br>ATTTAGCCCTCAGCTCTCCATTGCTCCTA<br>AGTAGGTCTTTCATGTATGAAGTCACAGA<br>GTCTTTGTACACTCAAGATAGTGACACAT<br>GGGGCTTGCAGTGCAAAGACCAGGATCT<br>GTCAGgcttatatagaagaaataagagtATGAAGTGA<br>ATGCTAAGGCTTCATGTTTGTGTGGGTAG<br>GATGTGgagagcagaggggagagagagaaaggcacaG<br>AAGTAGAAA |
| MSTRG.69684.1 | AAGCAAGAGGCAAAGTATTTTATAGATTA<br>ACAGAAGATAAGCGCGTGTATAGATGAAT<br>ATGCTGTATAGTTTACATGTACTTGGATAC<br>ACATGGTGCTTACGCACATAAACATATGT<br>ACACATAGACTTGTGTACGCATGGAATAT<br>TTTTGGAAGCTTACCCAAAGCAATTCAA<br>AGCAGTGCTTCTCTCTGCGGGGGAGAGG<br>GATCATAGAAAGAAGGGAAGCTTACATT<br>TCACTGTATGTATCTTTGTATTATTTGATTT<br>TCTATTAATTTCTATGTgacacttttaaataaataaaaa<br>acttcttacttaaaaaataaaacaggaatggCACACAGAC<br>TAGACATTCTCTAGTGAGGGAGTACACTC<br>GATCACACAATTTCTGGAATAAAGGGGG |

| | |
|---|---|
| | TTTAGATGTCTTAGTTTGCTCACGGCGGG<br>GAGAAATGATTGCTATTTGTTATTTTGGTT<br>CCCCCTTTGGAGTTTACCAGTCCTCATAC<br>GTTGTGCTTGGGGATCCACCTCCCACCTT<br>CGTCTACATGTCCGAGGTGCAGCTAACCC<br>CGCTGCCAGATCCAGAGTGTGGAGGCTG<br>CGAATGGTTATTAACACAATCACCTTGCC<br>TCACACACTCTGTCCCAGGTGTGACCTC<br>CAGGGATGGGCTATGAAACAAGGCAGAC<br>CATGAGGGCCTAGAAACCTTTGATCTGG<br>GAACCCTTAAACCTGTTTAAGCTTCCAGG<br>AAAGCAGACTCTGCTGCAGGACCCAGTG<br>CCCTAATAAGGTGAGCCTGAGACTGCTG<br>CTGTGGCCACCTTGCAGAGCTGAAGATG<br>gaaatctgaaaaacagaagTCAGAGCTGAGCAGCA<br>GAGAGAAGCTAAATGACAGTGTCAGCTC<br>TGAATCCAGTGGGCTTTGAAGCTAGCTA<br>GCTTTTCCTTTGGACTTTCcaactttttaaacaaata<br>agttACCATTTGCTGCTTAGTTTAGTTGATT<br>TTTCTCTCACGGCCATAGAAATATTCcaatag<br>agaaactgaggcagacaaAGCCCCATTTCAACCTC<br>GAAGTCTGGTGTCATGCCCAGGGTCTAA<br>GTTATAGTGTTCCAACTCACTGACTGGGA<br>CACGGGACACGGGATTCCAAAGTCAGGT<br>GAAtgggggaggaaaaggaaaaggaggctgGTGAGA<br>CAGGAAGAACAAGATGGAACAGCAGGT<br>GGAGGTGAGCTGTGAAGGCTGGTTGAAG<br>ACTGTATTTCCAATAACAACCTATGACTAT<br>TATGTCTATATAATAGTTATATAGACTATTT<br>GTCTCTTCTTTAGCGAAATTATGATAGTAA<br>TAGTGATAGTCTGGGAAAGAGAATTCTA<br>GCATCTCCTGCACATTGACGATTCATGTC<br>CTATACAGATAATTACTGGCTACCTACTAT<br>ATACTACATATTATTCCAGGTGCTGAACAT<br>ACAGCAGTAaacacaataggaaaaaaaaaatctcctcca<br>TTCATGGCGCTAACAATCATATTTAgtcttaaa<br>aacataaaatgtccCAAATGAACATTTAATGCTAT<br>AGATGTGCCATGACTGTGCTAGATGCTAT<br>TATGACAATTACTTAACCCTAATACAATTC<br>ATATTTCTGCAAagtaggtatttttattttcattttataaatg<br>aggaaaataaagtctgacgagATTATGTGACTTGTTG<br>GAGGTCACGCTATCTGTAAATCATATAATC<br>TCAAGTAAAATCTAGCTTTTGCTTTCAAG<br>TATGTTATTTTGCTCACTAAACCACAGCT |

| | |
|---|---|
| | CTATTTAACTACCATATGACAGATGTCATA<br>TAGCAACTCAATGGGTTTACTCAGATTCT<br>GCAAAGTCCCTAGGTATTATGTAACTAAC<br>ACATGAATAAAGAACTTTACATGAAGCG<br>ATTTATTATCTTCTCTCGATATGACTACTG<br>CTCATTAGGTTAATATCCAGGATGTGATCT<br>CATATCTGCTTTGCTTATGAAATCTTGCAG<br>TGGTATGCTAGAATTGGCTTGACCAACTC<br>ACAGGAACtgttaaattttcagaaaatttgcAAACCAG<br>TTGACTTTATGTTGATAGCTTGAAAGTGA<br>TCATAGTGTAAGTATTTACATcaagaaaattaaca<br>aatacTACAAATCAGGGTTttgtttcaatttcctttttttc<br>ctggacggTCAATTGTTAAGCATTTACTAGGG<br>CACAACTGTATTTAAGGTCCACCATTTAC<br>TCAATGGAGAGAGATCACAGGAATTATTC<br>TgttagaaaatattcaaaatactggAAAACTTTCCAAC<br>TTGaaagatttttaataataatgatcccaggtggtactagttgTA<br>AAtgtgggagatgtaagagatgtaggtttgatccccaggttggg<br>aagatcccctggagaaggggatggcaacccactccagccttcttg<br>cctggagaatcccatggacagaggaacctgatatgctacatagtc<br>catagggttgcaaagaggcagacacaactgaagccatgCACG<br>TAAGAGATACCATGGATACATGTAAATAT<br>CTTTCCCAGTGCATATTGGGCCTAAAATG<br>GTCATTATCCCAGACTTTCAGAACCATTG<br>CTTTATAAGCTTAATGCCATAAGATACTTG<br>TAATGGGTTGAATATTATCCTCCCTAAATT<br>CAAGTctacccagaacctcagaatgtggtcttatttggaaatgg<br>ggtcttCGCAGACATAATTAAGATGAGATCA<br>GACTGGATTACGGTGGGCCCTAAACACA<br>ATGACTATTGTCTTTATGGGAGAAAAGGA<br>gattcagacacagaaagacaaagggGAAGTCTggggaag<br>acagaggcagaaactggagTGATACAGCTACAAGC<br>CAAAGAATGTCAAGGATTGccagcaaccaccag<br>aagctacAAAGAGGCAAGGAAGGCTTCTTC<br>CatagagccttcagagggagcatggccttGCTGACAACT<br>TGATTTTCAACTTGTagcttccaaaactgtgagagaG<br>TAAATGTCTATTGCTTTAAACCACCTAATT<br>TGttgtaatttgttatggcagctctaggAAGCTAAAACA<br>ATACTTGTGCAAATTATCAGATGCATAATG<br>CCTAAGAGGACACAGTTAAAGAACCTAA<br>AAAGAATTTGATAAATTCCTTTCTGGCCT<br>CTTGCTACCAATCAATATTTTCATAAGAA<br>ATCATGTGTAACATCTAAAAGTAAGCTTT<br>TGACATAGCATCTGTTTTCAATCTCCCAC |

| | |
|---|---|
| | CTTCGTAACTTTAGAAAGAAGGACATGG CATAAATAGAGTGGAAGCAACATTAATTA GTGAAGTTGTGCTGAAAATAAATTAGTG ACTGGTGAAGCTTTTGTGTAGCTCCATGG TTCCATAAACCAATCAACAAAACTGTCGT TCAATACtcaaattaatgtattttagaaTACAAAAAAT AGATGCAAATGATAACTTTTAAGTGTTTA CTCTGGGCCATACACTGAACAACtcaatttata catatatcctatGAAGCAGGTATTACTTATCTCA ACtttacaaaggaagaaactgagcCTTGGCCAAATTA AATACCAATAACATTGTCAGTAGATGGCA AAACTAGGAATCAACATCTTTTCAGCTAT GAACTGACTGACTTCAGGGCCAATACTT GAAAAACACCTAAAATTATGGTCCATAGG CCACCTGAACTTTACCCTAGACGAGAAC CTCTAGGTATAGAACCAGAAACGTGCAA CTTTATTATCTCCTTTCAGGACTTCAATTA GACAGCTTTAATCTTTCTTTTGTAATCTGT ATCTCACTCTTTCTTTCaagttttccttctctctgtgct ATAGTTCAGGAAATTActtcagttctatttttaaatttgg taATTCTTTCTCCCATCTCTAATCTTAACTC ATTCACTGAGTTAAATCACTtcagtgatatttttaat ttagaattctgattttttccttaacagtAGGTCTCAAGTAT TTCCCATGTTGTGTAATcctcataatttttaaacagtat tCTATTCCATGGTTACACCAtgcttatctattcatcta cctGTTGTTATATCTTGTCATTATATGACATG AGTCCTTAGAAATTATCACAGAATTACCA TACTAATGGACCCACAGTGATTTAGCATG AGTGGAGTGGGCCCCTGACATCTCTGCA GTTAAAATATTCttgggtgattctgatgcacatcCATG GTCGAGAACCATAGATTTAAAGAACCCC TATAATTAATAGTATTCCCATGATGGTGAA GAGTCTGGACTATTAAATCACACTACCTG AACGGAAATCTTGGGCTCCATGCCTCAG ACCAGGGTAAGCTTTTTAACTTCTGTGgac tttggtttcctcatttatCAAATAAGGATCACAGAAC CTACTGCAGTGGGCTGTGATgactaaatgagatc atgtaAATAAGCACAGCAAGCCATCCTTACA ATCGTTCTTCTGGGTGGATGGGGACCTAA GGGTGAGAGGCATTATCTGTGAAAGTCT GCAGTCCACCCTCATGAGAGCTCAGCGA GAGATGGAGACTAGAGGACTCTGGTCTG AAATACACCTGGCGGGTGGGGAGAGAAG GTTGTGACTGCAGGGTAGTTCGCAGCCT |

| | |
|---|---|
| | CAGCTCCAGATCTGCTGACAAGTTGAAA ATCGGCTTGTCTGTGAGGAGGAGGTGTT ACACCTCTGGGGGCCATAGTACTGTATGT TCTTACCTTATCAGGAAGAATTACCAGTA CGTGACCTGATGAGACCTGAATTCTTAAA TGTAACGAGAAAAGGCTATCTAATCTTCA AAACCTGGTGTTGGAAGAAGCCATCCGA GATTCGCTTCTGATTAAATAATGGTCCCA GGAACAATAAGGTATTTAAGGAAAAGTG GATGAGAGAAAAATAGGAATAGTCAACT AAGGAAGGTCACCAAGGGGTGATGGAC CAACACTAAGCTTAAGGTTCACACTGTCT GTAAATTCAATAAGCCTATTAGGTTAATAG TTACATTTTTACTGTACTCCTAATAGTTAC ATTTCACAGCTATCATTTGTTCTAGAAGTT TCTTCTCATGAGTAGCTTTCTCTAGTTCAT AATGTATACTGTGACTACATACCATAAGTT TAGAgggctgtttttaaaaacattctacaAGGTAATATT TTGTTCTGGAGGCCCAATGaaaaacttttgaaaga aaaatatctagAAGTCTAGGTTATGGTTACACA TGGAAGGAGTTTTGTCTGCTTGCCAAGA CAGGACCTGACAcagagtgctcaataaatatttgttaaat gaactgttttctctcttttggctTCAGTCACTAATGGTT AACTTCATGCTTAATCAGCATCCCTGTGG AAgagcaagaaaggaaaagcagTGAAGCCTACTCC CTTTTAGGGCTATTGCTGCTATGTTTCCTC GCTTCTCCACCTGATACCCAAAGAGAAG GAAGGCTTTCCTGGGCCTTGTGTTGAGC AGCCTTAATTGTTTCCACTCACTAAGAAC tggtaaaaatacaaaaatgtactttttaagaagtacaaaaataaaaa aaaaaatttaaaaaggcagcctatatatcttttttgttagcaatttttaa aatcagctgcTGTCAGAAAGCTAAAGTGATAC ATACTCTGCAGCACTGGTTCTTAACTGTG TTTGAATCCTTCCATTTGAAAATCTGACG GAAAGTTATATTTATCTCCccagaaatatgtgtgtgt g |
| MSTRG.24637.1 | TGGAAAACGAAGGAAAAATAGTTGGTAA GAGAAAAGAGTGGAGAAGAGATTGAAA ACATGAGAAAGACTTGACCTCCCTTGTT GactttgaagacagaggaagggggCCATGAACCAAG GAATGCAAGTGGCTTCCAGAAGTTGGGA ACAACCCTTAGCTAACAGCCAGGAAGGA AATGAGAATCTTAGTCATCAGTGAAAGG AACTAAATTCTACTAGCAACTGGAATGAG |

| | |
|---|---|
| | TAAGGAAATGGGTTTTAATCCTGTCTAgg gattttccaggcaagagtactggagtgggatgccattgccttctcat ctACTGAAGGAAAATTTGGGATTCTGCTC TCAAAACCTAAAACCAGGATAAACAGAA TCATGGAAAGAACAACAAACTGAGAAA AACTACAACAAACCCAACCCCAGATTAA AACAAAGTAATCCATCCTCACTACCCAAG AAGCCTGATAGAAGAAGATGACTACCCT TTTCAGCCTATTAATACTGATCTCAGGTG GCCTCTGTCTGCAATGGCTTGGGGCTGG ACTTGGATTTCCAGCCAGACGTGGGCTG GATTGCAGCAGTGAAGGCACCAGATCCT AGTCActtagaccagtggtcagtgacaagggccctggcccttt ggctttgGAGAAAAGAGtttccacaaagatggaaagtag tgaagcaagtaaagtATTTACTAGGAGGAGAAGA GTACAGTACATGTGGAATAGACACGCAG GGAAAGTCACTGAGTTGCACCTTCATGG CAGTTTAAATCACTAATATGGGGCATATTA GTTTCATATTTCTTTCAGGTTTCCTTTGGT CAATCATtgtgatttgcctggttcacagtccatatttggtatatct cagaatcctcccatgtgtgcacatgcatctcttagccaagatggatc ctACTAAAGAGGCGTCTGGGTGGAACATC CCTTGACGTGACTCCCCTTTGGCCTCCAA GTGGCCCTTTCTGCACATATGTGGTCCAG GAGATCTCCCAACTTCTGGAAATGAGAA CTATGTGGTCTGTGCAGggtccagcctcctcccttaa ttgTCCCACTATTCTTATCTTAGAGTTTTGG TCAATAGAAAATGAATCTCCAATTTGCTT TACCCTGGTGGGAGGGGGGCgcccatctgcctc ctgcctcaatATTATATGCTTCAGTC |
| MSTRG.197583.1 | CTCGGTTACCTGGACATCCCAGGCAAAG GCCCTAGGCCTGTTGAGTATTCGTGTCGC GTAAACGGCTCCAGCCGGGCGCGTGCTA CCGCTTGAACACAGCCGAATGTGCGGAC TCTTTCGCGGGGCCCGCCAGAGATTCAG CTCCCGCCAGCGAGGGGCCTTTGAGGAC TTGGACCGCGAGTCCGTCCCCCCAACGC CGGGAAAATAGGACTGAACCCGGGTTTC TAACGCGCTCCCAGGTGATTCCGGGAGT GCTGGTCCGAGACCACACCTTGAAGGAG CGCGGCGTGAGGAAGCCTCTCCACGCTG AGAGCTGtgccccttttcctccttttaCCGCTTGCTTT CATTCAGTTCAGGATCTGATGGAAGCAG GGCTAAGGGGTGCGCTCCCCTGCAAAAG |

| | |
|---|---|
| | GATTGCAGTGCAGGGTGAACTCCCGCCG<br>CGCGTCCGGCCGCGCACCTGCCTAAGAT<br>GTCACACCTGGGACCAGACAAAGCAGCC<br>CCTCGGAGCCGGTAGCTGACACTCTGGA<br>ACCACAGAACAAGCAGTCTGGCAGCTCT<br>CAAACCTTCTTGGGGCCCGCTCCTCACTC<br>ACCAGGCAACAGGCCTCCACAGAGCAG<br>ATCTGAGCCACCCAGAAGCACTGGGCGG<br>GCGGCACGTCCTCCTGCTGCTGTTACTGtg<br>acagctgctgctgccgccgccgagGCATTTGCTAATG<br>ACTGAGCAGGTCTGGAGGCCAGCGCCGG<br>GAAGTGAATGTCGCCCAACCCGTTTGAG<br>GCCAGGAGGCTTTGGCGTTGCCCAGGGG<br>TTTGGCCCCTGTTGGAGTCAGGTGATCTT<br>GATGAAGATGCTCAGAGAGGAGGGTTCC<br>CCCACCCAAGGAGGAGTACAAGGATGGG<br>TGCTGGAGACAAGCAGAGGGTCTCAGCA<br>TGGGGGCAGTGACCCAGGACCCATCCCC<br>AAAGATGAGTCTCCAC |
| MSTRG.49414.1 | gaaatgtttagatagcatcactgactcaatggacatgaatttgagca<br>aactctgggagatagtgaggacagaggagcctggcctgttgaagt<br>ccatgggatcctaGGAACTGAACAAAAGCAATA<br>TTAGGTCTGCATCAGAGACAGCAAATGC<br>ATGACACAGATACTgcccctcctttctcctctggcCA<br>TCAGAGACACTCTTCACTGACCTCAGCT<br>CTCTCTCCTACTGAGCCCCACTCAGATTT<br>AGAATTTGTCTCAGGACAGTTGTCTGGG<br>GTGCCACTAGTGATCAAACAGTGGCACG<br>TGGGAGCACAGTGCAGCGGGCAGTAACC<br>CTGCCTTCTGACTGAAGAAGCTACAGGG<br>AAGGTCTTACTGAGGAACTGCCTTTAAG<br>Gaagtcatgggacttccctggcggtccaggggttaagactccgc<br>cttccagtgcagggggtgtgggttccatctctgg |
| MSTRG.197668.1 | CAGAAACAGATTAACAATAAGAGCTAAT<br>ATACAGAGGACTGaggttaaaaaaacagaaaagctaa<br>AGTAATAACGAACAtttcttaagaagaaaaaaatggga<br>agggatgaatgtttgaagaaaaaaatgaccaaGAATGTCC<br>ATAGCAGGGACttacttccctggcggtccagtgattaaga<br>ctccatccTCCCAAcgtgggggacatgggttcaatccctggtt<br>cagttcagttctgctgctgctgctgctgctaggtcgcttcagtcgtgt<br>ctgactctatgcgaccccatagatggcagcatgtatgttACAC<br>ATCCAAAACTTATATGTtaa |
| MSTRG.7914.1 | AATCAATCAGGACAAACAGTGGTCCGGG<br>CACGTACTTGATGAGCTCCTTCTCCCGCA |

| | |
|---|---|
| | TCTCTAGCTGCAGCATGATGGCACTCAGTTCCATGTACAAATTATTAGCCCGCTCAAGTTTCCGCTCATAGTGCTCACGAATATCCAAAGCATGCCTatcaaagaaaacacaggagCAGCGAAGCTGATGAATGTGGGGGAGAGCCAGGGGTCAGCTCTAATGAATTGTACAATTACAAAGCAACAAAAAGGGAAAACCATGCAGGTGCCTGGACATATCCGCCATATAGgtggattctttactgccgtgccacctgggaagccc |
| MSTRG.112314.1 | GGCGCGAGGCGGTGAGTTCGGGGATTAACCTGCGAGGAGAGGACACGGGGCCATAAGACCCGAGAGGGATGGGGTGTCCGAGGCGGTTCGGGGATCGCCGAGGCGGGGCAGCCTCCCTGCTTTTAAAGCTCCGCGCGCTCCTTTCCCGCTGGGCTCCTGCTCCCCGCTCCCACTGGCGCTGGTTGCCGTCCCCCTGCAGGGAAAAGACAAGATCTGGGTGAAAAAGGAATCCcagcaggaagggagggaaggcagggagaCGTGAGAGCCAGGTCGGCGTCACGAAATGCTGCTGCAGCCCCCCGGGTTTCCCCG |
| MSTRG.146080.1 | cgtctatggagtcgcatagagttggacatgactgaagtgacttagcagcagtagcagcagcagacagtaaAGAGAGATCTGTTGcgtgttttaaattttgttctaaATAACCCAAAATGTGGATAATTCATTGTTGACTGATTTTAGTTAACCAGGTTTTATCTGGAGAAAATGAACATCCTTTTAAGGACAAATCTCTTCAGTTTTGGCCTGCTGattcaaataaagatttttccttcaagttctctgtcctgtttttaaaaaatatttttcctttctaaattaaCTATATCAACTGCTACAAATAGGAGTTTGATAttgatttattcttatttatgcatttaaagtGCATAATTTAGTTAGAAAGTCATGTCATGCCACAAGCACTGTTCAATTATATAGGAaggatttatatattcattttgtcTATGTTATAGTTAAAAATCTATGTGTTTTTTCTTTACCTGAGAAAGTAGCTGGCCCAGGACTCAGTAAGAATGCCATTGGCTTTGTTACTTCTTTTGTAGAGTTAGGAACTTTGAGTAAATCAGTGAGTCTCATACCTTACAAAAGTTTTTTTCTGCaaagatgatttgggagaatggccttgaaacatgaataataccatatatgaaacgagtcgccagtccaggtttgatgcgcgatactggatgcttggggctggtgcactgggacgacccagagggatggtacgtggagggggggaaggaggagggttcaggatagggaacacgtgtatacctgtggcggatgcatgttgatatatggcaaaaccaatacaatattgtaaagttaaaaataaaataaaatatattaaaaaaagaaaacaacctttCTTCTAAAAAAAACTTCTGCGTGGAACCG |

| | |
|---|---|
| | TGTTACATAAAGTGGAGACAAGTATAGA ACCGTTTAGCTCAAGAGTGGGCAGAGCC CC |
| AC_000159.1:127039209\|127042698 | CCATCCAGTCCAATGGATCAGATGGGCA AGATGAGACCTCAGCCATATGGCGGGAC TAACCCATACTCGCAACAACAGGGACCT CCATCAGGACCGCAACAAGGACATGGGT ACCCAGGGCAGCCATATGGGTCCCAGAC CCCACAGCGGTACCCGATGACCATGCAG GGCCGAGCGCAGAGTACCATGGGCGGCC TCTCTTATGCACAGCAGATTCCTCCTTATG GACAGCAAGGCCCCAGCGGGTATGGTCA GCAGGGCCAGACTCCATATTACAACCAG CAAAGTCCTCACCCCCAGCAACAGCAGC CACCCTACTCCCAGCAGCCACCATCCCA GACCCCCCATGCCCAACCTTCGTATCAGC AGCAGCCTCAGTCTCAGCCACCGCAGCT CCAGTCCTCTCAACCTCCGTACTCCCAGC AGCCGTCCCAGCCTCCCCATCAGCCGTC CCCGACTCCATACCCCTCCCAACAGTCCA GCACCCAGCAGCACCCCCAGAGCCAGCC CCCCTACTCACAGCCGCAGGCACAGTCT ggaatggtcgtcgtcgttggagtcgAGCCAGCATCCTC GACGCTTTCCCAGCAGGCTGCATACCCTC AGCCCCAGTCTCAGCAGTCACAGCAAAC TGCCTACTCCCAGCAGCGCTTCCCTCCAC CGCAGGAGTTATCTCAAGATTCATTTGGG TCTCAGGCATCCTCAGCCCCCTCAATGAC CTCCAGTAAGGGAGGGCAAGAAGATATG AACCTGAGTCTTCAGTCAAGACCTTCAA GCTTGCCT |
| AC_000163.1:46430752\|46431533 | GTACTTGAAGTTTATCGAGTTGCCCTTGT CTCAGAGAAAGTTTTGTCAGAGCTGTCA GCAGTTGCTGCTGCCGGATGATGAGGAG AAGCATCATGAACATCAGGTAGTGGGTG ATGTTTCTATCACCCAGTTAAAAAGGCCC AGTAAACTCCTCTATCCACTGGAAAACA AGAAGACAAATGCCCAGTATTTGTTTGCT GACCGGAGCTGTCTGTTCTTGGTAGACCT GCTCTCGAACCTTGGATTCAGAAGAGTA CTGTGTGTTGGGACCCCAAGGTTACATG AGCTGATCAGGTTGAAAGAATCAGGTGG CACGAAGTCTAACATTAGAAGCCTTTTAT TGGATATTGATTTTCG |

| | |
|---|---|
| AC_000167.1:10418278\|10437326 | GGAACAACCCATCTTCAGCACTCGAGCT<br>CATGTCTTCCAGATCGACCCAAACACAA<br>AGAAGAACTGGGTGCCCACCAGCAAGC<br>ATGCAGTTACTGTGTCTTATTTCTATGACA<br>GCACAAGAAACGTGTATAGGATAATCAG<br>TTTAGACGGCTCAAAGcgttattaatACAGCAC<br>CATCACTCCAAACATGACATTTACTAAAA<br>CATCTCAGAAGTTTGGCCAGTGGGCCGA<br>TAGCAGGGCAAACACTGTTTATGGACTG<br>GGGTTCTCCTCTGAGCATCATCTTTCGAA<br>ATTTGCAGAAAAGTTTCAGGAATTTAAA<br>GAAGCTGCTCGACTAGCAAAGGAGAAAT<br>CACAAGAAAAGATGGAACTTACCAGTAC<br>ACCTTCACAGcttagtcgAGGTGGGGATCTTC<br>AGTCTCCTTTAACACCAGAAAGCATCAA<br>CGGGACAGATGATGAGAGAACACCTGAT<br>CTGACGCAGAACTCGGAGCCGCGGCCTG<br>AACCCACCCAGAACGCACTGCCGTTCAC<br>ACATAG |
| AC_000158.1:67422933\|67458942 | attctgtgaaaaatagtaTGCATAGTTTGATAGGAA<br>TCACATTAaatctatagactgctttgggtagtatgaccatttta<br>acagtattaattcttccaatccaagactGCACTATGATAA<br>ATAGAAGTGGTGAGTCAAATGTCCTTGA<br>CTTGTTCTAGAATTTAGTGGGAAGACTTT<br>TAGCTGTTCACTGTTGAGTATTTGGTTGA<br>CTATGGATTTGTCATAAATGcttttattaagcattttt<br>attatttttaaaaatttcctctgtACCTACTTTGGTGAGA<br>GTTTTTAACATGAATTaatactgaattttatcaaatgctt<br>tttctatatcTGTTGAAATGATCACATGGTTTTT<br>GTCTTTCGTTAacgtggtgtatcacattgatttgcatatgtt<br>gaaccatGCTTGCGACCCTGGATGAATccgactt<br>gatcatggtgtgtgatcctttttatgtgttgttggatccagtttcctaat<br>attttgttgagtatttttacaCCTATAGttatcaaagatattggcct<br>gtagttttctttttatatgtctGATTCTGGtatgagggtgatggt<br>ggcttcatggGATGACTTTGGGAGCGTACCCC<br>CCTCTTTAATGTTTTGGAGTAATTTGAGA<br>GAGATCATTGTGAGTTctgtgtttggtagaattctcca<br>gtgaagccatctggtcctggacttttgtttgaaGAGAGTTTT<br>GTTTTATTAGTACTACATATTCTATTTCAAT<br>TCCAGTGATCAGTATGTTCAGATTAGTTAT<br>TTCTGCTTGATTCATTTTTGggctgtatgtttctagg<br>aacttgtctatttcttctaggttatccagtTTGTTGGCATAT<br>ATTGTTTAAAGTATACTCTTaatggttgggtttttttt<br>tttttttttttgtatttctgtggcacTGGttattatttatcctttatttctta |

| | |
|---|---|
| | tttggaTCCTTTCTCTTGTTTACCCTAGCCAG<br>aagtttttgattttttttatcctttcaaaaAATGAGCTCTtggt<br>tttaatgattttttcaatgtctttcttaaactctattttatttccttgctgA<br>TCTTTACTTTCTTCTGCtatggttttgtttgttcttcata<br>TTCTAGTTCTTTTAGGTGGTTGGTTAGgctg<br>tttgagatttttcttggttttgggtggttttttttttcagttttcataaaAG<br>TGTGTTTTATTGTTGGCAGATAGCACCTC<br>TAACAGTTAAATGCATTGAAgtaatgtatgggctt<br>cccaggtggtgctggtggtaaagaacccatatgccagtgcagga<br>gacgcaagagacatgggtttgatccctgggttgcaaggATCC<br>Tttgaaggagggcatgcaacctactctatattcttgcctggagaat<br>cccacggacagaggagcctggtgggctgtagtccatagggtcac<br>aaagagttggacatgactgaagagacttagcatagcatagcaatgt<br>ATAGGTGACTGCACAACTGCAGCATTGG<br>TAACTAGATAGATAACCCTTTCAACTAGA<br>AACCAACTAATAAGTAactatctaaatatttaaaatac<br>agctcTTATTTAGATCATCCTTTGTTTTGATC<br>ACCTTGGGTATATGGGGCTCCTGTTGGTC<br>ACACTGCAAATATATTAAAGTCAGATCTT<br>CTGATACCCGTTCCTGGAGGTTTCTCTCA<br>TCCTCCAGCTCCAGAACAAGCCCAGGTT<br>TGTGACCTCCAGCATTATACTCTTCCCATC<br>TACCAGAGCAGACAATGGAGGCTTCATA<br>CCAGGCCTCAAAGTCTGAGTGTAGCCTAT<br>TCCAATTAAACTATAGTTGTTGACTTTTG<br>CATAAACTGAAGCAATAGGATCCAACTG<br>ATATTTAGCTGCAGTGCCAAAGCTAGTGC<br>AGCTGGTTCCTGATGTCCAAGCAAGGTT<br>TATTAAACTGTGAAGATAAATTGATCCTC<br>TAAATTCTTTCCCATCATTGGCATAGTATG<br>TAGCTGGAAGTCCTCAGTCCCACTATCTT<br>CATCTCCACCCCTTTATCgtgatttatataaaaatata<br>aagtgaattGTGAAAATGCTCCATCTTCCACA<br>AAGATCTCGTAGGTCTTTGGGGGAGACT<br>TCCCCAAAGTCCTACAAGATCTTTGGGTC<br>TCACctcctttttttatatttatgggCACCACTCCACTC<br>CTGATTTGTGGGACACAGAGGTTTATATC<br>CTCTGGCAACTCAGAAACTTTAACTCTCT<br>CTCATGATCTCACTGCATTGCTAGGTTTG<br>AGATGCtcgatgctgcaaagaacaaggTCCGGGTGA<br>AGATCAGCTATGTAATGATTGCCTTGACA<br>GTGGCAGGATGCGTATTGATGGTTATAGA<br>GGGCAAGAAGGTAAGAGTGGACCTCCCC<br>TCTATCTTTATGTATTTTCCACAAAAGACC |

| | |
|---|---|
| | TAGAGGAAATCAGGGGTTAACTCTTTATT<br>GTCTGTGATGAGGAAGTCACTTCCTATAC<br>AGGAAAtgataagaaacaaaataaaatatggtaggACCA<br>CACCTGAGATTATCATGCCTGGAGGTAAA<br>TAGCAAATCCTAACTTTGGCATGCTGAAG<br>AACTAAAATAGTTAGAAGGGTTTAGTTGC<br>CACAGGTGTCATAAGGAATGTGAAACAA<br>GATGTGTGACACTGGCATATATACTGATAT<br>CTCCTTGTATGTCTGTAAAGCACTCACAG<br>TGCATGGACTTACTGATCCTCACTCTGCC<br>TTAGTCTTGGTAAGTGTTCTTAGCACTGT<br>TCTCTACTTGGATGTTCCAACTAGGCCAT<br>CTGTCTAAGCTCCTAAGCAGTACACAGG<br>CATTCAGGTCCACAAATCCAGACATTATT<br>ATGTCTTACCGTCTTCCAGGTACCTTGTT<br>GTTGTCTCAGTTAAATCTTACAACAATGC<br>TGcaaggtagatactattattaggacctcatttataaatgaggaaa<br>cagcatAGAAAGCTTAGTAACTTACCCAAGT<br>TCACACACATCTCCCTAATAGCAGAGCCA<br>CAGTTTGAATCTTGACCTAATTGAGGTGA<br>CAGGAAAAGTATTCCCACTTACTGAGTG<br>CTCAGTTTATAACAGGTATTGTTAAACTT<br>Gggcttagtggttaagaatccccctgccagtgaaggagatccgg<br>gttcgatccttgggctgggatgatcccctggagttggaaacggca<br>acccgctccaggattcttacctggagaatcccattgacagaggag<br>cctggcaggctatagtccatcgggttgcacagagtctgatacaact<br>gaggcATCTGAGCACAGCACATTGTTCAGC<br>TTACTAATCACCACAGTAATCGTGAGAAC<br>CTAAGAGGTCAGTTCTCTTATCCATGTTT<br>TGTAAAAGAACCAAAACTTAGAGATGTT<br>ACTTTTTCCaatgtcacatagctagtaagtggtaaGACA<br>AAAAGGTAGAGCCAAGTGTGCCGACAAC<br>TATCTTGTgattaaaatacagatatttttctATGCAGCA<br>GTACTGGCCAAGCCATTATTTAAATCCCA<br>TGTCTAATGCTATATATCACAGCTTCCACA<br>TGGAAAGAAATGTCTTATATCTATTGTCA<br>AGAATCTACAAAGGTTCTGCAAACCCAG<br>TGATAACTGACTTCCTTATGCGCAGTGAG<br>TCCAGAGTAAAAGTGAACTTCTGATTCG<br>CTTGAGAGTCTTGGAATAGAAAATACCA<br>ACCATTTAGGCTGATGTTATGACAGCATG<br>AAGGGCAAGTGTGGAGGCCTGGATGAA<br>GTTCTTTTGACAAATGTGTTCCTACcctacttc<br>agtatttttcctgtctCCCTCTTTGACTTTTCATGC |

| | |
|---|---|
| | TCTCAGACTGAACCTACAGAAGCATATAA<br>AACAGGATATAGGGATTCTAAGAATTCTG<br>AGAAATCTGCATTGCTTTTGTCTACTTGA<br>TGCTAGCTGAGTTTAAATATTCCAGAAAC<br>AGATCACTCCCTGGGATCCCCCCAGCCTC<br>ATGAAAGGGTATGCACTGTATAAAGAGCT<br>CCTCTCCTGTCCTAtaccaaaaatattatttctcttttatgg<br>aACTGTCCGTTTCTGAAAAGTTGGTAAAT<br>GCTACTGTAATGACTAACTTCTGAACTTA<br>CTGTGATTTAGAAGAGAGAAATTAGCCCT<br>TGTGTTTTGTCCTCTTCATCATCTAACCAT<br>TCTGATTCTGCGCCCCTCTCCATACTTAAT<br>TCTGAAGAAAAACACCACCTTATAGGGT<br>GCTGGTTTCACTCATTTGGTATGAAAATT<br>TGAAACTAGACTGTGTATGTAAGCTTCAG<br>AGATTAAACAAGTCAGAGGGAGGGAAG<br>CAAGGGAAGAAGGTTGCATCTCCTCCAC<br>TGCTCTTCACTCTCGGAGGATGGAGTGC<br>CAAGGTAGACACTGCTGGGAGTCATCAA<br>GGGTAGTACTTGGTGAAATCTGGGATCCC<br>CAAAGCTGAATGTTTGTGGTAAAAGGAA<br>TACGGAACTAGAAGGGTGTGAAAGTATA<br>CACATGAGTTGGGGATGGAGAAATGGTA<br>GCAACAAAGAAAagtgagaggaggaagaaaggggg<br>tagaaatgcaaacaaaaaggTGTTTAATgctaaagagagg<br>aaaagagaaagacaagggaATATATTATAAAGGGA<br>GAAGAGAGGCTAGAAGATTTAAAGAGAG<br>AGgccagaaaggaagagaaggcagaggtATGGCATTT<br>CTCTTGTGCGTGAATGGATGGACTGCCAT<br>ACTGGGAGCATTAGAGGAGGCAGAAGGC<br>ACATGGAGAGGGCTGCTGTGCCTGGCGC<br>ACAAGCAGTGAACCATTCCACCATGCCT<br>GTACTTGCCACGTCAGGTAATGACATCCT<br>CCCCGAACCGGCATAAATAATCACTTGTT<br>CACATTTGTTTCACTTTAGGCTGCCAGAA<br>GAAATGAGACTTTAACAAGCTTGAACCT<br>AGAAAAGAAAGCTCGTCTGAGAGAAGA<br>AGCTGCTATGAAGGCCAAAACAGAGTAG<br>CAGATATATCGTTGGTTGGATTTTGAAGA<br>CCTAGAAATAATGCTGCAGCAATAGGCCT<br>TACTAAAAAGAATGTGGTGTATGAGCTTC<br>CCTATTTCCACTCTAGAGCtacaaataaattttctta<br>aagaaatgactTGTCTATATTTtacaactttcagttcagttc<br>agttgctcagtcgtgtccgactctttgcgaccccatgaaccgcagc |

| | |
|---|---|
| | atgccaggcctccctgtccatcaccaactcctggagttcacccaaa<br>ctcatgtccattgagtcggtgatgccatctaaccatctcatcctctgtt<br>gtccccttctcctcctgctttcagtctttcccaacatcagggtcctttc<br>aaatgagtcagctcttcacatcaggtggccaaaatactggagtttca<br>gcttcagcatcagtccttccaatgaacacccaggactgatttccttta<br>ggatagactggttggatctccttgcagtccaagggactctcaagag<br>tcttctccaacactacagtttgatCTAGGTTAAATGCAG<br>CCTgctttttcaagaaaatgaaagaatgaaaaatgagatGAC<br>TTATTTGAACTTTATTATTAACTCTTCTTTA<br>TAGTCAGGTCCCATATCCTACTTTAATTCC<br>CATTACCAAAGTGACATCTCCCCAGCAG<br>CCAGATGGTTTGTGTGTCCTTATTTCTGG<br>AGAGGTTAGGAGGGACAGCTGAGTACAC<br>TGCCTGGGGGCACGCTGCCCGAGTGCAC<br>GCCGAAGAGCTAACCTCCCCGAATGCCC<br>TGGTGCTTCTCAAGAGCTCTGGGAAGCA<br>GCCAGACCACACTCTGCCTTTGTCAGGT<br>GTTCTGGTAAACAGAGGCTTTTAGGAGA<br>ACAGTCTTGGATAGGTCCTGGCAAGGTG<br>ATGATGGCATTCTGGCCACAAGTTTCTGA<br>GGATTTTGAAATCCCAGCACCATGGAGTT<br>ACTTGGGACCAGCACAGTTGCCTCTTCT<br>GTGCTGTAGCTTAGAATTCCTAACTGgtcctt<br>tccttcttttacacTAAATTTCTCAATTACGAAAA<br>GCtatcaaatgtattttattgttgttgaaaACTAAGCATTT<br>GTACCTCATATATATCTTATTTAGTCTGAC<br>GGGGATGCCAAGTTGTCTCATATTTAGCT<br>ATGGATGCTGGTTTCTATTTaagtatttcatttattct<br>cacaTGCTGCATGAGTTTTAAGGGATGGCA<br>AGTCCTTATAAACATTCAGAGGGTTATAT<br>CAGATTAAATGATATATGCAGGTATTTGGG<br>AACTTAgtttaataaaatagtaaaatctaATGTACTATA<br>CAGCTTCATAATCCATCTGATAACTATCTA<br>TATAGAGTACTTTGACCTTAGCTTTTAGAT<br>ACCAAGCTCTTTGATTTTTATCACTTGGC<br>AAATTTATCATCAAAAAACAttactttatatacatttt<br>agcAGATTAGTCTTCCTTGATTAATATTTTG<br>GAATACTTACATTTTATCTAAATGTAGATT<br>TTAAATATGGGTAAatctaatgtatttttaagtcatttttct<br>tcataaaacaaTACCATTTTAACTTACaaagaagcatt<br>taaaaaatttttaagtgttgtATTCTTGAAGATTTATT<br>GTTGTAAAATGTTTAAGTCAAAGCTCAA<br>AAATCCTACCATGCAGCAAGAATTTATAT<br>CATCATTTTGTGTCAGCTGTCCTCTTTAGT |

| | |
|---|---|
| | TAAGTTATAATAGTGACTGCCTCTTTGGG GGTTGAATATAAATTTTCTGACCAGAATT TAAGACCTTCCCCCATCCGATCCCACTTC TCCCAGTAGCCTCACATATTAACTTCTGA GCTGTCACATACTCTACTAGTAATTATAGT ATCTTATATTGCTTACTTCTCAAGCAAATC AGATTTCATGAGCACTGTTATAAGCAACA AGGACAAAACTGTCTTCTTTTGTATTCTA TAGTACCCACTTTCAATTAAGTGATGTTA CTATTTTTTGCTTAATTTCTGAatcatttttcatgtt tatcatGTCTCTACAGGTAAAGGGGgcagaaaat gttttccaatttATATTTCCTATAAACCCTGTTTG AATGAATTAACATGTGAGGCTATGGCTAG GTACTTGttaagttttaataaaaataatagtatggGGAATT GACTCCAAAATTTATTGAAATCAGGCAAC AAGTTGTGATGGATGTATAATTCATTGTC ATTAATCAAACATTACTTGAGTCCCTTTTA TATACAAAAACTGAGAATTCAAAGATGA ATAAACATACTTAGAGTCTACAGAGCTCA TCATCTACTGGACACATAGCCTGTGAAAC CATTACAGCACAGGCAGGCAGTGATGGT TCCACCAAGTGGTGATTTCTGAACTGAG TTTGTAGCATGGGCAGAAGCGGGTAGTT CAGACATAATAAGAAAGAGGCTTTACTAC TCAGACGAGCTTGCCCTGAGAATCAGGA GAGGGCAGTCTGACATGGTAGTGAGAGG TAAATGCCTCTCACAGTAAAATGCCTTTA TTAATTttgatttggtttatttttactttttatcttgGTAAAAA GACTCAGAACAAATACAACCATTACTTGT TTTTTCCCAAATTACAAACGATTCCACAG ACCATTTCACATCCCATCATTGAGGCTGT CACTTAAACTGTGGAAGTAGCATCTGAC AAATCTCCAGTCCACCACCATCCCAGGCT TATTCTCTTCTGATCTCCTAAACCAAATGC CTTGGAGTTATTCCAAACTCCCTTGTTCC CCTCATTTAATCAGTCATCCTGTTTTGTGA GCTTTACTTCTAAATTTTACCAAATACAAT GATCCTCTAGAATAGTCATTGAAACGAAT AGAATGAGAATCTGTTCAAGGACAGACA CAAGAGATTAATCACAGATATCAAGATTC CAGTCAGAAACCCTGGGATCTAAACTTG CATTTCCAACTGTCTAACTTTGCAAACTG Aggctcggttcagttcagtcgctcagtcatggctgactctttgcga ccccatgaatcgcagcacgccaggcctgcctgtccatcaccaact |

| | |
|---|---|
| | cccagagttcacccaaacccacgcccattgagtcggtgatgccatccagccatctcatcctctgtcgtccccttctcctcctgcccccaaaccctcccagcatcagggtcttttccaatgagtcaactcttcgcatgaggtggccaaagtattggactttgagctttagcatcagtccctccaaaacacccaggactgatctcctttagaatggactggttggatcttgaaACAGGTCTTAAATTCTTATAATGCAAgacaatgttaaaaaacaaaacaaaatacatgcCAAAATTGAGAGAACACCATCTTTTAAAATACCTAATCTTTTTTTGGCCAGTTTGACTCTACCACTTGATTTCTAAAGGGTTTAGTAAGTACTTGTCCACAGTTTAACTGTTTTGTCATTTCCTAATGCTGCTGACGCAATGATGTTTTCTGTAGGATGACAAGCTGCTGAGATTACCACATCTGTATGACCTTGCAATTTCTGTACAATCTCTTTAGTTTGAAGATTCCAAATGTATACCAGGTTATCCTCTGAACCAGAGACAATCCATTTTCCACCAGTAACTGAAAAACTGGCAAATACACAGTACTTCTCATTCTTATGACCAGTATATGTTTTCAGGCATCTGCCTCTGCTGTAATCCCACAGTTTAAGAGTATTGTCCAAAGTTGCAATGAGAATATATTTACCGTTTGgagaaaattttacaaaagagaCAGGAGGGTTATCATCATCAACAAGTGCTTTTAAACACTGACCTGATGCAGCATCCCAGATTCGACAGACACCATCATAGCTACCTGATACTATCAATGACCCACTACAATTAAAATGAACAGCAGAAACTGGGTCAGAATGAGCAGACAAAGTCTTAAGGCACTTGCCTGTTTTCACCTCCCATATTTTCACGCTTTCATCAAAAGATCCTGAAATGATGAGGTTGGACGGTGGATTGAAATTACAGCAAAAAACGTAATTACTGTGCCCTTTAAgtgttttcaaacattttccaGACCTCACATCCCAAATTTTTAGAGTTTTATCATCTGAGGCAGAAACAAGACGACTGGAATCTGATGACCAGGCAACATCTGATATTTCAAGATTGTGTCCTTTAAgtgttttctcatattttccatCATAGGCTCCCCAAATTATAATTACTTTATCAGCAGAAGAACTTGCTAGCCATTCTCCATTAGGACTAAACTTAACTGATGATACTGCTTCCGTATGTCCCTCCAGAGTGAATTTCAGAGCGTAGTTTGGTTTTTCAGACACTGGCTTGCTCTGATTGGCAGATGAGGGGAGAGCCGGCTCTGCTTTCGCATTTCCTGACTGCTCTGTAGTCATGACTCTGAAGCTCGAAGTCTGTAGGTTGGCTAGAT |

| | |
|---|---|
| | GTCCGGTTTTGAACCAAGCAGTCCGTATTTTGGCTTTGTACACAGCAAAATGAAGATAAACAACATCTgactcaaaacaaaaacaaacaaagtcttTTAGTGACTAATCTCTGGATCGTCTTTAAACGATGGTATACACTAGTTCAGCAGCACAGCTTGGACCACTTCACTTTTTCCTCGCTGCTTCTTCTGGAAACAAACACTTCAGAAATACTATTAAAACGGTCCCGTGCGCTGTCGGCAAGTAAGGTATTAATGGTGGGTGTTTTGTGACTACATAACGCGCTTCAAAGCTGCAAGCggtggggtgtggggggaaaatctacagaaaatatttttcaccgCTTCTCCCCCCAGCTCTGAAATATCTGAGTACTCAGCACCCCTACCTCCCTCACTTTAGACATTGTACTTTGAGCATGAATAATCGCCGCTAAAAACAAGATTTCAGAAGCTAAGCAAAAACCTGCATACACACGATTTCCTAACGCCCACACCTGCTGCCGACGCTCCAAACAGCTGCGTAAGGAATAAAGCGTGCGCATGACAGCTGGGAGCTGGGCGCATGCGCAGGGAGCGTAGAGCGCATGCGCAGAGGATGCCCCTCCCACCGCTTCCGAGCCGAGGAGGCTTGTAGAAGtctgtttcagatcagatcagtcgctcagtcgtgtccgactctttgcgaccccatgaatcgcagcactccaggcctccctgtccatcaccaactcccggagttcactgagactcacggccatcgagtcagtgatgccatccagtcatcacTACGCTTGCGCAGAAGCAGACGCTGGGGTTTCCTTCCGGCGTCTGCAGGAAGGGCTTCTTCCAGATCTTGCTGGATAGCGTGGGTCGCTGTATTTCGGAGACTATTCATGACTTTCGCGAAACCGTGGGACGAGAAAGAACTAAATTTTGAGATGCCTGCGGGCATGGATTTGGATGTAAGCCATTCACACTGCGCGTTTCTTAGCCTTCCTGGGCCCACTCAGCGCCGGTGACTCTGTATAAGCTCCTTGCTGCACAGGTGGCTTtcgttctcttctccagcagtTATGTGGAGCCTGGTGGCGGGTTCGAAGGCCTGCAAGTAGGCGAGGGCAACGTAGCCAAATTTATGCCCAGGTGTAAATGTGTTCCCCAAGTGTTCGGGAAGgctaaaaaatatgttttagagGCCTTTGAAGGCTAGCGCCTAGGTAGCCTGAAATGATAGTGCCGGGGGAAATGAGCAAACGTTTTAGCACTTCCTATGGCTTAACTTTCCAAAGACAAGGAAAGTTCAGATCTGCTAGGTGTTTGGAACAGAAAAGGCGGAATGGAGTAAAAaatgtg |

| | |
|---|---|
| | tgaagtgaaagtgaagtcgctcagtcgtgtccgactctttgcgacc<br>cggtggactgtagcctaccaggttttccgtccatggtattttacagg<br>caagaatcctggagtggattgccatatccttttccagaggatcttcc<br>cgacccagggattgaacccaggtctccctcattgtaggcagacac<br>tttactatctgagccaccagggaagtccaaaaaaaaaaaagtgtaaa<br>gttACTCGTAATTAGgctttgttggagaaggaaatggca<br>acccactccagtattcttgcctggagaatcccagggNNNNNN<br>NNNNNNNNNNNNNNNNNNNNNNNNNN<br>NNNNNNNNNNNNNNNNNNNNNNNNNN<br>NNNNNNNNNNNNNNNNNNNNNNNNNN<br>NNNNNNNNNNNNNNNNCCCAGCCCCAC<br>AAAACAATCACTGAAAAGCATCAAGAAA<br>ATAGAAGTGTATAATactaggtatatatatattttttttca<br>atataatacTGAAAACATCTAACAAGTTAACA<br>TTTAATATTAATGTGTATTAAACCATCTTT<br>GCTTGATTCTCTACCTCCTTCAATATATGA<br>ACCTATTTCTTTGCCAAACTTAAATCTAG<br>GATTGAAATCaacttcttcatttcttccctcaatttatttattgg<br>gcatactgaaaatgacagtttttcatctgaaaattggCACGAG<br>TGTGaattttacaaattatttaaccATAAGATTATGtcaa<br>cttatttttattatagtttatttataatattatattaatttcaaacatataat<br>gtattgattcagtatttttatagattttactaaatttaaagttattataaaa<br>tattggctatattccctttGCTGTATCATCCATTTGTT<br>AAAATAAGCTTTTATTATGATTAAAGTATT<br>AATTACAtaatcattttagaaaattagaaaaaaatttaaagaaa<br>atagaattatcCATTGTTAACCATTATTTAGTTTT<br>TTCTATAcatatctgcaaatatttttattaaaatgtggtactatata<br>acatttaaaagtcCTACTTTATGAAATTATACCAT<br>GAGCACTTGCCCTTGCTATCTTGAAGATA<br>TATttaatacacacataaatattccATTCAGTGGGGAT<br>ATCACAACTTATTTGACTAGCCTATTATTC<br>TTTAATTAGTAGTTTAATGTCCTTTTCCAG<br>AATCCAATTTTGAAGTAAAACAGCTTGTA<br>AAAGGCTCATAATTTAAACTAATAAAAAG<br>AGGCAAATGTCAGGTCTAAAACCAACTT<br>CTTGTATCACCTCTTCTTGTATCTTTCTAT<br>TTTGGTCAACGGTATCAAGATTCTCTGTA<br>ATCTGGAATTCTTAGGGGATAATCTCAAC<br>ATACTAAGCAAGCATCACATTAAATAATG<br>TCTTATACACAGTAGAGGATATATAACGTC<br>AATAAGTGGCAAATTCCAAACAAACAGT<br>GGCAGCACTATTTAGAGAGGCAAAAACT<br>GGTTAAGTAGTAGACAGGGACCTTCCTG<br>GCAAACTTTACCTCCCACAACTTgcctattaaa |

| | |
|---|---|
| | acaaaaaaacctttaaaCTCTCATCTTTCCTCCTGT<br>CTAAATCAAACTGATCCTGAGGACAAAG<br>TTTATACCTTCCAGATAAAGCTTTCTTTGG<br>ATGAGAGTCCTCAGCGATCTTTCTTCTTC<br>AAACATACGAAGCaattagagagagagagcacagag<br>TTCATTCATTAACTCACTCttccttcaaaaaatattta<br>ctaatgtCTAGCACATGTTAGACACCATTCTA<br>AACACCAGGGCTATAATGATGAGTAAAA<br>CAGAAAAGGTCCTTGCTCTTATGGAGCA<br>AAATTCTCATGGGggaaacagacaataaacaagtaT<br>CCCAATAAACAAGACACTTTCAGTGCAG<br>AAAAGTactgcaaaaaagaaaactgtagtaATGAGAT<br>GAACAGTGGGGTTGAGGGGCTAATACAT<br>TATACAGGGAGATCAAGACAGACCTCTC<br>CAGGACATACCATTTGAACTGAGATCTGA<br>ATGACACAAATAAGGCAACTAAAAGGAC<br>ATTTTGGGGAACAAGGCAAAAATTAAGG<br>GGCAAGAACAAACTTGGGGATGTTTGAA<br>GAACAAAAAGAAGGCAAGTGGTCTGGG<br>AGCGAGTAAGTGAAAAAGTGGTATTAGG<br>TGAGAGCAGAGTTgatgagaaagtgaaggacaggag<br>agacCACAGAAACAATTAAGGATTCTGTTC<br>TAAAGGAAACAGGGTTTTAGGCAAGGGA<br>ATGAtataacagtattttaaaagacaacttCAGCTGCTA<br>AGTTGAGAATGGAAACATTGAGCAGACT<br>ACTATGGTGGTCCAGGCAAGAGATAAAG<br>ATAGTTTGATTAAGAGGTTGGTGAACCTG<br>GGCCTTCAGCAGTGAGAACCTGGAGTCC<br>TAAacattggacagccagggaattctccaaattaatttctttaac<br>GCCATGAAACAGGAAGAGATCACTGAAG<br>GAGACTATAGCTAATAAAGGGGGTTAAC<br>AATCAAGAAGGAACTCCAGCATCTAAAA<br>CTAcagaagaggacttccctggtggtacagtagataggaacct<br>gcctgctaaggcaggagacagggattcgatccctggtccaggaa<br>gaccccacagagCAAACTAAGCTCCTGcaccaacta<br>ctgagcccgcactctggagcctgccaggcacaactactgagcct<br>gcgtgctgcaactactgaagccctcacacctagagcctgtgctctg<br>caacaagagaagttacggcaatgagaagccatgcactgaaacta<br>gagagtagcccccacttgccacaactgcAGAAGAAAgct<br>cacatgcagcaacaaagacccagcccaaccaacaaaataataaa<br>gaaataaaatttaaaaaataaaactacaggaaAAAGACTG<br>ATGTCACAgaactgaaaagaagagaagtttcaAGGCA<br>GGAACAGTCAACTGTGTTgaatgctagtcactcag<br>ttgtgtctgactctttgcaaccccatggactgtagtccgccaggctc |

| | |
|---|---|
| | ctttgttcacaggattctccaggcaagaatactggggttgggtagc<br>cattcccttctccaggggctcttccagacccaaggatggaacccg<br>ggtttcctgcacagcaggttggttctttaccatctaagccaccaggg<br>aagcctgttgaaTGTTACTAGAAGTAAATAAAG<br>ATGTGGGCAGAAATTAACTTCTTAGATTT<br>AACATGATGGAAATTACAGGTCATCTTTA<br>CAGGAGTCATTAAAATAGAGTGTTTAATG<br>GACTGAACAGTTAACATGGCTTAAGTATA<br>GAGGGTAAGACAAAGTATGGTGTAAAAT<br>GAGGCCCACCATTATAGGTCACCTTATTG<br>GATGTTAGTCTTTTTCCTGAGCGCAAATA<br>GAGAACCAATGAAGTGTTTAAATACAGT<br>GGAGTTGTATGATAGGGTTTGTATTCTGA<br>AATGACTGCTTAGGTTCAAGTATTAACAT<br>GTGGAAAATGGATTAGACAGGAATAAGA<br>GTAGATACAAAGAGGTTAGTTAGGAGATT<br>GGTATAGTAATGCAGGTGAAAGATTATAT<br>ATCTTCACATTTCCCAGAATTATGACTTAC<br>TTTGAACTTCAGGTTTGAGTACAATCATT<br>AGCAATACAGCCAACTTTCCCTCTGATAA<br>AACAATCTGGATATACTATTAAGTTCCTTT<br>TAACAATGTGAATTTAGCAGCATTAACTA<br>GGTAATGAATCAAACAATTTAAGTCTTAC<br>AAATGGAAACTAAAAtgtgaattttcaaatatatcagC<br>CCTCTCTCTGTGTGCTAAAGATTCATCAA<br>TATAGGACCGAGTGCTTGGCTTTTAATAC<br>GGTAGACATTAGCCATATGAGGTTATTttagt<br>taaaattaaatttaaaattagtttttttagTTGCATGAGCTAC<br>ATTTCAAGAAGTACTCAAGAGCCACATAtt<br>gctagtggctaccatattgtaCAGGACACATTTCCAC<br>TACCACAGAAAATTCTACTGGACAGTACT<br>GAATTCAAGATGATATAATAACAATCTCTA<br>AACTGATAATGTACTCGTTCGGGAATTGC<br>TGAGAATCGTAAGCTATTTTTAAGATAGG<br>AAAAAAGATACTAGAAAAGCTCAAGGAG<br>GGGAAAGGGAGTAAGACTTAGGAaggaattt<br>ctttcaaaattgcaaaaagaaaaagaaaagaactataaACTAC<br>GTCACTAGGCACAAATTTCTTTATTACAA<br>AAAGTGCCAGGGCTGTTCAGGCAGCTCC<br>AAGGTCTAGAGAAAAGCTTGAGCAGACC<br>TCACAGGCTGAGAGCAGATAAGGTTAAT<br>GCAGGCACATAAATCCTGCCATTAAACCA<br>TGTGTATCATTCAAAATGAATTACTGACT<br>GCCTACCTGATACTAGCTCCAATGACAGT |

| | |
|---|---|
| | CCTTCTTTCTCACAAATTCgccaagaaaatataaa<br>gaagacacCCTTGAGCAGACATCCTACAATG<br>AACTGCAACACAAGTCCACAGAGATAAT<br>ACAGAAATATACACAGTTCAGTGGCTCTC<br>TCTGTATAGGAGCTGGAACCCTAAATTAG<br>CTTCTAAGTGTCAAAGCACAGCAAAAAG<br>ATAACTCTTCAAACTTTGTAGTATCTCTAG<br>CTTCTCCTTTCACCCTCCTCAAAACAAGA<br>AGAAATTGATACATCACGAATCTCTAATT<br>TTAGACTGACCCTTTAAAATTATTCTGGC<br>ACTTATCTTGGAATTGTATTTCATACATTA<br>TCTTTCATTCATTTCGCCCTCTTGGAAGG<br>CAATTTCAGTACTGAAGGACTAGCATGTC<br>TAGCAGCAAGGAGGGGTAGAGAAAACTT<br>CCTGTTCTCCCTACAATTACAATTATACTA<br>CACTCTATTCCTAAGGTTAATTCTTAGGTA<br>aatccattttattgttttaagaagCTTTCAAAGTATATAA<br>GGAGAGGTAAAAGGTCTATTACATgacagta<br>aaattaaaatgtgctCCATAGAATGATTTGGAATT<br>TAACCTTTCTTCTCCTCACTTTGCCTGAA<br>GGTACCCAGAAGTAGATCCCATTCACATG<br>GTGCTCTTTTTAGAAGACGTGCGTGGTTC<br>CTCAAACTCCATTTTTACAACAATGTTCA<br>CAGTGTGCTTCCTGCAAGCCAGAAATAC<br>AGACTTGCCTGCAACTGCCAGAATCGTC<br>TGCAGTCCTCTGCACTCCCAGGCACGGG<br>AACAGAGAGCAGCTAGGCATGttgcaaaagaa<br>accatcatGGATGCTAGAAAATAGCCTATTCA<br>GTCTATATTTAATTACCAACCAACTCAAA<br>ATTAATCCTGTAAGTTAATATTCCTATCAG<br>AAATATGAAAACTTACATAGCCTAGGAAT<br>GCTCAGTTGATTCTGCTCAGGTAATACAT<br>GCACTGGGTCTCAGGAAGAAGGTATTTC<br>AGGGTGTCAAGTGGGAAAAAAGCACAA<br>ATGACAAAACATCATGTGGTCAGGGAAA<br>AGCAGGCCTATATGGCTAGACAGCAAAG<br>TTCAAATCTATATTGGAAGTGGCAGGGGT<br>TGGGCAGAGAGAATATGTATGAATAAGG<br>GCAGTGGTAAGAGATTTAACTAAGCAGA<br>ATCACTTTGGGATTGGATTTTAAAGACTT<br>GTATGCTATGTTAAGTAGTTTACACCTAGT<br>CATACAATTCTGAAATTAGATGCATTAAC<br>CTGGTTCCAATCTTTTGTGTAATCCAAAT<br>ATGCCCCTGTGCAAATTCAGAATTCCAGC |

| | |
|---|---|
| | AATTTTCTATTTCCCCCGCAACACATAAAACTTTCTGCCTTTCTGCCTATATACGGTTTCTTCTACCGAGAATTACCCTGGTTCTATCTACCAAAGACCCAGTTCAATTAACATCTCTTTGACTTAGTTCTCTCTGAACAGTTATAACACTGTGCTCATAGTCTAAACATCTTCTACATGTTTTATACCCTGACTTGAgctctttttatgttttactcTCAATTTCTCCAAGTGCCTGGTACCTAGCAGATATCTGTTACGGATTGAACTATGCCCTCCCCCCAACCTGCCCCcattttcatatgttgaagccctaaaaCCCAATGTGGCTGCATGGAAACAGGGCCTTTAGGGaagcaataaaaagttaaataaggtcAAAAAGCGTGGGACACTAATCCCATAGAACTGATGTCACTGTAAGAGGAAAAGACACCAGAGACCTCTCTCCCTTTGTGGCCACAAATGGGTCATATGAGGATAGTGATGAACTAGCAACTTActttgaaatgcatcaaaaaaaattaaaaatgtggaTTGATGGATAGATTGAGGGACAGATATGTGATTTAGTAGATACAGTGAATCACTATAAAATCCAGATACTGGGTACACGAGTATTCATTGTAAACGTCTTTCAATTTTTCTACAGTGTTTGAACATCTTCATAATAAaattctgggggggggggggggaagtacctaaatctttataataaaaatacatgattttctttttctctttcacagtATTCAGTGCAGGGCTTTTCATATATAAGGTATTTACATACACAATGT |
| AC_000164.1:18971362\|18980712 | CGGATCTGTCAGTTAAGAGTGGGTGGAGGAATTCCTCTTCAGGGAAGGATATCAGATCCATCACAATTGGTTAGATGAGCTTTCCATCAACCTACATCCGGGACAATCTTTCTAGTGATTGGTGGGTGTGAAGGCGGGGTTAAGCAATATTAGCATTCCCATTCGGGACTGGTGCTGTCCATTTGGTGTTTTACAGGGGCGGGCGCGAGAGGAGGCAATCGGTGATTGGCTGGGAGGGCTCAAAAAGCAGAAGGGTCGGCCCCCACCGTGCGGCGATTGGTTCACCGGTAGGCGGGCCTGGGGGCGTCAGAGGGGGTGTCAAGGGAGGCGGTGGCTCCAGAGATGGCAGTGAGCGAGAGGAGGGGGCTCGGCCGTGGGAGCCCCGCGGAATGGGGACCGTccgatgaagacgacgacgacaatccgGGTTCCTCTGGACGCATTCACCGGCTGACGCTGACGGTGAGTCCCGCCGCGTGGCGGTCTGGGGTGCGGGGCGCAGCTTTGGCCCGGAA |

| | |
|---|---|
| | AAGCCCCTAGCTTGGAGTGGTCTCGTCCT<br>ATCTCCCCCGCTCCTTATGCGGGATGGAG<br>AGGTCCAGCGCCCCCTGCTTCAGGCCAG<br>GAGTGGTCTCGCCAGAGGAGCCCTCTCA<br>GTGGTTCTTTTGAAGATGCCTGGCCTGCT<br>GCGTTGGGGTCGTCCCTTGCCCACAGCG<br>GGTGGGGCTTCACCTCTGGGGACCCAGG<br>ACTCTGAGGCCTTTAAATTCTTGGGATCG<br>CTTTGGGGCTGCTAGGGACCCTTGACGCg<br>cgggtcccttcagggacccccaTCTAGAATGAGAGT<br>ACTAATTGGGATCTTGGGGACTCAGTTGG<br>AACCTAAAAATTTTCCTAGAAGTGAGGA<br>CCAGGGTCCCCAAGATGCGTAATCCTGCT<br>TTACGGGTGATGCCCACCTCGTAGGGAG<br>AATTCCACGTATCCCAGACCTTCTCTTCC<br>TGGACTCTCCGAATGGTGGCCAGAGGAT<br>CCCGAACCATTCTGTGCTTACCTGGAATT<br>GGTCTGGGGTCATGGCCTACAGGGGTccgg<br>gagagaaggagagggcaCACCCTTTGAACCTGTC<br>CTAAATACAGAGATACCGGGAGGGGGCC<br>GAAGCCCTTGGCGGAATCTCGTGCCActcg<br>ggaccccgacccgaccaccGAAGTCTGAACTGTC<br>TCTGTTTCCTTGGTCTCCGGCAGGGGGA<br>GAAGCGAGCAGATATCCAACTGAACAGC<br>TTTGGTTTCTACACCAACGGCTCCCTGGA<br>GGTGAATCTGAGCCTCCTGAGGCTAGGC<br>CGCCAGGATACAGAAGAGAAGGCCCCGC<br>TGGTGAGGGGCTTTGGGAAATaacgacctcctc<br>cctctccacgACAAGTTTCTGAGCggtcggtggggg<br>ggtggggggggtgggggggggtcaTCTGGATTCACA<br>TCGTAGCTCTGCCTCACCCTTGTGATCGT<br>GGGCCAGTCCTTTGGTCTCTCCTGtcaaaaat<br>aaacgaatagaGTAACTGAGGAATATTTGCTTA<br>TCTGattgactcctcttccgttaccgttgggtgaggtcatgagaa<br>cggaccttttggggtacctgcctcctcggaccatccgaagtcaggt<br>accccagcgactcagcctgtgctgactcgctgaagtgaaagaga<br>aaagtgaaagtacgcaatctcttcctttaccgttgggtgaggtcaca<br>agaacggacctcttagggtccctgccccccctccgccccgaccatc<br>cgacggtatataccccagtgtgtctcagcctgtgttgactgtactga<br>atcgtcgttgactcGAATACTTTCTCAGGCTATAT<br>AGGAAGCTGGTGAGAGGGGAGCCTACC<br>AGTGAACGGGACAGCCCCAGTTCCTGCA<br>GTCAAAATAAGAGGCTAGTGTTATTGAAC<br>CCCCGCTGGGTACCCATGTACTGCAAATC |

| | |
|---|---|
| | AATTTCCATGAGTCTGTTCTGTCATAGCC<br>ACCTTTCAGGATCCCTGTGATAATATTAC<br>ACCTGTAAACCAAATTTTACAGGTGTTTG<br>TACAGACTCAGAGGTATGTGGCTTACTCA<br>GAGTCCCAGAGCTTGGATGTAGATCCAA<br>GTGTGTTGGATTCCAGAGCCCAAGGCAG<br>GAAGCCGTAAACATTCTACTTATTTTTTAC<br>ATCATCATTaatttaacccccacccccccctctcctACCAc<br>gcttccagaggacctcctttaGACATCCTAGTCGAGT<br>GGTTAGGAGTTAAAGGAAGAGGATTGGG<br>GTGCTGGGGGCAAGTAGGCAAAGTCAGT<br>GAATGCAGGATCTCCCCCTTAGTAGGTAT<br>TAAGGAAGTTCGAGGTTATGTGTCTGTTG<br>CCACTGATAGAGGTCCTTGCTGGCATCCC<br>CCACCTCACAGTCTCTTTTCTTTCCACTT<br>GCCAGGTGGGGTTCAGTCTGACCCGGGT<br>GAGATCTGGCAGCATTCGCTCCTACTCAG<br>TGAGTGGTAGGGATGAAAGTGGGGAGAG<br>GACGGTGctccccgtctccgtctccttcccctcccaTGTC<br>TGTTGGCCTCTGAAACTCAGAGGCCCCT<br>GTCCTCagggtctctccttccctctttgaCACCCTTGC<br>CggccccttcctcctctcctacccctccctccgtcccacccggTG<br>GACCAGTGGGTGGGGGTAACCAAAGCTC<br>CCTACTGCCCACCCTCCAGAATCGGGAC<br>TCCCATGAGTGTCCTCTCCGGAAAAACA<br>GTAGCAGCCTCCTGGTTCTCTTCCTCATC<br>AACACCAAGGATCTGGAGTGAGTATGGG<br>GGCTACTCTAAAGATCCATCACTGGGGTG<br>TCCCTCGTAATACCCATGTGAGCTGATCA<br>GGTGGAATGAGAACCAGCCAGAAACAA<br>CCTTATGCCTTTGGAGAAAGCCACagcgaaa<br>acaacaacaacaatttaaaatcaataaataaaaaccgacgtgaccc<br>agaaacaacgacgtgtatccgaaagagatgTGGTGGCGA<br>GCAGAAGCTACTTTCTCTCTACTTAAGAT<br>GCTCTGGCTTCTGATTCCTATGGCATCTCT<br>ataacgcctcgtgcccgagatcccgtaagGGGCTTCAGT<br>AGGTTCGGCAGGTGAGCTCTAGttgcgtccga<br>gtcaccaacaccgcgtacccgaatctaGTTCCGTGGCtat<br>cccctaggagggtctggtccctaacttaggtagaggggacgtaaa<br>ccgtccacctaagaaatggtgactcggtggtcccgtcgggaggg<br>accaccatgtcgcctatttttaggtggatggttacttcctctccgtcta<br>agctagagacctagtccttctaggggacctcatcctttaccgttgga<br>cgaggtcataagaacggacctcttagggtgcctgtctccttggacc<br>tcccgatgtaaaggtatcccagggtttctcaacctgtcttgacttcgc |

| | |
|---|---|
| | tgaatcgtacgtgtgtactggtcccttcggactgtcgATTTTCT TACTAAATAAGGTGTATTTCAGTAGAATG AGTGACTTGGTATCATGGGAAGTGACAG ATAATCCCTGGTGCTAGCAACACAGGGAT GTGGCAGCCATCTCGATTGGATGGTGGCC AAATGCTGCCACTTGATGACCACTGTTAG AGCCCCCCGAGAGAGGAGACCCTGAGG ACCTGAGTGGCCATTGAGTCCCTCAGCA CAGCTGGTGTTCCTGACCTCGGGCTTCA GCCTCCCCACATCATCCCCAGGGTCCAG GTACGAAAGTATGGGGAGCAGAAAAAGC TATTCATCTCTGCTGGGCTCCTCCCGGAA TCACCCTCCAAACCAGGGCTCCCGAAGT CAGAGCACATGGTCACCCCCAAGGTGGA CCACGGTGAGTCGGGCTGGGAGGAGTG GGTGGGCAGGGGTGCTCCATACATCCCC CCATCGTTCTGCTGTCTGTCCATCTCTGAT CTCACCTGTGTTTCACCAGCAGGGACCA CTGCTGCACCTGACAAGGCCAAGTCAAA ACCCACAGGGTTACAAGGGGACCGGCA GGTACGGGAtcggtcccgtcctccctccttccCCCCG CCCCCATAGGGGCAACTGATGCTCCATCG AGTTACTCTGTCCCCCGCAGGGTGTCAGT GGGAAGGACCAGGAGCTGGTGTTGGGC CTGGGCCACCTCAACAACTCCTATAATTT CAGTGTGAGTATCTGGGAGTGggacgtcgggg aggggaggtgttAGTGGAGACGCCCCCAAGTT GAGGaggggagaggggagacaggGCCCGCCTCTC AGTTCCATGTAGTGATTGGCTCTAGGGCC GAGGAAGGCCAGTACAACCTCAACTTCC ACAACTGTGACAACTCGGTGCCAGGCCG GGAGCAGCCATTTGACATCACGGTGAGac ctcccctcccgtcacccACCATGGAGCCCTCACTG CGGGCCAGGTACCGTGTGTGCCTCAGGG AGACGGGAAATCTGAGGATCAGAAATGA GAGAACGGAACCTGGACACTCACACTCA GGGCCCAGCGTGTCTGGAGCTAGCCTGC AACCCCCTGTCCCCCACTGCTTCCTCCCC AGGTAATGATCCGGGAGAAGAACCCCGA GGGCTACCTGTCAGCGGCGGAAATCCCT CTTTTCAAGCTGTACATGGTCATGTCCGC ATGCTTCCTGGGCGCCGGCATCTTCTGGG TGTCCATCCTCTGCAAGAACACGTAATGC CCTTGATCCTGGGGGTCCTCCACCTCCTG |

| | |
|---|---|
| | TCTCCCCACTTTCCCATCCCTGCCCCAAA CCAATGCCCTCTCTCTTCTGTCCCTCATCT CTCCTCCCCCTCCAGGTACAACGTCTTCA AGATCCACTGGCTCATGGCAGCCCTGAC TTTCACCAAGAGCcagagagaggagaaggtgtGT GTGAGAGTCTGGGGCCAGGAACAGCGT GGGGTGGGCTGGAGTCTCTGGGGCCAGG AAAAAGCCCCTACAAGCCACTCCCCAAA TCTCTGCAGACCCTGGGCTGGAGAGTTC CATATGTCCATCCGAGTGTCCATCCATTTT ATCACTGTAGCCAGCTTTCCATCCAGCCC TTCTGTCCAGCTGTGATAGCCCACCTTTC AGTCTGACTATTTGTGGGTCCAGCTGTGA CTATCAACCAGGCCGATTATCTAACTGTC CATCCGTCCGACTGTGACCTTCCCTCCAT TGTCGAGGGTGTGtgacagagaaagagacaggtCG CGCCATCCTGATTGGAACCGTCTGTTCCT GTCCCCTTCCTGGCCATCCATCCATCAGT GATTGTGCATGTTCCCAAACCACCATCCA TCCACCCTCCCCATTCCCCTGTCCATCTG TCTGACTGTCAATCCATCCACCCACCTCC AGATCAACTACTACTTCATCAACAGCCAG GGCCACCCCATCGAAGGCCTCGCTGTCA TGCACTACATCACGCATCTGTGAGTGCCC CTTTCTGCTGGGCAAGGTGGTGGGGAGG CAGACAGAGGAGGGCTGGGCCCCCAGG GTCTCACCACACCTCCTACCTCCCTCCGC CAGGCTGAAAGGTGCCCTCCTCTTCATC ACCATTGCCTTGATCGGCTCCGGCTGGGC CTTCGTCAAGTATGTGCTGTCAGACAAG GAGAAGAAGATCTTTGGGATAGTGATCC CACTGCAG |
| AC_000180.1:2936016\|2951681 | GTTTGAGAGAGAAGATCTGATCACCTGG AATTAAAGTTGGCACAGAAACCTTTTCA ACTCCAAAAATGTTGGAGGAAGATATGG AAGTGGCCATCAAGATGGTGGTTGTAGG GAACGGTGCAGTTGGAAAGTCAAGTATG ATTCAGCGGTATTGCAAAGGCATTTTTAC AAAAGACTACAAGAAAACCATTGGAGTT GATTTTTTGGAGCGACAAATCCAAGTTAA TGACGAAGACGTCAGGCTCATGTTATGG GATACAGCGGGTCAGGAGGAGTTTGACG CGATAACCAAGGCCTACTATCGAGGAGC CCAGGCTTGTGTGCTTGTATTTCTACCA |

| | |
|---|---|
| | CAGACAGGGAATCTTTTGAAGCCATTTCC AGTTGGAGAGAGAAAGTGGTGGCTGAA GTTGGAGACATACCAACTGCACTCGTAC AAAACAAGATCGATCTCCTGGATGACTCT TGTATAAAGAATGAGGAAGCCGAGGCAC TGGCAAAAAAGTTGAAGTTGAGATTCTA CAGGACTTCAGTGAAGGAGGACCTGAAT GTCAGTGAAGtttttaaatatttggctgaaaaatatcttcaaa agcTTAAACAACAAATAGCTGAGAATCCA GAATCAATGCATTCAAGTAGTAACAAAAT TG |
| AC_000181.1:934078\|947669 | GAAATCTAAGCATAAGTACGTAAGCGTAT GTTTAAGACAAAACAAGGAAGTCTGAcca tcttttctcttccttcacaTCCCGCTGTTTCTGCTGTA ACAGAATAGCTCTTGGCCTTTCTGCATAA ACTCTTTCGGGAGCACAAGGTGGGATTC CTGTCGCTCATCAGGACGGCCGCAGCCG CCCCGCGGCTTACAGAGTCTCAGTCCTC CAGCACCCCGCCCTGAGCGAGTCCGTAT AGACTTAGCCTCTTGCCTCTCCCCCCCGC CCCGATCACTGGTCCACATGTAACCAGAT GCGGTCACGGTCAGACTGGCTTGAGGAC ATTCGAGCAAATGGAACGGGCTTCAGGC ACCAGGGCATCGGCTCCGGGCTGCTGGG CGCGTGGGTGCAGCGGGCACCCCCAGCT TCCTGTTCTGTTTCCTGAGAAGACGGAG CGTCACCTTGTCGCCACACAGCCTTCCTC AGTCATTTCTCTTTGCCTCCCTGGCTGTA GCAAGCAGAAAAGCTGGGGAGGGAAGA CCAGCAGCGACTGCACCGCAACAGGAA GCTGGTGCTCATGGTGGACTTGGACCAG ACGCTGATCCACACCACGGAGCAGCACT GCCAGCAGATGTCCAACAAGGTGAGCGC GGGCGGCACTGCCGTCCTGGGAAGGAGC CTGGTGTTCCGCTTAGGAAGTGAACAGG CTCTGCCCCTGAGCTCACGGCCACGCTG GGGGACAGTGGAGAGCTGGTGGGGGCT GGAGTGGACCCTCTCTCCCACAGAGCAG TGCAGGTGTTCGGATGGCTAGTGTTTCTG TGTCGTGGAATCTTCTGTCCTGTGACAGC TCTCCATGCCGTCTGTGCCATCGCCTGTG GACATGTCTCAGGGTTTCCTTATCACGAC CACCGCATTCATGGGCGGCAGAGgacccacc gtccccaccccacggGGGGTGGCAACTGCAGCC |

| | |
|---|---|
| | ACTCGGGGCTTGTCTGCTGCGGAGCCGG ACATTTCGTTTCATAAGAGTTTGAACGGT TGCATCACACCCACTTGCATGTGTGGTAG CAAGGTGTCCTTGGGGCCGCGTGGACCA TGCCCACTGCCGGGGGCTTTCCTGTGCC CTCCCGATTCCCCACCACCACAGCTCCCA GACCTGCGGGCAGCACTTAGAGAGGTGG GTTGAACCCTTAACCTCAGATACCCTGAC TTGGGATTTGAGGATTTTTCTGTTGCAGT GTCCAGACGATTTACCGCTGAGCGTGcccg accaccacccccccccgccaccaccaaGTACCATCTTG ACTCCCAAGTGTTTTACCAGAGTAGCCCT GTCGTGAACAGTGTGACAGATTTATAGCC AGTGAAAACTTAACAATGCCTCcccagtaaag gaaaagaaaaactcaaTATTATTACTGGGAAAATG AATGATAAACAATTAAAAGGATCAGAGTT TAACTTTCTAAGGAGAGTAGACTTACGTG CATGTTGATCATGTGTGAGCAGGTTGGAG TTACTGATCTCATCTGTCTCCTAAAGATG GACCTGTTTTTGTGCTGTAAGTCAGTGGA GTTGCATTTTATAACCGTAAGGAAGTTAG GCTGAGTTGTGTGGGGCTCTTAGCTTACC TTCCTCTCTGGCCCCGGGAGGCACTGTG GATAGACCCTGGAATCTGCGGTGGTTGG CTACCTGCCCTGCGTGAAGTGACCCACC CAGGGTGCTCGTGAAATGGTTGGGACGT GAGTGTCCACCGGAATCCTGAGACACTT AGTGAGGCCTCCGGTGCCCGGTCCTCAG ACAACTAAGGCGCCATAGTTGTCACAGG AAGCTATCACAGGTCACCTGGCTATTGTG GGCAGTCGCTGTGATTATCCGGAGTACTC ACCCAGGCGGTTAATGCAGGAAAAGGCT GAGCCCCCTTACAGAGTGTGAGAGAGGA AGGCGTTTGTGTGGCGCGGCAGGCTGTG CTGCCGTGACTTCCCCCCCCATAGCCCCC AGCCTGGCGGCTGTCACCTGCCACATGT CAGCGCTGCCCTCTGACTGGGCCTGCaga gacgtcccgtagaaggtgTTCCAGCTAGGCCGGGG AGAGCCCATGCTGCACACACGCCTGCGT CCCCACTGCAAGGAGTTCCTGGAGAAGG TGGCCCGGCTGTACGAGCTGCACGTGTT CACATTCGGCAGCCGGCTGTACGCGCAC ACCATCGCAGGTGAGCAGCCCATGGGGT TTTGTCCCACTCTGGATACTCAACGGCCG |

| | |
|---|---|
| | GCGTGTGGGTTTGGCTCCTCTCCTTCTCA<br>AAGGTGGGTCCTGCAGATGGAGGGAAG<br>GTCAGAGGCTCTGCCTGACTCGGCCTCG<br>GCCCCGTGGTAGTGGCTGGGGCGCGGGC<br>AGGCCAGTCCTCCAGAATCTCTGCTCTGC<br>TCGGCCAGCAGTCACGCTCCAGTCCCAA<br>GTGCAAGCCCCTGTCCCTCTGTCAGCTG<br>CACGCCGCCGGCCCCTGGAGAGGCTGGT<br>CAGGATCACAGACGCTCGGGGAAGTAGG<br>AGCTCTCTATAAGAGCTTTCTTATACAGG<br>TAGAGACCCTCGGGGCTGCCAGCCTGGT<br>CACCGGGGACATGTCTGTCTCAGTAGAC<br>AGCAGTCGGCGTGGGTGTCAAGTCTGTG<br>ACCTTGGGGTCCTGTCACAGAGGGCGGG<br>GGCTCTGGTGGGCACTCAGTGAGCCTGG<br>GAACAGCTGAGCACTGGTGCCGTGCGTG<br>GCTGGGCAGGTGCTTCCTCGTCCATGTCC<br>ATGCAGTCCTGCTGTCAGGGGCGAGTTT<br>CAAAGCACTGCCTGGATGCCCTGCTCTC<br>CCCTCTGCTGTACGAACACGACGGCCCA<br>GGACATGCACAGGCCACGGGCCCCTCTC<br>GGGAGCCGTGGCATGTGTGGGGTCCCCG<br>TGCTGGTTACTGGCAGGCCCTGGGCTCCT<br>GGGGCAGCACTGAGCTGTGTTCACCCCA<br>CCTGCTGAGCCAGCAGCATCTGGGTGGG<br>GGCTCTGTCAGCATCTGGACCGATCCCCT<br>TCCCCTAAGGGGGGTGGGCTCCGTGTCT<br>CCCCATCTCACACGCAGTGGGACGGCAG<br>CGCGTCTCAGACATGTGACCGTGTCCTGT<br>TTGgaaaagtttccgaaaaatcATCCTGAGAAGAA<br>GCTGTTTTCTCATCGAATATTATCGAGGG<br>ATGAATGTATTGACCCGTTTTCCAAAACA<br>GGGAACCTTAGGTAGGTACCTGGCTGTG<br>CACTTCCGGTTGTAAAGTCTGCATCTGGA<br>GCATTAGGCTGCGGGTTCCGTATCTCCTC<br>TCCCTGTCCCCAGCGTTCACTCTGGCGTG<br>TGGATAGCCACTGTCTTCTAACTActgggaag<br>ggaagggacgaaaCCCTGAATGTCCAGGCAGC<br>AGTGGCTCCTGGGGGGCTCAGGCTGGAG<br>CCTGGGATCGCACATGTGGGAGTGAGCA<br>CGGGGTGAGGGCGCGAGCGCCTGGGGC<br>GGAGCGCCTGCAGCCCCCTGGCCGCCCC<br>TGGGGCCTTGTTCCTTCAGGGCCTTGCGT<br>ATGCTGACCCTGCCGTCAGCGTCCTGCCT |

| | |
|---|---|
| | CCTTCCACCCCACGGGCCCCTCCGGTCCT GGCCCCCGACCCCTCACTGAAGGCCTGT TTTATATTGCGAATTTCTTGCTCAGCCTGT ATAAGGAATTTCCCTGTTTTCTACCTAGA GAGAGGCAGCCTCCCCCCGATAAGGGGG TACATACCCCTATCTCGGGTGTGGTCTTTT GGCCGGTGTTGGTGACCCTTAGCTGACAT TTCGAGTTTCAGGTTTAGGATGTCCTATT CACGTCTGTAGTACATAAGAGAGCCTGG GAGTACTTGTCCGGAGACAGCGCTCGTG TTTACAGGTTGAGCATCTGGATGAGCAG ATACAGGCACAAGGGAAGGTGTGATGCT TGTCAGGGGAGAAGCTGCCTGAGGACG GGGTGTCCAGGGACAGGGCTCCTGGCAG CTCCAGGCGGGGGTGCTTGGTGAGGCAG GTTTCTGGCCGCAAGCTGTTCCCTGGTCT TGCTGGAGTTAGATGTGCTGAAAGCATC ACCAGCGTAAGTCACTGTAGGCACCTTG TGCCGTGGAAACAGACGCACGGAAAGT CATTCCGGACATGTGAGCCTTCTTCCTGC CTTTAGTGGTAGCTGGAAAACCTGAGCA GTGGCAGGCGCTTCTGCTGGGTTTGTTCC TCAGGCCTGGAAGATGGGCAGGAAGCAC CATCTTCTGACTCTCTGTCTTCGCACTTG CTTGTCTGAATGTCGGCCTTGGGGGCAG AAACAAGGTGACGAGACCCCAGAGTGG GCCTGTGTCCCTCGGGCTTTCTGGACCCT TGCTCCCCTGGCACCCCAGCATCACTGTT CTGTCCTGACGTCACGTATGTGCCCAGCT GAGACGTGAGTGGGGCTCTGTCTCCCGC CTGGAGCCGGTAGCTCTCACATGGCGCG GGCCGATGGCCTGCTGGGTCAGGCCGCG GCCGGGCCTGTCGGTCCACGCCAGCTCC CCCAGGCAGGGCTCTCCCCAGCCCTGAC GCCTCACTCTGTCCTGCTCCTGCCCGGAG GAGCGCCAGACACGGGAGGAGTGTTAGC AATCTCAGTGTCACTTTCTCTGGAAATCT TGAAAGTCTTGTTTTTTAAACAAGCCTTT CCGTGCAGCTCATGCCTGGTTCTGGTTAG CCTGCTCTCCCTTGGTTCCACGTGAGAG ACAATGTAGATGTTCGGCGGGTGGTGAC TTCCATAAAGGGTACTAGTGATGCATATTT ACTTCCAGAAATCTCTTTCCCTGTGGAGA CTCCATGGTTTGCATCATCGACGACCGAG |

| | |
|---|---|
| | AAGACGTCTGGAAGTTCGCCCCCAACCT<br>GATAACCGTGAAGAAATACGTCTACTTCC<br>AGGGCATTGGGGACATCCATGCCCCGTCT<br>GCGCCCCGGGAGCCCCCAGCGAGGAAG<br>AGAGGTGGGTGGGCCCCTGCTGTCCCTT<br>CTCAACTGTGGCTACTGCTCCTTCCgtcaac<br>aaaaaccaaaacaaggaaataaacacaTTCAGCCTCAG<br>AGAGGCCCTGTGGGTTTGTTTGTTACACT<br>GATACTATGTGGTTTCAATGTAACAGTGT<br>TAGCCTGAGCGTCTTTCCTGTTGAGTTGA<br>GTGAAGAATGCTACTTGACTTAATGCATC<br>TGAACTTGCCCCTCTTCCTGGTTCTCCCT<br>GGAAAGAACAGCGGGGCCCAGGGGTGT<br>GGTGTGGGGGCTCCCGCAGAGGCAGGG<br>CCATTGCACCCTGCCCAGACCTACACGG<br>AGCCCCTCCGGCCAGTCGAGCCCAAAGT<br>CTGGAGAAAGTTTGAAGTTTCGGTTTAA<br>AGTGTTTCTGATAATCTaatcgaaaatttaataaaact<br>cttaACAGTTAACTGCTGCACTGGCTGAAG<br>CAGAACTTTGAGcggtccgtctcccctccctccgggg<br>AGGGCGACCCGCACGGCTGAGGCACGG<br>GCCTTCTGACCGTAGCCTGGGAGCCAAG<br>AGCGGTCCACACGTCTCGGACCCTGTGG<br>AGCCCCTAACCCAGTGACCAACGAGGCA<br>GCCAGCCTGGAGCAGTCACCCGCTGGCC<br>GCCTCCTGGTGTGTGCCACCGTCCAGAG<br>GTGACTGGAACCGCAGGCACGGGCTGGC<br>GGGCAGTGTGTCCACAGGAGGCTTCCTG<br>TTGCTCGCTGAGTAACGGTGGCCTTGTCT<br>TCTGTGTCCTCACCTGGTCGATCGTCCTG<br>AAGGTGCAGACGCCGCAGAGCAGCCTCT<br>GTCCATCAGGGACCCCGAGGAAGGAAG<br>GCCAGCGTCCACGGTGGAGAGTAACGGC<br>CTTGGGAGGCCCGCCCGGGAGCTCAACG<br>GGGGCCCGGGCCCTCGGGGGTGCTGGCC<br>Cacggtcctcctcctctccccgggccggcccactgcc<br>CGCACCCCCTCCGCTGACGGCCGCGGGA<br>CTTCGGCAGGTAACCTGCCGCGCGGAAA<br>GACCCCACCAGAAAAGAGGCCTGTGCG<br>GGGTCCAGCCGGCCCCGACGCGGACCCG<br>GACGTGCCCAGCGACAGCGGGAGCAGC<br>AGCGAGTCCGAGGGCCAGCCCTCGCCTG<br>CCCCCTCCGACGGGGAGAGCGGGGAGA<br>GGCGGACCCGGAGGAAGCCCCAGGCCG |

| | |
|---|---|
| | CCCGCGACGCTGGGAAGGCCATGCTGCA<br>GGGAGGCCCCCCAGGTCCGGCCGGGGA<br>GCGGCTCGCGGGCGCCAACCACTGCGGG<br>GAGCCCAGCCCCGGCGTGCCGGCGGACG<br>CGCAGGAGGACGGAGAGCGGGATGGGC<br>TGTGCGCCCTGGGCGGTGGCTGCGCCGA<br>CCGCAAGGAGGCCGAGACCGAGTCCCA<br>GAACAGCGAGCAGTCGGGCATCACGGTG<br>GGTGAGTCCCTGGACCAGAcatacctcctcctcct<br>cctcctcctgcgcGACGACGACGACCACCTGGT<br>GCACCTGGATTAAATCCTCTCCCGCGTGC<br>ACTCCGACTACTATGCCAAGTATGACCGC<br>TTCCTCAGCGGCGAGAGCCCCGAGGCCC<br>CCGACATCCGCAAGATCGTCCCTGAGCT<br>GCGCAGCAAGGTGCTGGCGGATGTGGCC<br>ATCATCTTCAGCGGGCTGCACCCCACCA<br>ACTTCCCCGTGGAGCGCACGCGGGAGCA<br>CTACCATGCCCGGGCGCTGGGCGCGCGC<br>ATCCTCACCCAGCTCGTGCTCGACCCGG<br>ACGACCCGGCCCGGCCTACCCACCTCAT<br>CGCCGCGCGGGCCGGTGAGTCACCCCCA<br>GCCCGCCCGCTGGTGAGCGCGGGGTCCA<br>GGTGTCCCCGAGGCCTGAAGCGCCCGGC<br>CCTGCCCTTCCGTGTCCCCCACCCTGAAC<br>CCCAGCAGCCCTGAGCCCCAGCAGCCCC<br>GCCCCTgacacggggacgggggtcggggtcgaagaCCC<br>CTCTGTCCCTGCCGCCCCCGAGGCGCCC<br>TGCCCCCCATCCCGTGGCTCTGAGTGGAT<br>TGAGTTTGCCTGCAGGCACGGAGAAGGT<br>GCGCCAGGCCCAGGAGTGCGGGCAGCT<br>GCATGTGGTCAACCCCctgaccgacacctcgacgg<br>accAGCGCTGGGACAGGGTGGAGGAGCA<br>GCTGTTCCCGCTGCGCGATGACCAGGGC<br>AGGACACAGAGGTGAGCTCCAGGCGGC<br>CCGGTCCCCGACGAGCAGGTGCGGGCGG<br>TGGGCCTGCTCAGCGCTTGCAGCCCGCG<br>GCTTCCCATCTGCGGAGACACCTGCCTGa<br>gagaggggacagggacgactGCGGGGTGTCACCG<br>ATAGCCCGTCTCCCTGGTGCCACCTGGA<br>GAAGGAGCAGAATCGCTGCTTCTAAGAC<br>ACCAGACAGCATTCTGCCATGATTCCTGG<br>TTTACAGGTGTTTTGAGGCTGGGAATGCT<br>CTGTCAAGTCCCGGGGTGTCTGCAGAGT<br>CCAACATGGAGGGAGGTGcaccgaccccctccc |

| | |
|---|---|
| | gtccTGCGGAGGTGTGTGGGCCGCGGTGTTGGGGGAGAGGGCTCACGGGCACCCCTCTCCATGAGAGCAGGTGCTGGGTTTACAGGCAGGGCTGCAAACAGCCTGAGAGACAGCAGTGCCGAGAGTGCCGGCCTGTGTCCAAGGCTGTGGCAGAGCCCCAGGCCTGACCAGGAACCACATGCCCCCTGCAGAGTCATGTCCCGGTGGGAGGGGTTGCCGGGGTCCCATGGGTTCCAGCCCCTTACACGGCCCTCCTTCTCACCCGTAGGGAGGACAGCCCCGCGTCCTTCCCAGACAGGCAGAGCCTGGTCCCCACCGCCCTGTTCCACCCGACGCCTGTGCACCCGAAGGCTCCGCCTGGTCCTGAAGTCCGGATCTACGATGCCAGCACGGGCAAGCTCATCCGCACAGCCACCTCGGGCCCCGGGCCGCCTGGCGCCCTGCCCCACGCAGGGCTTTCCTCCTTCAG |
| AC_000165.1:40621610\|40622735 | GTCCCTTATTTCACGAGAGTCTTTGGCCTCCTCAAATTTGAGTCTGACAGAAAGTCAGTCGACCTTGAACGTGAAACAAGAGTGGTCGCACGGCTATAGGGCTCTCCCTTCGCTCTCCTCGAACCACAACTCTCAGAATGGCACTGATCTAGGGGACCTAATTAGCCTTCCTCCTGGGACATCCATGTCCAGCAACAGTGTCTCTAACTCATTGCCACCCTACCTTTTCGGTATGGAAAATAGCCACTCTCCTTACCCCAGTCCCCGACACTCTTCAGCCAGGTCCCACTCGGCCCGCTCCAAGAAGAGAGCACTGTCCTTGTCCCCGTTGTCCGATGGCATCGGGATAGACTTCAATACCATCATCCGCACCTCGCCCACATCCTTGGTCGCCTACATCAACGGTTCGAGGGCTTCCCCCGCCAACATGTCTCCACAGCCTGAGGTCTACGGGCATTTCCTGGGAGTGCGAGGCAGCTGTATTCCCCAGCCATGCTCAATGCCAAGCAGCCAAAAGGGTGTGCGGGTGGCCAGCGGCGGCCTGGCTCTCCCGGCCTACGATGAGGACGGTGCACTGGAGTATGAGCGCATGCAGCAGCTGGAGCACGGCGGCCTCCAACCCGGCCTGGTCAACAACATGGTGGTGCAGCACGGCCTGCCGGACCCTGCCGGCCACGCGGCCGGCCTGCTGAAGACGGAGCGCCtggatgacttccctggtagcgTCCTGGACCTGCCCCCCGCaccctctctgcctcctctgccaCCGCCGCCCCAGCCCCA |

| | |
|---|---|
| | AGGCCCCCCGCCACCCTATCATGCCCACC CGCATCTGCATCACCCAGAGCTGGTCCA CCAGGCCCAGCCGCTGGCCCTGCCTCAG GCCGccctggaggaggatggggaGATGGATGACG TTGGGGGCAAGCACTGCTGTCGCTGGAT CGACTGCAGTGCCTTGTATGACCAGCAG GAGGAACTTGTGCGGCACATCGAGAAGG TTCATATAGACCAGCGCAAAGGCGAAGA CTTCACTTGTTTCTGGGCCGGTTGTCCTC GAAGGTACAAGCCATTTAATGCCCGCTAT AAACTGTTGATCCACATGAGAGTTCACTC GGGGGAAAAGCCCAACAAGTGTTCG |
| AC_000181.1:934078\|947669 | GAAATCTAAGCATAAGTACGTAAGCGTAT GTTTAAGACAAAACAAGGAAGTCTGAcca tcttttctcttccttcacaTCCCGCTGTTTCTGCTGTA ACAGAATAGCTCTTGGCCTTTCTGCATAA ACTCTTTCGGGAGCACAAGGTGGGATTC CTGTCGCTCATCAGGACGGCCGCAGCCG CCCCGCGGCTTACAGAGTCTCAGTCCTC CAGCACCCCGCCCTGAGCGAGTCCGTAT AGACTTAGCCTCTTGCCTCTCCCCCCCGC CCCGATCACTGGTCCACATGTAACCAGAT GCGGTCACGGTCAGACTGGCTTGAGGAC ATTCGAGCAAATGGAACGGGCTTCAGGC ACCAGGGCATCGGCTCCGGGCTGCTGGG CGCGTGGGTGCAGCGGGCACCCCCAGCT TCCTGTTCTGTTTCCTGAGAAGACGGAG CGTCACCTTGTCGCCACACAGCCTTCCTC AGTCATTTCTCTTTGCCTCCCTGGCTGTA GCAAGCAGAAAAGCTGGGGAGGGAAGA CCAGCAGCGACTGCACCGCAACAGGAA GCTGGTGCTCATGGTGGACTTGGACCAG ACGCTGATCCACACCACGGAGCAGCACT GCCAGCAGATGTCCAACAAGGTGAGCGC GGGCGGCACTGCCGTCCTGGGAAGGAGC CTGGTGTTCCGCTTAGGAAGTGAACAGG CTCTGCCCCTGAGCTCACGGCCACGCTG GGGGACAGTGGAGAGCTGGTGGGGGCT GGAGTGGACCCTCTCTCCCACAGAGCAG TGCAGGTGTTCGGATGGCTAGTGTTTCTG TGTCGTGGAATCTTCTGTCCTGTGACAGC TCTCCATGCCGTCTGTGCCATCGCCTGTG GACATGTCTCAGGGTTTCCTTATCACGAC CACCGCATTCATGGGCGGCAGAGgacccacc |

| | |
|---|---|
| | gtccccaccccacggGGGGTGGCAACTGCAGCC ACTCGGGGCTTGTCTGCTGCGGAGCCGG ACATTTCGTTTCATAAGAGTTTGAACGGT TGCATCACACCCACTTGCATGTGTGGTAG CAAGGTGTCCTTGGGGCCGCGTGGACCA TGCCCACTGCCGGGGGCTTTCCTGTGCC CTCCCGATTCCCCACCACCACAGCTCCCA GACCTGCGGGCAGCACTTAGAGAGGTGG GTTGAACCCTTAACCTCAGATACCCTGAC TTGGGATTTGAGGATTTTTCTGTTGCAGT GTCCAGACGATTTACCGCTGAGCGTGcccg accaccacccccccccgccaccaccaaGTACCATCTTG ACTCCCAAGTGTTTTACCAGAGTAGCCCT GTCGTGAACAGTGTGACAGATTTATAGCC AGTGAAAACTTAACAATGCCTCcccagtaaag gaaaagaaaaactcaaTATTATTACTGGGAAAATG AATGATAAACAATTAAAAGGATCAGAGTT TAACTTTCTAAGGAGAGTAGACTTACGTG CATGTTGATCATGTGTGAGCAGGTTGGAG TTACTGATCTCATCTGTCTCCTAAAGATG GACCTGTTTTTGTGCTGTAAGTCAGTGGA GTTGCATTTTATAACCGTAAGGAAGTTAG GCTGAGTTGTGTGGGGCTCTTAGCTTACC TTCCTCTCTGGCCCCGGGAGGCACTGTG GATAGACCCTGGAATCTGCGGTGGTTGG CTACCTGCCCTGCGTGAAGTGACCCACC CAGGGTGCTCGTGAAATGGTTGGGACGT GAGTGTCCACCGGAATCCTGAGACACTT AGTGAGGCCTCCGGTGCCCGGTCCTCAG ACAACTAAGGCGCCATAGTTGTCACAGG AAGCTATCACAGGTCACCTGGCTATTGTG GGCAGTCGCTGTGATTATCCGGAGTACTC ACCCAGGCGGTTAATGCAGGAAAAGGCT GAGCCCCCTTACAGAGTGTGAGAGAGGA AGGCGTTTGTGTGGCGCGGCAGGCTGTG CTGCCGTGACTTCCCCCCCCATAGCCCCC AGCCTGGCGGCTGTCACCTGCCACATGT CAGCGCTGCCCTCTGACTGGGCCTGCaga gacgtcccgtagaaggtgTTCCAGCTAGGCCGGGG AGAGCCCATGCTGCACACACGCCTGCGT CCCCACTGCAAGGAGTTCCTGGAGAAGG TGGCCCGGCTGTACGAGCTGCACGTGTT CACATTCGGCAGCCGGCTGTACGCGCAC ACCATCGCAGGTGAGCAGCCCATGGGGT |

| | |
|---|---|
| | TTTGTCCCACTCTGGATACTCAACGGCCG GCGTGTGGGTTTGGCTCCTCTCCTTCTCA AAGGTGGGTCCTGCAGATGGAGGGAAG GTCAGAGGCTCTGCCTGACTCGGCCTCG GCCCCGTGGTAGTGGCTGGGGCGCGGGC AGGCCAGTCCTCCAGAATCTCTGCTCTGC TCGGCCAGCAGTCACGCTCCAGTCCCAA GTGCAAGCCCCTGTCCCTCTGTCAGCTG CACGCCGCCGGCCCCTGGAGAGGCTGGT CAGGATCACAGACGCTCGGGGAAGTAGG AGCTCTCTATAAGAGCTTTCTTATACAGG TAGAGACCCTCGGGGCTGCCAGCCTGGT CACCGGGGACATGTCTGTCTCAGTAGAC AGCAGTCGGCGTGGGTGTCAAGTCTGTG ACCTTGGGGTCCTGTCACAGAGGGCGGG GGCTCTGGTGGGCACTCAGTGAGCCTGG GAACAGCTGAGCACTGGTGCCGTGCGTG GCTGGGCAGGTGCTTCCTCGTCCATGTCC ATGCAGTCCTGCTGTCAGGGGCGAGTTT CAAAGCACTGCCTGGATGCCCTGCTCTC CCCTCTGCTGTACGAACACGACGGCCCA GGACATGCACAGGCCACGGGCCCCTCTC GGGAGCCGTGGCATGTGTGGGGTCCCCG TGCTGGTTACTGGCAGGCCCTGGGCTCCT GGGGCAGCACTGAGCTGTGTTCACCCCA CCTGCTGAGCCAGCAGCATCTGGGTGGG GGCTCTGTCAGCATCTGGACCGATCCCCT TCCCCTAAGGGGGGTGGGCTCCGTGTCT CCCCATCTCACACGCAGTGGGACGGCAG CGCGTCTCAGACATGTGACCGTGTCCTGT TTGgaaaagtttccgaaaaatcATCCTGAGAAGAA GCTGTTTTCTCATCGAATATTATCGAGGG ATGAATGTATTGACCCGTTTTCCAAAACA GGGAACCTTAGGTAGGTACCTGGCTGTG CACTTCCGGTTGTAAAGTCTGCATCTGGA GCATTAGGCTGCGGGTTCCGTATCTCCTC TCCCTGTCCCCAGCGTTCACTCTGGCGTG TGGATAGCCACTGTCTTCTAACTActgggaag ggaagggacgaaaCCCTGAATGTCCAGGCAGC AGTGGCTCCTGGGGGGCTCAGGCTGGAG CCTGGGATCGCACATGTGGGAGTGAGCA CGGGGTGAGGGCGCGAGCGCCTGGGGC GGAGCGCCTGCAGCCCCCTGGCCGCCCC TGGGGCCTTGTTCCTTCAGGGCCTTGCGT |

| | |
|---|---|
| | ATGCTGACCCTGCCGTCAGCGTCCTGCCT<br>CCTTCCACCCCACGGGCCCCTCCGGTCCT<br>GGCCCCCGACCCCTCACTGAAGGCCTGT<br>TTTATATTGCGAATTTCTTGCTCAGCCTGT<br>ATAAGGAATTTCCCTGTTTTCTACCTAGA<br>GAGAGGCAGCCTCCCCCCGATAAGGGGG<br>TACATACCCCTATCTCGGGTGTGGTCTTTT<br>GGCCGGTGTTGGTGACCCTTAGCTGACAT<br>TTCGAGTTTCAGGTTTAGGATGTCCTATT<br>CACGTCTGTAGTACATAAGAGAGCCTGG<br>GAGTACTTGTCCGGAGACAGCGCTCGTG<br>TTTACAGGTTGAGCATCTGGATGAGCAG<br>ATACAGGCACAAGGGAAGGTGTGATGCT<br>TGTCAGGGGAGAAGCTGCCTGAGGACG<br>GGGTGTCCAGGGACAGGGCTCCTGGCAG<br>CTCCAGGCGGGGGTGCTTGGTGAGGCAG<br>GTTTCTGGCCGCAAGCTGTTCCCTGGTCT<br>TGCTGGAGTTAGATGTGCTGAAAGCATC<br>ACCAGCGTAAGTCACTGTAGGCACCTTG<br>TGCCGTGGAAACAGACGCACGGAAAGT<br>CATTCCGGACATGTGAGCCTTCTTCCTGC<br>CTTTAGTGGTAGCTGGAAAACCTGAGCA<br>GTGGCAGGCGCTTCTGCTGGGTTTGTTCC<br>TCAGGCCTGGAAGATGGGCAGGAAGCAC<br>CATCTTCTGACTCTCTGTCTTCGCACTTG<br>CTTGTCTGAATGTCGGCCTTGGGGGCAG<br>AAACAAGGTGACGAGACCCCAGAGTGG<br>GCCTGTGTCCCTCGGGCTTTCTGGACCCT<br>TGCTCCCCTGGCACCCCAGCATCACTGTT<br>CTGTCCTGACGTCACGTATGTGCCCAGCT<br>GAGACGTGAGTGGGGCTCTGTCTCCCGC<br>CTGGAGCCGGTAGCTCTCACATGGCGCG<br>GGCCGATGGCCTGCTGGGTCAGGCCGCG<br>GCCGGGCCTGTCGGTCCACGCCAGCTCC<br>CCCAGGCAGGGCTCTCCCCAGCCCTGAC<br>GCCTCACTCTGTCCTGCTCCTGCCCGGAG<br>GAGCGCCAGACACGGGAGGAGTGTTAGC<br>AATCTCAGTGTCACTTTCTCTGGAAATCT<br>TGAAAGTCTTGTTTTTTAAACAAGCCTTT<br>CCGTGCAGCTCATGCCTGGTTCTGGTTAG<br>CCTGCTCTCCCTTGGTTCCACGTGAGAG<br>ACAATGTAGATGTTCGGCGGGTGGTGAC<br>TTCCATAAAGGGTACTAGTGATGCATATTT<br>ACTTCCAGAAATCTCTTTCCCTGTGGAGA |

| | |
|---|---|
| | CTCCATGGTTTGCATCATCGACGACCGAG<br>AAGACGTCTGGAAGTTCGCCCCCAACCT<br>GATAACCGTGAAGAAATACGTCTACTTCC<br>AGGGCATTGGGGACATCCATGCCCCGTCT<br>GCGCCCCGGGAGCCCCCAGCGAGGAAG<br>AGAGGTGGGTGGGCCCCTGCTGTCCCTT<br>CTCAACTGTGGCTACTGCTCCTTCCgtcaac<br>aaaaaccaaaacaaggaaataaacacaTTCAGCCTCAG<br>AGAGGCCCTGTGGGTTTGTTTGTTACACT<br>GATACTATGTGGTTTCAATGTAACAGTGT<br>TAGCCTGAGCGTCTTTCCTGTTGAGTTGA<br>GTGAAGAATGCTACTTGACTTAATGCATC<br>TGAACTTGCCCCTCTTCCTGGTTCTCCCT<br>GGAAAGAACAGCGGGGCCCAGGGGTGT<br>GGTGTGGGGGCTCCCGCAGAGGCAGGG<br>CCATTGCACCCTGCCCAGACCTACACGG<br>AGCCCCTCCGGCCAGTCGAGCCCAAAGT<br>CTGGAGAAAGTTTGAAGTTTCGGTTTAA<br>AGTGTTTCTGATAATCTaatcgaaaatttaataaaact<br>cttaACAGTTAACTGCTGCACTGGCTGAAG<br>CAGAACTTTGAGcggtccgtctcccctccctccgggg<br>AGGGCGACCCGCACGGCTGAGGCACGG<br>GCCTTCTGACCGTAGCCTGGGAGCCAAG<br>AGCGGTCCACACGTCTCGGACCCTGTGG<br>AGCCCCTAACCCAGTGACCAACGAGGCA<br>GCCAGCCTGGAGCAGTCACCCGCTGGCC<br>GCCTCCTGGTGTGTGCCACCGTCCAGAG<br>GTGACTGGAACCGCAGGCACGGGCTGGC<br>GGGCAGTGTGTCCACAGGAGGCTTCCTG<br>TTGCTCGCTGAGTAACGGTGGCCTTGTCT<br>TCTGTGTCCTCACCTGGTCGATCGTCCTG<br>AAGGTGCAGACGCCGCAGAGCAGCCTCT<br>GTCCATCAGGGACCCCGAGGAAGGAAG<br>GCCAGCGTCCACGGTGGAGAGTAACGGC<br>CTTGGGAGGCCCGCCCGGGAGCTCAACG<br>GGGGCCCGGGCCCTCGGGGGTGCTGGCC<br>Cacggtcctcctcctctccccgggccggcccactgcc<br>CGCACCCCCTCCGCTGACGGCCGCGGGA<br>CTTCGGCAGGTAACCTGCCGCGCGGAAA<br>GACCCCACCAGAAAAGAGGCCTGTGCG<br>GGGTCCAGCCGGCCCCGACGCGGACCCG<br>GACGTGCCCAGCGACAGCGGGAGCAGC<br>AGCGAGTCCGAGGGCCAGCCCTCGCCTG<br>CCCCCTCCGACGGGGAGAGCGGGGAGA |

| | |
|---|---|
| | GGCGGACCCGGAGGAAGCCCCAGGCCG<br>CCCGCGACGCTGGGAAGGCCATGCTGCA<br>GGGAGGCCCCCCAGGTCCGGCCGGGGA<br>GCGGCTCGCGGGCGCCAACCACTGCGGG<br>GAGCCCAGCCCCGGCGTGCCGGCGGACG<br>CGCAGGAGGACGGAGAGCGGGATGGGC<br>TGTGCGCCCTGGGCGGTGGCTGCGCCGA<br>CCGCAAGGAGGCCGAGACCGAGTCCCA<br>GAACAGCGAGCAGTCGGGCATCACGGTG<br>GGTGAGTCCCTGGACCAGAcatacctcctcctcct<br>cctcctcctgcgcGACGACGACGACCACCTGGT<br>GCACCTGGATTAAATCCTCTCCCGCGTGC<br>ACTCCGACTACTATGCCAAGTATGACCGC<br>TTCCTCAGCGGCGAGAGCCCCGAGGCCC<br>CCGACATCCGCAAGATCGTCCCTGAGCT<br>GCGCAGCAAGGTGCTGGCGGATGTGGCC<br>ATCATCTTCAGCGGGCTGCACCCCACCA<br>ACTTCCCCGTGGAGCGCACGCGGGAGCA<br>CTACCATGCCCGGGCGCTGGGCGCGCGC<br>ATCCTCACCCAGCTCGTGCTCGACCCGG<br>ACGACCCGGCCCGGCCTACCCACCTCAT<br>CGCCGCGCGGGCCGGTGAGTCACCCCCA<br>GCCCGCCCGCTGGTGAGCGCGGGGTCCA<br>GGTGTCCCCGAGGCCTGAAGCGCCCGGC<br>CCTGCCCTTCCGTGTCCCCCACCCTGAAC<br>CCCAGCAGCCCTGAGCCCCAGCAGCCCC<br>GCCCCTgacacggggacgggggtcggggtcgaagaCCC<br>CTCTGTCCCTGCCGCCCCCGAGGCGCCC<br>TGCCCCCCATCCCGTGGCTCTGAGTGGAT<br>TGAGTTTGCCTGCAGGCACGGAGAAGGT<br>GCGCCAGGCCCAGGAGTGCGGGCAGCT<br>GCATGTGGTCAACCCCctgaccgacacctcgacgg<br>accAGCGCTGGGACAGGGTGGAGGAGCA<br>GCTGTTCCCGCTGCGCGATGACCAGGGC<br>AGGACACAGAGGTGAGCTCCAGGCGGC<br>CCGGTCCCCGACGAGCAGGTGCGGGCGG<br>TGGGCCTGCTCAGCGCTTGCAGCCCGCG<br>GCTTCCCATCTGCGGAGACACCTGCCTGa<br>gagaggggacagggacgactGCGGGGTGTCACCG<br>ATAGCCCGTCTCCCTGGTGCCACCTGGA<br>GAAGGAGCAGAATCGCTGCTTCTAAGAC<br>ACCAGACAGCATTCTGCCATGATTCCTGG<br>TTTACAGGTGTTTTGAGGCTGGGAATGCT<br>CTGTCAAGTCCCGGGGTGTCTGCAGAGT |

| | |
|---|---|
| | CCAACATGGAGGGAGGTGcaccgaccccctccc gtccTGCGGAGGTGTGTGGGCCGCGGTGTT GGGGGAGAGGGCTCACGGGCACCCCTCT CCATGAGAGCAGGTGCTGGGTTTACAGG CAGGGCTGCAAACAGCCTGAGAGACAG CAGTGCCGAGAGTGCCGGCCTGTGTCCA AGGCTGTGGCAGAGCCCCAGGCCTGACC AGGAACCACATGCCCCCTGCAGAGTCAT GTCCCGGTGGGAGGGGTTGCCGGGGTCC CATGGGTTCCAGCCCCTTACACGGCCCTC CTTCTCACCCGTAGGGAGGACAGCCCCG CGTCCTTCCCAGACAGGCAGAGCCTGGT CCCCACCGCCCTGTTCCACCCGACGCCT GTGCACCCGAAGGCTCCGCCTGGTCCTG AAGTCCGGATCTACGATGCCAGCACGGG CAAGCTCATCCGCACAGCCACCTCGGGC CCCGGGCCGCCTGGCGCCCTGCCCCACG CAGGGCTTTCCTCCTTCAG |
| AC_000169.1:81157119\|81162748 | CCTGAACACTGTGCCTCCTTCCTGCGTTC AGCGCGGCTTCTGCCTGGAAGGGACAGG CGTCCTCTCCTGGCTGACCTGGCGTGGAT GTGAGACCCCGCCTCGGCCTCGCTCAGC CTCCTCGCGGGCCCACCAGGACTCTGCT CCTGGGGTGTCGAGGACGCCGTGAGCCG CCGGGGCTGCGCGGAGCTGCTCTGCGGG CCAGAAGGCGGCAAAGTGCCGCCCCGG GCTTCTCTCACGCAGCACAGGTTCAGAG CAGATCGTTAAGATTCCAGGACAGGGGC CCTGCACGTGCTGGGCCCGGCAGGCTGC CTGGTGGGTGCCCCGTGCCTGGGAAACG CCGGCGTGTGCCGGAGGGCTGAGGGCG GAAGTGGAGCCCGGCCGGCGCCCCCGG GGCTGGGCGCTGGGCTTGTCCCGAGTCT GGGTGGGAGGCCCCGAGAGGGCACCTG GCACAGCCTCCgcagggcaggcaggaggggcTTC CTGGGGGTCCGGCTGCAGGCGGGACAG GCGTGGGAGCCCTCCTGGCGCAGGTCCT CAGCCCCTGCCCTCCCCGAGGACTCGGG CTCTGACCCCGCAGCTCCTCCGGCCTGG GGCGCTCCCCAGGTGAGGGGCCACCTCG TCTCCCCGGTGGTCCGTGTTTCAGAGATG GCAACGGTGGCCGTTACAGTGGCGTGGC ATTAAGTTTCCTCAGGAACGTCTGGCGTG GGCAGAGCAGGACGGCGGCCAGGCCGC |

| | |
|---|---|
| | ACTGGGAGGCCCCGAGGGGTGAGGGGA<br>CCCGGGAAGGCTCAGAACCTGCGGGGC<br>GACCGTGAGTGGGGACCAGCCTGCTGAC<br>CCCAGAGGCGCCGTGCCCGCCAATGCGG<br>GCGCCGTCAGGAAGCCGCTTTGCTGTCA<br>CGTGCCCGGGGCCTCCGGCGGCGGCAGC<br>AGGAAGCCCTGGGCCGTCGTGAGGGAA<br>GCGGGCGAGGAGGGGCATCAAAGCGGC<br>CGCAGGGCCCGGGAGGGGCGGCCCGAG<br>CCGGGGCAGAGGTCGAGCGGCTTGTTTT<br>CCCACCTGTTTCCTACAGCCCGAGGGAC<br>TTCTGAGGGATTTCCGTGTAACGAGCAG<br>ACACTCGGGCGTCTCTCAGGGGAGAGGA<br>ATGTTAGAGGGGGGAGGCGGTGCCCGTA<br>CAAGGAGACGAGCCCTCCAGAGAGCAC<br>ACGTCACACACGCGTGTGACAGGCCCGC<br>GGCCGCGCGGGGCCCAGGCGTCGCCTCG<br>CGTGGCTGAAGCAGGTCCTGGGTCACGG<br>GCCTGGTGGGAGCCGCGTGTGACGCACG<br>AGGTCGACCGAGGAGAGCTGAGGACAC<br>GGCCGCGGGGGAGGCCGCGGCGAGTGG<br>CCGTCCCCAGGCTGGTCCTGCGCCCGGC<br>CGGCCATGGCAGAGCCGCTTCGCCTAAC<br>TGTGCGACCCAAGCCGGTCGTTGCTCCT<br>GCCTTTTCTAGCGCGGTCTAATCTAACCC<br>TGACGGCAGGCAGTTGGGCGCTGTTCAT<br>GGCTTAAGGATTTATCACTTAGAGAAAAG<br>AGTAAGATCTCTGTGTCCTCGTTTCTAAC<br>TCTTTAGCATTGCCTTGTGTCATAAAATAT<br>TAATCCTGCATGAATATTTTGTAGAGACG<br>CCTCTTTTGTAGAGGTGGTTTTCATGTTA<br>GACGCTGGTGCCCACTTACACCCTTTTGC<br>AGTCTAAGGAGAGTTAAATATTAGCCCTT<br>TTCTATTTGGTTCACCTGGCGTGTGTTTCT<br>AAATGTGGTCCCAGTGTGTACAACAGTT<br>CTAATTCGTAAACCAGATTAAACTCTTAA<br>ATGAAATCCTGCCTTTTTACACTCTTGCC<br>ACCAGGTGgctaagaaaaaatattaagcagGGAAAA<br>AACCAACGATTATGCGGAGGACTCATAAT<br>TACTTTCAATTTCTGCTTCAGCAATGAAA<br>CAGGAAACTCTAGATGGACTGTGTTTCA<br>GTAAGAAAACTGCTGAATAAGTGACAGG<br>CATTTCAAGTAGTGCCAGACCAGATGGA<br>CATTTCcatgagtgtttttgttttcttaaagattGACAATGT |

| | |
|---|---|
| | TAAACTCACCAAATTTTGCTGAAGTGAA ACTTTTTGAgactctctctgagcctgttcCACCTCTG GGCTGACCCCTGGACGTCCACATTATTCC TGACCTGTTGGACTTGAGACAAGGGTTT GGCTTTTGGGCTTCAGGATTCCCGAGGG GAGGGTGACTAAGTCCTGATCACAGCAG GGCCTCCCGGTCCAGGCTTTAAACATCA GTAACGTTGTCTACAGAAACACTGCGTG AGTTCATTTAGAATCCGCTCTAAAACCCG AAAGTAAGATACACCAAGTTGGATGTTA ACTCCTTTGTGTCCCTTTTCATTGATTCTT TATATTAACGGTAAAACACTAGAGGGAA ATCTATGCATGGGGGGTGGTGACACAAC AGGGTAGAAACCCCTTCAGGCTTCTCTTC AGGAATATATCTATTAAAGACAGcacccaacc ccccaccccacactgcTACAGCATCCCGGCCGTG GCCTCGTCCAGTGAAAACACAACCCCAC CAGTTGCTCACCAGTCCTCCTGTGGCCG CTCAACTTCTGTGCCTCCACTTTGCCCTT CGGTGCTCTAATTTATTAACTTTGACAAG TAACCTCTTGGTTGTACAGGATTTTATTC GCAATTCATTTTTCTGATTCAAAGTGAAC ATGAGTGCCTTAATCCTCTAATGGGAATT AATTGCTTGTTTCAGTGCTTAACTCTGAT CCTTTCTCAATCCCCACAGGGACAAGGC TCTTCCTGAAGGCTCTGTGTGTGTTGTTC AAAACACAATCAGAAAG |
| AC_000158.1:107948998\|107975652 | GAtgtaattccttttatttctcatggaatcacttttattctttttcaaagt GTGCCCTCAGGAGTTCATGCTTTTTACAG TCTTATAGATTTTGACTGTCTAGTGTCTGC TTTTACCTTTTCAAGCTGAAGAATAATGA TCCTTTTAGACTTTCTTATTCTAGCAGTCC TCTTATGTCTCCCATAATTTTAGTTGACTT ACTAGTTTGAAGACACCTCACATATGTCT GCTGGGTACTTACATCAGTTTTTATAAggat aaaattaatgtttttttgttttggagtgtgtgtgtttattttctttctaggt TTTAGCAGACACTCTCCCTTTGTATAATA AAGGTTTTAACTAACAATGTTTAAGCTAT GACAGTTATACTGGaagaatttttttcagtaatgGTCT AGTGATATCAAGGTTCTTTTGTTTAGATTT TACTTTGAATTCTGAGTATTCCAATGCCA CTTTGTTACCTATTTGTGCAGACTCATCA GACCCttctataatttcatttttttaccttTCTGGTTATTTG CTTAATCTATCATATTttgaagtattaaaaaaaactTAG |

| | |
|---|---|
| | AACATCCCAGAATAATTATGGCACatagtctct<br>ttcttttttcttagagtTTATTGATTGCTTGGATAGAA<br>AGCATTgatgaaaagagaaggaagtaaaatgattatacaata<br>acaacaaaagagcAAACAACCCAACTAGGATG<br>ATATGGTAATATAGAAATCCATATATAAAC<br>AGGAGAAAGCATCTCTCATAAAAGACAA<br>TCTAAAGTTCCTTCTGTAGGATACACAAC<br>TCTGCTAGGTAAGCGCCTGTTCTAATGGA<br>GGAAGTAAGGGCCACTTTAGCACCTCAA<br>TTTATGGAAATCTACTTGATAAATCAGAG<br>ACCAAAAAATAGGTTAAAGTAAGAATTT<br>TAGGGTTTATATCTTATCCATATCACCTTG<br>TGTGGGCAGTAAATTGTAACTGAATTACT<br>TCCTGAATTTAACGTCAGGAAGTTTGGTA<br>GGATTTTAGTTTCAGCTATTAAGCTGTAA<br>ATTTTTTCCTGGGTAAATTATTATGTCATT<br>TAAGCAGGTATTTAACTGTTATATTTCAGT<br>GTTCTTTTagcagttgatttttaaaacttatcaaAATGAT<br>ACATGCTTATTCTAATAACAATAcagatatatgt<br>aaataaaacaatacatatttccctttctccccacttATCCTGC<br>AACAGATTAGATCTTTCATCCTTTTAATCT<br>GTTAGGTGTCTTTCTGCATCTTTTGGCCTT<br>GTTTATAGAATCTTttgtaacacattttaaaatttttcgtgt<br>attaaatctttgtttttttctcttttggcacCTGGGTTTCTTAT<br>CCTGCTTTCCATTCTTCTTACAACTGTTTT<br>GATAGAtgcttattttcctttccttttatggaaaaaatatgaaagt<br>cCTACACTGGAAAAGTAGGTTTTGAATCT<br>TGAAACATTTGACCTTATTTTATATTGTTC<br>TTATAATCTGCATCTGCAAAATATTGTGTT<br>TGTTAAAACTGAAGACTTTTGTGTGTTTT<br>ATGTCTCCTTAATATGGTGACACATCAAG<br>AATTAGTGAGCTGTGTGGGCTGGACTACT<br>GCTGAAGAGCTGTATTCATGTAGTGATGA<br>TCACCAGATCGTGAAGTGGAACTTGTTA<br>ACCGGTGAAACAAGTCAAATAGTAAAGC<br>TGCCTGATGATATTTACCCAATAGACCTTC<br>ATTGGTTTCCAAAAAGTTTAGGCATAAAG<br>AAACAAACTCAAGCAGAAATCTTTGTCC<br>TCACAAGTTCTGATGGTAagtttttaataaacattatt<br>tgctcatctttgtttttaaaagacaccGCTTCTTATTATAA<br>CTTATGTTTTGAGTTATTTGTCCATTTCCT<br>GTGTGGTCAGTTGGTCTGTGTATAACTTT<br>AAGACCAACTGTCATAGAAGAAAGTGAG<br>TTAAACTGGCATATACTGAAATTGAGTGT |

| | |
|---|---|
| | GAACCTTGGGCTTTACTTTTTCTGTGTTC<br>ACAGATATTGTTGTGCTTTTAACAGTTTT<br>GGTTTCTAATTTCTTAAAGTTTAACTGTG<br>TAAAATAACCACACTAAAAAGAGGTATA<br>ACACACATTCATTTAGTAAGGTCtctattaaata<br>aaatctttattttctagtACATgctaatttgggcttcccagatggca<br>ctagtggtaaagaacccacctgccaatgcaggagatgtaagagat<br>gcaggttggatccctgggttgggaagatcccctggagaaggacat<br>ggcaacccactccactgttcttacctggagaatccaatgaatggac<br>agaggagcttggcaggctatagtgcgaagagccagacaggact<br>gaagcaacttagcacgcacactaATTTTCACTTACAG<br>AGGTGGAAAGTAGTTTGATCATAGTCTTA<br>TTATTGGTGAGTGAAAAGCaccatttttctactttta<br>aggacatgtttttgtttcttagaaATAACTTGAACTGTT<br>TTCTGTCTGTGCGTGCNNNNNNNNNNNN<br>NNNNNNNNNNNNNNNNNNNNNNNNNN<br>NNNNNNNNNNNNNNNNNNNNNNNNNN<br>NNNNNNNNNNNNNNNNNNNNNNNNNN<br>NNNNNNNNNNtgcgtgcacgtgtgctaagttgcctcagt<br>cgtgtctgactctttgcgatcctgtgggctgtaacccgccagtccat<br>ggggattctccaggcaagaatactggagtgggttgccatgcccttc<br>tccaggggatcttcccgacccagggattgaatccacgtctctgatg<br>tctcctgcaatggcaggcgagttgtttactagtgccacctgggaag<br>ccctttccgtCTGTATTACACTTTATCTATGGTG<br>TCAGTTAGGAGTACTTTCATCAAAGTAAC<br>AGAAACTCATTAGGCTAACAACTTACTTA<br>CGCACAGCTGGAGTTTTCAGGTTTACCTA<br>GCTCCTGGGGAAGATCTTTAAGCACAGA<br>GACAGCAGGCCCCTGATCCTTACCATCTC<br>TTGGACAGACTCTCCCCAGGTGAGCATG<br>AATGGCAGCCAGCAGCCCAAGGCTTAAC<br>TTACAAGAAGAGGAAAGTCAGCAGACA<br>CAGTGCCTCTTCCTCAGTCCCTCTTGTAG<br>TCAGCTTGCAGAAAGGGTCCTGGACAAG<br>CTTGGGTCATCTGCCCATCACTGAGTGAA<br>TCATTTTAGTCAAGGGATTGCGACACTGA<br>TTGCCTGAGCATGGCAGGCCTTTGGCCA<br>CtgtggaaagggaaagagaagagggcttTTGTagccagaag<br>aagaaaaggatgtTATGGCAAACAATCACATCA<br>GATCCCAGTTCTTTTACAGGCAGAATGTA<br>TGGCAGTTTTTAAACTTACTTCCTGAATC<br>TTGATTAGATGAAATATTGTGTGAAAGtattt<br>aatacactgcctagtgCTTACAAGATTGACTCTGA<br>AAAGAGCTAGACTTGTTTACCTCTGATGA |

| | |
|---|---|
| | TAATATGAAGGGTTGACttaaaaaaacttaattttgat<br>ataatttcacATTTATAGAAAAGATGTAAGAAT<br>TGTAGAGATAATTCCTGCATATTCTTTATT<br>CATATTCActtaattgttaacattttgtccCATGTACTT<br>ATCATTGctactcttttctctctctcctgccctccaCACATA<br>CTTTTTCCTGAAACATTTGAGAGTAATTT<br>ACAGACATCCAACTTTAaccatatatcagttcagttc<br>agttcagttcagtcgctcatttgtgtccaactcttcacaaccccacgg<br>accacagcacaccaggcctccctgtccatcaccaactccgggatc<br>tactcaaactcatctccactgagtcgatgatgccgtccaaccatctc<br>agcctctgttgtccccttctcctcctgccttcaatctttgctaacatca<br>ggatcttttccaatgagtcagctcttcgcatcaggtggccagagtat<br>tggagtttcagctttagcatcagtccttccaatgaacacttaggactg<br>atctcctttaggatggacccattggatctccttgcagtccaagggac<br>tcccaagagtcttctctaacaccacagttcaaaagcatcagttctttg<br>gtgctcagcttctttatagtccaactctcacatccatacatgaccact<br>ggaaaaaccatagccttgactagatggacctctgttggcaaagtca<br>tgtctctgctttttaatatgctgtctaggttggtcactttccttccaagg<br>agcaagtgtcttttaatttcatggttggaattgccatctgcagtgatttt<br>agagcccccccaaaataaagtcagccactgtttccactatttcctcat<br>ctatttgccatgaagtgatgggactggatggcatgatcttagttttct<br>gaatgttgagctttaagccaactttttcactctcctctttcactttcatca<br>agaggctctttagttcttcttcactttctgccataagggtgatatcatct<br>gcatatctgaggttattgctatttctcccagcagtcttgattccagctt<br>gtgcttcatccagcccagcatttctcatgatgtataaattaattaagc<br>agggtaacaatatacagccttgatgtacccctttcctttttggaacc<br>agtcttttgttccatgtgcagttctaactgttgtttcctgacctgcatac<br>agatttcttaagaggcagatcaggtggtctggtattcccatctctttc<br>agaattttccacagttgattgtgatccacacagtcaaaggctttggc<br>atagtcaataaagcagaaatagatgtttttctggaactctcttgcttttt<br>caatgatctagcggatgttggcgatttgatctctggttcctctgccttt<br>tctaaaaccagcttgagcatctggaagttcacggttcatgtattgctg<br>aagcctggcttggagaattttgagcattactttactagcgtgtgagat<br>gagtgcaattgtgcggtagtttgagcattctttggcattacctttctttg<br>ggattggagtgaaaactgaccttttccagtcctataaccactgctga<br>gttttccagatttactggcatattgagtttcagcagtttcacagcatcg<br>tctttcaggatttgaaatagctcaactggaattccatcacctgcacta<br>gctttgttcatagtgatgcttcctaaggcccacttgacttgacctcca<br>ggatgtctagctctaggtgagtgatcacaccatcgagattatctgg<br>gttgtgaagctctttttgtacagttcttctgtgtattgttgccacctctt<br>cttaatggcTTTTGCATCTGTTAggtcataccatttctttc<br>ctttattgagcctatctttgcatgaggtgttcccttggtatctgactcta<br>ggtgagtaatcacaccatcatgattacctttGTCATTAACCa<br>tatatactgctactgctgctgcggctaagttgcttcagttgtgtccga |

| | |
|---|---|
| | cttggtttgaccgcatagacggcagcccaccaggctcccagtccc<br>tgggattctccaggcaagagtactgaagtgggttgccattgcctttt<br>ctgttaACCATATATACTTCAATGCATTTCCTA<br>AGAATAAGGATTTTTCAGTAATTTAACCA<br>CAATACAttgatcaaaatcaggaaatttaacACTGATAT<br>AATATTATTACCTAATCTACAGTTTGTATT<br>CAAATTTTAGCAAGTGTCCTAATTATGTCtt<br>ttatagaaattttttttttcttgtaaagaaTAACATTGTTTTT<br>GTCATATCTCACTAACTTCCTTTAATCTGG<br>AGCAAATCCTCCATCTTTCTTTTATGATCT<br>CGACTGTTTTGAAGAATATGGGCCTGTTA<br>TTTTTATAGAATGTCCAATCAATTCACTTT<br>TGTCTGATTTTTCCTTATGATTAAATTCAG<br>CTATACATTTTTAACAGTAATACTTAAGGC<br>ATCAGAGTGGAAAATTCATAATTCTGGTT<br>TGACTCCTTACTGGTGACATTAACTTTGA<br>TTACTTTGAAAGGTTGTGTTCACTAAATT<br>AAtctactgtgaaagtgaaagtgaagtcgtgtctgactctgtgac<br>cccatggactgtagcctaccaggcttctctgtccatgggattttcca<br>ggcaagagtactggagtggggtgccatttccttctccactgtaaag<br>ttaatgtttttccctttagtaaATAATTTCTAAGTGGAT<br>ACTTTCAgactatataaaaatattctcttcctCCTCAAAT<br>TTTCTCCCACTATCTTAGCTGAGTCATTTA<br>TTACTAAGATGGTTGCcgaatgatatttttttaaatgcc<br>atagtATCTTTTATATTGATTAGTTGGTATTCT<br>CCCTCAAAGAAGagccttctgtctcctgcacatttgtttat<br>ttgtttacttacatCAGATTAGACTCATAGatgcttatttt<br>atttcatttttaagttattcattctttttttataattagtggataattgctttac<br>agtgttgtattggtttctgccatatgactgctgctgctgctgttaagtc<br>gcttcagtcgtgtccgactctgtgcgaccccatagacggcagccc<br>accaggtatccccgtccctgggattctccaggcaagaacactgga<br>gtgggttgccatttccttctccaatgcatgaatgccATATGAC<br>AACATAAATCAATtataactatgtgtgtgtgtgtgtgtgtg<br>tgtgtgtgtgtatctactccctcttgaatctcccttccACCCCAC<br>GCCCCATCCTATcccttgttttatttcttgagCATCCTA<br>TTACTATCATTTAcacattttaatgttaaaattgccacagat<br>ttggccagtgggagcTCCATGAAGCCAGGTcaaatg<br>aatttttgttttgcCGTGGGGGATATGTTTCCTCAT<br>CTTTTGAGCACTTTGCTGGCACAAGATGT<br>TCCAGGCTCATTTTGTACTTTCCCAGTCC<br>CAACTTCCAAATCAGCTGTTTCTCCAAGG<br>AGacatgttcttttttgtgtgcttgATGATATCTAAAAG<br>CCAAGATGACAGTGCTAGGTGTGTGCATT<br>TCAGTGGGATATGTTTGCTTCAAGGCCCT |

| | |
|---|---|
| | CTCATAGGATGGAGCTAAGAAATATATGT<br>TGAAAACATACATATCTACAgctatttcttcctccct<br>ccctcgctTCCTttacttcctttcccttcctcctatgtttcctccctc<br>ccttccttccatggTTCCTTCCCGTGACAAAAGC<br>CATAAGTTAATTTTGATACTGCCAATTCTA<br>GTTCAGCACTGCAGAATTAATTCTAGTCT<br>CAATACTTGGAAACAGTGTGGAACCTTG<br>GCTCCCATTATTATCAGTATGTTTACTCAC<br>TTGTTTAATTCTACAATACATAGTAGGTTG<br>TTTTAGAATTGCTAATCCAtacaatttcaaaaaaata<br>aaagtttaatatttgtttttgtgcttgtgtgcttagttgtgtcctacttgtg<br>accccctggactgtaactctccaggctcctctgtccatggaattttcc<br>aggcaagaatactggcataggttgccatttccttctccataagaaac<br>tgtattttatttttaggtttattaCAAAGTAAAAAAGATA<br>AGGTAACCTAGCATAACTAGATGTGGGA<br>AGAAGAGAGAACCAAAGCAGAATAGTT<br>GAGTCTTTTCTCCTAGGTGGAATTACGAA<br>TATCTTGGATCTtgtgaaagagaaagcaaaagatgatag<br>ttcaaaggaaaaaagtggaagagtGAGACTAGAAACA<br>GAATccagaagtaaaaatgaaaaatgaaatgaaaaagaaatat<br>tcaaaaacagaagaaagtggATGCCATTTGAAGGT<br>GGTTGGTCAAGGGTTTCCTGCCAGATAA<br>ACATCTGAGATCATTTTATATGTGGATGCT<br>GTGTTCTCTGTTAATCAAATACTATAAAA<br>AGAAGTATTATTCTGGtttgaaaatatacataatatga<br>gATCTACACaacacatttttaagtgtatagtacaatatttaata<br>attttctatatatttatataaacatggAGAAACGTATAAAC<br>ttggagaaaatatatatatatttatataattttgtgtaaccacaatgttgt<br>acagcagatcgCTAGGTTACAGATTTTGGAGTT<br>CATCACTGTTCCCTTTGTTATACCCTCTCC<br>ATCTTCTGTGTTAgtggtaacattaaaaaaaacaacaac<br>aaacatggGATTTGGGGGTCAGATCCTGAAG<br>TACATAAAGCAGACTCTTGCCAAGTATTT<br>TGTCTCACCAATAATTCTCTCTTCACTGTT<br>ATTACCTCTTTTTTGAATATTTCATGCAGC<br>TGTATCCTACTTTTTAGAGGTGATATTTCT<br>TTCACAATGTGTTAAAAGATTTATATTCA<br>AAGAAGAATTATTTGAAATGCATTAGGGG<br>AACTGCTTTGTAAAGGAATTCTGGTATAA<br>AATCTCTTGTAAATCCAATAAACACTCCT<br>TAAATTATATTTAGTGAAAGTTGAATTTTT<br>TATACATTGTTAAATATCTTAAAGTAAGCC<br>TTTTATTCTCTTATTAGTTAATAAGTACATT<br>TAAAATCccactttaaaaagaatttttgtgttcatttctttttatca |

| | |
|---|---|
| | gaaGTTAGTGGTTACTGATCTCTTTGGCTT<br>TTTACAACCATCTGCCAAAAGATTTCTGC<br>TATAAAGCAGTCTGCAAAAGTGCTCATGT<br>GACCTTTTTTCTAATATCCTATTCCAAGCT<br>GCCTCATGTTTCCATGGACATTTCTTAAG<br>GCATTTCTAACCTTGAGAAATGTGTTTCA<br>TATAATTTATTCTACTTCCTCTGTTCTTTTA<br>AAtagcattttcctctttttagttTATCTTTAATAATAAG<br>AGATAAATTTTTTACATTTGGCTGTAAATA<br>TCCATGTTTTTTACCCAAATCATCCACTGT<br>TTAATCAATGAATTGCTAtcgattaaataaatgaatg<br>aattgtttATATCTCTTCCCAACCCTACCCTTA<br>GCAAATAATTAGAGGTAATTTATTTGGTCT<br>TTAGTTTTTTGGTCTAgcactttaaaaagtttatagaA<br>AAGctgaagggaagaagaactaaaatcgTTAAAGgaata<br>attatgtttaaaaaatttttttgatataTGTAGTTTTATACTT<br>AGGCTTTACTTGGGTTGTGCTATTCAAGC<br>AATGTGTAACAGTCAGGCAAATTACAAA<br>GGTAAAAGATTTATTGAGTGCCAAGTCTA<br>TGCAAAGAGCTAGCCTTAgttgtctcagtgtgtgtct<br>tcTGTGAAGTTGTAGAAAGTAAGAATTCA<br>GCATTTGTCAGAAAGTCAGATTACATATT<br>TTAGGATAAGAAATATCAGTGATCAAAGT<br>acataaaatttctaaatatgttGAAATTTATGGACCTTT<br>CATTTATGGTTCACGAGTTGCTTGGTTAA<br>TCTATTAGATAGATTCGTTTGTGACATGTA<br>GCAATTTAGTATGTAGATATTTTGTGTTAG<br>ACAGCATGCTAACATTTAGTGAAATAGAC<br>TTGGATAAAGTAAATATTGGCCACTCACT<br>ATATGTCATCTTTCTAGTTTCCTCTTTTGA<br>AATTCACCCCCACTCATATTTTCTCTGtaaaa<br>caatgtattaaaaaagtatatccaaattaatttttttagtcTCATAA<br>AGAGAGTACTAGTAAAGTTGTATTCAAA<br>GTAATCTTGTTACATTGAAAAATGACCCA<br>GAggcaaatataaaagtaaaaggaAGATATGAGGAA<br>AAGTAGATGGCACTAGTAGGTGGTTAGAT<br>GAAGGAAAGGAGTAGTTTATTGATGAGG<br>GCCTAGAAAATAAATGATCAgattgaatgagaaa<br>aaaaagaacgtCAAAGTCTTCTTAAGTTACTAA<br>ATATGGTTACTGAGTTGGTTAAACTTAAT<br>GAATGCTGTTTATGTAAAAGCACTATTGC<br>AAATAAAACTATGACTAAGACATGGCCCC<br>TTGAAAAGAATTACAGCCCAATAAAAAA<br>TGCTGCAACAGAGCTACAAATAATATGCA |

| | |
|---|---|
| | ATGTATATAAAATGGAGGAATGATTTTCTT<br>GATGGAAATGGTTTGGTGGTCAGTGAAA<br>TAACCACAAGATGCTaggtataaaatatttgaaaacca<br>tcTGTATtagaattctccagagaaacgTTTTAAAGCC<br>ACCTAAAGCATCtgacttaaatatatttttaatgaattgttA<br>GTAAGAATCTTGAGAAGTCTTAagttaaaaatg<br>aattcaaacaAATCCTTTGAGGCAAAATCTGAT<br>TAGGCAAGccacattattattttatagcaaaaaagatatacatt<br>ttttgggggggggtggatTTCTAATTCTGCTTACAGT<br>TGGCTCTCAGTATTCAAAGGGGTTGGTTT<br>CAGGACCCCTGTAGATACTAAAATCTACA<br>GATGGCCAAGTCCCttgtataaaatggcatagtatttgc<br>acatgacctacacacatcctcccatatacttaaagtcatctctgaaa<br>gtgaaagtgaagttgcttagttgcatgggacttttgcgaccccatgg<br>actgtaacccaccaggctctgctgtccatgagattgttcaggcaag<br>aatactggagtgggttgccatttccttctccaggggatcttcccaac<br>ccagggatggaacctgggtctcccgcattgaaggcagactttata<br>ccctctgagccactaaggaagcaaGTCATCTCTAGAC<br>ATCTCTAGACTGCTTAtaatacctcagttcagttcagtt<br>cagttcagtcgctcagtcatgtccgactctttgcgaccccatgaatc<br>gcagcatgccaggcctccctgtccatcaccaactctcggagttcac<br>tcggactcacatccatcgagtcagtgatgccatccagccatctcat<br>cctctgtcgtctccttctcctcttgcccccaatccctcccagcatcag<br>agtcttttccaatgagtcaactcttcgcatgaggtggccaaagtact<br>ggagtttcagcttcagcatcattccctccaaagaaatcccagggct<br>gatctccttcaggatggactggttggatctccttgcagtccaagaga<br>ctcaaagttgtaaatacaatgtaaatgctatgtaaatagttcaTGG<br>TGCAGGGCAAATTCAAGTTCTGTTTTTGG<br>AGCTTTCTgggctttatttgtttttggtagGTTGAGTC<br>CAAGGATTTCAGAACCCTTGAATATGGAG<br>GACCACCTATACttaattattttatcaaaaaCTATATA<br>TTACTAGATTTCATGATCATTCAAAACAA<br>CACTGCAATTGTTTTGGCTTCCTGTAGTA<br>CTTATTCTTTGTATAGTTGATTTGTTCCCT<br>TGACATCTCTTATATACTTAGCTTAGGTCT<br>TGTAATGTAAGTTTTCTGCTGTTCGCCTAT<br>AAATGCGTGAACTTTCAATGAGAGCAAC<br>CAAATAACCTACTTCTTTTTGTCTACCTG<br>GTGCCTGGTACAGTGTAAAATAAGGCAA<br>GGATTTAAAAAATTTGGGCTTGTCCTTTA<br>AATTAAAGAAGGAGGCCTTTAGACTTGA<br>GGTGACTAATGGTGTATATACCTCNNCTG<br>ACCTCCTGTCAGTGGAGCATTCCTACCCA<br>CTTAAGGTTTGGCACTGCTGAATTGGAAT |

| | |
|---|---|
| | TGATTTCTGTGCAAAtaaactcaatattttttaatatgcc<br>tcagtttttaaatttttttttaacagtgttcAACTATTTAGATC<br>CTCTCTTCTCCATAATTCCTTCTAcaacctgtttt<br>ttaaaaaaacaagttatATAACTTTTTCTAGCTTTT<br>CTAttggagaggcaatggcaccccactccagtactcttgcctgg<br>aaaatcccatggacggaggagcctggaaagctgcagtccatggg<br>gtcgctgagggtcggacacgactgagcaacttcactttcacttttca<br>ctgtcatgcattggagaaagaaatggcaacccactccagtgttctt<br>gcctggagaatcccagggacgggggagcctggtgggctgccgt<br>ctctggggtcgcacagtgtcggacacaactggagtgacttagcag<br>caacagcagcttttctataaccatttttaaaaaaaagcaaaacttcaa<br>aATTCTGCTTAAATAGAAGGCTTTGTTGAT<br>ATTCCTCTTGGGTTCCTTTGACCATATTCC<br>AGGACAATTTTATTGTCCCTCTGAACTAT<br>CATagtatcatatatatattctgtCACACCACTTGTTT<br>CAAggttttataattttgcttttgtgcCTGTTTTCCAATA<br>GATTTGTGCACTCCTCAAGAGTAAGAATA<br>ATTGTATTATTTGCCCTGTATCTCTGATGC<br>CTAGCACCATGCTGACTAGTCTAATAGTT<br>GGTTTTCAACAGATGCTTGTTGATTggataa<br>attaatttaataaaatagattTCTCAAGTATAATTTCTT<br>TTAGGTAAATTTCATCTGATTTCTAAGTTA<br>GGAAGAGTGGAAAAAAGTGTGGAAGCT<br>CACAGTGGAGCAGTGCTGGCAGGACGAT<br>GGAATTATGAAGGAACAGCATTAGTTACA<br>GGTAAGTTATCTGcataaatgacatttaaaatgaatttttt<br>aaagaaaaatttccagaAGAACAGAATGTTTTAAT<br>TTAGCAGTAGTGTCTTTCCTTGTCTAACA<br>GCAAGTAATTGGCACAGGATTGATCTAGA<br>CtgttatataatgttataattcAGAGTGTCATATTAGTT<br>TTCTTACATGGCTAANNNNNNNNNNNNN<br>NNNNNNNNNNNNNNNNNNNNNNNNNN<br>NNNNNNNNNNNNNNNNNNNNNNNNNN<br>NNNNNNNNNNNNNNNNNNNNNNNNNN<br>NNNNNNNNNgagtgggttgccattcctttctctaggggatc<br>ttcctgacccagggatcaaacctgggtctcctgcattacaggcaga<br>ttctttaccatctgagccatcaaaattcaaaattttctcatttaactttA<br>CTCCTTTCTatctctcttccatctccttcccTACTGAGG<br>GTTTCTCATCTTCACCTGTTATATTAGATA<br>TCTGTTGTATGACAGATTATCCCAAAAGT<br>TAGTGACTTAAGATAACATGTATTAGCTA<br>ACAGTTTATGTGAGACAGGGAATGTTTAG<br>CTGGGTGGTTCTGTCTCAGAATCAAGAA<br>GTTGGATGGGGCTGCAGTCATATGAAGCC |

| | |
|---|---|
| | CTCCTAGGGCTAGATAGTTCCAAGATGGC TCACTCACATGGCTGTTGGCAAGAGGTC CTACTTCCTCTGTGACTCTATGACTGAGG CCTAACTTAGTTCTTACAGCGTGGACTTC TCCATAGGGCTACTTGAATGGTCTTACAG TAAGCATGACCGCTGGAGATTCTCCCTGG GTGAATGATCCAAGAGAGAAGGATGGAA GCCACAATATATTTTATGACCCACCCGTG GATGTCATACTCCATCATTTCCACCATATT TtattagaagcaagtcactaagTATAGCCCAAACTGA AGGAGAGATAAATTAAGCTTAACTTCTTA AAAGAGAGAATAACAGAGAATTTGTAGA TATATTTGAAAAGCATGACACCTGTCCCC TGGGCCTTTTGTAGGACTTTTATCAGTGA GTTGTCTCCTGTCTTGACTGTTTATTCAA CTTCTCcttttccagtgttttttttcttttcccagtgtttttttttttt ctttctcccagcaTGTAAATGTGTTTAAATGCTG TTTATCTCGAAAATtgaggaaatagaaataaaaatcctt CCTTTAATAAATAATTCTAGCTacaatttcctctttc tttctttccttttcaggcAAACTGTTCAAAAGAGTA GTCTGTTACTTCACTCCATTTAGTCCCCA ACTCCCTGGAATTTGGCTTTAACTCTTCT TATCACACTGAAAACACTTTTCTTAGAGT GCTTCTCGGATGGAGATGTCAGTGAAAA TGAGAGCATTATTTGAATAATATACTTACA AATCTTCtaagattaaaggaaaaacaattatTGTCATG AAAGTTAGGCTACTTTGATAAAATTGTTT CAAAGTAATTTCAGGTAAATTTTTTAGTT GTGAAGCAGTGACATGTAATTAGAGAAA CGTGTCAATTTTAAATGTGCATTAGTGTA GATAGAGttgatttttatatgtaaatgtaaTGTTTACAT ATATTTCCTATAGGTAAAATGTCAATTATT ATGTTCTTTTTATAGTTGGAGAAGATGGA CAAATAAAAATATGGTCAAAGACGGGAA TGCTAAGATCGACTTTGGCTCATCAAG |
| AC_000159.1:20992753\|21047706 | GTAACATTAACTGCTATTAATATTACTTTT CTACAGATTAACCTGGGGGAAGAAAAGT AGTCATTTAATACGCTTTTTGTTCTCAAA CCCTTTTCATCATTTCACCATTTTTTAGTC TTCTgtatattttctgaaatacagTATAGTATTGATGA GCAGGTTTTGTGTATAATGCTATTTTCTAG TTATGAAAATAATTGACTTTTGTAGAATTC ACGTGTATATCTTAGAATTCACCTCTATAT ACGTTAACAAAATTAAgtattataatttttctctttgtatt |

| | |
|---|---|
| | aaTGCTTCTTTAAGattccagaattttaaaatttgtattgta TTTGACTGGCGATGTGtctatttctgaaaataattacA TCACAAAGCTTTAATATGATAAATCATAA ACTTTGATACTTCTGTTTAATACTGAGTTA ATCTATCAAAACAGATAACAAACTATGTG GGcccatgttcactgaagcatgcTTATTGATGAGTTG AGTCCTGAGTTctgagctttttgtttttcatttcgtGTAA CTTTATTGGAGGTTTTACTAAATCATAACC ACTTGTAAAATTCTTGGCAAGTAAGTATT GCTCTATGGCCAGAGAGTTGTAGAAAAC AGTGACTCTTAAAATTCTACCCTGGATAA ATTCTGTGAAGGTAGAATTACAGATGGTT attgacttcccttgtggctcagctggtaaagaatccacctgctatgtg ggagacctgggttcaatccctgggttgggaagatcccctggagaa gggaagggttatccactccagtattctggcctggaaaattccatgg actgtatagtccacggggtcgcaaagagttggacatgactgagtg actttcacttattgACTCCTGAAGTGTTCCACTGA ACAGTTTGCTTGCCCTTGTAGCAGAAATG GTCTGTACATGTCCATAgagtttcatttcactttctgctt ttcctttgacCACTGGATATAACACAACTTTGT ATAACATGACTCGACCTCCTGCTTTTCAA CTTTGGATTTTTGTAAGATTCCAAGTGGT GACAGTGCCATTTTATGTtccttttacaataaaaata gcatttttgtgACATGTGAACATTCCTCATCTTT TTGTGAGCATATGAAGTTTCCATACAGTA AACCTCGTTAACTTAGATGTCCCTAGCTA TGCAATTTTGGCTTTTTGTTctatatataaaaagag agaaagagattgtgATTAATTCCATAAACTAGAGt cgaaaatatctttaaaaataagttaaaaaattattttcaaatccaCC TAGAGTAtgtgatataatttatatttattaaaactgaTATGA TAAAACCATGAATTTATAAAACCTCCATG TaagcatattttctttaaaaagtattaattttgTTATCAAAAT ACTGGCATTTAttatatgctaagttgctttggttgtatccgac tctttgcagctctatggactatagcccgcctgactcctctgtccatgg gattctccagcaagaatactggagtgggttactattctACAAG GAACAAAACTAGAACTTTTTACTAGTGTA AACTGGCCTGTCCTCCAGATTTTTGAATC AGTAGCATTTGTAATATATTATAAGGGATC ATCTGTGAGGGTATGAAAATCTCCTTATT TGCTTTGCTGATGTAGTTTAACCATATTTC TCCTAGCAGTAATATTAGTTGCTATTTATT ATTacagaaacaaaacacagaaacaccTCAAAGTTAG GTGTTTATCTTTGATTTGGAAATTGAGTG GCCCAAATCTCTGCTCctgtcatttccttccctttcctat |

| | |
|---|---|
| | tCTTTAAACTTTGAAGaattaaagttattattattaaatta<br>ttattaagagatatgagttcaatGACACTCAAAAGAG<br>AGTTTGAAGGGAGTAAACTTTCAGATTA<br>GGAACCAATGCCTTTACTTGGCCTCTGCT<br>AATGGAGACAGAGGCAAAAGACTCTTCC<br>CTTTCGCTCCCTAGCATTCAAGGTATGTA<br>GCATATTCTGACCCAGAGGTTTAAATCCC<br>AAGGACTTGTCTTGCACAGGGATGAAGG<br>AAGACTTGTCTTGTCTTTGGAAAAGGTG<br>GATCCAGACCCCCACAGTCATGGATAATA<br>GAGATGTAAATCAGTGTGGGAAATCACA<br>CCACCTAAACACACATATGCACCCTACAA<br>AGGTACACATTTACTCATTGGTCACAGTG<br>TTGCCTGGAAGCAAGAGACACTAGTTCC<br>TTCAGGACACATAATGTGGGAGTTAGTGC<br>ATTTGCTGTTTTTCCTGGGCTGTTGTCTTA<br>TAATACatagttctattttaattttgatatattcAGACATTC<br>AGCTTTCAAATCCATCTTTAGGAAAGTTC<br>TTAAGTATTATgatagttcatttattttgtgaaCGATGG<br>GGCTTTAGGTAAAATTATGTTTATGGGTAT<br>GTTTCCCCCaaattgtttttccttcattcatacCATTTCA<br>TATATGGTATGCTCTAGGGATAGGCTTccaa<br>agtggctcagtggtaaagaatctgcctgccaatgcaggagacatg<br>ggtttgatccctgggttgggaatatcccctggagaagggcatggc<br>aacccactccagtattcttgcttggaagatcctgtgaacagaagag<br>cctggtgagatccagggattgcaaaagagtcaacgtgacttagca<br>actgattaTGCATGCATGATGTACTTAAGtacatta<br>aaaagatatattttaaggaTAATGTTAGACTATTCTT<br>CTGTTTCTTAGTTTCTTTGCACATGCTTAT<br>CTTATTTAGTAGTTATTAACATTGACTATT<br>CCTGAAGTTTTTACTCTTTATCTTTTCAGA<br>ATCTTTAGAAGAACAAGATGTTCTAGATA<br>ATAGTACAGAACAGACAGATGACAAAAT<br>ACCAGATACAGAGCAGACAAACCCAGTG<br>ATTGAACAAGCATCTGGCATAGAGGAAC<br>CAGAGGAGAAACAAGAAGAGACCGAGA<br>ATGAAGAAACCTCAATGAATGAAGACAA<br>GTCAGCAGTTCCCAGAGCTAGTGCTGTT<br>CCTAATCCTGAACTAAGTGGAGAATCTTT<br>GACAATGTAGTAAATACTTCCATGTGCCT<br>TCAACTGGATATTTGTAGTCTTGCTGATG<br>TCAGTTATTGCTTTTTTAGGtggtactttcttttttcc<br>gcCCCCCCAAAGACATGCTTGGTTTCGTT<br>TGAATTTCAAATTGCCTAGCTATTTCAATA |

| | |
|---|---|
| | TGTCAGACTTACGTCTACTggctattaaaattaaaa<br>gcaaatggtGTCAGTAAAGTAAAATCCTTTTG<br>AATGTACAGAAACCCACACTGTTCAGAC<br>AACGTTtaggtattttttctgttctaaaagtaaacaaatataata<br>gaagttttgttttcttcttgcgACCTGTAAATACATTTG<br>GCATAGGAAGAACTTTGGTTAGGAATAA<br>GTTCTAAtggcatttgttttatttttattttattttttttttatgtatgg<br>cATTTGTTTTAAGGAATGAAACtcaattaagatttt<br>taatacTTCATTATTAAAATGGATAGAATTTT<br>AACTCTTGTGATATTGGCCAGGGGGTAAA<br>AATCTATTTAGGCATTTACATTAAGAGGT<br>GTGAAAGTTATTACAGTAATAATATAGGC<br>ATTTGTGTGAATTCTCAGTAAGAATGCAG<br>TCGAACAGTCAAGTGCAGTGTTACTTTTG<br>ATGTCTCTTATTTTGCAGCTGGGCCAGAG<br>GTACCTGATGTATATAATTAGCTTATGTTT<br>ACATATTAACATATAGGAAATATCTCTGCA<br>CTTGACTGTActtgaattttgaacattatttatatttgtaaaata<br>atgctATTATAAGTGAATTTTGTGCTCGTATG<br>TGTATTTTgttaaggaaaataaagatgatCTGGAAGC<br>AATTTCTTTTTGTTAAATATCTAGACTTCT<br>TCAGACAATAGCTTGGTAGATGACTTAGC<br>TACCTGAAAAGTCTTAGTAGGAAGAGTT<br>CACGATTCAGTAAATCTATAAATTATGCTG<br>AGAAAAAATTATGATCTTTTTATTCAAATT<br>TGTTACCTTTCAAGTTCAGAGAATTAGTG<br>ACATTTAGAGGTTCTAATCCGAGGTTAGA<br>TTTTTCTGATTCAGGAAAACCTTAGGCCA<br>TttgtagtttttaattttgagagaCATAGTTTTGTGAGA<br>ATAGGCAGTTAATTACTAGTAGTTCATTG<br>GATTTAATATGTGTATTAAGGACTAACTG<br>GCAATTTTTTCGACCCAGAATAGTAATTC<br>TtgttatcttttctattttctttttatctattacTAAGAAAAAAC<br>TATCAAGTTATGCCAAAGGAAGGAATATA<br>CAGGAATGGTGTATATGGCAAGTATTTCT<br>AAGTTATATATggtttgttaaaaattaaataaaataaccattt<br>attatcatttaaaaattcatctttatGATTTAATACCTTATA<br>TCTGATAGGTTTTTTAAAGGAACCCAAAT<br>CTGTTAATAGTCTGAGAGTTTTCAAGATT<br>ATAGGATTGGGCTTGAATTCTAGCTCTGC<br>agtcttgggcaagtcacctgaCCTCACTGAGGTcatgttt<br>tttcatctttaaaattggaagaatacCTACTTTTTGGAGT<br>ATGAggacaataaaaagtaattaatgtAGTGGGCCTA<br>GCTGACTGTCTGATAGTAGATATTAAGTG |

| | |
|---|---|
| | GCAGCATTTTATACTCTTGATATCTTTGTG<br>AAATGTGGAAGAGGTCTGAATTCTCCttgc<br>agataaggaaatgaaaaaacaacatgAAATATAATTTG<br>GCTGGTCAGAATCAAGCCAAAGTTTTCT<br>GTATAGGTTTTCTTTatcttatacatgtatataaatgcaT<br>AGATTAAGGCAATAGGAACCTGAAACTG<br>TGGCATATAATAATACTACAGAATATAgtctc<br>ctttttccctttttttgatgAAGCCTATcagtttttttggtgtgtgtat<br>tATTGGCCCATAACACTTTAGTAACACTTT<br>GCTGAGTCTTCTTGTTATAAAACATTCAA<br>ATTCTGAAAAATGAGTAATCTCAGAGAA<br>ATATAATTTAGTGATAGAATTAAGTAGATA<br>TCAGAAGTATCTATGGAAGAAGGTTAAA<br>ATAGGTGAATTAATGTTAACAGGTAACAT<br>GCTGTAATCATGTAGTACCTATTAAAATAG<br>AAACGTTTCTTCTGATATTATAATAAACAG<br>TTAATGGTTGAAGAGTGGTATAGTGTCCT<br>ATTTCTGATTATTAAGACTAGTATAAATGA<br>GGAAAAGCAAACTAATCAGTGCTGTGAG<br>CTAGAGTAACCTTCGCTTCCCTACTTACT<br>AATGCTCAACAGGTCATCATGTAAACTTC<br>TCACTATGCCCTTAGCATAATCTACAAAC<br>ATGTCtggcttttctctctgtctctcttccatCATTATGCTG<br>AAGCCTTAGGGTGTCTTTCAATTCTCTGA<br>CTAAGCTAAACCCTTTCCCACCTTAGGTC<br>TCagtttaaatgttctttcctcAGAGAGATGCCCTCC<br>CAAGCTCTGTATCTAAAGCTGGTCTCTGC<br>CTTTTACTCAATCCTGTCATGTTATTATAA<br>TCTATGAgtattttatctgtttattaGTTTACTGTATTT<br>CTTCAAGACTTAAGAGTCTTTGTGTGCAA<br>GGTTCTGTCTTATTTACCTTTGTATCTCCA<br>GAGCCTGGTACAGTGTTtgacacatttattatttatta<br>ggtGAATGAATAACTTTGTTGCTAAATTCA<br>GCAAAAACTCAGTTCTTACCTAATTCCAT<br>TGACACTCTTGGGTGCTCCCTTTAGTTtgaa<br>acactttttttcttttggcttttgaGTTCTCCCACTATTGTT<br>CTCTTACTCCTCTGCTCCTTTTCTCTCAGT<br>GTCCTCTGCAggtttcccttcctctgcccatCATTTAC<br>ATGTTGGTGTTTCACCTTTCTACCTTGTC<br>AGCATTTCTTCACTCTGTGTATTCTCTCTC<br>TAAAGGCTCTCATCAACCCCCATGGACTT<br>AATTACATCTGTCTCTCTCATTCTCAGATC<br>TTCAGCCTTGATCTCTTTCCTTCACTTTAG<br>AGTGATAGATATACCTGCCTACTGGAATG |

| | |
|---|---|
| | ACTGTCTCTCCCAATCAATTTATTAAATAG<br>TGAACACTATTTTCTCCTTCAAAACCTTC<br>AAAAAGTTTCTATGTTTCAGTTTAATTTCT<br>GTGCTTCTCATCTTATCAATAGTGCTACTG<br>TGCAAGCTAGAAACTAGAGTTTGTCCAC<br>TCATTCCTTTCCCCTTTTTCTTGTACCTTA<br>ACTGTCATTTTCTGTTTCCTAAGCATCTTC<br>CTGTTATATTCTCATTTCTCCATCCCATTA<br>ACCTTACATTAATGATAGTAAACATTTGC<br>ATTCTTACTATGTACCAAGCAACATGCTA<br>ACTGTTGTTTAAGTATTATTTGTTCCTTGG<br>CAACAACCCTGTGAGGATTTTTTTCTCTG<br>GGTTATAGATAGGAAAACATGTATAGAGA<br>GTAAGCAACTGGATAAGTGAAAAATCTG<br>AGACTTGAATGCAGGCAAATAACTtcaggtg<br>gctcagaccataaagaatcaggctgcaatgcaagaaacccaggtt<br>caatccttgggtcaggaaaattccctggagaagagaatggctacc<br>cactccagtattcttgtctggagaatcccatggatggagcagccag<br>gtgggctacagtccatggggtcgcaaagagtcagacacaactga<br>gcaattaacacatacacacacatatgactTTAGAAGTCTT<br>ACTTCTTATTACTGTTGTACTGCCTTCTAA<br>TTTAGGCCTCCTTCATCTCAAAACTGTTT<br>CTTAACTGGTTTCCTTGTCTCCAGTCTTG<br>TCCATGGCTATCTATTCTGTCTACTTCAGC<br>CTGAAAGAGTTTTCATAACTGGAAAACG<br>AATCATGTCATACCATTGCTTAAACCCTTT<br>AATTATTCCTCACTGTTCTTAGAATGAGG<br>TCCAAAGTTACCCATACGGCTTACAAAGC<br>CTGTGTAACTTTGCTCCTAGTTTCTTCTG<br>GCGTTTCTCACTACAGCTGCTGCCACTGT<br>TTCTCTGCCCCTGTACCCCCACACTTTCA<br>GTACAGCTATACTGTTATCTCTGTTTGCAA<br>ATGCAGTGTTTTTTCCTAATCCCCCGCTCT<br>TCCTCTGCCTGGGATATTTTCCCACTTGC<br>ACAGACAGGAGGACACTCAGTTGTTGGA<br>TTCAAGTGACATAGTTCCAGTCAGAATCC<br>AAGTTTAGAAATCTTGCTAATTATTAAGC<br>TATGCTCTTTATTTTTGTACTTGGTCTTTC<br>TCactaccaggcaagaatacaggagtgggtagccattcccttctc<br>caggggatcttcccaaaccagggatcaaatctgggtctccagcat<br>cgcaggcatattctttattgtctgagccactggggaagcccagtgc<br>aTTGGACACACAATCACTATTGTTTGCTG<br>AACATAACTGAATCTGAAGAGCTATCTTT<br>TCATGTAGTGAGGGGAGAGGttctaattatataatt |

| | |
|---|---|
| | ttcattaaacATTATATGCTGGGTGCTAGAAATA<br>CTAAATGTCTTACATGAAGTCATGGGGAG<br>AGTTAGCGCCTAAGCAAGTTAGAACTCG<br>AATCTCAGCTCTTAATGTAGGATGCTTTAT<br>ATTAAACCACTGTTTTTATACTGCCAATAA<br>TTATTGGTGTTAGagtttatgtatttaaaatatgtaacatgt<br>ttatttctatttccagCTGGCAGTAACTACTTAACT<br>ATCTTAaaacttcttttcttattttttcctttattttacctTATAGT<br>ATTACTCTACAATAATGCCTGTTGGACTG<br>CGCTCAAATCAGTTAGTGGAAGGGTCAA<br>AGAATGAGGATAATTTGTGGAATTATAGC<br>AATTTTGAGACAATTATGGATACAGATCT<br>GCTttagaaaattgaaagaaaataatgtggTGTCATTATC<br>TAGTATAACATCAATTCATTGTAAGAATTA<br>TAGAAGAGATTTACATACAGTTTATGAAC<br>TCTTAgagctattaaaataataaaagtatttacATGAAGA<br>CAGACTAAGACAGAGATGATTGTGAATT<br>GATGATCTGCCAAATCTTACAGTTTGTAA<br>TTTGACTTGTTTTAGAattattaaagacctaaatatatt<br>GACTCTACCAAAttagtatattgtcaccctggttatttaact<br>tatatgcagagtatatcatgtgaaacgctgggctagatgaaccaca<br>ggctggaatcaagatttctgggagaaataccaataacgtcaaatat<br>gcagatgacaccagcctcatggcagaaaatgaagaggaactaaa<br>gagcctcttgatgaaagtgaaagagagtgaaaaagctggcttcaa<br>actcaacattcaaaaaagagatcatggcatctgatgatgtcacttca<br>tggcaaatagaggggaaaaaatggaaactgacagacttcattttct<br>tgggctccaaaatcactgcagatggtgattgcagccatgaaattaa<br>aagatgcttgctccttggaagaatggccaacctagacagcatatta<br>aaaagcagagacattactttgccaacaaaggtccatctggtccaaa<br>gctctggtttttccagaagtcatgtatggatgggagagttgcactata<br>aagaaggaaggctgagtgccagagagttgatttttttttgaactgt<br>ggtgttggagaagactcatgagagtcagttggattgcaaggagat<br>caaaccagtaaattctaaaggaaattgttcctgaatattcattggaag<br>gactgatggtgaagctgaaactccaatactttggccacccaatgca<br>aagagccgcctcattggaaaagatcctgatgctgagaaagattga<br>aggcaggaagagaaggcgatgacagaggatgagatgattggat<br>agcatcaccgactcgatggacatgagtttgagcacactctgggag<br>ataatgaaagacagggaagcctggtgtgctgcagtccatgggatt<br>gcagaaagttggacacaacagagcgactgaacaccaccaccaa<br>attAGTAAAGCAATAAAACAAAGAGTTTTT<br>GTTGTGAACTGCAAACAATAAACTTAGTT<br>TTAAATAGTTCATTGCTAGAGCTTCAGTT<br>TCTGTCTCAGTGAAACCCAGTTGAGATA<br>ATCACACAGTAACATGGTCAGAGATAAA |

| | |
|---|---|
| | GAGGAACAGCTGCTAGAAAAAAGACAG<br>GAACCCTAGTCAGTCTTCCTCATAGTCAC<br>CTGGTCAGTTGACTTTATTatgcacacacagaaaa<br>acagaagcaaatgaaACTGGTTAAGTGTGCTGGT<br>GACCTTCTTTAAGGTGATTTATATCCACA<br>GATTCAGGCTCTGAGTTTAGACTGATAAA<br>CttataaatatagaaaagtacATTTATTAGAATATAAC<br>CATAAGAATACAAATAAAgtgaattaaaattattaat<br>gagtaCAGAATTTTTATCAATCCATAAACTT<br>TGTATCAGCCTAATTTCTTTCCTCCTTGGA<br>AACTAATCTACATTTTCTActttaatactattttaaaat<br>gcctttaaaaattatttattagatAGTAGGTGTCCCCCC<br>AGTTACCTTTATCAGTTTACCTTTGTATGA<br>AACTTATATTGCATTCGATTTCCTATTCTT<br>TTTCCTCCAAAGCTCCTGTTATATGTAGTT<br>TAATTTATACTACTCATGggttttaggaaaagaaaat<br>actatCCACTTTCATACCTCTATATTACTTGA<br>TGTTTTTTAAAACCtagattttctgttttatgcatttttctctt<br>ttttaaaaaaatatttgtgccttcctttttttttttttttttggcagcaaaa<br>TTGATAGAATCTGGAAGCACtctgagaagattaa<br>aaaaagttCTCAAAATCTCCCTCATAAATTAG<br>TTTAAGTTCTTATTCCAGGATTATATGAGA<br>ACGGGCCAAATTTTCAACTGCCAAAGAC<br>GTTTACCAGATCCCTGGCACTTGATTTAG<br>GAAAACtgggaaaaatttatttttgtgaaaataagTATTTC<br>TATCTTAAGTCTGGTTTTGAAggatgaagaaagt<br>ggggaattaaatgaacatttttttttgaatACCTGTTCTTGG<br>TCAGGCCCTGTGCCCTGGATACTTTCATA<br>TACATTCTCATATAGTCTCACACAGCCAT<br>GTGAGGGGCTGGAATCCTGTCAAGCAGG<br>GATTAAATGAATTCCCCAAGATTACTTAA<br>TTTATGGATAGTAAATGGAATTGAAACCT<br>AGGATCTCTAAGTTCTGTCCTTTTCACTA<br>CCTATGagttttctcctcctctgttggcTGAAAGAAAT<br>AGACTCAGGCTCTTGGGTTTCTCATTTTT<br>GTGATAACTCAATTGTTACTTCTTCAGGG<br>CAGTCTTCTGTTAACCTAACACTTGTCAG<br>ACTTTCCACTCTGACGTACCCTCCTCTTG<br>AAATTCTTTCATAATATTATCTTTTGCACC<br>AGACTATGAACTCGTTGAAGTCAAGGCC<br>ATATCTGGTTTTGCTTATTATATTCCCAGT<br>GCCTGGTGTATAGAAGAGACAATAAATTA<br>TCTGTTGAATAGTAGTATGCATGAACCTA<br>CCCATATGCACAATGGGAAATACTACTAT |

| | |
|---|---|
| | AGTCTTTAGTGCAGATTTTATAGTGTGAA<br>GGAGTTTGCAAATCTCAAATTATTTGAAT<br>TATTGAGAAGGGAAAGGGCCAGAAAGTT<br>TGTTCCTCACAACTTAGGAAAATTTCAAC<br>TgatgcttttgtcttttttttttttccttttttttaaagtccaTTGTCCA<br>CATTGCTGTTGGAGTGTTCTAAAACACAA<br>ATTTCAAAAACTATTGTCAGGCTCACTTG<br>TGTCTGGCTACCTCCTTGGGTGGCTTGTA<br>AGCATGCCCTTCTTTTCTGGCCTTGTTTC<br>CTACTGTTCTCCCTCGCCTTCTGACCACA<br>CTTCTGAGAGATGCCTCGCACTGATCTTC<br>CATGATGCTACTCCTGTCAACTCTCATTTC<br>CTGAAGAGCTAACTCTGTAATCCGAGTG<br>ACTTCCTCAACCTCCCTAAGCAGTTACTT<br>GCTTTTTGTTCCTATTGTTCTTTATACACC<br>ATGTAGAACTTGTAATCTCCTGACCTCTT<br>GTTTATTGTCTGCCTCCCTCGTTTGATTGT<br>TAACACAGAAAAAAGGCCATGTTGCTCA<br>GTCTTGTATTTCTCACTTTTGCATTCCTCC<br>AACAGAACAGCACAgattaaaacaaaactgaaagct<br>gGCATGGTGTCAGCTACCTGTCCGTTTAG<br>AGAATGGATTCCTGTGATAGACCTGAGTT<br>GATCAGCACTAGTGGTTGGTATGCTACTT<br>CTATCTGTAACTCCTACAAATACAGGAAT<br>TGGGGATACAAAGTGAGAAGGAGTAGCT<br>CAAGATATGAGAAAATGGGAACTGTATG<br>AGCACCTTGAGGATGGGGAGGATTCATT<br>TGTTTTGAATTCTTAATGTCTGAGATAGAT<br>GCTTAATAACTGTGAATGAACCAAGCTG<br>AAGATGGCCTAAGAGCTGGTGTGCTGAG<br>GGGCACCACAGGGTCAGAGCTCTTCCTT<br>GATTCACGTGAGTCTGTTATTTGGGTATG<br>ATTTCTAAATTGTATCTGTAATTTGGGTTC<br>TTTAGAAAATTTCCATTCTTGAAGATCCC<br>TACTCTCCCCACCCTCGATCCCATATTTTA<br>ATTACCTATGgaatggtgtttttaaaaaatctcatttttatgttt<br>attttctcatttctgaccATTTAATGTTGCAACTGAC<br>ATTAAGTTAGAATTTTCTGGATTAAGCTG<br>CTGGTTTTGGACATTGTATCTGTTGGAGC<br>CCAGACacaaaaacatcttaaaattgaGTACTTTTCC<br>TCAAAAAATCATTAAACATGAGGTATACT<br>GTACATAGCAAATGTAATTCAAACTTGCT<br>TAGTGTGTTATAGTTTATGAATTATAGTCag<br>ctattttttttctgaaaatgagaaaGGCTACTTGTGCCTT |

| | |
|---|---|
| | AATGTCTATAGCAAGAATTATAAAGGCTT<br>TGCCTACCTGCCAAGAGTTGacgtattttattaaat<br>tataagtagtagggtttaaaaaaaaacctttcctaGGAATAGA<br>TGAAATCAAATTTTGGAATGAATTTGTAT<br>TCTCAATTATGAGTCTTCATACAGCCAAA<br>GAATACATGACTTTTCAGAATAGGATGTtg<br>tgaattttataaaatgaactgAGTAttgactgaaataaaatttatac<br>gcTTGAAACATGTTTTGGGTGGATGTTGTA<br>GACTCTTTTCAACacgatttttttcttttggtttttaaagaC<br>CTGGATTCTTTTTGATGTTTGTATGATCTT<br>TCTTCAACAGGGCAATCTCCTGTAGTTGG<br>GCTTGATGAAAGTAAAtggtattttatttgtttaataca<br>GCCACAGTCCTTACTGAAGAGTGGGGAT<br>TTTTCCTTTCCTGCTGCCAtttccccccctacccccca<br>ccctccaccctgccacacctcctcccctcccccaaccagcAAA<br>TGCAGGGGCAGCAGCTGTTCAGGTTTTA<br>ATAGATTCTGATAAGACCAATTTATCCTAG<br>CAAACAATTGTTAGCAATAGTTTGAAGCA<br>AGCATGAATATTCAGCACTCCTTTTAGAA<br>TAGGATGGCACAAGACTACTAAATTGTG<br>AGTCAGGACAGTAGTGCTGTGAGCTTCG<br>AGTGGGCAGAGGAAAAGGGGAGCAGAT<br>GACTTCCCTTCTAAGGAAGAAGCAGAAT<br>TCTGGACTGCCTTGTGCAGATGAGTAAC<br>AAGAGGTATGTGGCTGACACTTCTGTTTT<br>CAACAGTGCCTCCTCCAGTAGACATGTA<br>AACAATTCAATTTGTGCATTATAGGTTTTA<br>TTGCTTTTAAAGTGATTTATGTCCTTATCT<br>CTGCATTATTGCCCACAAGATACTGCCCT<br>CTGTCAGAAGGGCTGTGCTATCTAATTTT<br>TTAGACCTTTGTCTTCATTAGTACAGCAA<br>TTTTAGCTTGTGATCATTGTACTAGCCCTC<br>ACTGATAAGCTAAGACACTGCAAGATTC<br>ATGGTACTGACATCTAACCTTGCCACTGG<br>CTTCTTGACCCTTCTACTTCCGAAAcataggt<br>tcatttttttttttttaaacccaaggTAGAACTCCATCTAT<br>TTTTCACACACATTGTGGGCAGTTTAAAG<br>GTTGGTCAGAATCAAATATTGTGCTTTCA<br>AATTTGATAATGTATGAGTACAGAAGGAT<br>GACCTTTGATACATGGTTATACATCAACAT<br>GACATGAGCAGTAAATGCAGCAATTACCT<br>TTACCTCATTGAAATGGTACTTTAGTTTTC<br>ATCTTCAGAGCAATtactgcaaaaacaaaaaaatgcat<br>ttaagttCTCATAACAGTTGCAAAATAAGTAT |

| | |
|---|---|
| | CTTTGGAAACAGTCCTAATTTATGAACGT<br>ATTTTCAATATGAGTTTGGTGTTCATAGG<br>ATGAAATATAGTTCACTTATCAAAAGATC<br>TTGACATGATAATGTTAGGCAGGTTTCTG<br>TAAATCTACTTCgtgttttataaatatgtgaTTTTTCA<br>AAAGGATTAAATGGAAGAGGTGTATACA<br>AGAGGTGTACCATTTGTGTTTTGAAACAT<br>TGAAGTCTGTGCCCGGTGGTCATCTAAA<br>GGAGTATTTAATGGTGATAAGGATCTTTAT<br>GATATGATCATACACAGCTGCATGTAGCT<br>TCTACCACTATTTATGGGATTGTTGCCCCA<br>GTgtgttaatttaaatatttatttctccattaatgAGTGCctcctgt<br>gtgtcaggcactggagATGTAGAATGAGTGTGAC<br>ATGGACCCTACTCTCAGGGAGTTCATTGT<br>CTACTCAGTGGTTAATCTAATAGGTCAGT<br>TCTATCTTAAGTCCCAAGATAAAGAAGCA<br>GTAAGTTGACTTAGTAAGTCCTAAGTGAA<br>GTATTCATAGGACTTAAAAAGTTAGGAGA<br>CCAGGTTTGCATTTTGACTCAGCCAATTT<br>CCAGTAGTATGACCTTGTGGGGAAGTTA<br>GGTAATCTCTCTAAACCTTGGTTTCCTTG<br>ACTATTGAGAAATGATATGTAATGCATGG<br>AactgctgtgaaaattaaataggGAATGTGGACTAGA<br>ATGGGAAACTTCCCTGTCTACTTCCCATC<br>CTTTCTTTATCTCCTCCTACACCTCTCATC<br>CGGTTTACTGTTTCTCTATAAATTCCTCTC<br>TTTCTGGTATAGAGGTTGTTAACAAGGAT<br>CCAGGGAGTCCAAGGGCCTCTGGAAATT<br>TTCTACAggattttatgtgtgtatgtgcacatttTTCTAGG<br>AAAAGAATACATAACTTTCATCTAATTTT<br>CAAAGGGGATCACAAGTCAGTAAAGCAG<br>GAATGCTGCAGCTCTCTTCTAAGAGAATA<br>GCTAAGTACCAAAGATACTAGTTAACATC<br>aatgtttaataatattaaaaacaacaattgAATCTCAAGAT<br>CCTTTTCTTTGGGCATTAAAGAACCCATC<br>AAATTACCCCCATGGCCAGAAGCTATACA<br>TGAGGTACAAAGCATCAGTGCCACAGCA<br>GTGGCAAAATTAGTATAAAAATGGAACT<br>GAAGTTCAGTGTGTGAGTCTTGCATGTTT<br>TTAGATTGCTGATAGCTTTATCAGTGATCA<br>CTTCTAGACATGAGAATTGCTTAAAACTC<br>ATCAGGTTAGTGAGATTGCATTTTGAAAA<br>TAAGATAGGGGTATTGTAAGGAATCACAA<br>GGTATCTTACTGGTTTTGATGTTATAAGTA |

| | |
|---|---|
| | AAGTTTCACCATCCTACCCCAAACACCTA CTTGTTGCCTCCCCTCAACCCAGGAACTT CCTGCTCATGAAATGATGTCATAAAATTA GCTTTTCACCAATACTTGTCTGTCTCCTTC TCTTGGTTTTAATACAGGAAATCTAAGT GCaatgatcaaaattttaaaagctatttcacTGCATTCTAT CACTGTAACACATTAACTGCTTTCTCTTC CTTAGGTTCCCTTTCACTCTTTAGATCTTT TAGCTTATGTATGTGTTTTATAAATTGATA GAAACACAGTTGcattctttattgtttttgtctgtgctggg tatTCACAGCCTTTTCtcgttgcggtgagcaggggcta ctctctagtcgcagtgcactggcttctcattgcagtggccttccttctt gtggagcatgggctctagggcgcacaggcttcagtagttgtggct cctgggctccagagcatagtctcagtaattgtggcacacgagctta gttgctttgtggcatttgggatcttcctggatggaTAAGGGaag gaacctgtgtctcctgcatttgcaggtggattctttaccactgagcca ccagggaagcatcccCCGCCCTGTCCCCGTGCA GTTGTATTCTTAAACCAAACTGTGAGCAA GAGGCTCTCTGTGGTGTTCCACTTGTTCC TGTAGCTTTTCTCACATGAACTGTTATGA AAATCTTGAGTCAATTATTGACCATTCCAT AGCACCTGAAGCCATGGGCATTTGGTCA CTGGGAAGAGGTTTGAACCCTTAAACCT TTACAGGCCATTGAACAGCTGTCAAGTG GTAATGAGTTTTTCCTTTACACTCAAGCA AAAGTAGGCCGAAGGCTTGAATGCATGG TTTTACTCCTTTCCCTATCGTCTCTGCTTA catttctccttcagaatgtacctTAAGTTTGAGTTTGTA CATTTGCTTTGTAGGTCTAAACCTATTGC ATTTTCAAGTGAGTTTCATCCCAGAGTTA ATTATGACAGTGAAAAAAAGTAGATGGT CAAAATGTTCAGGAGTGGGCAAGTTTTT AAATagtgcatttaaatatttttgaagtatttttaataatgaaagaaa atactcaAGATTTGATATTAAGAGAAACATGG ATATAAAGTTACAGGTGTATATACAGTCTG GTCTCATATACAAGTATATGAAAATAAGA CTAAATATAGATCTATCTAtaaatagatacagatata tgaGAGGGAGGGGTTTATACAAATATAACA AATTTTAACTAATCATATTGGGTTATAGTA TGTGTTACtctgtttttcatcttttcccccAGACTTTCC CAAACAATATCAAGAATGTGCATTACTTT TAGAaagtctaatttttaaattaaaaatctagagtaatgAGAT CCAACAAATTACTGTGTTACAGAAAGATT TTTAATTCAAGTTAATAGTTCTGTTATTCC |

<table>
<tr><td></td><td>AAAATTCAGAACATTTTTGTATGTGCAAA<br>TTCTGGGGAAacagttcattttcagaaaaggcagagaaa<br>aaagtgAGTTTATCTGGGCAGTAAAAATATA<br>GAGACCTCTGTTGTTACTTtggaataaagaataga<br>aatgaacgttttataaaaaatatggaGTTAGAAATTAGG<br>AAGGAATCTGagaaacttagatttttttctttcagcagctT<br>GAATAAAGTGCTCTCCACCTATGTAGAAT<br>GGTTTGGAAGCCCTGGTACATCTACAtacttt<br>ctcctccttcctttattTGCACAAAGCCATACACAG<br>CTCATTAATTCCTTTAATTAAGAGGAATG<br>AATCAAAGGTTTTTGTCTTCTAATAAGtagt<br>tgctttatttttatgtaatatgaCACCATAATATTTAATC<br>TTCCCTTAATATATTTCTTTGGAAATCTAA<br>CAAGTTATGGAGACTGCTTCTTATGGATG<br>GGTTCTGATAAACATTGAGAAATAATCAC<br>AAACACAGAATACAGTGATGTGTCTTAGT<br>GCAGATTGCTCTAGAATCGTCATATGATA<br>GCAGTTATAGCAAAATCTGACTCAAGCTT<br>CCCAAATTGAGAATTTGTGGCAATTTGTG<br>AGAGAGATGAGAATGAATTTCATGATTTA<br>GAGAATTAggtaaattataatatatttagagATTTgtagaa<br>atatttactttttttgggTAGTTATTTTAAATGTCCATT<br>TATGTACAGCAGTGTACCTGGAGGCCCCT<br>CTGATGTGCTGGTTTTCTACTAGGGAAAC<br>TGTAATTGACTGAAAGTTTAATATATACCC<br>AACCTGCTATAAGACATCTTATATATACAG<br>TGTAATTTGATCCTTAGAAGAATTCtttgaaat<br>agaaattatttatcCTCATTCtttagataaggaaattgaggttc<br>CTGGAAATTCAGTAATGCatcaaaggtcacacagc<br>tgataagtgGGAAAcacaggatttgaacctaggttGATCC<br>ATAAAtgaaccttccctggtggctcataggctaaagagtttgcc<br>tgcagtgtggaagaccggggttcgatccctgggtcaggaagggc<br>tcctggataaggaaatggcaacccactccagtatccttgtctggaa<br>aattccgtggacagaggagcctggcaggctatagtccatgcggtc<br>acaaagagtcagacatgactgagtgacttcacttgatCCACA<br>AATGTTTCACTATATTGCACTACTTCCAGT<br>CTTACCTATTTGTTCAAATTTACTTTTTAC<br>TAGCA</td></tr>
<tr><td>AC_000171.1:38926837|38986125</td><td>TAGACTTCTATGGCATCTCAGAAAGACAG<br>TGGTTCAAGACACCGATCATGCCAACCC<br>CCACCACAGGGCAACGGGCTTCTGTGAT<br>TGCCTTTGGAGGCGTGGTCCTTGGGAATT<br>TGGAGGACAAAAATGGGCTTTTTCAGGC<br>CAAGAATCTCAGATCCTTCTACTCTAGCA</td></tr>
</table>

| | |
|---|---|
| | GAGAGGAACTCACAGGAGAATGAACCTC TAGAAATAAAACCTGCGTAGCTGAGAGT CCTTTTGAgagacacgtccgaagagtcgaGGTGCA GTGGTGAACAAACCACCCGTTAATGCAA GAGGCTCGGGAGATGggtcaagttcgggacccagtc cttctaggggacttcctccggtaccgttgggtgaggtcataagaac ggacctcttagggtacctgtctcctcggaccacctgatatcgagca tcccagagtttcttagtctgtgtagaGAGGGGGACAAAT TCTAACCCAACCCAGAACCAAACCTCTG AAAGCTGACCATGACCCGATCTCAAGCC CCGAGGTGAGAGAGCCCATCTCTGTGctat agaaaaagaaggagactttaaacCAAATAAAACAGC CTTTTAAGATCTGTTGCCTCATTAAAGAT GGTGGAAACTCATCCAGAAACCACCTCT GCAGCTGAACCCATGGCCCTGACTCCTC TGTGTCCAGGTCCACATGTCCTGTGCTCA CCATCAGCCCAGTTGGAAGAGGATCGTT TTCTCCCTTCACTTGGCCACCCTTGTTGG GCAGGCAGGGCCTGATTTGGCCTGGGAG CTGTCAGCACATGCCTCTGAGATGGTAGA GAAACACAAACTCTTCAGAGAAAAGTGC TCCGTAGGAGCGTTGCCATTTATGGAGTG ACTAATCTTCTCAGTTTGCCCAGGACAGG AGTCTCCCGGGATGTGGGACTTTTAGTAC TAAAACTAGAAACACCAAGCAAACCTGG ACAGCTGGTCCCCTGCACTGCCAGTGTC CATCCTggtcctcgtctctctcctttaCACAAAGGTG ATATGTCTCCTAATGCCAGAGAAGAAGA ATCATACGAGAAGGGTCTAGGTTATTCTC ACCGTGGTTCTCTGCCGCCTCTTGGAGG GTGCACCTCCCCAGGATTGCCTGCTTCGT AATCTCACACAGAGGCAGTTTGGAGGCT TGGCAGCCCAAGGGTGTTGTAAGATCCA CAAGAGCTGGTCAACAGATTTCTTGCTG CCTGAGGCCATCTGAGCATCTTTATAGGA TTCTGAGTCAGAAACCTAGGTTTCGTCA AGGTAGGGCCTTTGCTAATTCTGGAAAA GACACACAGATTCTCTGGAGGGGAGACA TTTGTTTTTGTCAGGAGATGGTGTGTCCT ACCAGGAAAGAATGCACAATACAGGAAG CACAGGCTGTGTCACGTGGGAGCCCCGA GCCATGTCTGCACATTCGAAGCATCTTAT TCATGGCCCAGGCCACTGAGGATGGAAC GCCAGCATCCCAGGTGTTCTCTTTTAACT |

| | |
|---|---|
| | ACCTGCCCCAGTCCTGCTTCACCCACCTC<br>CAAAACCATGGACTACACAGAGTGCTCA<br>AAGTCACAAACAGGGGCATTTAAGTAGT<br>AATGACAGTACTTAggaccaattcttggacggacggtt<br>acgttccctatttcaaagttaggaaccagacccttctaaggtgtacg<br>acatcccgttacttcggacacacggtgttgatgacttaggccttgtg<br>gatctcggacgcgagacgttgttctcttcggtgacgttactcttcgg<br>acgtgtggtgtggaCAGCATAGCCCggcaagtggtgttg<br>atctctttcgggtacatcgttgcttctgggtcgcgtcggtttttagttat<br>ttgtttctAGCAATGACAGTGAGATTTCCTGCT<br>ATGTCCAGTTgaaccccctcttccgttaccgttgggtgag<br>gtcatgagaacggacctcttagggtacctacctcctcggaccatcc<br>gacaccaggtgccccagtccttcttagactgtactgactctctgaag<br>cgaaagtgaaaagtgaaagtacgtaacctcttcctttaccgttgggt<br>gaggtcacaagaacggacctcttagggtccctgtcccctcggacc<br>acccgacggctgataccccagcgtgtctcagtctgtgttgacttcg<br>ctgaatcgtcgtcgtcgtcgtcgtcgtcaagaaccGAAGCTT<br>ATCCACCTACCTGGCAAACTCAAAAGTT<br>GGAACATCACTAGGGCAGTGCTGAAATG<br>ACATACTCGACTTTCTGCAACCAGAAATT<br>CTAAGGAACATGAAAAATCTGCAGTCCT<br>CTCTGGATTTTGAGAGTTTTGTTTGGGGA<br>CTAAAATTACTGGTCACCCAACTCACTGA<br>GggggtcctctccttcctcctcttagTTTCAAAACCTGA<br>AGTTCTGGGACCTGATGGTCAATGATCTG<br>ATCCCCAAACATTCTGCAGCCTGATTTTA<br>TGCTAGTCTTCTCTCCAGGTGTTCTAGGC<br>CAGGTCAACTTATCTTCTCTTTGTCTTCTG<br>AACAGGCCTGGCCCTGTCCTGCCCCCAA<br>ATTTTCCTCACCACCTCCTGTCCAACTCG<br>TggcggggagggggaagggaggagtgggaggagagaGG<br>GGGCAGCTCAAGCCCGCCTCCTGCATGA<br>AGCTCTCCAAGATTCTCCAACCTCTGTCT<br>GCCCAAACTCATGACAGAGGCTGCCTTA<br>CCTTTTCTCCTTAGCAGATGGTTAGCCCC<br>AGGTCCCACACTTTcctaaaacaagaacaaacaaagc<br>aaaacaatgtaaagaaataaaaccgacgtgacctagaagtaacgc<br>cacgtgtccgagtcttcactcgaccgagtcatgaacgtcgaaGC<br>CGTGGTCCTTATTTGatcatacacacaatcaacgagtc<br>agtacgttggggtacctgacattggtcggtcctaCTTCCATC<br>Atatcctaaaaggttcattcttatgacctcacccaacggtaaagaa<br>agaggtcctctagaaggatttcgtccctaacttgagcccagaggac<br>gtagtctccatctgagaaataacagactcggtggtcccttcggggg<br>tatcaacgtcatacaccctagaatcaagggactggttcctgacttga |

| | |
|---|---|
| | ccACCCCCTGTGTTGActcgaattggtgacctagtggt<br>ccttcaggggggACACGTTGTCATTTAAGTGGCC<br>ACGTGTGCATGCCTGTCCCCCTCAAGAA<br>ACTGTAAGCTCTCTGAAGGCAAGGTGTG<br>TGTGTCACTTTTATGGACACATCCAGCCA<br>TATCTGACCCAGAGGAAGAACTCAGATG<br>TGATTGCTGATATCATTGGATCAAGTGGA<br>AACCCCAGTTAAAACCCAGTAACATAAG<br>CATCTGCTTTTTGACCCAGACATaggtgggttc<br>tctttacttgtaCCAAATGCCTGTCCAAGAATGT<br>TCACAACAAATCTATTCTTAACTGCCCCA<br>AACTGTAAACAACTTATCAATAGCAAATT<br>AGATAAACATACAAAGGATCATAGAAATT<br>CATCATTTGAATGAATCTCCAATGgtaatatga<br>ctcacttcggTTGCACAAAATAGTCCATACTAA<br>ATGACTagtaaaaccgaagggaccaccaagtcaccaatttttt<br>tttagatggacggtcacgtcctctgtgtccaagctagggacccaac<br>ccttctaggggatctcttcctttaccgttgggtaaggtcataagaac<br>gaacctcttagggttcctgtctcctcggaccgcccgaggtcagtta<br>ccccagtgttttctcgacctgtgttgaatcactgatttgttgtcgttggt<br>gtttTGACTTTATTTATGTAAAGCTTCAAAA<br>CAAGCAAACTTGTCTCATCTATGATGAAA<br>ACATTCAGAGCAGTGCTTGCTCCTGGGG<br>GACAGGATGGGAGATCAACAGGGAAAG<br>GGGCACGAGGGAAGTCTCTGAGGTGATA<br>ATAATGTTCTGGGTACATGTATTTGTTAGA<br>CCTCAAAACTAAATACTTAAGATTTGTTC<br>ATTGCATGGAAGATAAATGCTTATCTCAA<br>AAAACTTTttgtttataacttataataattTGTGCATGT<br>TGAAGAGGGGAAATATACTGATGGGCAC<br>AATTTACTTGCAAATACATCAGAACAGAA<br>AGTGTATTGATGGATGGAGAGAGGcgaccta<br>cctatctatttaccTGAAACAAGCATAGTAAAAA<br>AAAGCGTCACATAACAGCTGGTGCTGGT<br>CACCACCCAAACCATCGCCTTTAGTGCTG<br>CCTCTGCCAAAGAAGAGGAGTTGCAAGG<br>AGAAACACCTCCTCTGACTGGGCTTTAG<br>GGAGGAGGTGGGGTTGGGGGCCAGCAG<br>GTTCCTAGGGAGCCAGGAGAGAGGCAG<br>AAGGGAGGAGTGAAGCTGGAGTTTCAA<br>GGGTGgtcccttccgtctccctccttccttaaTGCTGGAG<br>CATCATGGCTCAAGGATCTGATCTTCAGC<br>TGCTGTCCAAGGGCTGCACCCTCCTTGG<br>CACATGCCAAGTACAGAGAAATGTTAAG |

| | |
|---|---|
| | CAGATGGCATGCTTCTGGGCTCGCCCCAT<br>CACTGGGCCTACCGCCCTTGAAATCCAG<br>GGTCGAATTCATGGTTCCGAGGACTTTTC<br>TTAGGAAACACTGATAATCCAAACCACG<br>TGTAACCTCACTCCTCCCTTTGATAGGGT<br>GGCTGGGCTGGTAGATCAGAGTGGAGCC<br>ATGGATTCAGTCCATCTAGATTTCAGCAG<br>AGGTTGCACACTGGCAGCCCTCCGGCTG<br>GACCCAGCCCACAGATGTGCTGTGTGTT<br>TTGTTTGGCCTggggatccaaaaaaaaaaaaaaaaaa<br>aaaaaaaaatcaaatcaacggttctaaaaaatttagtgtctccATC<br>TGGATTGTCTGCATCTCTTGAAATGTTGG<br>AAGATTCAGCAGCCAGGAGCCTGCATGA<br>TGACAGTTGGCAGGAAGAAGTGGCAGC<br>AGCTTTCTCAGAGGCCTGACCCTCTTTCA<br>CTCCTGTCCCACCAAGTCCACCCTATGGG<br>CTGGGGCATACCAACCTGTGGGTCTTACG<br>TCACTTGTGTGGACAAGTGAGAAGTCAG<br>TGTTGGCTGATAGTAACACTAAAGTAACC<br>TTAATGTGATAAATATTGACTGAGCAATTA<br>ACTACCCAGAGTGTTTGGTCCTGGGAAC<br>ACAGTGGAGAACAACTTGGTCCCTTTTC<br>TAAAGGAAGTTACATCCTAGAGGAATActa<br>tctccctctctctcttctttccttccttctctcatcACAGAGgatcc<br>ctttccttccttccttccttctcttcctccttcctttgtcTCATTACA<br>AGCTGTGGTAACTATTCTAAAGGGCCTAG<br>AGGAAAGAGAAGATACTATACAGCAGAG<br>TTTGGGGCCAGGAAGCTGGCCCTCGACA<br>AGCCGATGGGGAAGATCTGTCTGGccactgt<br>aaagaaaaaaacccccccccactgATTTTTAAGTGAA<br>GCTCTGAAGGACAAGGAGATCTGGGAAA<br>GATTGCTGGACGAAAGGCCATAAATGAG<br>GAAGTGTTTGGGTTCTTTCAAGAGCTGA<br>GAGAAGTCTACTGGCAAGAGTGAGAGAT<br>AAGGGAGCAGGATAAAGGTGGGGAGAT<br>GAGCAGTGGATGGGACTTGTAAACCAAG<br>TGAAGTGCTTAAATTTCATTCTGAGTGCA<br>GTAGTAGCCATCGAAGGTTTCGGGTCAG<br>AGAGAGACACAATCAGATTTAGGTTTCAT<br>AAAACCAATCTTGTTATTAGTAGGGAATA<br>TAGCCAAAGGTCTCATTGCCAGTGGCAA<br>GTAAGACATGATGGTGTGTTGcaattacctccttt<br>tttttatttagggtcccgtTTCTGGAGGAAGTAATTT<br>CATAGCTGGTTGCAGAAGGAGGTAAACC |

| | |
|---|---|
| | TGGAAATTCTATCCACTGGCTGCATCAAC<br>ATGCAAATGGAGGGATTTAGACAAAGAT<br>TGACTAAGATGATACTGTTCAATCTTGGC<br>TTCTTCCAAGATACAAGACATTTTCACAT<br>AAATTATTACTTCCTTTGGTAGCAGAATG<br>TAATCTAAACCAGTAtcgtcaagtaccgtttatctttga<br>GCCTGAAAATTCACATACATTTTGCATTC<br>TtcagatattaaaaaatttcgatatttacACATAGatagttgttgt<br>tttgtttgttatttttgtggCAAATATTCCTCCCACTGA<br>CCCTGGTTAATGCCCTTAACATTGGgacgtgt<br>aaccttagtggacccttcgaaatttcAAATGCTGTTTCC<br>TGGGTTCCCCTCCCAGACATTCTGACTTA<br>GTTGTTCTGGGATATATTCTGGACATCTG<br>GATTTTTAAAGCTTCCTGGTGACTCTAAT<br>GTGCATGTGTGGTACAGAACTGCTTGTTA<br>ATCCAAGGCAGAGGTTCCCAAAGCAGCA<br>AGGGCATGATGCAGAAATGTGCAAGAAA<br>TGCAATTTTCTCAGGCTCTACACCAGACT<br>TACTGAACCCAAttaagacccccccacccagagAGC<br>AAGATGCGTTGTAATCAGTCCTCTGGGTG<br>TTTTACGAGCACCCTGAAGTTTTAGAATA<br>TCCCTTTTGATTATGCaaagatctagactatctgtccc<br>acggactacttgatacctacctccaagtactgtaacatgtcctctgtc<br>cttagttctggtaggggttcttcttctttacgtttttttgttttaccgacag<br>actcctccggaatgtttatcgactcttttcttcccttcaatttcatttcct<br>cttttcatttctatatgggtaaacttacgtctcaaggtttcttatcgttcc<br>tctctattctttcggaaggagtcactagttacgtttctttatctccttttgt<br>tatcttaccctttctgatctctagagaagttcttttaatctttatggttccc<br>ttgtaaagtacgtttctacccgagctatttcctgtctttaccatacctg<br>gattgtcttcgtcttccacaattcttctctaccgttcttatgtgtcttcttg<br>acatgtttttttctagaagtgttgggtctattagtactaccacactagtg<br>agtagatctcggtctgtatgaccttacactttagttcacccggaatcc<br>ttcgtagtaatgcttgtttcgatcacctccactaccttaaggtcaactc<br>gataaagtttaggactttctattacgacactttcacgacgtgagttata<br>cggtcgtttaaaccttttgagtcgtcaccggtgtcctgaccttttttcag<br>tcaaaagtaagattggggtttctttctgttacggtttcttacgagtttga<br>tggtgtgttaacgtgagtaaagtgtgcgatcatttcattacgagtttta<br>agaggttcggtccgaagtcgttatacacttggcacttgaaggacta<br>caagttagaccaaaatcttttccgtctccttggtctctagtttaacggt<br>tgtaggcgacctagtagtttttttcgttctctcaaggtctttttgtagata<br>aagccgaaataactgatacggtttcggaaactgacacacctagtgt<br>tatttgacaccttttaagactttctctacccttatggtctggtggactag<br>acggagaactctttagacatacgtccagtcctttgctgtcaatcttga<br>cctgtaccttgttgtctgaccaaggtttatccttttcctcatgtagttct |

| | |
|---|---|
| | gacatataacagtggggtgaataaattgaagatacgtctcatgtagt<br>actctttacgacccgaccttcttcgtgttcgaccttagttctaacgga<br>cctctttatagttattggagtctatacgtctactggggtgggaatattt<br>ctcggagaactacttgcactttctcctctcactttttcaaccgaatttc<br>gagttgtaagtcttttgcttctagtaccgtaggccagggtagtgaag<br>taccctttatctacccctttgtcacctttgtcacagtctaaaataaaaa<br>gacccgaggttttagtgacgtctaccactaacgtcggtactttaattt<br>tctgcgaatgaggaaccttcctttcNNNNNNNNNNNNN<br>NNNNNNNNNNNNNNNNNNNNNNNNNN<br>NNNNNNNNNNNNNNNNNNNNNNNNNN<br>NNNNNNNNNNNNNNNNNNNNNNNNNN<br>NNNNNNNNNcaaagaagagaaaaatatagCTGATG<br>AATCTAGCTAGGGGTCTGTCAATTGTTTT<br>TATCATATTCTAACAACAAGATTcactaaagtaa<br>ctaaaaaaagggacaataagacaaaagtcaaaataactaaagac<br>aagaaataaaataggaaggaaataaacgaaacccaaattaaaag<br>acaagaaaaagattaaaacattGCCAGAAGCTTAGG<br>CTGTGGATCTGAGAATTTAGactatatataaagag<br>aaattcTACTGGTTTAGCCCTGGCCCACAAA<br>TTTTGAGAAGTTGAGTTTTTACTTCATTT<br>CATCAAAATATGTTCTAATTTCCCTTTGGT<br>TTCTTCATTCAAGTggatataaagaagactaaaacaa<br>TACTTCTTTTATCAAGTGCTAATGTCACC<br>AACTATAAAaaacttaaactgatgaaatttTTTTATCG<br>GTTTTGTTTTATATACTTTGAAGCTATcaaca<br>atatatgtatatgtaaatcctaacaatatagaaaaactaGACTTT<br>ACAACTTTATTATTATGTGATTTTCTTTCC<br>ATCCCTGGTAATATTCCTTGTTCTAAATTC<br>TTTTTGATATTAATAGagtgaagtcaaaataaaaataa<br>tcacaaaGCATGGGGTATTTTCCCCggtggtaaaat<br>aaaaatcgCTATCAGTGTCTTTATGaaatttaactaaa<br>gaaaatctttcgATATAATTGtcagaacgaaaatttttatggg<br>aTTGACAATCTCTACCTTTAGTTGGGAGT<br>GATCAGACCATTTGTACTTATTGTAGCTTT<br>TGGTATGGTTGGATTTAAACcaaagtaaaaagatt<br>aacaaaagataaataaggtaaataagaaacaagtgaaaaaagag<br>aaactggTCTCATGTAGTCGTGCATTTTACTT<br>ACCTTTATATGTATAAAACCCATAATTCAT<br>TGTTACAAATTTCGCTTTATATAGTTAATT<br>TTCTTTCTATGTGGTTAGAATCACAGAAA<br>ATACATTTACCgagtaaaaatggtaaaagttTACTGT<br>TCATTTGTTTAGATCCAGATTTCTTTCTGG<br>TATTATACTCCTGTTTGTAGAACTTCCTTT<br>AATACTACTTGTATTAGCAGGTCTGCTGG |

| | |
|---|---|
| | CAGTGATTTCTGTCAACTTTTGTTTGAactc<br>tttaaataaaacataagtaaaatctAGTTACTTTTGTGT<br>GGGTATAGGATTgactcaaaaagacaaagaaagtcgtg<br>aaagtcAGATCTCATTCAATTGTCTTATGACT<br>TGCATAGTCTTGTATGAAGTCTGCTGTGA<br>TTATCTTCTTTTTCATCTTGACATGCTTTG<br>TTCCTCCCTCTAAAGGCCTTCAGAAATGT<br>CCTTTTTTTATTAGTTCTTAGCAGTTTAAC<br>TGTGATGTATCTAAAaaactaagaaaggaaagaaag<br>aggagaagaagggagaggaggaaggaaaagaaaaaagggac<br>agagaagtaaaagtaggagaaggaggaaggaaagaagaggaa<br>aaacataaaaaaagacgaattacttatacttatataacctagacact<br>aaacaacaaaaaaaattaaaaacagtttaaCTCAGCTGTT<br>ATCTaagaaaagtctataaagaagaaggaacaaaagagagag<br>gaactGAGTTTACtcatatatattgacttttacgaccccttttac<br>tttacaattacAATTCTAAAAGACTGTGTGAGG<br>GACAGCACAGCAGTTTAGAAAAATTAGA<br>CCATTAGTATTGTCTTATGGATCCAcctaaaag<br>acaaggaaaaataaaaagtgaggcaaaaaaaacacacacacag<br>agtcaaaGAGTAATTTCTATTAATTTATCTGTA<br>ATATTACTGACTTATTTCTTCAGGTGTGTC<br>CATCTGTTAATAATGTTATCAATATTAATTT<br>TCATCTGTGGTATTTTCCCCCTAGGATTTC<br>CATATGCTTTTCTTATAtccaaagacaaaaagattaG<br>AAATTCTCCAAATATTCATGCATGTTGTCT<br>GCCTTTTTCATTAGATCTTTTATCCTGTTA<br>ATCATAGATATTATAAAACTTATTTCTTAG<br>AGTTCTAGCGCTTGGGTCATCTCTGGGCT<br>CTGTTTTtaactgaacaaaagaaaaattaaaagtaaataaata<br>aaaattaacctcctattaacgaaatgttacaacataaacaaatacga<br>tatattgttgtacttagtcgatattcatatgcatatagaggggggtg<br>gtggtgggaactGCCTCCCTTTTATTGACTTCTT<br>TATCAaaactgtataaataaaaacgttaaaacGGTGTCT<br>TATAATGTTTGAATGAACACTGGACATTG<br>TGTATTGTGAAACAGTAAAGGCAGAGTT<br>AAGTAGTACTTATGTTAAGAAACAGGCAT<br>GTCTCCCTTTCTGTCAGACTGTTAACATG<br>GAGGGTGACATCAATCCAGTTGGTAATTA<br>AGCTCATTTTGGGTGTTTTGTTTCCACAG<br>TCACTATTAACTTCAGGTTCCTTTAGCTTT<br>GGTTTGCCAGTGTCTTTGTTTTGTGTGGG<br>GTGAGGTTTGGGGAAGATTTTCCTCAATA<br>TTTCTGCTCCATCCTCTGGTTTTATCAGTC<br>CTCTCTTTGCTATGCCATGAGGTGAGGGA |

| | |
|---|---|
| | TATCTCTATGCTCCATAGTCAATTGCTGTT GTTTGTTATTTGGTTGTAGGATTGTGTTG GGTATAGAAGCATAGCTCTTGATTTTCTT GTACAGCTTCATTCTCAAACTCTGTGTGC CTGAGTCTCAGAAGTGGGTTTTTCCCTAA TGTTTCTACCTCCGTCTCCATGATAGCCA AATTTTACTTTTGAACTGGAGGTTGTCTT AGGTGCAGTGGAGTTTTCCAGCCCCTTC CCCAGCATTTTCAGAAATCTGCTTAATATT GGTGCAGGGATTTGCTCTCAAGGTTTTCt ggaggaagaaaaaaaggatccAGCAGGTAACTTTT GCTTCTAGTCTTTTTGCAGAAACAGTGAG TCCTTGCCTGAGCTTTTGGAGCAACAAA ATTTCTTGCCTTATCTCAGAGGCAGACAcc taaacaaagaaggaaaagagaacatGCTTGTGCTGTT TGCTTTGTGTAAAAGAAGAGTgatcactcacttc agcgagtcagcatagactgagaaatgttggggtacctgacatcgg atgtggtgaagaggtaggtaccctaaaaggttcgttctcatgacctt acccaacggtaaaggaagaggtctcctcacTGCAGATTT CTGAGCTGTTCTCTCTGCTTCACACCTGC ACCACTGAGTGAGGCTCTTTGGTTCTCTG CTGTATCTCCAGTTGTCCTGACTTCCCAA AGGAAGCCCATAGAAAAGAACTTCTCTC TGGAGTTCCTAATTTTTCTAAACTGCTGT GCTGACCCTCACACAGTCTTTTAGAATTC ATTAACATTTCATCTGCTTTCTTCTTACCA ATATTTTTGATtaagtagagaaaaaagaaacggtATGT TGAAATATTTTGTGAGCTGAACCTCGCCT CAGAGGGCTTTGTCACTCTCTAGAATTCA TGTCATCTGTTTGCTTTACAATCTCAGATC TCTGATGGGTTTGAGAAAAGTTATGATTT CTTACATTGTCTGAGTCATTGTTATATTTC TACATTCTAAGGAAGAGCAGAAgtgagaaatg aaaaatttttttatgtgacaagaacgtagaacaaaaaaaaaaaagtta atgtacGGGTTTCGGTTACAACTTAGAGCTT GTTCCGCATCAAGATGTAACTGGCATGTG CTTCATTCTTTGTTATATTTGATTATTATAT ATTGTTCAAAGTAATACTCATATATGTATA ACAAATCAAGACTTAGTCAAGATTAATGA TATAAAGACTCAAGATTTATAATAAACCA TGCTGTCCTGCCCTGGGACTGCCCATTTC TGTTACCTTTTTTATAGAGGCAACCACTT ACTACCTGTTTTAGCTGTTCTGTTATTTTG TACTGTTAATAACTTACACTcgtcaaagaactaaa |

| | |
|---|---|
| | aaaaagaaaaatctacaaATCTGGGTTCCTGCTTT<br>GAAAACCAAAGAGTTAGTTTTCTTACTC<br>AAgtacatatcaaaagacacagagTCTAACTTATCCC<br>TGTTACCCTCTGCCAAGTAAACGCTTCAT<br>GTACTGaaatcaaagaaacaaaaaagaaacaacctcTTC<br>TTTATCTTCCTCTTCTGTGTTGGATTATTT<br>GTTTGATAGATCTGCTGAAGACCCAGTGT<br>TTTGGTTCTTCTCTTCACGATTTCCCTGTT<br>AATTCCTTTTGCCTCTCTCTTGTTGAGTT<br>GCCTGCTTTCTGGACAGTTTCCTTcaagaatt<br>aaataaaagaacacaacATGGAGTACACACTTTCA<br>TCGTTTCCTGATAGAGAATGCATGATTAA<br>TATAGTTGTTTGAGACCTTGTAGTTCTGA<br>TATATCTTTCTATTTCCCCCGCACTTAATT<br>GGTAGTTTTTCTGAGAGATAATTCTActatcc<br>tttataaaaaaggtgAGTTTTGAAGGgtaacatggttaca<br>aaagatCCTCTCAGCATTACTGTTGAAATTC<br>CAAAGTCTTCAGATTCTTTAGCCAATTTA<br>TGAAACTTTTACATTTCCTCTCCCTCGTG<br>ATGCTTTTCTCACATCCTTTCTCGGAAGT<br>TCTGAAATTTCTTGAGCGACTGACTGGAT<br>TTGTGTATTCTTTGATAAATTGTTTTTGGG<br>TGTTTGGGTTTTAATCCGGAAATTCGTGT<br>ACTTTAAAAGTGGtctttgaaaatataaaaaagagacta<br>ctaaagaaaggaaggtaaaaacgTGTTGGTTCTTTCT<br>AGGATACCTGTTAGTCTAAAGAAactaaaaaa<br>cgaagaaagaaagaaggaaacaaagaaaagacaaagagaaaa<br>acaagacaaatgaGGAGATTTCTTCAGTTTTGT<br>CTTCCAGaggaagaaactttataaaaggaaaccgatgacat<br>aaaaaataaagggtctGCTATTCTTCATTCTCAGA<br>ATATTCCTTTTTATAACCTCTTGTTCTTGT<br>TTAATGGGTATAATACTTATGTCTCTCTGA<br>ctcgaaaaaaataaaattactaaaaactcGTTTTATTCTG<br>CTCCCTGTGTTATGCTCACCATTCCTTGTG<br>GATTGGGAGACTGATTATTGTCCCGGTGA<br>TGAAGGGAGCTGGGGATCTTACCTTCCA<br>GGATGTACCTTTGGAACCCATCCCTCTTT<br>TCAGTGTGGCGTATAGACCCATCCCACCC<br>TCAGCTGTACCTGGTGTCCCTTTGGAACC<br>CATCCCTCTTTTCAGTGTGGTGTATAGAC<br>CCACCCCACCCTCAGCTGTACCTGGTGTC<br>CAGAGCCTCTTTGTTTTAGGCCTTTCTGA<br>AGAGGCAACATTTTAGGAAATTTGTgagga<br>accccagtgtcttcaGACCTGAAAGTTTCATAAC |

| | |
|---|---|
| | ATTTGAAAGACTTCCAAACAATCTTCCTA<br>TTTTCATACTCTTCCAGGATTCATCTTTAG<br>AAGTACCAGGTGTGCTTCTGATTCCTGAG<br>GCTTTCTGGGGTCCTGAAGCACAAATCA<br>GTTTACTCTTTGTAAGCTCTTTGTTAGCTT<br>CTAGCTCTGCAGGTGAGGTTTCAGCCGG<br>CCCACACCTGCTAAGTTAGTTATAATTAAT<br>CTACTCTCCATGTTACAAATTTactgaaaagaga<br>agagaactaaCCTTTCATATTCAGGGGAGGCTt<br>ccccgtctcccttcctctctctcaaaATGGATTATTGGAT<br>TATCAATTTAGAGTTcaaacccctctctcttctctaatC<br>TCTTAATGTTTACTCTGTCATGTTAAAGC<br>AGAAACCTTAGAAAAGCATTTTCATTAGA<br>GTGTTGAGATAGAAATCAGATAACAGCA<br>GGCTTGGGATTCAGTTGATGAAAAATCA<br>GCCTTAGccttattttgttataaatttattgaaaccTTATTtca<br>ccttcctctcttttcctccttacAAGTGCTAAATATATTA<br>TTACCAACTTCAACTGAGCCCTCAAATGC<br>CTGAGGGTCTGTATTCTTCTAATCATTGA<br>GCTCTTTCTTTCCTAGACTTTTTGCTTACT<br>ATGTACACTTTCCTTAGATTTtattggagggagg<br>gagggcgtcCGCCCCCCTCTCTCTATGATCCT<br>GTCTTGTGCTTCACAGAGAACATGTGTGT<br>GGGAGTCTCGTCAGGCCTTCCCATCCATG<br>TGTATCTGCATTTACACACGTTACCTTCTC<br>TTCCACCCTTTCACAGTTGCAAGCTATTT<br>GCTAGAAATGTAGTaacacaaacctcttccgtcaccgt<br>gggatgaggtcatgaggacggaccttttagggtgcctgcccttcg<br>gaccacccggcgtcaggtaccccagtgattctcagcctgtactga<br>ctcgttgaagtgaaagtacgtaacctcttcctttaccgttgggtgag<br>gtcacaagaacggacctcttagggtccctgtcgtctcggaccacc<br>cgacggcagataccccagtgtctcagcctgtgttgacttcgctaaa<br>tcgtcgtcgtcgtcgtcgtattatattAATACCGtaatatttttatt<br>catacaCTCCCCCACCCCGAAGTACTTTATAT<br>CAATAATACCAGCTCCACAAAAAGAGAA<br>CATAACCAGTTAATGTCACTTATAATTTGA<br>TTAAAATACTACTTTAGAGTCTGAGCTgtct<br>cacataaaaaaaaaaattaattttaaaaattaaaaaaatttaaaataaa<br>aatttgaaatgttataacataatcaaaacggtctatagctttacttagg<br>cggtgtccatatgtgcacaaggggtaggacttgggaggagggag<br>gagtctcTGTATTTTACACTAAATGTTGACCA<br>AATCATCTGGCAAAAGCCAGGATATTCCA<br>CATTTTGCTTCAAGTTAGGGaaatcaaaagtcaa<br>aaaacatCCTGTCATTAGCAGTATTCAGTGAC |

| | |
|---|---|
| | CCTTATCATGACGTTTCCGGAACAGACAT AATAATATTTCTATCATTAATTTAACTTCA GAAGTGATAAAAAATCTCTTTTAGTTACT TTAGCACTTGCGATGATTATTGTTTACTGa aacttttgtttaataaatgaTCTTCACCACCTTGATAG TGCCCAgcaatatgtattatatatatgatttataataGCTAA ATACAGGGCTTTCTTGATGtacatttaatatttaaatt tatctatAAATCAGGAGGCATAAGAAACAGA TATGAATGTAGAAAAGGGATAGATGTTAT AGTTCTTGTTAGTAATTCTTGTGTCCGTG GTCCTGGGGTCAGCAGTGGAGAGGATGA ACAGAATAACCTGTACCCTTTTTTTATTAA TTAcctaattgaaaaaaaaacttaaatgtaaagaaaggacAAT TTAAACAATTGGTTTAATTTTTCAGACAG GGGAAAACAAAGGATGGAGTGGTCCAA AGGCTGGGTTTCCTGAACCAACAAATAA CAGTAAGTTTCTACCTTTATATTAGCTACA CTTGGTGTTCGTCAGTGGTTCCATGACAT GTCCCTCACTGGGAATTTACTGGGTCTGA GATGCTATGCTAAGTGTTTTGAAGGATGC AGGAAGTTGTGGTGTTTGCTTTCCTGCA ACTGACAGAAGACACAGCAACTTATATC TTTAACATGGTTAAGTCATTGCAGACAGA AAACCATTTTATCCATAAAGTGAactacaacaa aaaaaatttcaacaaaaataacTGACATTATCAgtcatcttt ttttagttctttctctttgtcaTATCTACCAGTCCAATGT AACCCAGCATTAACTGTATTCACAGTTCT ACTTTTTTAGGTACACACACAGATACTTT AACATAATTAGAATATAGACAAAAATTTG TAGTGTGTTTTCATTTAACACTGTAGTAC ACATTTTTTAGGTTGTGCTGAGTCATTAG AATTTTTGGTATTTTATTAAAGTTACTAAT TGAAATATCAGAAATGTAAGGATAAAATT GCTCACAATTTTATTCCAAATAATAGTATA TCATCATCTAAAAATCCACTTCATTTCTGA AGTGGGAGAAGGGAGCTATGGTGATAGC TCCCTTCCTGGAGTGAATCCTCTAACTAA ATACTTCTAAGTAGTTAATGGGAAGATGA AAGGGTCTTCCTGAGTCTTTGGGtggaactaa caaaagtcgagttttattagatgATATCACAGAGAAAC ATTTTAGGATGGCAGGCAAATTTTGCTCA CCTAAGATAATTCCCAGTGTTTGGAACAT CCTCCTGGTTGCTAGATAGTATGCTGCCT GATTCATGAATCATCGAATAAAGCCAATT |

| | |
|---|---|
| | CGaagaagtttttgtaaaatttcttatttttatttttaggtgaagtccat<br>aaagacaaaagtttccTACATGATCAAGCTGTTAC<br>ACATTGAAAGTGTAGACATTTTTAACTTC<br>CTCCCCGCCTCCCCAAAAGCATCTGTTTT<br>CACTGAAGTGACCATTTAAgacatttagttattttac<br>tttgtcgCAGCCAGTGAAGACCCTGACAAAG<br>TGAAGTCCCCAGTAAAGTTGGATCTCTCT<br>CATCTCTAACCCCAGTTCTCTTCCTGATA<br>ACCTTCAGAAGCAGAACTAGCTTAAATA<br>CGTAAATTGTTGTCACGAAGAGTATATTC<br>TTTCATATTAGTAGCTCATCTTTTGATGTA<br>TAATGAAATTTGTTACAATTCTTATCAGA<br>GCCTGCTTATGAGAATTGAATTCTTGGAT<br>TATCAAAACTAGCCTCTTGAAAAAACTGT<br>TTCAGTGAGGCAGTGGTtaagtacttaataaaagac<br>cGAATAATTTCAATAATTCAGTATGAGTCT<br>GGGCCTTTTGTCTGTGTATGTAAGGATGT<br>TGAATGTGATAGGCTCAGCACATTGCCCG<br>AAGGAAAAATGGAATCAGTTCAGGATTC<br>TCAGAGAGTTTTTAATTCCTTTGTTGTTT<br>GAATATAGAGGATTGttaaatgaacccgaagggacca<br>ccgaatctgtcatttcttagacggacgtcacaccctctggacccaa<br>gctagggacccaacccttctaggggacctcctcccgaaccgttgg<br>gtgaggtcataagaacggacctcttagggtacttgtctcctcggatc<br>gcccgatgtcaggtaccccaacgtttctcaacctgtactgactcact<br>gattcgtgtcgtatatcTTATTTGGTCAAGGAAAGT<br>GAGAAACATTCAGTTGATTTCAGTaaatacat<br>ttcatgaatcgtgtcGAGCTATTTCTTAGCAAACA<br>CATAATGAATGTTAGTTTTCTTCCATGCCa<br>agaaggggggggtggggacaaaaaaattatttttaagatatttaaac<br>TGT |
| AC_000171.1:63954121\|64038908 | aatggcaacccactccagtgttcttgcctggagaatcccatggacg<br>gagaagcctggtaggctgcagtccatggggtcgcacagagtcag<br>acatgactgaagcgacttagcagcgtcTAGGTTTCTGTC<br>CGGGACCCACTGGGCccactcttttttttgtttgttttaat<br>gtttgtaGCAGTTTCAGTTGCTGTCTGGTGGA<br>TTTTTGTCCCCATTTggcccattcttttttttttttttaatg<br>gttgtagcggtttctttttattttcttttatggctatgctaggtctttgatg<br>ctgcacttgggctttctctagttgtggcgagtgggagctactcttcat<br>tgcggctCCTGGgccttctcatcgtggtggcctCCCTTAC<br>GGCGGAGCCCTGGCTCTAGGTGCTCGGG<br>CTTctgcagttgcagcacgtgagctCTAGAGCgagggct<br>cagcagctgtggtgcaggggcttagctgccctgtggcatgtggca<br>tcttcctggaccagggatcaaacctgtgtcccctgcattgccaggc |

| | |
|---|---|
| | agactcttaactactgggccaccagggaagcctccgcTCCAT TCTGACCCGTCCTCTAGTTGGACCAGGA GCAGCTTGGATGCCACCTGTGCTTTAGAC ACTCACCTGGAGACCCATCCTTTCCATTA CGATTCCTGAGGTATCTCTGCCCTCCCGC CCAGTCCCTCTCTTTCCTGATGGTCCATT GGCCTAAGTATCTCAGCTCCCAGCTAAAT CCTGTCCTGACCTGTTGTCGCGTTGACCC AAAGCCCActgtcacctcctctgagaatTCTCACCT CCCTGGACAGGGACAGTGTCTGATGCTG TGGTGGCCCCACACCCGGGGCAGAGCTT TGCGTGGGGCTAATAGGGACGGACTCAA CCCACCGAGGGACCCAGGACTCCCCCTC GACTTCCCTGCCTGAGCTGGCGGGATTTA TTACTGGCACAAATGACCCTTTAGGACTT CTCTGGACCACAGGCTTCCAAGAACATT CTCAGGCCAgacaagagaaagggaaggaagtaaAAT AAATGCCGGTGGTGGGGGGGGACCCACA CCTGGATTCCAGGGAAATGTCATGGGCT GAAACCTCCTCCACAGTGGCAGGTAGGC AGAACCTCCTATGACAGGGCCCTGGCGG CCTCCCTGCCCACAGGCAGGTGCCCACA CTTGGGGAACACAAACTGAGGATGATGT GGTCTTATCTGTGTGGCTGCGGTAACCTT TTTCCAACATTAGATGGGCACTTCCAATC CTTACCTGTGAGCTCTGTCTTCACTGGCT GATTCAGGCATCCATTCTGCTGTCTGGGG AGCCCGCGGGAAATCAGCCATCCACATA ATGTCCTGGCTTTTAGGATTGGacggtggggg caggggtgaggagggggcagacacgcgtgtgtgagagagaga ctgaaCCATGTTACAGGACGAGCATTAATAC TGCATTTTGCTTTCAAGCTACACAAATGT TTATGGTGGTCTTATTCTGTGTGGGTCCTA CTCCCAGGGTAAATATTCTAAAACGCAgat cttaaaactcagcattgagAGTCACCCCACTATCCA CAGGTTAAGGCCCAGATTTCTGATGGCAT CCAGGGCATCTCACCATCTGACCTTGGCC TGCTGTTCCAGAATCGCCCACTACCACCC TTCCCACAGTCTACAGATGCCCAGGGAC AGAGTGTCCACCGAGCACATTGCTGCCT CCAAGGAGCTCGGTGCTGGCATGGGCAG GGCCCCATCCTCTTCCATGGGCCAGGTCT ACCTGGCCAGGTAGAGAGAGCCACTTAC ACCCTGCATGGTGACAAGAGTGGAGGCA |

| | |
|---|---|
| | GATGGAGCCCGTCCGTCTGTGTGATACTC TGGCCCTGGGCCCAGCCTCCCTGGCTTCa ggcttcctctccttccctccggGCCAGCCTGTGGCCG CTTTCCCCACCCAGCCCCGCAGCAGGCT CAGGTTCCCTCCTGGAAGGGGATCCACA CACTGGGGGTGTCCACCCAGGAGGGCAG AAATGGGGAGCCTGCCCTGGGCTCACAC CTCCTTCCTGCTCCAGGCCTCTGCTGGTC GGCAGAAGAGCCAGTGCTTACGGGGGCA GCCACGAGGTGCTGGCAGAGGGCAGTCC GCGCCCCTGGCCCcagttctgttttgttctttggagGG AGAGGGTCTGGTTCTCAGCCTCCCCGTT CTGACAACAGGACTTTTCTGTGTGTTCA GCGACAGAGCTAGAATTATAAATTCCACA AGACTGCTGTGCAAACTAGCACATCCATT TATTGCTgtatggtggtgggggtggtggggataTCAGT TGAGATAAGTTTGGCCACCGTAAccaataaat gcagaaaacagAAGCTAACGTGGCTCCTGCCC TATGGCCACTCCTGCTTCTACTGAGCAGT GACTCAGGAATCCAGGCTCATTCCTTCCA TCCCTGGGCCCCAGCAAGATGACCCAGA GATCATCTCCAGGCCACCCTGCCCGACA GGGAAAGAGCAGGGAAGGTTGTTGTATG GAGGAGGAGATTGTCTGAGcgctgctgtaacaaa tggccACGACCTGGGTAGCTTAAAAGAACA ATGGTAGATTCTCTTACAGTCCTGGAGAC ctgaagtccaaagtcaaggtgtcagtGGAGTTGATTCctc tggaggctctgagggaAGACATTTTCCAGGCCCC TCTCCTAGCTtcggggcagggagggagggggttgTGT GTCTGCTGACCCTTAGCATTCCTGACTTA CAGAGCATCACTTCCGTCTCTGCCTGTGT GTCTgcgtgtctgtctgtcttctcctTTGCTGTCTTACA AGGATACCTGTTGTTAGACACAGGACCC ACTCTAATCCAGGAggatctcatctcaagatccttgcC TTAATTACATCTGAAAAGACCTTTCTTCC AAAAAAGGTCACTGTGACGTAAGAAGCA TATATTTAGTCTCTGCCCTCAGgtcctgacacag agctcctaaaatcctTAAAATCTGAGTGATAAATA TGCTAGGACatcttttgtttttgatgtctgggacttccctggtg gtccggtggctaagaccacaggctcccaatgctgggggcctggg ttcgatccctgcagggaaatagatcccacatgctgcaactaagagt tcacgtgccacaactgagagctggcacagccaaacagataaata aatattaaaaaaatttttgatctGTGCCTGCATGTCCT GACCCAGAGCTCCTAAATCTCTCGGGATT |

| | |
|---|---|
| | CCCTGGGTTACTGGAGCATCGTTTGTTCG<br>AATGAGGTGACTCTTGGTGGGCTTCTGG<br>AtggcttcaggatgggggctgGGCACCAGATAGAC<br>CAAGTCACGATTAGAAGCTTGGAGTTTTC<br>AGTTCCaccccccacgcccacccccattcttctggggaagg<br>gagagggactaGAAATGAGCTCATGATGGATC<br>ATACCCACATGATGAAGTCTccatgtgtgcacgct<br>cagtcatgtctgactctttgcgaccccatggactgtagcctgccag<br>gctcctttgtccatgggattctccagcaagaatactggagtgggttg<br>ccgtgccctcctccaggggatcatcccaaaccagggactgagca<br>gattctttactgctgagccaccaggaaagcccaaaatcTCCAT<br>AATAATCCCTAATCTATGAGGTTTTGAGA<br>GATtcaggttggtgaacacatccTTGTGCTAGAAGG<br>GTGGTCACTTCAACTCCATGGGTACAAA<br>AGCTCCTGCACTTGGAACTTTCCAGACC<br>TCACCCTATGCACCTCTTCACTTGGCTGT<br>TGTTCACTTGTATCCTTCACTATATCCTTT<br>ACAGTAAACCAGTAAACATAATTAAGTTT<br>GGTGAGCAGTTCTCACAAATTCTCAAAC<br>CTGAGGAGCAGGTCATGGAAACTCCCAA<br>TTTACAGCTGATTCGTCAGAAGTACAGGT<br>GACAACCTGGAACTTTCGATTGGTGTCT<br>GAAGTGGGGGACTGAGCCCTTGAGCTGT<br>GGGGTCTCTGATAATTCCAGGCAGTTAGA<br>ATCAGAGTTCAGTTAACTTAGAGGACAC<br>CCAGTTGGTGTCCCCGGAGAACTAGAGA<br>ACTGGTTGGTGTGGGAACCACCACCCCA<br>CACACATTTGGTGTCAAAAGCATCCTGTG<br>GGCATAGACACAGGGTTTCTTTTAACGAC<br>ATTCTGAGCTTCTGGCTGGACATAAATTC<br>TGGGGGCACTGCCTTCCACTACAGGGAC<br>GTAGCTGGATATGACGCACCTGTTGTGCC<br>CACTTTCCATTGGCCAGAAAGTGGTCAC<br>ACGACTTTACTACAAGAGATGCTGAGAA<br>ACATCATCCAGTCGTGCCCAGGATGACC<br>AGGAAACTGGTTTGAGGAACAGCTAACA<br>GTGTCTGCCTCATGGCGAGGGGGGAGCG<br>GGGAGGAGAGTCCTTGTCCAGGACCAGG<br>AAGCTCAGGCTTCTAGAGAAAAATGACC<br>CTCCCTTAGGCAGAACCGGATATATACTT<br>ACTAATCTCTCCAGATCCAACCATGCCCC<br>TCCGCCTTGGCCTTGCAAGTCCAACTTCT<br>AGTTCCAGAACTTGCCAAGAATCCAGTC<br>TGTAGAGATGGTCCCTGAGGGCTCCAGT |

| | |
|---|---|
| | TAAGAAGTATTCCTCCCCCCCCTCCCCTG<br>TAAGGGTTCcagagatggagggaggaagggtACATG<br>GAGTAGTGGGGTTGGGTAGGGAGGAGCA<br>GGTGCCCTGGAGACTTTGAGAGAGACAc<br>agccccacccctggcccctgcGACTGACCCTCAAC<br>GCCCTAAGATTTCAGTGCCTTATGGGAGG<br>AGATGCGTCTGCTTCCTTACTCCTGTCTT<br>CAAAACTATGCAAAACCAACTCTTCATCT<br>TCTTGCTGCTttgccaagatttttttttttaagtattgttcTTG<br>GCTCCCTTGGTTTTCAGTTCCCTAAACAG<br>CAGTTTTGAGCTGTTCAAGCTATTTTAAA<br>GTGTTCTCTTTAAACTGAGACCAAACCCT<br>ACTCTTTGGGAAAAACTATCACTTCAGA<br>GGAGATATTCTTTTCTCCCCATTCCTTTCT<br>TCTACTGCCCTCTGAACGATCCCTGTTCT<br>CCAAGAcgcttgtgttttattttttgctttatgtcAAAACAA<br>CCTTCCCTTTGAACCACTCCCCTGCAGAA<br>AAACCTTTCCTAGAACAAAAATGTTTCAC<br>ACCCATGACACTATTTTGTAGCGAGTATA<br>CACGGGTTAAATGGCATAGCAATTCCCCC<br>AAGAGTCAGAAGCAAGCAACAACTTTAC<br>TTATTTTGAGTGGATGCCCTTCAGGTTTA<br>GGAACACTCCcgtttcatgatccagcagataagATAG<br>AACCCTGGGTGGCTGTGGCTGTTATTAGA<br>TGACTTCTGAGGTTTTCACTTCCATTGCT<br>GATCTGCTGCTTCTAGGATCAATTCATTTT<br>ATTGCCTGTCCATTTTTAGAGAAAGACTG<br>ACAAAGCCACTCTAATAGATCAGGGGTC<br>TGAGCTCAGAGTAGGTCAAAGGCACTCA<br>CAGGTAGGGAGGTGGATAAAAATAGGAG<br>GAGGGTTGGCAAGTACAGCCAGGACACC<br>TGCGTTCAGGGTGGACACTAACGAAGGC<br>CAGCCTCTCCTCATTCATCTCTGGAGGGG<br>AGTCATGATGGTTTTACAGGCTCAAACTC<br>AAACCACACGCAAATCAGGTGATTTCCC<br>CTGAGCATTCCATAAAAGGAAGATCCTTC<br>CATAGTCGGAGTGGCTGTTAAATCCTGGG<br>CTGTTCTGACCGGTCTGATGAGAAGAGC<br>TTGTCTAGAATGGAGCCAAAGGTTTGCTC<br>GCTCACATGTAACCATGTGAAAACGCTC<br>ATGGACATCAAATCTAcattgttggacttccctggtgg<br>tccagtggtaaagaatccctcccGCTAGACATAGACG<br>TGTGGACACCAGGGGAGGGggtgaaggagagg<br>aagggatgaatgggtgggatgggggagggggagggagatc |

| | |
|---|---|
| | AAGAGGGAGGCGGTATGTGTATACCTACacctgattcatgttgctgtatggcagaaaccaacacaatatttaaagtaattatctttcaattaaaaacaaatacaattttttaaaaagtaaaatataaagtcaatacaaagtaaaaaaaaagaacccacctgccaatgcaggaaacacgggtttgattcctggtccggtaggatcccacatgcctcgggacagtgaagcccacatgccacaaccactgagcctgcggcctagaatctgtgctccacaacaagagaagccgctgcagggACAAGCCAGCGCGCCGTAACAAAAAGCAggccccactcgccgcaactagagaaaacctcgAGCAGCacggccagaaagaaaaaaaagaaacggGGGATGTCTTTATTCTCGTCTGCATTTGAATATTTGGAACCCACCTGAAAAGGTATTTATATGGTTTGCCCCTGGGCCTTCATGGCTACCTTTATTCACACTAATAAAGGGTTTTTCCATTTGCAGGTAAACATGTTGGAGGCAAGTTTTTAATACTCTGTGTAATCAGTATCTCACATTTTCAGAGGGGCCTCAATCCAGaataaagatgagaaaaggaCTCTGATGGTAGCAACCCCACCAATTATGAGAAAGTCCCTGTACCATCTGGGAAAGTCACTTCATGagtctttgtgtttttttaatcagatatataaccaaatactttggccacctgctgcaaagagctgactcatttgaaaagaccctgatgctgggaaagattgaaggcaggaggagaaggggacaacagaggatgagatggttggatggcatcaccaactcaatggacatgagtttgggtaaactccaggagttggtgatggacaggcaggcctggcgtgctgcagttcatggggtcacaaagagttggacacgactgagcgactgaactgaactgataaccaaAGGATCTGTATTTCatgaatttttaagagaaaaggagCTGAATTTATCCTCTATatttactttttccatttttctcaagTTAGTATTTAAAgacaaaccaacaaaaaccttTTAGATCCCTGCAGTAGCCTAAAGTCATATTGCTTTGGCAACAGTAGCTAGTCCACTGGACTAACCAAATATCAAGACAGAAAATTGTTAATGCactttaaattttacaaaatgtCACATGGTTCAAGATCATCCTATAGTCATAACTGACCTTCCAGCCTAAGATAATAAAATTGCCTTTCTCCTGATTGAATGTAAATGGTCAAGAGAGATTACAGAACTTGATTCTTTTCAGGATGTCCTAAAAATTCTAGAATGGTTTTTCTTTTGAGGGAAGTGTGTTATCCAAATCAAAGGGATCTGAAATTACAAATCCTTTCTTGAAGTTGGTTATTCTTATGCAAGTGAGTGGTAAGTCCAAAATCAAACTCAATCAATGAGTCAAGCATCTATTTCTATGCTACTTGAAACAGACCATATGTAGTAGaacttcagagaaggcgatggcaccccactccagtactcttgcctggaaaatcccatggatggag |

| | |
|---|---|
| | gagcctggtaggctgcagtccatggggtcgcgaagagtcagaca<br>cgactgaatgacttccctttcacttttcactttcatgcattggagaaga<br>aaatggcaacccactccagtgttcttgcctggagaatcccaggga<br>caggggagcctggtgggctgccatctgtggggtcacacagagtc<br>agacacaactgaagcgacttagcagtaggaCTTCGGGGtc<br>tttcctggcagctcagcaggtaaagaactgcctgcaatgcaggag<br>accctggttcaattcctgggtcaggaagatcccctggaaaaggga<br>taggccacccactccagtattcttgagcttccctagtggctcagatg<br>gtaaagaatttgattgcaatgcgggagacctgggtttgatccctgg<br>gttgggaagatcccctggaggagggcatggcaacccactccagt<br>attcttgcctggagaatccccatggacagaggagcctggcagga<br>gtccatggggttgcaacgagttggacatgactgagcaacaaagca<br>tAGTAGAACCTCAGTTAGTGTTATGCTGA<br>ACCTGTGGGTATCTGCTACATCTTGTGAG<br>TGCAGAATCAAAACACAGTGATGCCTAC<br>ATATACTTTCAGTCTTTTCTATACTAGACG<br>TCTCACTAATGTGGGAACTTTCTGCTTAG<br>TCTATGAACTACTAAACACAAATGACATA<br>TAGTATATGTTTTCCCTAAGTTAGCAAAA<br>ACTTGGAAGCCCTTTATCTGACTGCTGTA<br>TTCTTTGGAATGCCTCTTAAGAACCACTA<br>GCTTTTAGCTCAATAAACATGGAAGCCCT<br>AAAGACAAATTTTGAAAGGGATGACTAA<br>TTAGGGAAGGTTTGTGCTTGTCCAATGAT<br>TTTGGTGCTTTCCTAGGGGTAGGAGAGC<br>CCATCTAAGCTAGCCTGTGGACAACAAC<br>TTTATCCCTTTGAATCTATGATGCTCAGAT<br>CTGGGCGAGAGCTTGTGGATCAACTATTG<br>ACTCTAGTTGTTTAATTTGACAGATGTAA<br>GAACCAAGATCAGAGGACACACTCAACA<br>ACCCTCTTGGTCACTCCTCTCACAGAGA<br>GACTCAGGTTCCCTGGAGATAATTACTGC<br>ACTGGGGCCATGGGCACTGATGATCTTG<br>ACTGATTTGGGGGTGGTCGGGGGGGTTG<br>TCCCATGTCCACTGGGAGGAGCAGGCTT<br>GAGTCTTATTGTTTTGCTCTCTCTTGGAG<br>CCAGTGAAATATTTACCCAATAGATATGAT<br>TCAGTTTGTACAAAGTCATCGTCTGCAGC<br>TGCCCCTGTGACCATGCATGTTTTGTGAA<br>GAGGAATTGCACACACACAGTCCTGGCT<br>TTCTATTTTAAATGGTCACAACCAAGTCA<br>GTCAAGTCAACGCAGAGACTGTATGTGT<br>TTACAGGTCAAGGAATCCACAGGGGAAT<br>CCTTTATTTTGTCTTCAATGAAAGGtccttta |

| | |
|---|---|
| | aaaagaataaactttggggacttgcctggtggtccaggggttaactccacacttccagtgcagggggctcaggttccatctctggtcagggagttaagaccccacatgctgtgaaaTGTACCAAAAGAAAGGGGTGGGGAAGAAcaaactttctaaaatatttcttttccatctctGGCTCAGAACATCATATTTATAACTTTGTATTTCTTATGACTTTGAAAACTAGATCAATTTCTCCTTGGAAACATCaggagaaaaatacatttctttcatgTTCCTTCTAGAAGCCACCTTCTCAGGGTTAAGGTGTATTGACTAAAAGAGCTTCCTTCTTCAAGAGATCCTAAGTGTCTGTAGGGCACTTCATTGCCCTTAGAGGGATGGAGGGATGAAATCAGTCTGGTTGGGCGCAGCGCCCCGCGTCCCGACCCCCGCCCTTCCGTGCCGGTGGTCCTGGAGCTgaggaggaaggcaggggaaAGAGTTGAGTTGCGTTAGCGCAGGCGGCCACTAGAGGGCGCCGGAGTACCGGCACGCCCACCTGTAGCGCCCAGGAAAGCCCTCTAGGCTCGGGAGATGGAGATGACCCCAGCAGAATCCCAAGGCGGTGTCCACAAAGGAGGGCTTGGTGCCTGGGTGCCTGTGACTCTCCGGCCCAAATTACCTCGCTTCAGAGGAGGGACAAGGTCTGCTTTCAGCAGCTGGGGCACTTCCAGCCTGGCAGGCTGTCTGGGGTCTGACTGCCCTTTGTTTTTCCCAGAGGAAGGCCTCTGGACCCAGAAATACCTGCCTTGACAGTTTTCAAGCTGCGGGCCTACACAAGGGTTGGCATCGGGATAACGGGGCTGGTGGGCGTGAGTGTGGAGTGTGTAAAAGTGGTGGCATTTTGGTGGGTGGGCTGTGCTCATTCCCGTGACCTTCCTCCccattcttccttcttcctttccgcCTGAGTCTCTCATCCTCTCCCCACAACCCTGCCCTCAGCAGAAGGGTGTCACTGTTCACGCTCTATGTCAAAAGCTGTATTAGGTGCTGGGATCAATGTGAAGTTCCCAGCTTTTGAAGATGCAGGCTTGAGGGGCAGACGGTGATGTAAGTTAGGGTCATGTGATGCAAAGTCACATCATGTGATGGAAAGTCACATCATGCGATGGAAAGCCATCACCTGACATAAAGTTGAGGCACCTGAAGTAAAGTCCAGCACCTGATGTAAAGCCAGACATGATGTAGACAGCCACCTCCTGAAGACAGAGATTTTTATCTTGGACCAGCCTATTCAGGGAGACTTCAGAAACCATCacttgccaggaaaaaaaaaatcttttagctTCCAGGTAC |

| | |
|---|---|
| | CTTGTACCTGTCCACTTGCTGTCTAGTcaag<br>tgataaaatatttttatgagcaATGGGAACTACAGAAA<br>GCAGTCAGTGATAAGGATCCTTGGTTTAT<br>TATCcttggtttatttccttttcattaggTAATCTTTAAAT<br>TCATTAAGTTCATACACTTCACCAGAGTT<br>ATATCAGGATCTCCTGAGTTGGAATAGCC<br>TCTGTGGTTGTCACAGACACggggaccatcccc<br>atgggaagaGTGGAAACCCCAGGGGCCTGTC<br>TGCTGAGAAAAAAGAGTTTCCATCAGGC<br>ATGTCTGTATATTtctaagggctttcctggtggctcagac<br>ggtaaagaatttaccttcaatgcaggggacctgggttcaacccctg<br>ggttgggaagatccccctggaggagggcatggcaatcccctcca<br>gtattttcgcctggagaatccccatggacagaggagcctggtggg<br>ctacagtccatggggtcgcaacaagtCAGAGAcaatgagcg<br>actaagcacagcacagcaccgcATAGCACCACCAGG<br>AGCAGGCTGCTCGATGCTGCCCGCTGAC<br>ACTGGTCTTCAGGCTCTGCCACTCTGCCG<br>TCTCCTCCATCCTGCTTCCTTTCCCAAAC<br>TGGCATCAAACTTCACAGCCCATCAGTC<br>ACCAAATCCCCTTTGTTTTCTGCCCTCAG<br>TATTTCCAATCACAATCCTCTTTTCACCAT<br>CACCTCTGCCCCACCCTGGTTTGCTCAGG<br>GGATTATTTCAAGCCTGAGGATGAAGGTT<br>GATAGAAATGAAATGATGCAGCAGCACT<br>AGCACAAGAGTGAAGAAAGAGAGGCAC<br>CCAGGACATGAAGTTCAGGAGGCATCGC<br>TCTCAGGATCAGTGTGCGGGGTCAGCTC<br>AGAGGAGTGCCTTCCCGAGTTCATGCCC<br>TAGTCATCTCCCTTGACTTACCCTAAATC<br>CagtcacatgatttttttaaaattttaatgtaaattgtTGCCAAA<br>ATCTATTAGCACCCATCTGTACGTTATGTA<br>CACTTCTCCCCTCACCACAGGTACTCATC<br>CAGGTGAGATACCTGGGCTCAGATGCTG<br>CCACTTGGTGGCATGCTTGTAAGATCTTG<br>CCCATGCAAAATGTTCAGTTATGTTTCTT<br>GGGTTGGCATTTTTCTTTATGGTGGCTTTT<br>CCCTCTGCTCTTTATATTATCTGATTTCATT<br>CTCTGGTTTTACATCAAGACACCAACATA<br>TTTTTGTCCTCACAGGCTACCCTTGACAA<br>TTAGGTAATCAGCACTTGGGACAGGTTGT<br>CAGTTGCAGAATTAAGAAACACACAGGT<br>AGATGCGGCACGCTGAGGGATATCGTAA<br>ACAGAAGTGACAGCAGCCACAGGTACTC<br>GTTATTAATAGTAACGtttgggacttcctggtggtcca |

| | |
|---|---|
| | gtggtttagactccatgctcccaatgcagggggctcaggttcaatc<br>cctggtcagggaactaagatcccacttgtaACAAAGATCC<br>CCTGTGCCAACACTTAgatctggtacagccaaataa<br>aaataataaattaccaACACCTGCTAGTGTTGGTT<br>TGCCATACTTGCAGTGATAGCAGTAAATC<br>AGAGCAGAACCAAGGTTCTCTCTTCAAT<br>AACATCCTTACATTCTCTAGGATTCTCTAG<br>AGTTCTAGacggctttcctccacaggagGGAAAGC<br>GGTGGCTTTACCTTTAGCCTTCCCAGCAC<br>ATTCTTACCTTGTGAACACGATGCACACA<br>CGTGTGAGGTGCTGCGATCTCTTAGTGTG<br>TCTCCTcaggacaaaaataaaagagaacgGTGGGTG<br>TGTGTTGGAGCCTAGTAAATCCAGGAGC<br>CCTGAGATGTTCGACTTCTCCAAACACTG<br>TCTCTCAGGTCCCGCTTGGGTGTTTTCCT<br>GGGAGAGATTTTCCAGGAACTGTCTGCA<br>TGTGCCCAGACCTCTGTCTGGTTGGGGC<br>CCCATggagcaggggtgggtgggggcggtTCCTGGA<br>GAGTTTGCCCCTCCTTCTTGTTCACAAAA<br>TGCGTGTTGGCACCCCCATTTTCACTGGC<br>TTTCCATACAGTACTTGACTAAAAGGCCC<br>TTTCCCTGTTATCTCCCTCTTTTTCCAGTG<br>GATTCACCTTCAGGATGAACTCAGTCTCA<br>GTAGATACTGTAGTTCACTGGCCCTTTTA<br>GGTCAATCAGGTCATCCTCAGTCACACTT<br>TCTCCCAATGTTTTACACACTCATATACAC<br>GTTTCCATTGCCCTTTCCTACTTTTACAGG<br>TGCTGTAGTCATAAAGAGCTGTTTATTCA<br>GGACATTTCTTCTCTCCTAAATTACTGTA<br>GTTGCCTCCTGGTTAGTTTTCCTGCCTTC<br>AGGCCAAATCCCCTCattctttatagaaataaaatatac<br>aggaATACAACATACCCATGAGACATCTGG<br>ATATGCTGATCACAGACTTACTCGTTCTC<br>TTTTGTGTATAATGATTCGATGAAGAGGG<br>AGAGTCTTTGGAACCACACAGACTTGGA<br>TTTAAACCCTGGTGCATCCCCTAAGACCT<br>TGTGATTTTCATCACCTTTGGGCATCAGG<br>CTcttcatcaataaaaaataataacaatctcTACTTCCTAgc<br>atttttatgaaaatgaaatagcaCAATGCCTGTAAAAC<br>AGTCAGTACAggtgtctgacacatagtagggttgttttttttt<br>aaatgcattctctatttatttgtttgactgcgttgggtcttagatgaggc<br>atgcgggatctttcattaaggtgcatggactctctagctgtggcacg<br>caGACTCGGTAGTTGCTTAGGCCTGGGGA<br>ATGCTAAAATAAGAAGGCATAGTTCTTCT |

| | |
|---|---|
| | GATTTCTAATATCACCATCATTTCAATGAAAGTTAACCTGGCAAACATCTCTCCAAGGAAATGCTTAAGCCTGGGAGTGGAGAGTGGTGAGAAAGGGGCGGAGATGAAAGGAATGATAGGGAAGGAAAGGGACGCTCATAGTGCACCCAGCCCACCAGCACCCATTCACCGGCTGATTGATGACAGGAGGGCTCTATTTTCTCTTAAGAACTTTTGAATGAGAAGCATTACTTATATCAAATTATATCATAACAAATAACCAGAATGAATATCCCACTGGACTTGAGGTGGAATGTTAattctggaggacagaggaaaggGACTATGTGCTCTGTATCTGCTTCTGAAGGATTAGATTGTAAGAGCAAAGTTGATGGAGAAAAGTGCCACCATGTCTTATGTTGTAAGACTGCAATCAAATCAAAATAACTAgaagagaaaaagcaatagaCTAACAGTAATCACTGACAAtcaatctctctttttctttcaaaaaggtttttttttatcactgaattTTCCCTCTTTTATCCTCACCCCTCCATAGGACTatgctgaaaataaataataacaagtgaaagtcactcagtcatgtctacctctttgcaaccctacgggctatccagtccatggaattgtccaggccagaatactggagtgggtagcctttcctttctccaggggatcttcccaacccagggatcgaatccaggtctcctgcagtgcaggcggattctttaccagctgagccacaagggaagcccaagaatactggagtgggtagcctatttcttctccagcggatcttcccaacccaggaatcgaatcggggtctcctgcattgcaggcagattgtttaccagctgagctaccagcgaaGCCCACATAATAATAAGCAGGattcaaattctatttttagaaTACCCAGATGTTTTCAACAAGATCGTACAAACTGATAAAATGCTAGTACTAAGTGTTTCGAAAGGAAAGTTCATGTGAAGAAAAATTGCACAATATTTCCCTAACCCTTAAATGTCCTTACTTTGGATaacaaagggaagaaggaagggttACTTCTGTTAAAACCCTacgatggggacttccctggtggtccggttgTTAAAAATCAGCCTGCCGCTGCAGGgtacatgagtttgatccctggtcagggaagatcccacatgtcatggagcaacctGTGTACAACAACGATTGAGCCTGCACTTTACAGCCTGAGAGACGCAATTACCGAGCCCAAGTGcagcaattactgaagcccacatgcctagagcccgtgctccccaacaagagaaaccaccacaatgagaagcccacgcaccacaacgaagagtagccccagcttaccccaactagagaaagcccatgtgcagcaacgagGTTCAGCCAAGTACAAGCAACTCAGCACAggccccccacccaaaaaaataTACAGTGCAAATCCATGCATCTACAAAGCATCTCTATGTtctacagacatagagaacaggcttgtggttgccaaggttgGGGGGATGGGAG |

| | |
|---|---|
| | GGTAGGAacgggagtttgggattagcagaggcaaagtAA<br>GTACCTACAACAAGGACGTTGCTACAGA<br>TTGAGGTCGTGGTCCCGGGAAGACTGAC<br>ATAGGAGGCACTTCAGTTAATAAGGCCG<br>AGGTTAGTTGTAGGGGCTGGGTGGTTAA<br>GGGGAAAGATCCTGGCCTGACTGGAGCC<br>AGGCTTCTGTCTAGAGGACTGGACTGGG<br>CAACTGGTTGTGTAAAGGCAGTGAATCC<br>TCTACTATTGGGTGAGATGGACAAGTTCC<br>CAGCAAGGGGACTGGATTGGACGAGAA<br>GTCCTCCGGAGGCCCCTGCGTGgtaagaacat<br>cttttttttctgggTGTCTTAAGTCCTAGTCTCTGA<br>TCTTATCTCTGTTTTATGTGGTCTTGTCTG<br>TCCTAATGCTCTTGGAAATCTTTGCTGCG<br>AATGTGAGCATGGGCTGAGCACTTAAAG<br>GCCACTAGAGTACTGGCCACTGGAAGAC<br>CCCCATAAAAAGGTAAGTCAGTCCAGCC<br>CAGGGTAGATGAGCCCTAGGCACCTTGC<br>TGCCTGGAAGCAAATGTCTGGGTAAGAA<br>AGCTttaagttttagttgctcagtcacatcctttttgcgaccccata<br>tcctactctttgcgaccccatggactgtagtaggcACACAGC<br>TGTAGTAGGCACCCCTATGACCAAGAGC<br>CAGGTCATGTGATTTGAAGGGGGCTGGT<br>GGCCCTTCAAATTTTAATTGGGGTTAAAC<br>CACGATCCAGCACCACCCTCCTGGCGGG<br>GGGCAGTGTGGCTGTCTGCAAACTTGAG<br>GCTGGTTCCCGTTGAGTCAAATTCCAGTC<br>CTGAGAAGCCAGCTTGGTTGGTCTGGCA<br>GTTTGCTGGGGAGAGAAGCGTTTGGTTG<br>CACGGTGGCGTTCCTGGGCtctgggccagggct<br>gggggtgcTCAGATGTGGCTGAGCCACAGG<br>GCGAGGCAAAGGGGGCAAACGAGAGAG<br>GCCAGTCCCCAAGCCAGGGTCCAGGAGG<br>TCTTGCCCGGCCTCTCCAGGCCTGAGCTC<br>CTCCAGGAGACCCTTTCTCTGGATGCTAc<br>actaaataaataacttaataaCTGCTGAGTACTTGTC<br>AGTCATAGATTCTGTGCAAGGCTCCAGG<br>AAGACCACCCTGGACAGCACATCATTCC<br>TCTTTTCCAGTTGTTTGCAGTCTAGTGGA<br>GAGCTTGTTGAAAAGGCCAATTTGTGAG<br>ACTCAGCCTCAAGATTCTGattccacaggactgg<br>gaggGACCCAGTGCATCTCTGGTGGTTATG<br>CTGGCGGAGCTGTCTTCCCTCCAGGCTT<br>GGAGAAACCCTGTTCTAGCCTGAACCCT |

| | |
|---|---|
| | TTGGGGAAGAGCCAAGTGAGACACAGC CCTTGAGAATGTTGCCTGCTTCAGGGTAG CCCCCCAGAGAGTTAGctctggaggaaggagagg gtgccCCTGGTGAACAAAGCAAATTGTGGG GAGATGGAGATGCATTTTCTCTGGGCTGG GTCCCCTGTTTCCCACCAGGGGGTGCTC CTTGGTCAGTCCAGCCTCCTTGAATTTGC AGCTGTTCAGCATCAAGTGAAGATGTTTT GCCTGGTAGGAGAACTCACTGGGGCTGG ATTCCCCATGCTCTGTGATTTCTGCAGTT GGCCTGGCTGTTGACTCTTGGATTATTTT CTGGACACCTTATTTTTCATCGTAGTTTTC TAACAAAAGAAACCAGTACCCGATGAGT CTCAAAACCAGTGCCCAGTTCTCACTCAT TCTAGAATCTTAAGCAGTATCTCTTTTCA GACTAAAAAAGCTATTGGCCCTACTCAA CCTcaaacccattaaaaaaaaataatgcagaatCTCTGGA TTAGAAACCTGTTAAATGCACACAATGG GATCAGAACTCTATTCTCTGCCCGCTACC ACCCCTTGCCCAcacaaagcttttaaaattcGATGT CAAAACTATTAGaacaagtttttaaaggaataacctaga ggagaacataggcaaaacactctccgacataaatcacagcagga tcctctatgacccacctcccagaatattggaaataaaagcaaaaat aaacaaatgggacctaattaaacttaa |
| AC_000175.1:46155528\|46202870 | GTTGATATCTCTAGTTATATTTGTGGATTT GCCCATTTCTCCCATCAGTCCTGGCAGAT TTTGCTCTAAATTGTGTTTTAAATGTTGTA TGGCTACGACTTAAGCCTTCTGAAGAAC AGACATGGCCTTGGTTTTGACTGGTCTTC CTCCATTTCACTTGTGCTGAAGTGGTCTC CCCAAGCCACAGGCCTGACTACATGCCA AGAAGGGCCCCAGACTCGAGGGCCTAG ACCCCTCGGGGAAGAGAACACAAGGTC CAGACCCACTGACAGGGCATTCAGGGCC TTCACAACCTATTTCTGCCACCCTTTCCG ATCCCCCTGCCCCAACTCCTTCGCCCACC AGAATGTTTCCATGTCGCCTTTTCTGGGT TCATGATGCCTGTCTGCCCACTGTCCTTC CTCTCAGAAACCAGCCCCATCTTTCCGTT CTAAACCTTTCTCTGAAGAGACAGTCCA CCCCACTCCACGCTGAAAGCAGTACTCC GCCCGGGCATTCCCCAGGCACTGGGCAC TGTCTTCAGTTGTAACCCCCAGTCTGTGG TGTCAGTGTCTACCATTCACGTGAATTTC |

| | |
|---|---|
| | ACGTTGGTGCTGTGAGTGCTGGCACGAC<br>AGCGGTTGAGTCCACCATGGACTGGCCC<br>TGCAGAGTGCCCTGCACACAGTAGGGCA<br>AGCAGTAAGTTTTGAAGAAGTGAATACG<br>AGCCAGAGGTGTCCCTTACCCCCTGCCC<br>GGCCCCCAGTGAGGAAGGGAGGAGTGG<br>GCATGGGATGCGGCTGCAGCATGGGACC<br>CAGGCTCAAGGATGTCAGACTGCAGGAG<br>GGTAGCGAAGGGCAGCTGTCTGCTCGCG<br>AGCTCCTCAGGTGGCACCTCAGAATGGA<br>TGTGGAGGCTGGGCCGGACTCTTAGCTC<br>AGCTGCTGTTCGGGGCCACAGAGTCCCC<br>AAGGACCGTAAGACATGACCGCCCTGAC<br>TGGGAAGGTTCACTCACCAGGGTAGTGA<br>TGGGGCACATGGCTGTGTACTCACTGAC<br>CCCAGGGCCAGGGTCAGGCGAAGCTCG<br>ATACCGGGCAGTATTCTTATCCCTGTGGT<br>AAAGGGAGGTTGTGGGCAGCAAGAGAG<br>GAGAATGAGGGAGGAAGCAAGTCCATGT<br>GGGAGGGTGTTGTCACCCAGGCCAGAGT<br>AGGGGCACTTGGAAGGACCGGTCAGCA<br>GTGAGAGGACAAAAGCAAGTGCCAGGG<br>GAAGACAGTCGGGTTTTCTTCTAGTGGC<br>CAGGCACGTCCTTTGAGGGAACAGCTGG<br>GTTGAGGGGCAGTGCTGGCCTCAGGAGG<br>AGAGGGGGTCAAGGGCAGCCTTCTGGCC<br>CACCCCACTTTTCTCACTCGCATCCCGAC<br>CCTCTCCGCCAGTCGCCCAAGCCTGAGA<br>AAATACTCTCCTTTACCCTCCTTTCCAAAT<br>CCCACCCACAGTCTCCCCTTCCTATAGTG<br>ACCACCTGCATCTGTCCATGGACTGCCTT<br>GGCAGGAGGGTGAGACTGTGGGGCCAA<br>CTGGGCTTTCTTGTGTGCCCCTAGCAGTG<br>CCTCGTACTGTGCTTAGAAGGTGAGGAA<br>AGGCTGGGACAGCTTTCTCAGTGAGCCC<br>CAAACCAGGGGTCTCAGGCTGCAGTGCA<br>GGCAGCCCCCAGGCAAGTCGTGACAGGC<br>CACAGCAGAGATGCATACGAGCCCTGTC<br>TGCAGGGGGCAGCTGCCACTCAACTCAG<br>CAGACAGAGTGAGACAGAGCAGACAGA<br>GTGTTATAAATCACACATCTGGACTTTCC<br>AAGTAAAATCTGCCAGTAGCTTGTCAAA<br>CCTGGCCTTCAAGATAGGGTTCGTGGTGT<br>TTCTGGGTGACCCAGTGCCCTGATGATGG |

| | |
|---|---|
| | GCCAGGCCCCTGAGAACTGCAGTGCCAG<br>CTGGGGAAGCTGAGACTTGCTGCCGGGG<br>CCATGGAGAAGGATTGTAAGTGGAGTGA<br>GATGTGGTCAGACTGGGATTTTAATGATG<br>AAGAGAAAAACAGGACTGTGGGGTCCA<br>GAACTCGGGATACATGAGTACCGCCATG<br>GAGAATCAAGTTCAGGGGCTGTGGGGAC<br>CAGAAAGGGTCATCCGGGTCCTGTGTAA<br>AGGGGCTCTGGCCCCTTCTTGCCTCCCA<br>GGGACACGTGCCCACTGTGCCACAACTC<br>CTTTTTTCAAAAGAGGCCAGTTGTTATGT<br>gaacttttctaatttttaaatgttgactcAAATTTTTCAAAA<br>CCTGCTGGGCCAACCAACAGAACAAACC<br>CTGCAGCCCCTCAGCTGTCGATGCTGCG<br>CTGAGGGTATATGTGGCACTTGAGGGTG<br>GCACTTGAATTTCTCGGTTCCTATTGAGG<br>AACCATGTGGAGCGGGCTAGAGCCAGCT<br>GCCTGGGTTCAGGTCGGGCCCCTGCACT<br>TGCTGGCCATGTGACCTCGGGCGGGCCC<br>AGCTGTGCCTCGGCAGCCTCGGCTCTCG<br>TGGCATCTGCCTCGGGAGGTAACTTGCCC<br>GGGCATAGAGGGAGCACACTGGACACAG<br>CCTTGTTCCTACTCTTTTCGCCGCAGTGG<br>AGCGGAGGCGGAGGGACAAGATCAACA<br>ACTGGATCGTCCAGCTTTCAAAAATCATT<br>CCAGATTGTAATGCAGATAATAGCAAGAC<br>GGGAGCGGTGAGTGTCCCCCTGGTCCCC<br>CAGCCCCCTTGCAGAGAATCCGTCTTGA<br>GTGCTGGGCGTGGGAGTTGTCTCGGGCT<br>GAGACGACTGGTGGGCATTGGCTGCCAG<br>GCAGTGCTCACTGATGCTCATGCCCACCA<br>CTCCCCAGAGTAAAGGAGGGATCCTGTC<br>AAAGGCCTGCGACTACATCCGGGAGCTG<br>CGCCAGACCAACCAGCGCATGCAGGAGA<br>CCTTCAAGGAGGCCGAGCGGCTGCAGAT<br>GGACAATGAGCTCCTGCGGCAACAGGTG<br>AGCCacagggctgggagggcagggacGGGGGGCGC<br>CCAGGAACCCCAGGACGCGGGGAGCCA<br>GCTCTGGCTGCTTGTCCCCCGTCGTGTCT<br>GGCAGATCGAGGAGCTGAAGAACGAGA<br>ACGCCGTGCTCCGCgcccagctgcagcagcacaaC<br>CTGGAGATGGTGGGCGAGAGCGCCCGGC<br>AGTGACACCACCTACCATGCGGCGGGGG<br>CCGCCTCCGGGCCCCCACcgtccccttccccagcc |

| | |
|---|---|
| | cttaGCACAGAGAGGGACAGacgcccctcccccag ctgcGTTTTTTATAGtagatttttaacaaaaaaatggGG AGAAATAATGCATTTCTGTGGATAAGCGC CCACCGCCCTCCTCAACTTGGAAACCAT ATCCCGTCCCCCATTTGTCTGTCTCCCTTC TCCCGGCCCCCACTCAACCCGGCACTTC TGGTGGTCTCACCTGGAGGCCAGAGGGA GGAGGACAGAGCCCCTGCCACACCCCGC TGCCTCCTCGGACTCCCGAGGTACTGAG ACCAGGGTGCTTATGGGGAGGAGGGGGT CCTGCGGGGGGCCTGGACCCAAGCAGG GAGGCCACGTCCCACCCCACCTCTTGTTT CTGGAACCCTGCTCCCCtctgggtgtgtgagtgtgtt ctaattttctttatggaaaaaatggacaaaaaaatagagagagaga ggtattTAACTGCAATAAACTGGCCCCATGT GGCCCCGCCTTGTCTGTCTGTGTATTTGT CCATCTCAGATGTGGGGAGGGGGTCTGG GGTCTACGCAGGGCTCCCGGGGACAGGA CCTCGCTCTTCTGTTCGTGGCTGAGTACC AGTCCCGGCCATCCCGCGCCGCCCGGGA CCCAGTCTGTTGGTTGTACAGATGGAGG AATTAAGGGATGAATAGTGTCTTTGAGGC CCCAGGTGGTGCATATGtggtgtgggggcaggg ggagccaGGGTCAAGGATATGCCACATGTTT TTGGAGGAGAGGCTGGGGTCTTTCATGT CACCAGTTCCTAAAAACAGGACTATCTCC TGACTCTGCCCTTCTGGGCTAACGCCCTC CCCTCTGCACTAGCCAATGGTGGGGCCT GGCCTTGAGCCCCCCACCCTCAGGGAGG GCAGATGGCCAGGAAGCCAGGCTTGGCC CGTCAGCCTGTCGCCTTGCACCGCGACT CTGGCGCCTGTGCTGTGACCCCTGCCCCT GGTTGATGATGAAACCTGGCCTGAGCTG AGATGATGCAGTGATGCCTGGGGGGTGC TGCTGTGTCTCCCTGTGGGTGAGCATGCA GCCCCCATGCAACTCCAGCCACTCCCAG AATCTAGGTCTGGTCACCTTGCTGAGCTT GCCACCCCACACCCATCCCAGATATCCCC GGctgctttcttccttccctctgagAGCCCTGTCTCCT GTCCTGGCCATTTCTCTTTTGGAGAGTGA GACTGTGTACAGCTGGGGGAGCCTAACA GATGGCAGGAAACTGTTACTTCCCAGGG TCCCATGTTTTCATGCTTTTCGAGTGGGC CTTGGCAGCAGTTCAGGGCCAGGAAGAA |

| | |
|---|---|
| | GAGTTCATTCCTTGGCAGCACCATCCCCA<br>GGCTCGTAGGGTTGTCACTGGGTGGCCC<br>AGGAGTGACAGAGAGAAGACAGCCAAG<br>GACCTATCTCTGAAGTCACGATCTGCTGT<br>TCCCCTGGGCCGTGAGCACCCTAAGATG<br>GTTCAGGTCATACGTGGCAATGAGATTGC<br>CACAGCCAGGCATCCTTGGTTCAGCTCCT<br>CTTCCATCGTATGCAGGAAGCCAGCCCTC<br>CAAAGAAAAGCCTGATTTGTAGTGTTTG<br>CCAATGTCTGTGGTGTAAGTACACCCACC<br>AGTGGGGGTTTCAAGGTGCCAACATGAT<br>GTTCCCGACTTGAGTTGGGATGCACACA<br>GTGTGCTCATTGAGCCAGTACAAGCGTG<br>GTCTAGCAGACCCACCATGCCCTCCTGTG<br>TTTTCTGGAAGCAGGAAAGCCTGGTGCT<br>GAGGACACAGGACCCAGGCTGGaggcccag<br>ctctgcctctggaTGACCAAGCTTTGGCAGTGT<br>CTTAAATCTTTGCCTTCAGCTCCTCTCCCT<br>AAACAGTGAAAAAGATGAGTCTCTGGAC<br>CTCCCTgatgggagtccagtggttaagaatccaactgccaat<br>gcaggggacgtgggtttgatccccagtctgggaagaaTCCAC<br>AAACCACGGGGTGACTTAGCCCATGctccat<br>gactactgagcctgtgcgctgcaactacggaagcgcACACA<br>CTCCAGAGCCCCTGCTgtgtgacaagagaagccac<br>cgcaacgagaagcccaggcactgcaaggaagagtagcccgtgt<br>gcagcaatgaaaGACCCCagggcagccataaataaataaat<br>aataaatctttaGTTAAAAAGTCTTAGGTGGGCA<br>TGGTGGGCTCCTCTCCCAGGAGCAGGGT<br>TTGGGAAGCTGGGGGGTCGGTGGCTGAG<br>AAGGCAGCTAGGGCCCTGAAAGCAGAA<br>CAGAGCCCGGGATGGGGCAGGCAGGAG<br>CTGCTCAGAGGCAAGGTCGCACGGGCTG<br>AGTTAACACAGAAATGAGTCAGTGTATCT<br>GTTGGCTTGTGAGGTGCAGTCATCGGGT<br>GTTCGCTGTTACCGTCACCACTGTCCTTT<br>TTGGCCAAAACTTCACAGGAACATTTCC<br>CAGATCCACAGGGCCACGTCTTAAGGTT<br>CTGACCCTTGAGAAACCCCACACGTGTG<br>CTGGGGGCTCCTGTGGACATGAGCTCCT<br>GCACATGGGCATCACGGTGTTCCCTCCCA<br>GGAGCCAACCTCAATCCAGTGCCACTGT<br>ggtttactgagtgcctgctctGCATTAAGTCCTATCCT<br>AGAGAGTGGTGACCAAGCTGTGAACAA<br>GGTAGTTGACAGAAGAATATTCTCATGGA |

| | |
|---|---|
| | GCTGACATCCTGGTCGGAATTAGTGAactta aaaacaaggaaacactTGTGTTTAGGAAAGGCTG TTCATGAATAACATAAAAGATGGTGGGAG AGTGATGAGGGGCCCGGGAAGTGACAG CCATACTGCTTATGAATGAGGAGGTCAGG AACCGTGTGAGGTGGAAGAAACCTTACT CCTGCTCTTCGATCCGTTCTACTGCAGGG GCAAAAGGGAATGTTTTTCCTGAAATCA ACAAGGTGATGTCATGTCAGGGAAAGTG TCTCCCAGGTGGCTGACTAGAGGCGTGT GTCCGTGGTCGTGTCTGCCCCACCTCCTG GACACACCTCTGTCGGCTGAGGGTGACA CAACCCTGTTCCCTGTCACCTCTGTTCCC GCTTATCTCTCCCGCCTTTTCGGCGCCAC CACCTTCTTGGAAATGAGTTGGGCCAAA GGGGAGAGGGCTTCGCATCCCCGCCTCC CCCAGGAGGACCCATAAAAGCAACTGTA ACTGGGCTCCCAGACACCAGAGCAGGTC CTAGACCTAACGGCAGGACGGCCAGACA GACGGCACAATGGCACTGAACACACAGA TCCGGGCCACCTGCCTTCTGCTCCTTGTC CTGCTCAGCCTGACCAGCGGCTCCGTTC TCCCTCCCCAGGTGAGAGCCCACAGGGC CTGGGGCCCCGGAGGGCAGCCGGAATCG CTACGGCCCCTGGGAGACGactgcagagggcag agggagacCCCCAGCATGTGGGCAGAGCTC AGGGCAAGGGAAGGGGGGACAGACAGC TGTTCAGAGCACAGAGATGGGGTACTAA GCGGCAGGGTCTGGGAGCCCTGATCTAA TTCCACAGtgatttactgagtgcctgctcAGCAGTAA GTCCTGTTCTAGACAGCGGTGACCAAGC TGTAATAGTCTCATGGAGCTTGAATATTCT CATGAATATTCTCATACATCCAGGGCAGG AAACCAGCCAATGAACttaaaacaaggaaatatttG TGTTTAGGAAATGCTATTCCATGAATAAC GTAAAAGATGGTGGGAGAGTAATGGTGG GGTTGGCTATTTTGTGAGAAAGTGTTATT TGACCTATTTGGGAGGATAAGAGAAGGG GGCAGCCAGCAGAGACCGAGGTGGGGG CAAGCACGGTGGACCCGAGGAGCAGAG GGCTGGGTGAGTGAAGGAGATGGTAATT GGGGATGAGACCAAGGGGTGGCCGTGCC AAGTCTTACAGGCAAGTGCAGAGGAAGC CCTGCAGGCTTGGGAAAGGAGAAAGC |

| | |
|---|---|
| | TGAGATCTGACTGATAACACGTGGGGAA<br>TGGGTTGTGAGGGGCAAGATACAGGGTG<br>GGGACAGGCCTCCAGGCCATGGAGGGTC<br>CTATAGAAGAGTGAGCAGctgcagagaggggag<br>gagacagaTCTGGGATGTTTGCAGACAGGAG<br>AACCGCctgtgggaaggagaggaaaggtcCGAACGA<br>GAGGCCACTCCTGGGACTCCAGCTGGGC<br>CAGGAGATGCCAACCCCTGGGGAGTCAC<br>TGCTCCGGGGAGCTAGACTGAGAGCTCT<br>GCCAGTGCCCTGCCATCCTCAGTACCCCC<br>TTTTGCTTTTCACAGACACGACAGCTCAC<br>AGACCTCCAAACCAAGGACACAGCTGG<br>AGCGGCAGCTGGCTTGACGGTGAGAGCC<br>CCTGGGACTCCCCTCTGAGCCTCGCCCG<br>CCTTCCCGTGCCTGCGCCCCCTACGCCCA<br>GCTCACTGCCCTTCCTTCCCACAGCCCGT<br>GCTCCAGAGACGGAGACGAGACACCCA<br>CTTTCCCATCTGCATCTTCTGCTGTGGCT<br>GCTGTCGTAAAGGCACATGTGGGATGTG<br>CTGCAGGACATAGACCACCCCCCCAAAC<br>CCCCGCCCTCCGTGCCCTCCTCCCTTATT<br>TATTCCTGCTGCCCTCCAACACTCGATGA<br>ATAGTCTTGAAATAAAATGGCTGGTTCCA<br>GCTCCTGTTTTCCAAACCAGAGTCTCTGT<br>TGTCTTTGCTCCCTGCCCAGGTCTAGGCC<br>AGAGGCTTGTCTTGGGCCCTGTCTCGGA<br>GGGCCAGTCCTCCTCGGTGGGGTGTGCA<br>GGGTCTGTGTGTACTGGGAGGTCGCAAG<br>GAAGCTTGGTGAAGGAtatgttttctcttcttcccctac<br>TTCAAAAGACCTTGAATTTACTTTGCAAC<br>AGCAGGGAGTGAAAGTTCAAGTTTCCTC<br>TTTGGAATCTTCTTAAAAACGGGTAAATT<br>CAGATCCCTAGCAAGGGTTCTCAGAGCA<br>AGATCTGGGGACTCCTCCAGGGCCCCAC<br>AAAGTGGAGACAACTAAAGATGCTACCT<br>ATCTTTCTCACACTCATTCTCTTGAGAAG<br>AGACTGTGGAGCTCTCCAGAGGCTCCAT<br>GACATGTGTTGATCTTATCCTTCTGACAG<br>CTAGCTGATGGTGTGCTCTGTGTCTTGTG<br>CTTTAAAAATGTCCcagttttgggacttccctggtgata<br>cagtggttaagagtctgcccaCCAATCTGGGAGTCT<br>CGGGCTCGATCCTTACTAcaggaactgggatccca<br>catgctgtggggcaactaagcccaggctccacaagaagagaagc<br>cacggcaatgagaagcctgcacatcacaactacagagtagcccc |

| | |
|---|---|
| | cgctggctgcaagtagagaaagctcACTCGTAGCAAC<br>GAAGaaccagcgcagccaaaaaaataataaaagataaaaaat<br>acctAGTTTCAATTTCTAATAGATAAAGCAA<br>CTATAGCGATGACTCACGAGAACAAAAG<br>GTTTTTGAAGTTCTCAACAAGTTTTCAGA<br>GTATAAAGGGGTCctgagaccaaaaagtttgagaagc<br>actgtcctTCAGGGAGCAGACAGGTGCAGTA<br>AATGAGTGAGGCCTGATGGATTTGTTTCA<br>CAGGAGCGTCTTCAGTTGTATGCTCTCAG<br>CTAGGACACGCTTGGCCTAGGACCCATA<br>GGACAATACCATTCACCTCCACAGGGCTT<br>gaatttcctccctttcttcttgaAAACTGCATTCCTCTC<br>AATTTATGTGATTTGCCCACTTTTGATAGA<br>AGAGGAGATGCAAGATGCATTTCATTATC<br>ATCTTAGCCCGTGAGAGCACATCGGCGC<br>TAGCCCGATGATAAAGGGCCTCAGCCAC<br>TTGGCCTCAGAGTCGTGTAAAGATAGGG<br>ACTGCGGAGGCTGGGGGCCCCCTTCAAC<br>AAGGGCTGCCAGGCAGGGCTGCCGCACC<br>CACTGGCAGGCCAAACATCACTAGAGCT<br>ACTTTTCAAAAGAAGGTGGCGATccttattttta<br>tgtgaaatttccTGATTCCTAAAATCTTGGCAAC<br>TGATGAAAggaatccaatttttaaaaaatcaacactcTGG<br>GAGCCAAACTAAACCCAGCTGGGAGCCA<br>GATTGGTTCTGCACACCATCAGCTTCTGC<br>CTTGGAAGCCACAAGAGGCTCGTCGTCT<br>GCCAGAAGTTTTCCTAAACAGAGATGGA<br>AGGACAGGGAGAGGAAGAGCTGTGTGG<br>GGAGCTTGGCCGCACCTCTCAGCTGTGG<br>GATTTCTGAGGCGTCCTTGCCCCACTGTG<br>AAACCCcacctcctcatctgtaaaaggagagAAGCTTC<br>TAGAGTCCTCAGTTCTTTGGACTGTCATG<br>ATGCTCAGCAACAGAAGAGTGTGAAGCG<br>GTTTTGGAAACCATCCAATGCCTCATCAA<br>CGGGAAGGATTCGTACTGACAGGAGGTG<br>GAGGCCTCTCAGACTCTCCCCATAAATAA<br>ATACCCCGGAGGTGTGAAACTCGAAATT<br>GCTGATTGCATTTGATGACACCAGAGCttttt<br>gaaagaatatttatttttatttatttatttggctgcactgggtgttaATT<br>GTGATATGCggaatctttgttgcagcatgtgggatctagctc<br>cctgaccaggaagtgaacctgggccccctgtatcgggagcaaag<br>agtcttagccactggaccaccagggtcaTCCCCACACCA<br>GTGCTCTTCTCTCAGCTTAGCCAGTCCTC<br>CAGGTGGTCCTAGTGATGGGAAGCAGAG |

| | |
|---|---|
| | GTACCCAAACATCAGTGTGCACCCGAAT<br>CTCCCAGAAGGCCTAGTGAAAGGCAGAC<br>TTGGCAAGTCCAGGGTGGGGCCCTGGAA<br>tgagcatttctaacaagctcccagctgATACCGCTGCTG<br>CTGGCCTGGGGGCCACACTTCGAAGACT<br>CCTGTTGTTGGCTGACGGCAGAGAGCGC<br>CTCTGGGTTAGACTAAGCACCTGCTCCCA<br>GTCAAGAGCACGTGGACCAAGGGGGTG<br>CAAGGGCTTGATTCTGTCCTAAGATGAGT<br>TTTGCTCAGTCTGTACAGTGTTTTAGGTA<br>AAAATTGGGCAAATACCTAAAGACTGAG<br>ATATTTCACATAGAAATCTGAACTCTTGC<br>TGTTACAGACTGAACTAGGTCCGTCTGA<br>AATTTATATgctggggtttccctggcagggctttcctggtgg<br>ctcagatgatacagagaatctgcctgccatgcgggagacctgggt<br>tcaatccctgggttgggaagatcccctggagaagggaatagctac<br>ccactccagtattctggcctggagaattccatggactgtatagtcca<br>tggggtcgcaaagagtcggacacgactgagtgactttcactctttG<br>GCAGTCTGGTAGTTAGAACTcaattcagtgctttc<br>actgctggggcctgggttcaacccttggttgggaaactaagatctt<br>gcaagccatgtggccaataaataaattaattaattaaatgtaaatgtt<br>gGCATCTTAACCCCCAATGCAATTCTGTG<br>ACCTTATTTGGTGAtaaggtctctcttttttttttttttttctttt<br>gattgctTGGCTCaatatgtggggtcttagttcctgcaccagg<br>gatcaatcttaatcactagaccatcagggatgTCCTTTGAT<br>AGAGTCTTTACAAGGATAATCacattaaaatgag<br>gtcattaggtgGGTCCTAATCCAGTAGCTTTGT<br>TGTGCTAATAAAAAgtggaaatttggacacagagacc<br>cACATATAGGAGAACACCATGTGGATGTG<br>GAGACCTCTAGTCTCCAGCATCATGAGaca<br>atttgttgctgttgttcagtgctaagttgtgtctgattctggcttccctct<br>ccttcactatcttctggagtttgctcaaactcatgtccactgagttagt<br>gatgccatgcagtcatctcatcctctgttatccccttctcatcctgccc<br>aatctttcccagaatccagGTCTTTTCCCGTGAGAT<br>GGCTCTTTAACAtcaatggccaaagtattggagcttcag<br>catcagtccttccaatgatcacccaggactgatcttctttaggatgg<br>actggttggatctccttgcagtccaagggactctcaagaagtcttct<br>ccaacacacacagttcaaaagcatcagtctttTGACgattagccttc<br>tttatggtccaactctcacatccgtacatgactactggtgaAACC<br>ATAGCCAAGAGACAATAAACTCTATTTTT<br>TAAGCCACTTGATTTAAGAGGCTTTGTCA<br>CAATCACCCTAGCAAACCTGCCTTTCCTG<br>GAAAGGCCTGGTGATTGGGCAGCCCTGG<br>TAGATGGATCTGAGAGGCACCTCACCTTT |

| | |
|---|---|
| | CTTCGGGCACAGGCTCTCAGGTCCCTGA<br>GTCCTCCAGTGGGCTGGGGACCCGGGTG<br>CAGATCTCAGCAATAGCACCCCCTGTGG<br>GACACACTCACCTGTGCAGCCCGCCCCC<br>AGGGACAGCCTTCGGTTCACCCCCAGAG<br>AGATGGTGGACCCCTCAAACTAGACACA<br>CAACTCCAACGCGGCTGGTGTTGATGTG<br>GAAGGCTGTGACATCTGAGTCCTCCAAG<br>ATCTCCGTGTCCTCCCCCAAAGCCCACGC<br>ACCAGGTCCCTCTCTTCTCTGGGACCCA<br>GGCGTACAGGCCCCACCCCAATCTCAAT<br>GGGCCTTTGTCCCTGCCTGGGAAAGGCA<br>CGGGCAGAGTCTGACATCATGAAgcctgagg<br>tgggggtgggactCCTCATCCCCCaccgcccgcccccc<br>acccccccgcccgccCCTAGGCTTCTCTGACTCCT<br>CACTCCCCAGTCTGGGGATTTCCCCGCCT<br>CGACCCATTCCTGTGCCTCAGGGCGGGG<br>TTCTCCCTTGGGAGAAAgggccctctctccctccct<br>tcctccccactgctCTGGCCTGAAGGACAGAAG<br>ATGGCCTAGTGTGGCTGAGCAGCTCACG<br>ATCAAGCCACTACTACGAGGAGgctgagatga<br>cagaggatgagatgattggatggcatcacagactcactggacatg<br>agtttgggtaaactccaggagtcgatgatgaacagggaggactgg<br>cgtgctgcagtccatggggtatcaaagagtcagacatgactgagt<br>aactgaactgaactgaacacttagtCACACCACCACCA<br>GGCAAAGGTTATGACTGGGCCTGGGGCA<br>GCAGGAGGATCACCATAGAATCTGCACA<br>CAGAGGGTCCCCACCCAGCTCAGAGAGT<br>CAGCACAGCCCGGGTTGGGCACCCTCGtg<br>caccaggcactgtgctagggaACACGGGggtgaacaaga<br>cagacagacaggcggACAGACGGAAGCCTGAG<br>CTCAGATACCAGTGGGGAAGCAGCTCAC<br>TGAGGGTGCAGGTGGGCAAATGAGGTGC<br>TGAGGGCCATGGAAGGACCGGgcagtatttaaa<br>tattttatttatggttgtgctgggtcttcactgctgctcgcgggctttct<br>ctacttgaggtgagtgggggctactcttcattgtggaaTGAGG<br>GCTTCTCCTtgccagtggcttctcttattgcagagcatgggc<br>tctagaacacaagctcagtagttgtggtacaaggATGTGGG<br>TGCTCCgtgacgtgtgggatcttcctggaccagagatcgaac<br>ccatgtttcctgtgttggcaagcagattcttttccactaagtcatgag<br>ggaagcccctggggcagCTTTTAAACAGCGGGGC<br>TCGGCTTGGCGGAGAGGGGCTGGTCAGC<br>AGCAGATCAGGAAGTGGAATGGGGCAG<br>GGGAGTTTCCAGGAGGAAGGAACAGTG |

| | |
|---|---|
| | CCAAGGCCTCCAGTGAGGGTATTTGAGG AACACACTCGTGCACCCACTCTGTGGTT AGGAAGTTTAACTCAGGAGTCCGTCTGC CTGCAGGGCAGATTGGGAGGAAGCAAA GCCCTGTCCTCCTGAAGGTCATGTTCTCA CGGGAGGAAACAGTTAACACATAAAGTC ATTTCCAATGGCAAGAAGAGCCTGGAAG AGAATAGACCAGGGTGTGGGCACCAAAG ACGACACCCTCTAGGGAGGATGGAGGGG ACTAAAGCCGAGagtgagctctagagcctgcctCCT TCTGGTCACACTGGCCTCTGTGGCCTCCC GGGCCTAAGGTTATTACTAGCATTGCCAC CAAGATTCCCCAGAAGCTGAGGACAACA CGTTCTGCCTCTCAGCCTGAGACTTCCAG ATATGTTAGAGACGCACCcccgccctcctcccagg gacccAGAACTAGGGTGCGCAGGGATGGA GCGGGGAACGAGTGAGGcagccacccccagccc ctccaggaAGGAGCTCGTGGAACCCAGGCGT CAGGCTGCCCAGTTCGCTCTGTGGTGAC AAGGGCCCTTTGTGCCCCCCTCCCCcgggg gcagggaggagctggGCCCTGGAGGCAGGCGG CCCCTGGCAcccggggggaggggaggggctggcgaG TGGGGGCCTAGACCCTGGGAGGCCAGGG GACGGCGAGCAGGGCCGGTGGAGCAGA GGGTGCAGAAGCAAGGGCATCAAGTGA GTACTGCAGCTGAGAGGGCTGGGGACGG GGAGGAGAGCAGAGGACCTGGGTTGAG AGCACAGGGCAGGGTCACCCAGGGCGC GTGCAACGCAGGGCGTTCTGGGCCGTGG ATGGTGGCTGTGAGCTGCCACTAATTTAG GATGGGGGAGGGTGCTGCTCAGGACCGC GCTGGGCGTGGGCTGGGATCATGGCACG GACACTGACTGAGCTTTGGGAGCAaaccac agagagagagacagaatgagGCACCCTCCTTGGGA CCCCTCCGGTGGAGATTCTGCCCTGGGG CTCCTAGGTTTGTGGCAGTTGGGGTGGA AGTGGTCCAGGGCCAGGAGAAGACCGA AAGGATGCCCAGGTAATCAGAGGTCTGT ATCCTGCCATctgggaggggatgggggtgggggggttg agTGGGCAAGTTCCAGCACTGAGCACAC GacaggggtgtgtgtgcacaggcgCGTGCACCTCTC TGAGTGAGCCTTAATCCACGTcagtgtttctccat cttttttttaacatctcaCTAAGGAAACCTTTCAGAC TTGCTTTTCCTAAACCCACTTCCTCCATG |

| | |
|---|---|
| | AAATCTTAAGATAATAGATGGactgtgtttatgtac cacacaGCCCTTTAGAGAGCCACAAACCAT CATAATAACTCAGGCTTTTCTGGACCCCA TCCCCAAGAACCAGTTTTCATCCTTATTC AGAATGCATGATCTACACGCTTGGTTCAT GAGTCTGGGCCCGTGGCCCTCACCTGTG TCTACCCGCCGTACCTCCCCGCGGCAGG CACAAAGACGGCATCTGCCGTGTGTTCT TCGGCATGGGTCTGGGTGGGTCTGGCCA CAGCCACACCTCCATGTGTGTGCTGGTCC GAGTGCCTTTGGGAGTGTGCAGTGTGCA GTGTGTGTGCCTGATGGCCATGCCTGTAG CTGTGCAGGGGTGTGGTGGGCAGGAGG GTGAGGTGTGTGTGGGTATCTGCTGCAC GTGGGTCCCGGTGCCTGTTGGTGTGTGC AGGGTCCTGAGCAGTGCTGTTGGGGTGA GCCGCCTCCTTCTGGTTTTGTCCAGATCT GTAACATTTCATGCCAGCATTTGCATGTG TCTGACTACATGCATCTGTCCGTGGGAAG ACAGTGTCAGAGGCCAAAAAAGCAGCC ACTTGGAAACTCTGGCTCCAACCCGAGG CGACCCACGGAGCAGCCGGGTGCCAGG GACTCACTAACCCCAGTCCCTGGTCCTTC CTGGCTCCACTCAGAGCCCGGCCAGGCG TTCCCAGCTCTTCTCTCGACCCCACCTAC CTCAGAAGCCCCCCAGGACGAGGCGCCC TCACCCTGAGGGAACAGGCCTCTTTTGT AACCGGGCTCTGGCCTCTGGAGGGTGAG GACACCAGGGGCCATCTGTGGGGCGGGA GAGGGCGGCAGGAATTCCCGCGGCATGT TGGGAATGCTGATGCTGTGAAACCCAGT CATTGATGGGGCTGGGTCCCTGCCTGCCA CCCCCAGGTCTCAGCCATGGGACAAGTG CTCCCTTGCCCCATTCACACCCCTCGCCT CCCCCTACCCCAAGGCACTGCTGCTCCC CTTTGCTTCCCTGGAGTTCTCCATCATGG CTTGCCACGGGAGGTGACCACAGAGTAG CTTTGAGGCTGTGTGGGAGGGTCTCAAA CATCCAGAGGCCATGGGCAACAGAGGCT GTGTGGGCAGCCTGGCAACGGCCACGAC TCGTGCTGGATCCTGTGAACGGGGGTGG GGATGTTCGGGCCCCCACTTCCTTGCACC TGTTTGATTCTTGCTCCAGGTTGGCCGGC TGACAGCTGCAAGCGGGACCAGAAGgcatt |

| | |
|---|---|
| | cccaactcagggagaCGGAGGTGAGCGGGGAG<br>GGTTCCAACTTCCCTTCCTCCTGGCTTTT<br>CCTGCTTCCTCTTGTTCATCCAAACTTGG<br>CTCTATCCTTACTTTCTGGAGATCCCCAA<br>GGGCATAATGGTGATAAAGATAATTATTA<br>GCACGTCACTAACCATGTAGATATcttcaccgg<br>ctcaatggacgtgaatctgaatagtgaaggacagggaagcccag<br>cgtgctgcagtccattgggttgcaaagagttggacgtgactgagc<br>aactgaataacaacaaacccACGGAGAGTCAGGAC<br>AGTTTGGTGGTGAAGTCTACAACCCTGG<br>ACAGGCAGACAGGGCTTGAATCCTAGCT<br>CTGTtgcttgctggctgtgtgaccttgggcaagttactcaatcT<br>TGCTGGAGCTCAGCTACAAAAACAGGAT<br>AATAATTGCACCTACCTCAAATGAATTTG<br>TATAGTAGAGTACCTCGGGTGGTACATGG<br>TTGATGCCATAGATGTGTCTGTGATCATA<br>AATactaacagcagcagcagctatcatCTGAACGGGC<br>TGCTTCCCAAAATCTTACCAGAAgtcttactttt<br>cacttctctgggccttcaacactctcctctcccctctccagctCTG<br>CTCACTTGCTGTACAAAATGATATTCCTC<br>ACGGCACTGTCTCTATTTTGGATTATGATT<br>TCAGGTAATAATGCATGGGGATCTGGAGG<br>TTTGTGGGTCCTGGCCCCTGAGCAGGCT<br>GGGGTCCCTGGACCCCTCAGGTGGTGGG<br>AGGTGATGagagtgggggttgggggcacCCGGGCA<br>GAATGTGTCTCTCAGCCTCGGCGGTCCTG<br>CTCACCCACAGCCTCTCGTGGGGGTCAG<br>TGGGGCGCCTGGATGCCATCGTCCATCTC<br>GGCCTTTGAGGGCACGTGCGTCTCCATC<br>CCCTGCCGCTTCAGCTTCCCTGATGAGCT<br>GCGGCCGGCCGTCGTGCATGGCGTTTGG<br>TACTTCAATAGCCCCTATCCGAAAAACTA<br>CCCTCCGGTGGTCTTCAAGTCACGCACG<br>CAGGTGGTGCACGAGAGCTTCCAGGGAC<br>GCAGCCGCCTCCTGGGGGACCTGGGCCT<br>GCGCAACTGCACCCTCCTGCTCAGCAAC<br>CTAAGCCCCGAGCTGGGCGGCAAATACT<br>ACTTTCGAGGGGACCTGGGGGGCTACAA<br>CCAGTACACCTTCTCCGAGCACAGCGTC<br>CTGGACATCATCAGTGAGTCTTGGGGGG<br>CTGTGCCGGGGGCGGGGACCATGGGAAA<br>CAGGAAGCCCTCCCAGTGCCCTTCTGGG<br>AAGGTCTGGTCGGCAGAACCAAGGACTT<br>GCTGGTTTGGCTGGAGGGAGGGGTGCCA |

| | |
|---|---|
| | AGCCTCTGGCCCACATGACCAGGCAGATGACAGAAAGGGGCAAAGCAGGGCCCCAAGTTGTATTGTCCTCCCCAGAGGAAAGCAGTGGCTCTTACTCTTCAGAGACACCCGGGAGAAAGACAGTGGGGCTCGGGGATTGCGGTAAGGTCTCTGCATCCCTGTATTAGCTGGGCTACTGTAGGCAGTTTGCTGAAATTCTCTGGGCCTTGTtatcctcttctgtaaaatggggataagaacAGCATTTAAAGTTCAATAAGGGAATAGTTGTCAAGTGCtaaaaacagtgcctggcacatggagcCTGGCTCGAGCTAAATGGGGTAGCATGAGAATGCAGGGCCATAGATCTCAGGCAGtctgagtttttgttgttttgttttgttttgagccaTGCCAAGTGGCAtgggggatcctagttccctgactagggatagaacccgtatccccctgcagtggaagcttggagtcctaaccactgaatggCCAGGAAATTCTCTGAATTTTCAAGGGAAACCAGAACTGTGTGTTTTCAGCATAGGCTGctacttcagattttttttttaatgtcttaagtTTATGCCAAATAAAATGCAGCTCTGGGCCAGTTTGGGCAGAGTAGGGTCCACTGCCTCATTTCTGAGAGGCCCTGAGAGGCATGGAATCCTTAGATTCAGGTTCCCCATCAGCTCTGAGACCATATGAGGAGCCAGATTATAGCCCTGA |
| AC_000176.1:39742140\|39768649 | GCGGACTCtgcaccactggaccaccagggaagtccttctgtcTGTTTTCTTTAATGTCGATTTTTTTCACATTGGGTTTCCTTGGATCATTTTACTGGACAAAAGATACTCTGCTGGAAAAGATGTTTCAGAAAACCATGAATTTGGGCAGGAAAGTCCTATGAGTCACATACTCTGAAGCTGTATGTGAAGACATAAGACAGTCTTTCTGCCCTTGGGAAGGAGCTCGGGAGACAAGATGAGCACATGTGAAATAGTGGACAAGAGAAAAGGCTGGTTCTCATTAGGGGCCAGATTGTGTATCCCAGAGAGGGATCTGAGTGTCAGAACAGAGAGTGGACCCACCTCTTGGGCTTGGCAGTGGGGAGGGTGAGGCCCAGGGCCGAGGACAGAGGGCTTTTGGTGGcttcactaaagcctttgaccttgAGCTAATGGACAGTGGGGAGCCTTGGATGGTTCTTCAGTTGGGGAGGCATAGATGAGAGAAGGGTTTGAGAAGCAGGGCTTTAGCGTATGCCTaataggtgtgtgggcttctctggtggctcagctggtgaagaatccgcctgcaatgtgggagaccctggtttgatccctgggttgggaagattcccctggagaagagaagggctacccactccagtattctggcctggagaattccatggactat |

| | |
|---|---|
| | atagtccatggggtcgcaaagagctggacacaactaaggaccttt<br>cacttcacttgagtGGATGTAAACTAAGGAGGCC<br>CAGAGTAGGATGTCTAGGGAAGAAATTA<br>CCCATCTTGTGGGAGAGGGGGTCAGGAG<br>GACTCAGTGGTCCCAGGGGGATGAGGAG<br>GGTGGGAGCAGCTCTAGGACTTGGCTGC<br>AcgagagggaagaaggaggggtAGGAAAGGAGCC<br>AGAAGTTTGAGCTGCGGACTGGTGGTAT<br>CTGTCCCTGGCAGACCTGAGGTGTAGGA<br>CAGGAAAGAGGATGTCATGGAGCAACCC<br>GTTGGACTGGGCTTGTCGATGGAAATTC<br>AATTCACTATcagttaagaagaaaaaagtgttGAGCT<br>ATGGACCCATTGTAATGAGTACTATATTGC<br>TGAGTTGTGTGTCTTTGCTCCAGGATTTT<br>GTTGCTTTCTGAACATGTGTTATTCTGAG<br>CAGTTCCTATCCAGGAAGGACTTTGCATT<br>GGGTGTTGGCGCCCCCTGGTGCACAGAA<br>GGGACACTGACCTGGCTATTTTTCATTCT<br>TGTGTTCAACAACCATTTGTTTAGCCCAG<br>TTTCTCAACCTTGACACTTCGGCAGAATA<br>ACTCTTTTCTTGTGGGAGGTTGTCTTGTG<br>CACTGGAGGAAGTTTAGCAGCACCAGGC<br>TTCTGTCCGCTCGATGCCAGTGGCGTGCC<br>CCAGGTGTGAAAACCAAACAGTTCCAAC<br>GTCCCTTCTTGAGAATTCGTGATTTAGAC<br>CAGCAGGAGTTGGCACCAGCAGGTCCAA<br>CGGGCAGGCTCATGTGGGGGTGGGTTTT<br>GCTCACTTCTGTCCCCCACAGTGCCTGCA<br>CCAGGGAAGGGATGTTCTTTCTGTGGAG<br>GGACAAATGGCTGGCTGGAGCAGGGCAC<br>TGGGGCTAGGGGCTCAAGGGGCTTGGAC<br>CTGGCTGGGGACTCTGTTCTGCTGTAGTG<br>GGTATTCTTAGCCCAGTGACCCTTGGTCT<br>GGGCAGAGCTGGGTATGGTATCAGTAGAT<br>CCCAGAGAAGGCCAGAACCGGGCTGGA<br>CTGGGCTCTAGGGCTGGACTAGCATGGA<br>GGCGTGGGCACCGGGGCGGGGCCTGACT<br>ACAGCCTTATGTCTTTGCCCCCTCCCCAG<br>CGCTGGCAAGACCTCAACGTGATCAGCA<br>GCCTGCTCAAGTCCTTCTTCCGAAAGCTA<br>CCTGAGCCTCTTTTCACTGATGGTGAGTT<br>GGGGGCGGACGTGGAGGATGGAAGGGG<br>GAGGGAGTACCTATCTATGCTGCATCCTG<br>ACTGCTTTTTAAATTGAGTGTGTGGTTTT |

| | |
|---|---|
| | ACTTTGGGGGTTTAATACAAAAGAAACA<br>CATGCTTGTAAAAAACTTCATCAGTACCG<br>ATATAAATAAAGTGAACACAGTCATCTTC<br>CCGTTTCCTGTCCACAGAGGTTATCCTAA<br>CATTTTCTGGGCATTTaccgtgtgccaggcactactC<br>CCTACAAATCCATGAGGAGGTACTATTAT<br>TTCCACCCCATAGATGAGGGAGCTgctgcac<br>agagaggttaagtgacttgcccgaggtcacagaGGTTGTA<br>AATGTCAGAGCCAGAATTCAACCCCAGG<br>CATCTGTCCCCAGAGCCCAGCTCTTGGCC<br>GTTTGCTGAGCTCCTTGTTAACAGTGCAC<br>TGTGTTTGCTTCCAGACCCTGTTACACGG<br>GGTttctcttgtgactcagctgataaagaatcctcctgcaatgtgg<br>gagacctgggttcgatccctgggttgggaagatctcctggaaaag<br>ggaaaggctacccactccagtcttctggcctgggtcgcaaagagt<br>tggacacgactgagcgactttcactttccttttcctctcacaCGT<br>GTGACAAAGACACACGTGCCTACATAGG<br>GCTTGCAGTTGGGTGATGCTGGTAGCTGT<br>TTCATCTGGCTCTGAAAGGAATAAAGCTG<br>CGATGATAGCACTTTCCAGTTTCTACAGT<br>ATAGATACTCCTGCCATGGCTGGCTTCAG<br>GATCCCAATCTGCTGTCACCGACATGAGC<br>TGGGACAAGACAGACACATCCAGTTGTC<br>AGGAGTCCTGTGAGCGAGTGGCTCCGCT<br>GCTTGCGTGGTTCTCAGCAGAAGGAGGC<br>TCGTtaattttgagattcattcatgtcaGTACAAAGAGC<br>TCGaccttgttttccttttaaatcaaCCACATAGTGTTT<br>CCCAGCCTGAATAGACAGTAAATTGTCTA<br>ATCACtccccttgttgttgtttagttggtgagtcgtgtctgactgtt<br>tggtaccccatggactgtagcccaccaagctcctctgtccttggga<br>tttcccagccaaggattctgcagtgggttgctatttccttctccaggg<br>gatcttcccaacccagggatcgaacccgtgtctcctgcttggcagg<br>cagattctttaccactgagccacctgagaagctcaatCACTCT<br>CCTactgatggatatttagattgcCTTCACATTTTTGC<br>TATTTCAATACACGTTATAATGATTTTTCT<br>TGACTGTGTCCCTGCATGCCTGGACCAG<br>GATCCCAGAGGTAGATTCTTAGACATGGA<br>CTCGCTGGGTCAGAGTCCTGGAAAGCAT<br>TTTTGAAAGCTAAAGTCAAGTTGCATGCC<br>GAGGAGCTGACCCAAGCTCACACTGCCG<br>CTGGTGGTGTAAGATACTTTCCCACGTCC<br>CCATCACACTGgatgatgttattaatattttaaaattttgctg<br>atCAGAGAGGGGAAATGGCATCTcactgttgtttt<br>cattttcacttctgtcATTACTAGGAGATTAACCTT |

| | |
|---|---|
| | TAATGGATTCTGTGCTGAGATACATACGT AAACATGAGTGTTTAggaatacctttatgattataaaaa tattttcttatttttcccaaTACTTGAATAAGTATTATT TTTGACTCTGTAATGCAGTTAGATTTGTTT GTGTATTACATGAGctagaaatggatttattttccCGA CAAGATAGCTAATAGTCCGCAGGACATTA TCGACACCACCATCCTTCCCCACTgacttgaa gatcccctggagaagggcatggcaacccactccagtgttcttgcc aggaaaatcccatggacagaggagcctggtgggctgcagtccag agggtcgcgaagagtcggacacactttTTCCTGTAGATC CACAGGGCCTGTCTCCCTCCTTTCAGTTC TGACCCTTCTTCCCCTTTCCATCAGCGTC ACGTTGCCTGAGCTGTACGATGGTTTTAG TCCGTGTCAGTTTCTCACTGGCAGGTCCT CCCTCATCATTCTTCCCTTGGAGAATATTC TTGGCTGTCTTTGCACATTCTCTCATCCA GGTGTATTTCAGGATGAACTTACCACGTT CCCTTTTTTAAAGTTCCCCGTGGACTTCTt ctgaggggaggtgggggaaccCTGATACAGATGGG GAGGAGTGTTTGGAGGCTGAAAGGGCCC TCCCTCTTTCTAGGTAACCTGTCTTCTAA CCTCGTGGTGTCCTTGTCCTGGAGCCTGG GGCAGGgaccccttctccagggtcaaCAGGCAGCT GAGGTCTCACTTCTTCCCAAACGAGCAcg cctgggcttctccaggtcaGGCGCACCCCTGAGGG CAGCTGCGGTGGGTTTGGTCACATGAAC TTAGGGCCctggagccccccctcccaccaacTGAACA ACCTCAGCACAAACATGACCTCCCCGCA GGGCCTCCTCAGCCCGGAAGCCCCCCTC TCTAAAGAATCCTCACTGTTCTAGACAAA TACAACGACTTCATCGAGGCCAATCGCAT TGAAGACTCGAGGGAGCGCTTGAAGAC GCTGCGGAAGCTGGTAAGGAGGGGGCTG CTGCTGGGCGAAGGGCAGACTGGACAG GCGAGCGCTGCGGCCCCAGCAGCCCAAG CCCAGTGCCACCAGCTTCCCCCGTGACC TCGGCAAGCCCTTCTCTCCTCAGGGCCTC AGTTCCTCTGTCTATCCCCGGGGGACAGT TAGAGAGTGGGTTCCAGACCCCCTGAGA TATGGCAGCGGAGCCCCTTTGAGGGGCA GTGGGGAGGTGGAAGCCATCACAGCCTG GTCCCatgccccttccttctcccacccAGATCCGGGA TCTCCCAGGACACTACTATGAAACACTCA AATTCCTCGTGAGTCATCTGAAGACCATT |

| | |
|---|---|
| | GCCGACCACTCAGAGAAAAACAAGGTG<br>GGTCCAGCTCCAGTTTGAAGTGTGGGGG<br>AGGGAAGCCTAGTGGGGTCTCCTCAGCA<br>TATTCTATTCAGTAAGCTCCTCCAGGTCA<br>GGTAACCTCAGGCTTCACTGGCCCCGCC<br>TTCCGACCTAGGGGGTCCTTCTGTCCCGC<br>TGCCCCCACATACTGGCCAGTACTTCTTC<br>CTGAGCCGGCTCCTGAATTTCTGAGGGT<br>CTTCAGAGGCGGAGAGCCTCAGACTCTG<br>TGTCTGGTGGAATCTACCCTCTGACTGAG<br>CTTGGGTCCCTTGCGTTGCGTTTTCGTAA<br>GCAAACCCCTATATCTTCTAGTTCCGCAG<br>GGGGGTAGAATGAGGGGACATACAGGAT<br>TCCTGCCAGTCCAAAACACCCTTGGGAC<br>GTCCAGTGGGTCTTCACTCTGGCCTCTCC<br>CATTGCTAGACAGTACGGGTGTGCAGGT<br>GGGGAACTGCCCGTGAGAGCTGGGCCTG<br>GGGTTGAGTGGGGCCTATTCTCTACTTAC<br>CAGGCACCTCCATGCCCAGCAAGGAGGC<br>ACATGGCCCCAGGCCAGGACCAGCTCAG<br>GATGTGGTCGGGCTGGGGGCCAGAGGGG<br>CCCAGAGTCAGGGAGGCCCAGGGGCCT<br>GGAGGTCAAAGCTCACAGGGAGACTAAC<br>ACTGATCCGCTTCCCTCCCCTGCCCAGAT<br>GGAGCCCCGGAACTTGGCCCTGGTCTTC<br>GGGCCGACGCTGGTGAGGACGTCTGAGG<br>ACAACATGGCAGATATGGTGACCCACAT<br>GCCTGACCGCTACAAGATTGTAGAGACG<br>CTGATCCAGCACGTAAGTGGCCTCCCTG<br>GGCCCTGGCTCCTGCTCGCCGGGGTGGG<br>TGTGCCCAGGGAAAGATGTGGAAAATGC<br>TCCGGGGGTGCAGGAGGCCCTTCAGAAG<br>CAGTAGCCTGCATTTTAAGGCCAGGCAG<br>GTATGAATCCTGAATGCCTGTCCCCAGCG<br>GGACAACCAGGAGCCGCTGAGTTTCCAG<br>GATGTGTGTGTGGgacagagggaggagaaaggTC<br>AGGGAGAGCTGCTGCTGGGTTGGAGGGC<br>AGAAGGGTTGAAAGAAGCGGGGTGCCT<br>TCAGAAGGTGACCCACGGGAGAGATGTC<br>CAAGCAGAACTGGGCAGACAGATGGGA<br>CAGCACTCTCCCTGCAGGCCCACTGAGA<br>CTTTAAGGGAAAAATGGTGATTCCCTGG<br>GGAATGGGAGGCAGATGGGGCAGGGCG<br>GATGATGGGCTGGGGCAGGATCTGGCTG |

| | |
|---|---|
| | CGTGGTCTCCGTGTCTTGGGCTGGTCACC<br>TTCCTCCTTAAGGCTGCCTGGAGGCCCC<br>ACAAGAGCCTGTGGTTTCTAAGGAGTCT<br>TATTATCCACCACAGGATGAGAACCCAGA<br>ATGGCCAGCAGAAGGCGCaggaggctttttatttttt<br>tttctcttttttttaagcgCCAAACCCAGGGCAGCAC<br>TGCCCCCTTCTAGGGGCACTCAGAGATGT<br>TTTTATGAATCATTGATTTTTCCATTTCCA<br>AATCTTGGTGGGACCTGTGGTTTAACCCA<br>CAAAGAATGGAATCAAAACCCTCCTGCC<br>AGGAGAAATAAGATGGGCAACTGGTCAA<br>AAAAAGggtttgcttccctggtggctcagtagtaaagaatcc<br>acctgccaatgcaggagatgtggtttcgacTCctcggttaggaa<br>gatcccctgcagtaggaaacggcaactcatAGACACTTT<br>AGGGAAGGGAATAAGCCAGGTCATGTTA<br>CtcctgttacagatgaggaaactggggctcagagaggaaaAG<br>CGATTtcttcaagatcacacagctattaaGTAGCAGCC<br>TCAGGATTTGAAGGCAGGTCTGTCTGAC<br>CAACTCCTCTGCTTTACTCCCCACTGAAT<br>GGGAGGGCCGACTCCTGGGTCCACCCTA<br>GAGCTGGCTCTTGAGTGAGCGAGGTGGT<br>CAGAGTTAGCCGAGTCAGGCTGTGGGTA<br>GACTTTGGTCATGCCGGCTGGCCTTGCctg<br>ctcctttccttccttcactgGGTGTCAGAGCTCTGAG<br>CCATAGGAGGGTATGTCGAGGCCTGTTCT<br>CACTCTAACATTGGGACCACCAGCCCGG<br>AGGCCCACTCCAGAGCTGCTGGGGAGGC<br>CAAGGCTGCTGGGCAGTGTCAGAGATCC<br>TGATGAGGCCTTGGTATGGGGAAGCTTTC<br>CTCCCTTGGAAAAGTTCAGCTCCCCAAA<br>CGAGGAACCCATGGAGCCCCCATGAGTG<br>TATGCTTGGTGGGGAAGGTagtgaggaagagag<br>ggagactgGCCCCCTGACTCCTAGCGAAGTC<br>CAAAGAAGCCCAAAGCCTTGGTCAGCTG<br>GAGAGTCAGGACCTTCagggagatgggaggccca<br>aAGTGTGTCCACTTCCCCTACCCTAGGGT<br>CTCAGGGGGACATCCCCAGCCAGTGGCA<br>GCATCTTTTCTGGGTAGGGTGTGGGAGGT<br>GGAGTACATTGGTCTCCTTCATAGGTGTA<br>ACCCCAGCTCTGACCCTGTGCCTGGCATA<br>GAGTGAACCCTCAgtattggttgaatgaatgaatgaatg<br>aatgatttgttGTTGAGGTTATGCCAGTCTGGGC<br>ATTCCCACTGAGGAAATCATTTCTGGGGA<br>ATGACCAAACATGCACCTCTCCATTTTCT |

| | |
|---|---|
| | GCCCCGTTGGCATTAAAGTAATCAGCCCT<br>GGATGATTGTCAATTGTCAAGAAAGTGA<br>GCTGCCTGTTCATTCACACTGAGCCCACT<br>GAGCCAACCCACCCAAGTTGGCCAGTCT<br>GTCTAGCACAGAGACTCTTGAGCCAGGG<br>TAATCCCAGTGTAGTCCCGAGCCCAGGC<br>AGGGCAGAATTTGTGGCAGCAGTTGCTC<br>TGGCTCTGAGCCCCTGGCCAGTGAGTGG<br>GTGGTTGGAATTCGTGGGTGACATGACC<br>TCCCGTCCCACTGGGAGGTTCAGCTGTG<br>AccacccctgcctccctgcccggGGGTTCTGTGTTC<br>CATGGAATTTTTGGACAGCAGAGCTGGA<br>AGGGGCCTTAAGTTATGAGACCTGACCTT<br>GCATTGTACAGAGAGTAGAAACCGAGGC<br>TTGAAGAGGAATGGTGACTTGCTCAGGG<br>TCCCACAGCAAGATATGGCCGAGTTATAA<br>CCAAACCTAAGTGTTCAGCCACCCGGCA<br>GCAAGTCACCAGCCAGCTCATACTCATG<br>ATTACTAAGCTAGCTAAAACCGAAACAA<br>AGACCATGCTGaggcatttgttcatttattcactcagcaaA<br>CGTGGTGATTCCTACTGTGCGTGCACTGG<br>AACTTCTCAACCCGGCACTAGTGACCTTT<br>GGGGCCAGACCATTGTTTGTTGGGGGCT<br>GTCCTGTGGCTGATAATATGTTTCGTGGC<br>ATCTCTTGGCCAGATGCCAGGAGGCTCC<br>CTCCACTTGTGACAATTGAAAGTGTCTCC<br>AGATGTCACCAAATGCCCCCTTGGAGGG<br>GCACAGTCACCCCAGTTGAGAAGTACTG<br>ATGCAGGGGCTCAGGATACTGGCCCTGG<br>AGCCAGTCTGCCTGAGGTTGCACCCTGG<br>TTCTGCCCCTTATATGCTGTGGGGCCTTG<br>GACAAGTTAGTTACCTTttttgtgcctctgtttcctcac<br>ctgtttgTCTCGCAGTGTTATTTTGAGGGTTG<br>CATGAGTTATTATCTGTAAGGCActtagaatggt<br>gcctggcaGGTAGTAAGTGTTATACAAGGGT<br>TAGCTTATTGATCACCAttgccatcatcaccaccattg<br>tCATTGTTCCATACCAGATACAAATGCAGG<br>TGCTGGCAGCACAGAGGTGAATGGAGCT<br>CAGACCCTGCCCTGAAGGCACGCTGCCC<br>AGTGAAGGCAGCACTTCTGGGCTATATCC<br>AGGGAAGCCACATGGAAggacccagcccagccc<br>tgggtgCAGGGCTGCAACCAGAGTCAGGGA<br>GTTCCTGAAAGGCACTAGGGTTagccaggca<br>gagaagggagaatgagcattccaggcagagggaacggcATG |

| | |
|---|---|
| | TGCAAAAGCGGGTGGCCTGAGCTAGTGT GAATTCTTCCAGAGAACCACGGGCCATC CGGAAGCTTCCCGTAGACGTCCAGAAGC TTCCTGTAGCCTTCCGGGTTCTCAGAGCA CTTTCAGGTACTCTTAAACCTGAGTTAGA GGCTGGGTTTTTAGGGCTCCTTCTAGACA GTCTGTCTCTAGGAACTGCACCTTTtagtaat agctaacatttattatacACTATTTGTTGtactaaatgcttta catacagtatttctttttcgcttttttaaaaaattgagatgtattgacatat tagtttcaagtatacaaaatGATGGTTCAATATTTGTA TAGATTGTGAAATGATCAGCACCAAAAG TCTAGCTAATGTCCAACACCATACatagaaaa tttttctttgatgaagccttttaagatctactctcttagcatcTTTG AAATATACGATGCAGTGTTATTAGCTGTA GTCACCATGATTACATCCTCAcgacttatttattca ttttacaatTGGAAGTTTATATCTTTTGACCACC TTTGCCCATTTCAGTCACCTCCCCATCCC TTTTCCCCTGCAGCCAACAATCAGTTGTC TGTATCTATGAGCCTggattatttcttttcaaatcccacat gtaagtgagatcttacagtatttgcctttctctgcctgactttgaatttc acttagcataatgccctcagggtccatccgtgttgcttcaaatggca aggtttccttctttcttactgTTGGGTAATATTCTGCTG GGCATATAGACTGCATTTTCTTCATGcactca tccattgatggacacttaggttgcttccgcgTCTTGGCTAT GGTAAATGAGGCTGCAACAAACAGGGAG GTGcgtatatgttttcatttccttcagataaatacccagaagtgaat tttctgggtcatatggtaattctatttgtaattttttgagaagcctccata ctgttttccatagtggctgcaccactttacattcccaccagcaccgC ACAAGGGTTCTTgccacatcctcgccaacacttgttattt cttgtcttttcgaTAACGGCCATAcggtattttattttaatccg CAGCACAGTTCTCAAAGTAGATACTCATG TATCatgcccattttaaagataagtaaGTAGACTCAGA GAGGTGGTTTACCTCAGGTGAACTGGAG CCCTGAGTGACTGACTCTAGATCTTGCGT TTCCCCCTCGtcatctctttttcctctgtctcttccttgTTC CTGTTCTGCGCTGCTTATTTGAGTTAATC ATATGCCTGATTATGATGAATAATAACTGA AGTTCGTGACCCAAGGTCATAATCAACTT TGCAAGTCTCCTTGTTTCCTCCCTTCATT CCAAAGCCCACTCTGCAGCCCCCAATAA TTACCACTCTAAAATAGGCTAACGCATGT CTTTTCAATCCACCTTCTACATGCTAATTT TAAGTATAAAGATatccataaatataaataatactcCCT GTGTTTTAACTTTCAGTTAGTGAGCTTAG |

| | |
|---|---|
| | CTCATTTACTTTTTGTAATTTCTGTACTGT<br>CAGGACTTATTTCTGCCATCTTAAATGGC<br>ATCTTCTTTCCTACCTTTGTATgggttgattttattt<br>tcctgatataAAACTCACACATTTTATAAAACC<br>CATACTTATGTTGAATGATACAATTGCAA<br>AAAGCCTGTAGTCATGTTGAATTTTTACT<br>ATCAATTTCTAAAGTTTATCAGTGTCCAC<br>GTCTTCCTTCCACCACATCTAAGGGTCGA<br>GTATCTTAGTATATTTTCACCTGTCACTAG<br>TTCCTCCCTATGAATTTGAAGTCCACATT<br>GTCACGGGTATACTTGGTGCctgtttgtatatgtat<br>gtgtgcaatCATTGCTTAGTTTGATAACAATTG<br>ACTTTTCTACACTATTATCCATCTTTACTT<br>CTTAGAACTTCCTATTCTTtcctggatttatttatttac<br>ttgctcATGAGGTCAGCTAAGAGAATCTTCA<br>TGTAGCAAACTTTCTGATGTTAGAATAGC<br>TTTATTTCACACTCAAATTTAAATGAGAG<br>TTTAGCAAGATATAAAATTCTAGATTAAA<br>AATCTCTTAAATTTTAGTTCAGAAACACA<br>TCAAAAAATATTACTTTCATGTCATCTTGT<br>ATCTGTTGCTGCCGAAAGTTCTAGTTAAG<br>GCTGATACCTGTTCACATGTAAGCAATGT<br>TTTCGGTTTTCAGAAAACTTTTAGGGTGT<br>TATCATCAAAATAAATTTGAGTgcgatttttatttt<br>ctattctgtttggCATGTATGAGAACACCCTTGCC<br>TGCCTGACCCTCGGGGAACCTCCTGACA<br>CCTCACTGTAGGCACTTGCCTTATCTGGG<br>GTTGGGGGCACCAACCCTGTTATCTGGG<br>GTTGGGGCACCCACCCCAGCCTGGGGTG<br>GGATACACACAGCTACAGCCGCGGGGGC<br>TGGCCTTCTCCCTGCAGCGGAACTCGCT<br>GGCACTAGATGGCGTTGCTGCACCCCTCT<br>CTTCTGGTTTGCAGTAGGGAGTGGCTGT<br>GGTGATGCGCAGGGACTGTGCGTGGTGA<br>AAAACAGGCAGAGAGAAGCTGTCCTGA<br>GCCGGGCATCTCTCTGTACTCAGACCCTC<br>TCCCACTCCAGGCTGTGTTTCCTTTCCAC<br>ACTGGGTCCCAACTTTTCCATCAGGTGCC<br>TGAGTGGGTCCTCTGAACCGAGCAGTTT<br>GGGAGCTCTTGTCTCCCTGTCTTCCCTCA<br>GCTCCTTCCCACGctgattccatcacttccttTTGTC<br>TCCTCTGCAGTCAGACTGGTTCTTCAGCG<br>ACAGCGAGGACAAGGGAGAGAGAGTGA<br>GTGATGCAGAGGGGCCGTGGGGTGGGGT |

| | |
|---|---|
| | CAGGTTGGTGGTGGGTCCTCATCCcaggtgggtggggtgggcaggaagtCTGAAGCAGGAGAAGGTTCCTGCCCTTAGGACATCCCAGTCTGTTGGAGGAAGCATAGGCCCTCTCCTCAGGAGCCTCCAGTCTGATGGGAGATATAGCCCTCACTTTCATGAACCCCAAGTCTGAGGGGGAGATAGAGGCCATGCCCTAAGGAATCTGAGTCCAGTAGCAAAGACAGACCCTGCCCTCCAATCTTATGAGGAGGACAGAGGGCTTAGGAAGGTCCAGTCTAATGGCGGAGACACAGACCCTACCCTCAGTGGAGGGAGCAGTGAGGCCTTTAGGGTTCAGCCAAGGTGTGGGGAAGGCAGGGACATGTAGGACTGCCCTGGAGTCCCTGGCTGATGAGGGAACACTCCCTGAGCCGGTGAGCCCCAGTCTGATGGAGGAGACCTGCGCCCTGTCTTCAGGGTTTCCCGTTCCAAGGGGTTTGCAGTCACCCATCTGAGGCCGTGGGCCTGACCCTTCTCCTGTTTGCAGACTCCCGTGGATGACAAGGAGCCTCAGTCAGTGCCAAACATCGAGTACCTCCTGCCTAACATTGGCAGGACAGTGCCACCTGGAGAGCCAGGCTCAGGTGAGAGCACGGGCCCAGGAAGAGGTGAAGAGGCCATGGGATCTGCATTCCTGAGGGTCCCAGATGCCTGGCCAGGCGCCCAGCTGCCCTGCTCCTGCCCCAcggggcccctccctgccccaccactGGGCTGGGCTGTGGCGGGGGCTTCCGCCTCCTCGGCTGCCCTGGGTTGGGTAGGACCTTTGTTTTCACCGGTTTCTTTCCTGAGCTTTTTGTTTCTCGAGGGCTGTGTCCTGGGCACGtgcctctttttaatttttcttccttttctccttgcaccccctcctcccctcttccctcttctccctgtGCTCTCCCCCCATCCCCTGGATCCtgctcttctcttctcctcccttccttcctccagcGGACCTGTTGGAGATTTAAAGGGTACAGTGTGGCATTGCGACCTCGGTCACTAACAGTGGCGGGTGATTATAAGAAGGCTGGGCAGGCATGGGGCAAGGGACAGGCAGAGCTGTCCAGGGCGGTGAGGACCTCTCCCCTACAGTCCAGCTCCCCACGCTTGTTAACAGCGTACCGGTCTCCCATCGGAAAGCCCCTTCTCAGGAGGAAGGAAGCACTTGTTAGAGCCAAGTCGGGTCCCCTTGCCaggaagcctgggcaccacatgGCTGCCCTCCACAGCCCCCCGGCCCCCCTACTCTATGGCTTTCTCTTTGT |

| | |
|---|---|
| | GCTGAGCTTGGCCACTTCTGATTATCTGG<br>GTGGAGTAGGCTTGGCTGGTAGTGGGAC<br>CCAGCATCTCCAgacttcagtgatgccatcccagaAG<br>CCTCTCTGCATCTATAGCTCCTGCGTCGG<br>CCGCCTCTGAAGGCTGGCTCTTCCTGCTC<br>CCGAAATCAGAGCCCCTGGTCACTCAGC<br>CCCTAGTCCTGCTCCCAGGGGTCTGGCT<br>GTCATAGTAGAGGAGTCCAGGGACAAGA<br>GGGTAGCCTTCTGGCTCTAGCCACTAACT<br>CCCCACTCTGTGGGTGACCTCCCCCCTTG<br>CGTCTCTGACAGAGGCATACCACAGCAC<br>CCCTAGTTGCAAgaggcaggctggggagggggccag<br>aggacgtgggggtggggatggccgAGAGGAGCCCCC<br>CGTCTCTTCTAATCACCTGCTGCCACTCT<br>CCTGCCCGTGTGTGTCGCTGTGTGGTCA<br>GCCCATCTGCTCTCACTGTGTgcaggggaggg<br>ctggggaggaggcatCTAACTTGCTTGCTGGAG<br>AGTGTGGATTAACCGCACCTGCCCCCCTT<br>CAGACCCCCAGAGATCCTTGGGTGGGCC<br>TGCTCCTTCCCCAGTCCTGGGGACAGCA<br>CTGCAAGACTTATTTCTGggtccttccccaccccag<br>tGTTAATAAGGGACTGGAACCCACATTCC<br>TTCATAACTCTGATAGTAAACAGGGCCTT<br>TGCCCCTGAGGACAGCCTGACCTCTGGT<br>CTGGAGGTAGACGCTTTCCAGGCTGTAAT<br>CACCAGCCCTCCCTACCTCCTGCCCTGCC<br>CATTTCCTGTCTGCCCTGTGCCCCATCATT<br>CACATGGTCCCTCTGCCCTCTCATGTCCC<br>TGAAGTCGGGGGTGAGGGTAGGTATAGA<br>GCGCCCCTCTCTGTGCACTGTGTGGTCTC<br>CATTTTTCCAGAGCATCTGTGATTTGCTgt<br>gtattgtggtggtttctgtgAACTGCATCTGATGTTT<br>GGCGTTTTCTCTCTAGTGCTTCAGAGTAA<br>CAgggacaggaggggaggggagaggccaaAGAAAAG<br>TCCGCCTTGCCCTTAACCCTGGGCAAATG<br>CAGGTCCAGGGGCCTGATGTCACTTCCT<br>GCTTCAAAAGGAAGTGGTGATTGACATC<br>CTTCTCCACTGCATCCTTATCCCCCAGGA<br>AATGACATCAGAAGTCCACCGCGCTCCC<br>AGGACCTGGGGAGAGCTCAGGGTTCTGA<br>ACTCCCTGGTTTCTGAAGCTGTTGAGGA<br>GAGGGAATTGTGTTCACAAGAATCAGCC<br>TTCTCTTGCCCAATccaactttcatttttcctttgtttcttac<br>cAACAGGTTCCACCACCTGTAGTTCAGCC |

| | |
|---|---|
| | AAGTCTAAG |
| AC_000182.1:38287754\|38329596 | ACTGAGCAAAGGAAAGCATGCACATCTG<br>AGAGGCGCTGCCCTTCGAATACAGACCT<br>GAGACCATCTCAGTGACCGCTCACCCCC<br>AAGTGTTCTGGAGCAGACACTCTGCCCC<br>AGGAGGCAACACCACCAGTGACTGCCCA<br>GCTCATGGAGCTGATGCTTTAATGGGCCG<br>GGGCTGCAAAGCGGTAGGTGCTGGGGGT<br>TGGGTGAGCAGGATGGGCCGTGTTACAC<br>TGGTATTCAAAGCAGGCCGGGCCGGAAG<br>GTGCGCAGGTATAGAAGGAGGTGGGAGA<br>GTCTACCCACAGGAGGCCAAAAGGACCA<br>GCTCTTGTGTGTTGTGAGTTTGCAGGTGT<br>TTGAATGGAAGGGGAAGTAACAGCAGGT<br>TTGTAAGTTGGTGGGAACAATCAGATGCT<br>Gagggggagtgggaggaggggctgggggagggggagaaag<br>gggATGGGGTCCCTGGCGGGATCCTGGAG<br>ACGTGGGTGCCTGGGACGTGGGTGCCTG<br>TGCAGGTGGCGCGTGGGTGGAGGGGAC<br>ACTTTCTTCCAATGGCCTCTGTGTGAAAC<br>AGGAAGCAAGACCCGCTTCTGGGAGTAA<br>CCTCACCTCCAAGCCACCTCCTACCTCAG<br>CTCATCCCGGGGCCATCCCAAGAAGTCC<br>CCCAACCACTTAACGAGGAAACCTGGGC<br>AGAGAGGTGGCAGGGCCTTTCCACGGTC<br>CCCGAGCCACAGAAGGTGGGTCTCTGCA<br>TCCCAGATCCAGCACTCCTGACCCGAAT<br>GAACCGGGCCTCCATGCCAGGTGCGCCA<br>GAGAAGCCAGCCACTGGATGCTCATTTTA<br>ACAGAAACATGTTCTTGAAGAAGACTTG<br>GCAACATACCtcctcaggcttcccagatggcacagcagta<br>aagaatctgcgtgccagtgcaggagatgcaggttcgatccctgttc<br>ccagaagatcccacgtgccgagaagcaactaagcccatgtgcca<br>caaccactgagcttgtgctctggagTCCAGAATCCGTG<br>ACTGTGCAAGCCTGcaggccctagagcccgtgcttcc<br>GCAGCAGGAGAACCACGGCAGTGAGCA<br>GCCCGTGTGCTGCCAggggccacagctagagagaa<br>gcccgcgccgcagcgaagacccagcaaagtcaaacatttttaaa<br>aatagaaaaaaaaaaaaaataccttcctCCTGTGCATATT<br>TTTCAGATTGCTGAAATTCTGCTCCTAAT<br>CCACTTTTACATCATGAGTACATtaaccaaaaa<br>aatatatatcttagcATTGCCAGTTCCACAGAAGT<br>GAGAATATGGTTTTAACTggtatgtgtgcatgttttt<br>GCTGCGAGCCCTCAGCAGGGCACGTGGT |

| | |
|---|---|
| | TGGGTCCACGTGGTCAGGTAGTGGACGC<br>GCTATCCCCTGGCTCAGGAGGAGGGTTTT<br>GGCGGGGCGGGCAGAGAAGCTGCCACT<br>GTCCTCCCTGCAGACCTTGACAGTCGCC<br>AAGAACTGTCCTCAGCTGTAGTCAGAGA<br>AAACCAAAACCAGGATGGTGCCCTTTCT<br>GGCAGGCTTTGAGAAGTGCTAACAGCCC<br>ATCACATGGGCTGCTCACACATTCAAGA<br>AGTTACAGTCTCACATGGTTGGgattgtggcaa<br>aaaaaaaaaaagaggaggaggaagcaaaTATCATTTCA<br>AAATCCAAGACTGTAACTGATTACAGACT<br>GCCTGCCTCAGAGAAAGCCCTTaccaaatattt<br>gctaaatgtttCTCTTAGATAAACATCGAGTTTT<br>CGGTTTGATGGTAAGCCTGTGGCAAGCC<br>TTACGGTTCTACTAGACAAatctttctaaatgttttat<br>aaattaagcACAGATGCTTTTTATACCACACA<br>GGCGACAATTTTATGCAAGTCACGTGGTG<br>AGTCTTACTGAAAGTGAAGGCAAGACCT<br>CGTCCAGtgcactttgcagatactgcatttttttttttttaattaa<br>agaaagtgAAGCCCATGGCAAGTCTGTCAGA<br>GCCAGTTTTACAGCAGCACATGCTCACCT<br>CGTGTcggtgtcacattttggtaactgTCCCAATATTT<br>CAAGTTGGttcattattattacatttgtgatggtgatctgtgatc<br>aatgATCTTTGATGTCACAGCTGTGACTCA<br>CTAAAGGCTCAGGTGTTggttagtatttttttaataaag<br>tatcttTAGGtatggggatggggaaggaggttcaaaacgaggg<br>gatacatgtatacctatggctgattcatgttgaggtttgacagaaaca<br>aCAAATTCTGTCAAGCATTATCCTTCAATA<br>GAAAAATTAAGGTATGTatgtttttttagacataatgct<br>attagACCACAGGGTGTTGtaagcataacttttatatgc<br>actgcggaaagaaaaaaaaattgtgactcactttattgcggTGAA<br>CCAAAACCCAAAATACCTCTGGGGTCTG<br>CCTGGATGTATTACTGTATTTAAATCCATac<br>tgggacttccgtggtggttcagtggttaagaatccgcctgccaatg<br>cgggaaacacgggttcgatccctcgtccAGGGACGAAG<br>GTCCCGTGTGCCATGGGGCGACTGAGCCt<br>tggagccacaactactgagcctgtgcgctgTAGAGCCCG<br>CGTTCCCcgagaagagaagccaccgcagtgcaagcccgc<br>acaccgcaacgGGAGGAGCCCCCAttcgcctcaacta<br>gagaaagcccacttgcagcaacgaagacccactgcagccaaaa<br>ctACATCACTTAAAATAAGATCTAAAAGTA<br>AAACACCTTCACACCCTTCGTTCTCTCAG<br>AGGTGGGCCTCTCTGCATAATCCTGCCCG<br>GAGGAACGCATTCAGTCTTGCAGCGGAT |

| | |
|---|---|
| | CACAGAAATCATCTCTTGAAGTCAGAATT TCCTAGTAATTCTCACAGGAACAGAGCTA GTTGTGGATGGCAAGTGTCTAGCTAAGTA AGTTGCCAAGCAGCTGTACTCAACACCT CTGAGCAGCCCTACAGTACCAAAAGATG TCACTGGCGTGTCTAGACCAAGCACGGG CTAAAACACAAGGTCCGCACGTTTGTGG CGACCACACGCAAGGCGAACCGAATGCC TCTCGTCACTACTGCGTTCAGAGGAGCA GAGCAGGGTGCTTCTGCGGGAGGAAGC GAGCTCCGGTCCCCGCTCTGCCTGTCCC ACACGCTCCCCGTGTGACTTAACAAGTC GCCTCGCCTCTGGAAGCCCGGCTTCCTTA CACAGAGACACTGCACATCCCTCAGGAT TATAACAGTGTCCCCCTCCTGGGGGCAG GTTCACCTCACTTTCCACGGCATAATCGC CGTTTCAAGGGCGTAGTCCGTGTGTCCC ACTCGGAGAGCGGGTGGACACCGCAGG GGCCCCCACTTCGTATCAGGGCTGTGATG CTTGGGCACCACCCCAACCTGGGGAGAC GGTTCATCTCCCCACTGAGTCGGGTCCAC TGAGCCCCTGGTGTCTTATTTCTGCACCC CAGTGCCTGGAACCTGCCGGATGTTCCAT GAACATGTAATGGAGCTCAAGGTCGCCT GCCTGGGAGGGGTCACACCTAGGTCCCT CTGCTCCAGAGACCATGAGGTTGTGCCC CTTGGGCTAGAAATAGGTCTGGATCATTC CCGAAGGCCACTCCCAGTTTTACAAAAG CAAAggacccagggacttccctggtggtccagtggttaagact cgacCTTCTAATGCAAGGGAggcaggtttggtccct gatccagagctgagatcccacatgcctcgtggccaaaaatacaaa acacaaaaacagaagcagtgttgtagCAGACTCAATAA AGATGTAAAAATGGTCCACGTTGAAAAA GTCTTAAAAAGCCAGTGTCCAGATAGTA GGGAGATGCTTGTTAAGACGACACCTGC TCTTTGGGACCCGCTCTATTTTCTGTGAT GACAGAATTTCCACCCTCTGCCAGATGTT AGTGAAAGGTACTCAGACTGACAGAAGG GATTTCTCTCTGAAATCTGCCGCCGCACG GACTTGTGACACTTGACCAAAGCAGTGA ATTTGGTGACACCTGGGCACCTCTCTGGC TGGGGGCAAGTGAATGACCACTCTCACG CCGAGAGGAAAGGGCACAGCCTGGGGT CAGGAGGGGCCCTCTGTGGGCCACCCCC |

| | |
|---|---|
| | CAGCACGTGCTCGCTCAGGGCCCTGCAG<br>GGTGCGGGAGGGCCTCGATCTGCACTCT<br>TGGTCATTATTCTGTCAGCGAGCCCTACA<br>GCTCGTTTTTCCTGCAACCgcagagagaagcag<br>aagaagCCACACAGCCCTGGCTTTGAAAAC<br>GGGGGGGCCTGGAGGACTGAGGAGGGC<br>AGGGGTCCTCAGGAAGGAGAGGggaccctct<br>cccccacccccctccatcccCGCTCCTACCTGCCGCC<br>TGCACGGTGGACATGGCTGGCTTCAGCA<br>AGGGGCCCCTGGGCGGGAGGAGGGAGC<br>CCGCGGATGGGGCCAGGAAGAGCGCCCT<br>TACGCTCCAGTAGAAGAGAGCAGGAGGC<br>TGGGGGCGACATGAATGAGGGACACAGA<br>AGGCCCCGGGGCCGGGGCCATTGCGGGC<br>AGAAGGCCGGGAGGAGTGGGGCAGGGT<br>GGGCCGCGGGGGCATAGAATGCGGCCTC<br>ACAATCCAGGGTTCTCAGGTTCTGAGCC<br>TGCAGTCGCCACCGCTGGGAAGAAGCCA<br>CAGTCCGGATTCCACCTCTGGCAGCTGG<br>GGCACTCAGAGAGGAGCCCGTTTAATAC<br>CACTGTCCAGGGGATCCTTGATAAAAGC<br>CACGACTTAGGagcggtattcttgcctggagacttccgtg<br>gacagaggaccctggcgggctacggtccagggggttgccaaga<br>gtcagacaagactgagcaactaacaaaaaCGAGTggtaggg<br>gcttccccggtggctatgccaacgcaggagacgtgggttcgatcc<br>ctgcattgggaagatctcctggagaaggaaacagcaacccactcc<br>agtactcttgcctggcaaatcccatggactgaggagcctggcagg<br>ctacagtctagggggtcacagaagagtctcGGAGACTAA<br>GCAGCAGAAGCAGCCCAAGCCCCAGAG<br>CAGCCAGGTGAGGACCCAAGGGGGCAG<br>GCTCTCCCGTCCCCCAGGGCGGCGGGGA<br>GGACTGTCAGCAAGCAGAGCGCTGAGG<br>CCCCAGGAAGCAGGCGCCCCTCGAATGG<br>GTCCACCGCTCCCTACTTTGCTTCCGTGG<br>TGGCTCCCCTTGCTTTCTGAGGACAAGTT<br>CCTTCCCAGTTTCCAGGGACACCCAAGG<br>AAAGGACGGGGGTTCTGCAGCTCCGCCA<br>GGAGGAAGGGCCCAGGGTCTGTGCGTG<br>GAGCCAGAGAGGAGCCCAGAGGCGTGC<br>CCCGTGATCACGACCATCAGTACTTTCAG<br>CTGCTGCCGCGAACAGTGCTAACAGGTG<br>TGGCCACGTGAAGGGCTCCGGGGCTCCT<br>CGCGGGCTGCCCTGCCGGAGGAACCCGC<br>TTCCAAGGCTGGGCTTTCCCTGCAGGTC |

| | |
|---|---|
| | ACACGATTACAGTCAGCTGACCTGGACG<br>CTGCAGCATAGGACTGGGTCTTTGGTGCC<br>TCCCACTAAGTAGCCCTCACCTCTCATTT<br>AGAAGAGGCGCCCTTTCCTAAAACGGTC<br>TCCTTCTGTCTTCCTACCTGGCGGAACCA<br>GGGCCTGTAGCCTGTTGCAGGTGTTAAC<br>AGTGTGGTCTCTCTTTTAAAGGGGACAC<br>AGCCTGCTCTTCCATCTCGGGGCTGCGTG<br>TCCTGGCCCACTAAGGAGGAGCCCActtgtg<br>actcagtttcctcacctgtaaaatgcggtcagtcagttcagttgccc<br>agtcgtgtccaactctttgcgacctcatggactacagcacaccagg<br>cctccctgtccatcaccaactccccgagcttgctcaaactcatgtcc<br>attgagtcggtgatgccatccagccatctcctctgttgtccccttatc<br>ctcccaccttcaatcttccccagcatcagggtcttttctgatgaatag<br>gctcttcacatcaggtggccaaggcattggagcctcagcttcagca<br>tcagtccttccaataaacacccaggactgatctcctttaggatggac<br>tggttggatctccttgcagtcccagggactcccaAGTAAAAT<br>GAGGTACTTCATCGGATCTCTGTGAGGGG<br>ACATGGCTGAGTGGGCGGCCGTCAGCGG<br>AGTGTGGGGCTGCCTCGGAGCCCAGGCG<br>CTGCTGGGGGCCCCACGCGGACGTGGCA<br>CACACAGCACCGACTGCGTGTGCAGCCc<br>gcccgcccccccgccccagaAGTTTTGGCGAGTGG<br>TTTAAGGTGGGAATTGTCATCGCTCTTGT<br>TACATGTATATgcgaggcttcccaagtggctcagtggtaa<br>agaatctgcctgcaatgcaggaggctcaggagatgtgggttcgat<br>ccctgggtcgcgaagatcccctggagaaggaaatggccacccac<br>tccagtattcttgcctggagaatcccatggacagaggagcctggtg<br>ggctgcagtccatggggtcacaaagagttggataagacttagcga<br>ctaaagaaaaACATGCATGCATGTAATTTGACA<br>AGGGTTGACTTTTTCCCCACATGGGCTcac<br>ctttctgttttgttgtgcAATTTGCTTTTTTCCCTGGA<br>GAGCTTGGCTCCTCCTCCGAGATCCCATG<br>ATCTCCATCCTCCGTCTGACCAGTGTGTA<br>TGCCCCTGCTCGTTGAAAGGCCAGGCCC<br>CCCTCAGATCAGACTGCAGTCTCCTTTCC<br>CTTGTTGATGATTTCCCAGGACTGCCCGT<br>GCGCCGTGCACTCTGTTGAACTCAATTCA<br>TGTTCAGTGAACCAGATTTTACAAATCTC<br>TCTTTTTTGGCACATCTAAATAACAATTAC<br>AAATACATTAAAGTAGTTCTCTTATTAAA<br>CAATGTAACCTGCAGTTATAAGTGTGGCA<br>TGGTTAGATTCTTTTGAATATTGCAAACA<br>CCTCAGACAACAAATAAAGCATATATTAA |

| | |
|---|---|
| | TGCAATGATTACCAAGCCAACCTCTTACT<br>CCAAGTAGCAACATCATGGATTTAACTAA<br>CATCTTCAttattccctctcttttttttttttcaaataccttTTTC<br>AGGTtacagctatttttattttgatactttTCCAGGACTG<br>AAGCCCCCAGAGAAGAGGAACACCATCT<br>CTCATCAGCTGGATTAAGGCATTTTGGTT<br>ATAGATGGAGGAATTCCTGGATGCAGCCG<br>GATCTGGACTGCTCTTCCCAGGGCCATCA<br>GACTAAAGATGCTGTTTATAAGTAAACAT<br>CATTTGCTGCATCGATCAGATTTGAAAGC<br>TTAAACCCTTCACTTAGGTTTCAgtccttttcca<br>gtttgtttcTCCCCTAAAACGATGCCATCTGTA<br>GTCCTGCTTGATGGTATTCTGTAACTTCTT<br>ATAGAAAAAGCCAAGCGAAATTCTTGGG<br>CCATTACATAATCCATCTCTGGGTTTTTAT<br>GTTTGACTAACGCAGTCCTGTCATTATGA<br>ATTAGTGACTTGACCTGTTAGCGTTATTA<br>AAGAGTTATGTTTGCTCTGTTAGTATTGT<br>GTGAAAATTAACAAGGCTGCCTAATTAA<br>ATGAAGGGTTGAGTCCGCCTGGACACCT<br>GGGGAGACGGCTGCAGGATCTGGGAGC<br>ACCCTGCTGAGCCCACCCCTTCCCTGGCT<br>GCTCTGTGCGAGTGCTAGGGCAGACCCC<br>TCCTGCTTCTCGAGTGACCACCAGTGTG<br>GACACTTCCCGCTTTCTGGGGGGCCAGT<br>GGCTTTGTCCCTTGGTCCATGCGGTTACT<br>GGTTTCTACTTCGACAGAAACAAGTAAG<br>CAGCATCACTTTATAATGGGAGGAGAAC<br>AGCACAGCTCTATGCTAGCAGCTGCCCCT<br>GGATTAGCTTTCTGTGCTGCCCAACAGAA<br>GACCAcagacttagcagctgaacacaGCACCCCATC<br>AATGGTCTCACAGTTCTCGGGGTCAGGA<br>GTCTGGGCTCAACTTCACTGGTCATCTGC<br>TCGGGGGGTCTCACAAGTTTGCGATCGA<br>CTGGGCTGCAATCTTACCTGGAGGTCTAA<br>GTGAGAATGATCTGGTCTCAGTCCACGC<br>AAGCTGCCAGCCGGATTCACTTCTTGGA<br>AGCTGCTGACGGAGGGCACCGGCTTCTT<br>GACTGTGGGCCGCCCTTGGATTTAGAGG<br>CTGCCCATGGTCCCCCAGGGGCTGCTCA<br>CAGCCTGGCTATTTGCTTCCGCAAGGCCA<br>TCAGGAGAGAGAGTTTCTTGCCTCCAAG<br>AGTGAAGTCCCTCTGCAAAGGGCTTTTA<br>CATAAATAAGCCAAGCTCACCCAGGATA |

| | |
|---|---|
| | ATCTTCATTCTGAATAACTCAAGACTCAA<br>CTGATGTGGTATTGGAATTACACCTGCAA<br>AATCCCTTTCTCTTTGCGTTATTCTGCCAG<br>CTAGAAGGTTGTTACATGTCAGCCCCCAT<br>CCAGGGAAGGAGTTTtatgggggagggcagag<br>accTCAGGGACCACCCTGAGTAGATGGTG<br>AAATCAGTGAGAGCTAAACACAGTCTGT<br>CACTCCCCCAgctgcccaggtggcaccagCGTGAA<br>GAACCCTCCTGCTAACGCAGGGGAcaagag<br>agacctgggtttgatccctgtgttgggaagatccgctggtggagg<br>gcatggcaactcactccagtatagtgtccttgcctggagaatcccc<br>gtggccagagaagcctggcgggctacagtccatggggtctcaga<br>atctgacatgaccgaagcgacttagcatgcacacagacacattaC<br>TCCTCCACTTCTGTGTTACAACCACCCTT<br>GTGCACCCTTCACAGTAACGGGTCCCTG<br>CAGAGCATCAGTATGAGCTCATAGGTTCG<br>CCATGGCCCCAGAGGCCACCTCCCTCTA<br>GGCCGTCTTTGCCCTGGCTACAGTCAGCT<br>GGCCAAAGTCCCTGCTTCCGGGCCTTTG<br>CACTTGACGATCGTTTTGTCCACTTCCCT<br>CCCCCGCATCCTCACACCTTCACTGTATC<br>CTGGCCTTGGCTAAACTACCGCCCCATCA<br>GAGAAACCCTAATGCCCTGAAAGCCGCA<br>CTACACCCCTCCTGATCAGATGGCCCCCG<br>GAGCTCCCAGGGGCCCTTGGCTTCTGCC<br>TGTTTTCTGAACTTGGTTTGGCAGCCCGG<br>TGCGGACTCCACAAGCTAAACAAAACCC<br>GTCACCAGGGATCCTCTTTTATGGTTTGG<br>TTTGTGACCCAAGAATCCTGAGCGGCCG<br>TGTGAACGTGGGCAAATTACTCTCCCTCG<br>GGGAACCCAAGGATGCTGTCAGGCAGGG<br>CTCAGGGGCTGTGTGAACTCAGAGGACC<br>TTCCTCTTTCCCCAAGATAAGTACCTGCA<br>AAGGATGGGGTTCACTTGGGCTCAGTTT<br>TTTGAGGATGAGAATGAGATACATGCCTA<br>TAATATTTGCTAACTTGGCTTCAGGTACTT<br>ATTTTCAAGCCACATTTGGGATAAATTATT<br>ATCTTGAAAAAACTTTTCTCGCTCTTACT<br>TTTCTGGAAATGACTTTTAACTCAAGCTT<br>CTCGTCATCTTCAACGTTGGTGTCACTGT<br>GACCCCAGagttctctctctgcctcagatTCCCAGCT<br>CACCTCGTCTTGTCTTATCTTCTGGACTG<br>TTCCAGCCAcggcctttttaaaaatcacgtgtgggggactt<br>ccttagtggcccagtggataagactccaccTCCCCAGTGC |

| | |
|---|---|
| | atgggacacagatttgattccaCAAGCTGAGAGtgaggc<br>caaaagaaaaaaaatcaggtgaTGCTGTCATTGGTA<br>AGTCATCCCACTCATGCAAAACTAGGAC<br>AGTCACTTTTACTATACAtcctttttcaaaaaagaaaa<br>ggaaagaccttttttaaattgtaaaatataaaacacagagaaaaatat<br>agaagatgtaaatgtgaattattttaaagcaaaacatgTAATCA<br>CCACATGGGTCAAGAACTGGAATGTTGG<br>CAACAATCCCAAAGCCTCAGTATGTCCCT<br>TTAGTGCCACCAAAGGCGTCTACATCCCG<br>GTTGCTATGGTGACATTACGGTACTCACT<br>GTCTTGATGCTTTGACCATTAAACGTACA<br>TCCCTTGATGTTATAATTAAGTTCTTGCCT<br>ATTTTTGAGTCTCACCTGTAAAAGGATCG<br>TACAAGGCTCTCTGGGGCTCTGGCTTGTT<br>TACTTGGCACGTGTTTAAGATTCACCCAC<br>GTTGTTGCACTGTTGTGCttcattctgtttttgttgtta<br>ccAATAATatcccaaatataaataaataggccacatttattcatc<br>cattctacTGGTGATGGACACTCGGGCCGTGC<br>CAGACACGGCTGCTGAGAACGCTCTTGC<br>ACCCGCTCCTGCTGTGTGTACAATCTAAG<br>GCGATACTGTCCCCGGGCCAGTCCCAGC<br>TTCGGCGTCAGAGATAAGTGATGATGTCA<br>GACCAGTTTCCCCAGTGACAGCGTCAGC<br>CCACAGTTCCATGTGGCCTTCCTGGGGG<br>GTGTCTCATTGTGGGTTTTATCTGCATGTC<br>TGTGCTTGCTGAGGTCGAGCACTTTTCCC<br>TACACTTACTGACCATCcatatttccccctttttttttctt<br>aaaagttgattcgagtcctttgcttttttttttaagtgtttgtcTTGTT<br>CATACTGACTGACAGGCGTTGTTTTGATA<br>ATCTAGATCTTGGCTCTTTGTTGGCAACA<br>GgtgttgcaaatatcttctcctgcTTGGTGGTTTGCCA<br>TTTGGGGGTTTGTTTgttaatgctatgctatgctaagtc<br>gcttcagtcgtgtcagaccctgtgcgaccccatagacggcagccc<br>accaggctcccccgtccctgagattctccaggcaagaacactgga<br>gtgggttgccatttccttctccaatgcatgaaagtgaaaagtgaaaa<br>tgaagtcgctcagtcgtgtccgactcttagcgaccccagggactg<br>cagccttccaggctcctccgtccatgggattttccaggcaagagta<br>ctggagtgggttgccttgtttgttaatattttatttatttaaaaacttttaa<br>ttttatattggagtatagctgattaacaatgctgtgatagtttcaggtg<br>aacagcaaagagactcaccCATACACGTATCCactctc<br>ccctaaactcccttcccatccaggctgctacataatattgagcagag<br>ttctctgtgctatacagtgggaccttgttggttatctattttatatgtaac<br>tgTGTATATGTCGATCTCAAGGTGGTTTGC<br>CTTTTCACCCTCTTAATGATCAACAGAAG |

| | |
|---|---|
| | TTTTCGATTTTAAAGTACTTGAAGGGGGAGAgacaaactgggagattgggactgacatacacacactactacatGTGAAGCAGagaactaataagaacctactgtatagcacagggaagtctatgCAATACTCTCTAATGTCCTGTATGGgagagaatctaaaaaagagtggatgtatgtgtacgtgtgtgtgtgtgtatatatatgtcatgcccggtggggccacccaagacgggcgggcatggtggagaggtctgacacagtgtggtccactggagaagggaatggcaaaccacttcagtattcttgccttgagaaccccatgaacagtatgaaaaggcaaaatgataggattgaaagaagaactccccaggtcagtaggtgcccaatatgctactggagatcagtggagaaataactccagaaagaatgaagggatggagccaaagcaaaaacaatacccagttgtggatgtgactggtgatagaagcaaggtccgatgctgtaaagagcaatattgcgtaggaacctggaatgtcaggtccatgaatcaaggcaaattggaagtggtcaaacgagacggcaagagtgaacgtcgacattctaggaatcagcgaactgaaatggactggaatgggtgaatttaactcagatgaccattatatctaccactgtgggcaggaatcccttagaagaaatggagtagccatcatggtcaacaaaagagtccgaaatgcagtacttggatgcaatctcaaaaacgacagaatgatctctgttcgtttccaaggcaaactattcaatatcacagtaatccaagtctatgccccaaacagtaacgctgaagaagctgaagttgaacggttctatgaagatctacaagaccttttagaactaacacccaaaaaagatgtccttttcattatagaggactggaatgcaaaagtaggaagtcacgaaccagctggagtaacaggcaaatttggccttggaatacggaatgaagcagggcaaagactaatagagttttgccaagaaaatgcactggtcatagcaaacaccctcttccaacaacacaagagaacactctacacatggacatcaccagatggtcaacaccgaaatcagattgattatatccttgcagccaaagatggagaagctctatacagtcaacaaaaacaagaccaggagctgactgtggctcaaatcatgaactccttattgccgaattcagacttaaattgaagaaagtagggaaaaccactcgaccattcaggtatgacctaaatcaagtcccttatgattatacagtggaagtaagaaatagatttaagggactagatctgatagatagagtgcctgatgaactatggatggaggttcatgacattgtacaggagacagggatcaagaccatccccatggaaaagaaatgcaaaaaagcaaaatggctgtctggggaggccttacaaatagctgtgaaaagaagagaagcgaaaagcaaagaagaaaaggaaagatataagcatctgaatgcagagttccaaagaatagcaagaagagataagaaagccttcctcagcaatcaatgcaaagaaatagaggaaaacaacagaatgggaaagactagagatcccttcaagaaaattagagataccaagggaacatttcatgcaaagatgggcttgataaaggacagaaatggtatggacctaacagaagcagaagatattaagaagaggtggcaagaatacacagaagaactgtacaacaaagatcttcacgacccagataatcacgatggtgtgatcactcatctagagctagacatcctggaatgtgaagtcaagtgggccttagaaagcatcactatgaacaaagctagtggaggtgatggaattccagttgagatatttcaaatcctgaaagatgatgctgtgaaagtgctgcactcaatatgccagc |

| | |
|---|---|
| | aaatttggaaaactcagcagtggccacaggactggaaaaggtca<br>gttttcattccaatcccaaagaaaggcaatgccaaagaatgctcaa<br>acgaccgcacaattgcactcatctcacacactagtaaagtaatgct<br>caaaattctccaagccaggcttcagcaatacgtgaaccctgaactt<br>ccagatgttcaagctggttttagaaaaggcagaggaaccagagat<br>caaattgccaacatccgctggatcatggaaaaaccaagagagttc<br>cagagaaacatctatttctgctttattgactatgccaaagcctttgact<br>gtgtggatcacaataaactgtggaaaattctgaaagagatgggaat<br>accagaccacctgatctgcctcttgagaaatgtgtatgcaggtcag<br>gaagcaacagttagaactggacatggaacaagagactggttcca<br>aataggaaaaggagtacatcaaggctgtatattgtcaccctgtttatt<br>taacttctatgcagagtacatcatgagaaacgctgggctggaagaa<br>gcacaagctggaatcaagattgctgggagaaatatcaataacctc<br>aggtatgcagatgacaccacccttatggcagaaagtgaatgggaa<br>ctaaaaagcctcttgatgaaagtgaaagaggaaagtgaaaaagtt<br>ggcttaaagctcaacattcagaaaatgaagatcatggcatccggtc<br>ccatcacttcatgggaaatagatgggcaaacagtggaaacagtgt<br>cagactttatttttggggttccaaaatcactgcagatggtgactgca<br>gccatgaaattaaacaatgcttactccttggaagaaaagttatgacc<br>aacctagatagcatattgaaaagcagagacatttctttgccaaaaaa<br>ggtccatctagtcaaggctaaggtttttccagtggtcatgtatggatg<br>taagagttggactgtgaagaaagctgagcaccgaagaattgatgc<br>ttttgaactgtggtgttggagaagactcttgagagtcccttggactg<br>caaggagatccaaccagtccattctgaaggagatcagccctggg<br>atttctttggagggaatgattctgaagctgaaactccagtactttggc<br>cacctcacgcgaagagttgactcattaggaaagactctgatgctgg<br>gagggattgggggcaggaggagaaggggacgacagaggatga<br>gatggctggatggcatcactgactcaatggacgtgagtctgagtg<br>aactccgggagttggtgatggacagggacgcctggtgtgctgcg<br>attcatggggttgcaaggagtcggacacgactgagcgactgatct<br>gatctgatctagcaactaaataacaaagtAATTGagtatattaat<br>tttttcctttaaggtTGGTGCTTTTTGCATCTTTTGT<br>TGAAATCCTCCCTTCCCTCAAGATTGTTA<br>GGATGTTCTCTTATACTGTCTTCATGATGC<br>TTTGTTTGGTCTCGTACACTTGGCCCTGT<br>GATTAGCGTGGTCACGTCTCGGTGTGTGC<br>CCCTTTTCTTAGCATCTCTCCCAATAAGC<br>ATCGCTTTTACTCTCAAAAGTAAAAGAAT<br>TTACTGCAGAATTTCCTGTAGAAATTCCT<br>GTAGAATTTCCttaagcttttcttccagggtAGATGAT<br>ACTTGTGGTGCTGGCCGTGGGCAGGGCT<br>CCGCTTCTATGCTAGAGGGTTGCTGATGG<br>GGTCTGGGATGGGAAACGCCACTCTGGA<br>GTAGCTTAGTGAAAAACTTCATCTGTAAA |

| | |
|---|---|
| | TATCTTGACAATGAAGCACATTTTAGGTG<br>CAACTGGTGTGCTGGGAAAATTATATTCC<br>TGGAGTCAAATATACAGAACACGCCTTTT<br>TTATGCAGGTCTAACACAGATCGCGGTCT<br>GGTTTTGAATATTCTGTGAGACTTTGGTG<br>Ccatttgacttccctggtagctcagagggtaaggagtctgccagc<br>aatgtgggagacccgggttggatccctgggtggggaagatcccc<br>tggagaaggaaatggcaacccactccaatattcttgcctggaaaat<br>ctcatggacggacgagcctggcaggctacagtccatggggtccc<br>agagagtcagacacgactgagcggcttcacttcacttcacttagtg<br>cCATGAGAAGCTCCTCAAACTGCTCCTGA<br>CCAGTAGGCGTCACCTGGTGGGGTACAG<br>GGTGAAGCATAAGGCAAGTGGAAGGTGG<br>GCCCTGCCTGAAAGGCTTCTGGTGGAGA<br>ACAGATAGATATACAGACCTGTATTCCGA<br>CAGGTGTATAAATATTAGGGACTTGAAGG<br>TACGAGTGcaagtttgtttatttggctgtgctgggtcttagttg<br>cggcatgtgggatctagttccctgaccagggaccgaaccagggc<br>cccctgcactggtggggagtcttagccaccagaccaccagggaa<br>gtcccatgagtGCAAGTTTTATGTTGTTTGTTG<br>GAGTGTAGCTTATGTTAAGCATATTAACC<br>TGGGCTTCTTGGTCCACTCCTGACCCTTG<br>TACTTCACCACTGACCTCATCAGTTTATC<br>CTCCTTAACCCTTGATTAGCTGCCTTTATA<br>CACCCAACTCAAATAAACCCCTACAGGT<br>TACAGCCTTTTCTGCCTTCTCACCTATTTC<br>TCTCTCCCTGATTGGACCTTTTCATCCAG<br>ATGAAGAGGTCTCTTGACCAATCTGAAC<br>CCCCAGTAGGGCCGGACACAGTGAGCAC<br>TCCTAAAATGTCTGTCATGTGAACGTTTG<br>ACTCGGCCGTGTTTACATGACTTTGCATG<br>ACTGGGTGGGGGTCAGGCAGCTGACAGT<br>GACTGTCGCTGGCTCCAGGTTTAGCTCGC<br>TGTTTGTTAGAGGTTGTGTAGGTTTTCTC<br>TTAGATACAAAACATCCCCTCTTCACGTG<br>AAGGCACGGGTGGCTGACGTTGGCTGTG<br>TGAATCTGGACATGCACGTGAGCTGCAC<br>GTTCTACACAGTGTTTCTGCAAGTTTTTC<br>TCTACCATAGATGGGGGAAATCCTACATT<br>TTTAGCTGCTTTATCTCTTAAGTATCAAGT<br>GAAAAACGAAAGTGTTGAAATCCATTTC<br>ATCAGCAGAGTAAAGGAAGGAGCTGTAT<br>GGCGTGGGCTGCCCCGGGGGCCAGCCAG<br>CACTGGCAGAGTGGTGAGAAGGGGTCGT |

| | |
|---|---|
| | CAAGCTGAGGAGCGAGGCTGCCCAGGG TCCCGCCCCAGGAGCGCCGGTTTCATGG CCAACATATGACTGACTGGCTTTACAGAG GGAGGAACGACTGTAAGCAGAATTAAAG TGAAAAGACTGTGTTCTGCTTGTGAGTA AGCGTTTACTCCCAGTGAAAAATAATGTC CACAGAGAAAGGAAACATCAGTGGCTAA CAAAGAGAAATGCCATTGCAGTTTAAGA ACAACCTAAGCACCTCAGCTTAGCTCAC GGTAGACAGCCTACCATGTCACGAGTCG GTGAAGACAGCCCCACACATTGCTGATG GGGGGGCTGAAATGCAGCCATGGGGTGA ATGTGTGTGAGTCAGTTCTGCTAATCTCT GCCCTTTCGTTCAGGCTCTTCCAACATTT CTCAATGTATGAATTTACCCTTCTTTCTGG GGGACTGAGGGAGGGATATTCATTCCTTT ATAAAAATCAAGTACCTGTTGTGTGCTGA GCAATCATAAAGCCCTGGAAATTCCAAC ATAAGTAAGACAGGCACTCCCTCGGAGA TCTCATGGTGTGGTCAGGGAGACAGACG GGAAGACAGACAATTGGCATGTGACACA GGAGGGGCTAGAATGGAGAATGTGCAAA GTGTGAGGGTGTAGACTCAGGTCTATTTT CCCTTGGTAAGATCTGACCTTTGATCTGA GACTTAAGGTGGCATGAGAATGGACAGT GGAAGAAGGAAGGTGGACAGGACGTGT GGGACAGCGGGGTCTGTGTGCAGGAAG CCGAGGGGTCTGATGGAGCAAGGGAGG GCCCGCATATGGAGCTCTGCCAAGGCTC AAGCTGAAAGGTTCAGGGGAGCTGAGG CTACCACGGAAGGGGAAGCCAGGTGGG GGAAGCCATCGGGAAGAACCTCAGCTCT TTCCTATGGTTGATGGGCAACCGTTGGAG ACATCCAGTCAGCCCATCCTGGCA |
| unconservative_AC_000158.1_1338 | aguugcuguugguuuacuuuu |
| unconservative_AC_000159.1_32276 | guggacuucccugguagcucagc |
| unconservative_AC_000161.1_106583 | gguggcgggggagguc u |
| unconservative_AC_000162.1_112908 | gugggcuucccugguagcucaga |
| unconservative_AC_000163.1_141219 | cgagggucgggucaucgugcu |
| unconservative_AC_000163.1_144851 | aggcugguccucugugcucaga |
| unconservative_AC_000168.1_251602 | cuggccccucccuccaccacc |
| unconservative_AC_000169.1_288145 | gcauggaggaaguggacugagcgc |
| unconservative_AC_000170.1_301545 | cacacucagauaaaguaggcacu |
| unconservative_AC_000171.1_311957 | ugccgcgcuguuucccuggugu |

| | |
|---|---|
| unconservative_AC_000171.1_312038 | guggacuucccugguagcucagu |
| unconservative_AC_000173.1_360722 | gugggcuucccugguagcucaga |
| unconservative_AC_000176.1_409242 | acuccuggcucugccacuccca |
| unconservative_AC_000176.1_413881 | uccucacugccugccucucccuac |
| unconservative_AC_000178.1_450303 | uccccuuccuuccggccuccgcc |
| unconservative_AC_000183.1_527362 | ccccgccaccaagccucuggacc |
| unconservative_AC_000184.1_528794 | ucccggggccgggagcga |
