# Supplementary material for: DNA Double-Strand Break-Related Competitive Endogenous RNA Network of Noncoding RNA in Bovine Cumulus Cells
Source: Genes (Basel). 2023 Jan 22;14(2):290. doi: 10.3390/genes14020290 (PMC9956238; doi:10.3390/genes14020290)
Supplement: Supplementary file 1 [file genes-14-00290-s001.zip › Table S3.pdf]

Supplementary Table S3. Differentially expressed mRNAs

#ID	gene_name	NC_ FPKM	NC_ FPKM	NC_ FPKM	BLM_ FPKM	BLM_ FPKM	BLM_ FPKM	Pvalue	Log2FC	regulated
gene9992	HMGB2	43.96784	46.08824	49.79656	22.02191	15.51768	15.56524	4.98E-13	-1.32074	down
gene9866	SLF1	4.62396	5.024332	5.134513	3.660386	3.094533	3.620888	3.80E-05	-0.81022	down
gene9775	HMMR	12.68542	16.28508	15.01507	26.21043	19.7397	19.42994	0.000311	0.630958	up
gene9773	CCNG1	87.66326	102.542	90.7674	288.0516	163.2501	192.1848	3.59E-08	1.244513	up
gene9732	LSM11	2.758013	2.638591	2.352052	1.381698	1.662005	1.219207	0.000194	-0.82136	down
gene9728	NIPAL4	0.429769	0.268893	0.319445	0.601118	0.752116	0.848384	0.003751	1.040367	up
gene9662	ZNF300	0.32101	0.269624	0.421455	0.889678	0.423449	0.561526	0.018691	0.935307	up
gene9636	PPARGC1B	5.338995	5.135946	5.319066	2.489349	2.487739	3.299883	1.49E-07	-0.89296	down
gene96	ADAMTS5	1.12535	1.033734	1.125443	1.920965	1.707687	1.944984	0.004064	0.829081	up
gene9550	PCDH12	0.030928	0.14934	0.069819	0.181109	0.173128	0.140246	0.041479	1.028123	up
gene9485	HBEGF	3.297333	3.534202	3.86769	8.037475	6.394584	6.934909	6.31E-06	1.05501	up
gene9451	EGR1	1.004691	1.219638	2.145862	1.959622	3.022036	6.961574	0.000901	1.479071	up
gene9166	ZFP62	0.684711	1.140929	1.426992	0.429769	0.877657	0.312004	0.035091	-0.93092	down
gene9128	MXD3	3.796266	5.018057	3.283664	1.034948	0.929221	0.875134	2.31E-07	-1.64103	down
gene9036	LMNB1	25.93197	27.72567	28.30609	18.72105	13.62368	13.36093	2.70E-06	-0.8103	down
gene9026	SLC12A2	7.063255	7.898139	7.897692	13.35394	10.18498	11.04987	6.12E-05	0.654689	up
gene8991	ALDKA	8.022347	14.38593	8.240848	13.58241	12.62818	12.5043	0.010819	0.631149	up
gene8969	LMNB2	21.69919	20.15887	20.17407	10.72371	11.02245	10.18895	7.49E-08	-0.89427	down
gene8957	ZNF77	1.821168	1.956249	0.854162	6.893705	2.52781	1.769203	0.000118	1.258274	up
gene8956	TLE6	1.209052	1.472917	2.165964	2.733946	1.906081	3.784497	0.011746	0.781053	up
gene8903	CHAF1A	10.95338	9.968894	9.882492	3.804269	5.479927	5.330959	2.36E-06	-1.00439	down
gene8895	SEMA6B	0.245205	0.192306	0.21313	0.410701	0.882081	0.741203	0.000172	1.597508	up

gene8881	UHRF1	17.00643	16.27049	16.53472	6.733267	8.046743	6.975356	1.89E-10	-1.11236	down
gene8859	VMAC	1.024284	0.661762	0.694753	0.968473	1.757264	2.048961	0.007049	1.069724	up
gene8739	LDLR	18.79735	16.84837	18.26317	10.03381	11.19204	11.59197	0.000133	-0.6513	down
gene8717	CDKN2D	2.240696	2.302668	1.881223	1.167554	1.02522	1.503115	0.032623	-0.71791	down
gene8715	ATG4D	12.45628	11.69809	11.73009	20.07225	19.83609	19.36763	4.53E-06	0.785931	up
gene8699	ICAM1	0.744855	0.583516	0.578937	3.766055	3.963993	4.007011	1.23E-21	2.686143	up
gene8679	FBXL12	1.468706	3.762554	4.284695	6.478699	6.033441	6.900114	0.001907	1.143925	up
gene8595	JUNB	8.73691	10.45005	9.423005	16.94137	13.36468	16.58476	3.15E-05	0.770204	up
gene8545	ASF1B	6.278074	6.123668	6.714884	2.958524	3.254832	3.624059	0.000218	-0.89243	down
gene849	RAP2B	5.995306	5.739767	6.113952	10.55804	8.891588	8.953241	2.12E-05	0.728964	up
gene8375	AKAP8	3.484319	4.102551	3.301219	6.981201	5.392575	5.890873	2.74E-05	0.746846	up
gene8316	HAUS8	9.312759	9.90508	8.642304	7.119604	6.016715	5.6237	0.004177	-0.59055	down
gene8308	ANKLE1	0.500207	0.486661	0.444891	0.114759	0.231764	0.152299	0.000464	-1.43036	down
gene8307	ABHD8	14.90787	15.40809	13.6194	52.58307	53.89713	55.52717	1.98E-31	1.976927	up
gene8289	FCHO1	0.041082	0.059878	0.077724	0.342473	0.629782	0.617352	1.49E-11	3.065941	up
gene8281	ARRDC2	0.547696	0.936351	0.877352	1.389681	1.236886	1.264974	0.014947	0.800872	up
gene8263	PGPEP1	2.493181	2.315084	2.338962	6.945499	3.896705	3.405727	8.96E-05	1.01297	up
gene8262	GDF15	0	0.069155	0.04054	7.279359	6.950543	5.590806	2.53E-42	6.972219	up
gene8257	ELL	3.054632	3.806638	3.177893	3.624613	5.64193	6.201522	0.000364	0.833076	up
gene8238	SLC25A42	0.541149	0.408382	0.448406	1.841648	2.067543	2.787741	1.08E-10	2.402887	up
gene8185	ZNF879	3.025102	3.027255	3.74214	6.288746	5.521955	6.339189	4.49E-06	0.818335	up
gene8128	BLOC1S4	7.100567	7.710164	6.542966	4.812364	3.578804	4.162073	0.002826	-0.71008	down
gene8078	SLBP	14.44691	15.92613	17.66169	8.369638	8.864975	10.10938	4.93E-05	-0.74221	down
gene8053	DGKQ	3.972465	2.799624	2.874798	6.210882	7.456139	7.024854	4.90E-09	1.181834	up
gene8040	HAUS3	8.955249	11.39623	9.433988	6.818618	3.785332	5.376264	2.16E-05	-0.90589	down

gene8035	RNF4	11.56539	14.22003	10.54643	9.302729	3.670316	13.28444	0.005961	-0.67351	down
gene7970	HSD17B11	1.901561	3.541183	2.586229	5.35909	2.820677	3.920765	0.044661	0.641339	up
gene7966	KLHL8	1.67301	3.100917	2.126314	1.217224	1.184729	1.963235	0.009345	-0.61169	down
gene7960	PTPN13	17.00196	18.98077	19.52148	27.6621	25.2975	25.92374	0.001137	0.619236	up
gene792	SMC4	27.66437	32.0267	31.00345	21.1389	16.61632	16.27767	0.000192	-0.71054	down
gene7901	CNOT6L	0.97182	1.83919	2.512763	6.14797	4.363225	1.500411	0.004287	1.182536	up
gene7896	CCNG2	2.850756	2.828006	2.828462	5.101991	4.527187	4.965357	0.000748	0.690099	up
gene7882	FAM47E	1.735254	2.50551	1.785588	3.137449	2.676651	3.138062	0.017319	0.695625	up
gene7850	CXCL3	0.829076	0.967266	1.039947	0.593355	0.560209	0.228291	0.037172	-0.85291	down
gene7716	CEP135	3.290369	3.56316	3.732471	2.072707	1.659996	2.107178	1.47E-06	-1.03829	down
gene7675	LRRC66	0.170509	0.365248	0.533278	1.668825	0.887418	1.493073	1.23E-06	1.733814	up
gene7510	NCAPG	38.96336	45.7821	44.42846	31.6151	20.28153	22.61639	0.000122	-0.75718	down
gene7467	BMPR1B	13.7569	11.59674	20.99035	17.84583	26.11091	18.32886	0.020036	0.59999	up
gene7412	BANK1	1.169625	1.051964	1.14374	1.483849	1.423247	1.485077	0.047042	0.608161	up
gene7410	SLC39A8	24.01701	25.57977	22.24243	55.38096	37.22757	47.57541	2.28E-07	1.012138	up
gene7409	SLC39A8	14.29433	11.9796	11.66953	29.31309	22.1012	26.88351	8.21E-07	1.097883	up
gene7390	CENPE	9.328205	10.0958	8.478071	4.591776	3.900284	4.316746	2.92E-12	-1.12202	down
gene7309	ZGRF1	3.569387	4.284202	3.717789	2.36909	2.06097	1.762208	5.66E-05	-0.82515	down
gene7213	CCNA2	25.64771	26.41305	17.6844	10.24686	6.97427	6.59298	4.43E-17	-1.42278	down
gene7208	TKTL2	0.303317	0.286152	0.175689	0.114376	0.283253	0.131779	0.042327	-1.09102	down
gene712	LOC101904810	0.183001	0.450781	0.169074	0.433517	2.921122	0.32206	0.001358	2.323891	up
gene7115	LOC101903200	2.651766	4.029823	3.190507	2.078852	2.874558	2.889654	0.015362	-0.77707	down
gene706	ECT2	10.99445	13.89227	14.00864	8.912682	6.098158	6.692631	1.61E-05	-0.78979	down
gene6931	BID	2.587712	2.737689	2.646648	1.728824	1.752986	0.872709	0.000133	-1.08911	down
gene6928	BCL2L13	48.06026	43.4414	43.74669	59.02055	73.22275	59.63124	0.002194	0.645877	up

gene6897	CCDC77	1.663466	2.017344	1.708376	1.095939	1.176639	1.010619	0.028016	-0.59311	down
gene6887	DDX11	3.361825	3.079364	3.192045	2.150792	1.916288	1.368424	0.000149	-0.90102	down
gene6881	FOXM1	2.405357	1.884387	2.006964	0.362381	0.877304	0.842269	9.00E-05	-1.50744	down
gene6880	RHNO1	11.53283	7.937124	12.8731	8.184679	6.152528	6.426708	0.010969	-0.60365	down
gene6877	TEAD4	7.108792	4.16821	5.362321	2.699086	4.78098	2.723271	0.007623	-0.65683	down
gene6859	RAD51AP1	3.662364	4.387672	4.364148	2.31473	2.124472	2.019578	0.000441	-0.91107	down
gene6856	LOC100336690	1.926437	1.933334	2.205163	1.102399	0.948186	0.188173	0.002587	-1.37071	down
gene6839	PLEKHG6	2.91544	2.592922	2.916152	2.455366	4.863337	4.310684	0.03127	0.598504	up
gene6713	KLRF2	0.739947	0.706615	0.752064	1.842439	1.276204	1.376273	0.001353	0.911447	up
gene666	ZMAT3	16.78509	9.234202	18.96619	23.18296	15.9207	18.44403	0.014833	0.601812	up
gene6619	LOC530773	18.3982	21.33084	19.68166	15.39488	9.78225	9.649515	0.010079	-0.71514	down
gene6517	FAR2	0.281487	0.403134	0.408548	0.875069	0.764422	0.981072	0.002228	1.036456	up
gene6401	MTERF2	0.470679	0.65373	0.658016	1.323917	1.586476	1.218532	0.000698	1.116226	up
gene6357	PARPBP	2.857958	4.643044	3.128496	3.236389	2.318361	2.181023	0.009991	-0.82309	down
gene6351	DRAM1	0.193333	0.246006	0.194723	0.504578	0.539793	0.375626	0.002534	1.206079	up
gene6335	GAS2L3	4.875959	5.092055	5.003855	2.132206	1.243118	1.569144	3.38E-12	-1.54374	down
gene6317	IKBIP	24.30638	26.84006	23.12416	46.43347	30.16977	33.77155	0.000464	0.616579	up
gene6314	TMPO	25.42625	31.4353	28.875	19.1045	14.23465	15.92625	1.47E-05	-0.82501	down
gene6158	CDK2	7.979313	8.53195	10.0465	6.185359	5.911141	4.917291	0.00551	-0.59032	down
gene6117	PRIM1	3.076213	3.981853	4.472106	3.328304	2.062317	2.042059	0.000444	-0.86657	down
gene6102	NEMP1	3.18531	3.391226	3.857779	2.016068	1.733959	2.039325	3.84E-07	-1.18151	down
gene6036	C5H12orf66	1.456432	1.76006	1.697901	0.854984	0.80372	1.103319	0.026476	-0.76674	down
gene5994	MDM1	1.367399	1.453878	1.43042	0.951675	0.883877	0.960085	0.002218	-0.81718	down
gene5986	MDM2	25.55241	26.18081	26.87702	194.2978	160.1104	159.1494	2.61E-43	2.921045	up
gene5902	AMIGO2	6.123428	6.422277	6.455153	4.053719	3.283857	4.002301	0.000684	-0.68663	down

gene5895	RAPGEF3	0.311201	0.273965	0.331209	2.959549	0.91692	2.215787	1.12E-15	2.652284	up
gene5815	TROAP	6.685071	5.012791	6.176778	2.705543	3.251647	3.215429	0.000148	-0.90328	down
gene5782	LOC619094	1.945813	1.990752	2.154173	1.121063	0.7918	0.966163	0.010688	-1.01965	down
gene5696	ITGB7	0.172866	0.318682	0.277701	0.699157	0.443381	0.471055	0.040906	0.806165	up
gene5693	ESPL1	8.424942	7.082206	8.075151	3.341469	5.006079	4.677915	0.00012	-0.79838	down
gene5648	ZNF385A	2.829148	2.876869	2.97398	5.490699	10.54909	8.91681	1.62E-07	1.563388	up
gene5598	BTG1	27.83222	32.25333	34.10863	103.939	70.26447	79.46655	7.99E-14	1.484008	up
gene5569	KITLG	4.60285	5.776688	5.657367	13.10511	8.914527	9.586798	1.80E-09	1.068372	up
gene5568	TMTC3	11.79354	14.69856	13.5581	26.43436	15.74875	18.71008	0.001578	0.655907	up
gene5450	PAXIP1	4.038065	2.642995	2.877772	1.643913	2.543883	2.192408	0.000226	-0.94374	down
gene5441	XRCC2	1.70409	2.04717	1.763592	1.051493	0.780428	0.961746	0.002037	-0.90871	down
gene5378	ZBED6CL	0.606653	0.391296	0.404866	0.543186	1.025435	0.745989	0.036246	0.797488	up
gene5370	KRBA1	1.95845	1.676463	1.794263	2.472525	3.552147	3.457131	3.64E-05	0.925125	up
gene5342	EZH2	6.295283	6.284856	5.773555	2.56355	4.164737	2.883592	2.03E-12	-1.29692	down
gene5337	CNTNAP2	3.75613	1.373271	0.870329	4.541015	2.974773	4.472385	0.02077	1.005905	up
gene5293	CASP2	9.784668	7.159235	8.908744	3.986631	5.637144	6.147667	0.00123	-0.71748	down
gene5066	UBE2H	7.006016	6.814852	7.062409	11.70353	11.65414	12.97967	1.68E-06	0.875734	up
gene497	FAM43A	5.671045	5.52936	5.683419	13.82849	11.38021	13.92164	2.63E-13	1.269506	up
gene4807	PRR15	5.397696	5.087924	5.329492	3.439588	3.162125	3.196214	0.001491	-0.62713	down
gene4806	WIPF3	2.421955	2.017837	2.376649	0.638173	0.943459	2.049876	0.022991	-0.83243	down
gene48	HUNK	0.27318	0.339561	0.331277	0.163121	0.136314	0.207991	0.024915	-0.83408	down
gene4755	KIAA0895	1.916306	2.205389	1.86664	1.273626	0.980087	1.362568	0.015301	-0.65196	down
gene4735	IMMP2L	3.407318	4.975433	2.303127	2.714751	8.082848	4.676579	0.015363	0.633405	up
gene4731	C4H7orf60	5.019381	5.895829	6.20148	11.82177	8.801295	10.09741	1.57E-06	0.847972	up
gene4710	MET	4.188875	4.340566	4.787038	8.940685	6.987492	6.558216	5.25E-05	0.681804	up

gene47	MIS18A	4.314513	6.636694	5.05862	3.656695	2.446716	2.333945	6.27E-05	-0.85112	down
gene4660	SYPL1	18.74124	22.56446	20.66177	23.24818	6.744039	8.602591	0.045067	-0.65051	down
gene4646	SRPK2	6.561656	6.496209	4.792704	5.514757	4.363542	4.384995	0.000284	-0.59096	down
gene4624	GSAP	0.51116	0.770864	0.487529	1.147936	0.757041	1.049517	0.04709	0.629313	up
gene4561	LOC100296627	1.769993	0.843519	1.020165	1.681402	2.594742	2.419263	0.019334	0.749721	up
gene4557	DBF4	5.016275	5.687445	5.190138	3.050232	1.533634	2.299665	5.74E-06	-1.0331	down
gene4433	SHFM1	13.92653	14.47653	11.02604	19.59803	25.46449	17.38405	0.007914	0.723486	up
gene4367	C4H7orf57	0.40217	0.278058	0.286829	2.475844	2.225525	2.292231	1.01E-14	2.574189	up
gene4348	FIGNL1	2.511356	3.12106	3.085847	1.068753	0.669031	0.953253	8.10E-08	-1.55256	down
gene4213	HJURP	12.90918	11.67092	12.54273	2.463889	2.224222	2.55078	3.45E-34	-2.29044	down
gene4195	INPP5D	0.012309	0.006214	0.010675	0.160122	0.195507	0.212825	4.81E-15	4.194937	up
gene4172	LOC101907642	0.999567	1.465433	1.331774	1.145823	1.295285	1.39856	0.021076	0.917306	up
gene4167	ZMYM1	5.734062	7.064783	6.373488	4.891465	2.747292	3.518207	0.000135	-0.779	down
gene4157	CLSPN	3.606878	3.217004	3.074839	1.292982	1.470513	1.092144	1.73E-07	-1.27444	down
gene4130	ZC3H12A	1.158232	1.363773	1.292894	2.53896	2.611783	3.060493	2.91E-06	1.269216	up
gene4123	CDCA8	11.79475	10.34192	11.77838	4.720889	3.785131	3.92099	8.62E-12	-1.39104	down
gene4092	HPCAL4	0.195239	0.190717	0.179024	0.392015	0.253185	0.402741	0.01154	0.930232	up
gene4084	MFSD2A	2.247546	2.620598	2.987848	1.952764	1.329966	1.558616	0.038636	-0.59785	down
gene4070	EXO5	4.005902	4.133738	4.51368	27.40561	19.90604	18.65738	6.93E-33	2.384513	up
gene4064	NFYC	6.379361	8.775804	8.609594	11.73598	11.18073	9.770502	1.04E-05	0.762114	up
gene4034	ZNF691	5.028293	6.661908	6.674101	8.128093	8.497872	10.73227	0.00069	0.663847	up
gene4009	CDC20	27.26911	28.74381	27.87497	10.03128	8.081583	8.641989	1.06E-20	-1.59092	down
gene3974	KIF2C	11.77483	12.36938	11.20586	5.811912	6.054235	5.381358	3.20E-07	-0.95964	down
gene3950	NASP	22.72069	12.42718	13.14179	10.9828	8.843007	13.03452	0.000144	-0.61859	down
gene3946	IPP	2.558869	2.087432	2.514448	1.863697	1.413613	1.35561	0.022424	-0.69388	down

gene394	POLQ	4.024393	4.890099	5.376238	2.864291	2.392162	2.903934	0.000433	-0.6079	down
gene3939	RAD54L	3.816282	3.690834	4.337848	2.249413	2.392335	2.384847	2.67E-05	-0.89213	down
gene3910	STIL	2.43826	3.035119	3.908414	0.896457	1.081156	0.882876	1.43E-11	-1.64833	down
gene391	GTF2E1	3.413351	5.452854	5.667407	3.174914	2.407968	3.196121	0.009639	-0.78308	down
gene3888	CDKN2C	14.07906	14.82876	14.47052	8.12703	6.92815	7.196881	9.83E-07	-0.91789	down
gene3865	ORC1	3.04135	3.593602	3.834931	1.653106	1.743458	1.416795	2.72E-05	-1.06185	down
gene3860	LOC520518	3.551997	3.282943	3.258577	1.789533	1.623052	2.231003	0.007067	-0.79635	down
gene386	LRRC58	12.49599	17.12554	12.62998	6.40006	14.04571	9.871834	0.047565	-0.80306	down
gene3857	ECHDC2	7.432768	6.976483	6.009272	14.39381	12.06622	14.65852	3.44E-16	1.532593	up
gene3761	USP1	16.7201	19.87982	20.76126	13.83009	9.728989	10.00653	6.19E-05	-0.70194	down
gene3712	GADD45A	40.68629	36.41402	38.31083	70.88255	67.69214	60.0293	6.99E-07	0.830388	up
gene3710	DIRAS3	0.504311	0.378317	0.450527	1.757337	0.826926	1.115946	0.00035	1.450017	up
gene3703	LOC107132322	3.250346	4.454196	4.457723	2.325883	1.260063	1.811586	1.09E-05	-1.13087	down
gene3702	LOC527397	3.684476	5.112447	5.556176	2.338995	1.813272	1.83735	6.18E-07	-1.20874	down
gene3615	SYDE2	0.352637	0.377699	0.377354	0.685489	0.589489	0.557348	0.022057	0.756421	up
gene3471	SASS6	2.695665	3.25131	2.840726	2.149238	2.606015	1.943588	0.000345	-0.7083	down
gene3414	GPSM2	8.342044	9.429374	8.750817	5.564069	4.597112	5.71294	0.000314	-0.68644	down
gene3404	PSRC1	4.909148	5.024465	4.318523	16.64919	14.66019	16.27838	1.35E-21	1.839279	up
gene3379	CSF1	5.37433	4.218191	4.394815	10.90691	18.44191	15.81341	8.80E-15	1.757562	up
gene3340	FAM212B	0.369668	0.443792	0.382479	2.37219	4.352422	3.617	1.96E-16	3.172371	up
gene3304	DCLRE1B	2.399661	2.902892	2.127501	9.113678	37.208	7.878731	1.36E-09	3.046274	up
gene3282	NGF	5.225329	3.783018	4.243056	14.67258	11.22859	9.245259	6.52E-09	1.513183	up
gene32423	LOC615899	0.358675	0.454786	0.365862	0.748824	0.539058	0.682156	0.027801	0.794029	up
gene32377	LOC510961	0.919938	1.307755	0.843328	2.372369	1.725651	1.865158	0.002974	1.083645	up
gene3218	LOC788724	32.67104	27.93315	33.23177	12.64287	24.63018	12.0207	0.004906	-0.84328	down

gene32172	ASB11	1.24093	1.138929	1.172325	1.668181	1.65369	2.49593	1.17E-06	1.136746	up
gene32162	ZRSR2	2.576048	2.549936	2.319249	1.616465	1.779685	2.114487	0.000242	-0.8637	down
gene3210	GJA5	7.94233	8.314336	8.347241	5.455759	4.318217	5.909641	0.001769	-0.59285	down
gene32098	RPS6KA3	3.501089	5.783711	4.713206	4.110925	4.469339	2.949195	0.010756	-0.62224	down
gene32054	PCYT1B	3.44869	3.618969	1.887799	2.767372	3.486249	3.453687	0.023	0.610132	up
gene31740	NUDT11	0.90783	0.913623	0.8646	2.273609	2.286458	2.306549	1.32E-07	1.61615	up
gene31738	NUDT10	0.046697	0.25382	0.127459	1.89141	0.99295	1.09396	3.37E-10	3.214649	up
gene31678	SUV39H1	6.415011	4.493427	5.512521	2.69098	2.80318	2.798141	1.03E-05	-0.97628	down
gene31662	CSNK1B	0.942679	1.535562	1.085563	1.612882	1.255739	1.525218	0.037081	0.596313	up
gene31596	KIF4A	7.810634	8.89721	9.088207	5.612999	4.981637	4.595418	8.96E-05	-0.69883	down
gene31553	ERCC6L	4.246966	4.570543	5.125643	2.257311	2.228047	1.907099	1.33E-07	-1.11788	down
gene31526	KIAA2022	3.090197	2.824408	2.682338	7.236743	6.068273	6.46491	6.58E-13	1.228733	up
gene31072	SPRY3	0.187653	0.113286	0.233308	0.368978	0.272253	0.250766	0.041229	0.801311	up
gene30911	ARHGEF6	2.308594	2.535477	3.593297	4.954096	3.948164	4.93296	0.000596	0.706326	up
gene3084	HIST2H2BF	55.88183	74.86159	64.44193	46.4555	24.7869	26.25791	3.33E-05	-0.95155	down
gene3083	LOC504599	24.57056	18.37983	24.89216	9.712016	8.360706	7.943654	1.51E-06	-1.32304	down
gene3081	HIST1H2AK	41.9375	75.54108	77.21534	48.43973	18.02215	29.51345	0.000967	-0.97608	down
gene3080	H2B	22.14235	31.41376	26.49289	9.3316	5.090679	5.666301	3.89E-14	-1.94139	down
gene308	PLCXD2	0.740265	0.962194	1.147686	2.074467	2.343338	2.129463	3.98E-07	1.256616	up
gene3078	HIST1H2BL	6.836293	5.657242	5.231015	1.966865	1.611765	0.924701	4.20E-07	-1.90588	down
gene3077	HIST2H2AA4	107.9448	86.18662	65.36346	25.23245	20.04817	23.47536	5.85E-18	-1.86353	down
gene3076	LOC516742	79.2038	94.35586	74.0139	28.46529	39.11546	25.84183	3.20E-10	-1.32914	down
gene3074	HIST2H2AB	10.79024	11.43763	10.11983	4.969129	4.914296	3.841758	1.61E-05	-1.17151	down
gene3071	HIST2H2AC	49.98314	55.21176	50.59554	25.65699	17.35426	18.60548	2.98E-09	-1.28336	down
gene30631	SLC25A22	5.414189	4.167148	4.360089	2.496985	1.864582	3.293588	0.002185	-0.81084	down

gene30628	PIDD1	1.401185	1.277082	1.133454	1.739192	3.363142	2.683313	0.000421	1.126591	up
gene30516	POLD4	6.319127	7.938349	7.663	14.9078	9.920523	12.37682	0.000281	0.836634	up
gene30440	PCNX3	95.68337	7.761721	10.09551	4.977424	9.171849	9.609856	0.001872	-2.16525	down
gene30426	TIGD3	0.565679	0.486844	0.649638	0.198946	0.165981	0.192274	0.000207	-1.5298	down
gene30423	POLA2	6.672429	6.567564	7.150423	3.808112	4.045243	3.617324	0.000275	-0.77965	down
gene30410	CDCA5	4.796615	2.878531	5.1894	2.533945	2.844446	2.733527	0.009012	-0.70375	down
gene30408	SAC3D1	1.814371	1.526324	1.573126	3.834816	4.683467	4.903206	2.92E-10	1.58933	up
gene30307	INTS5	4.144672	4.289638	2.931974	1.878908	1.462548	2.86264	0.003589	-0.82125	down
gene30292	INCENP	5.947236	4.61096	5.940861	2.837802	2.067087	3.310838	2.13E-09	-1.10342	down
gene30282	FEN1	14.85706	16.38719	16.04585	6.196041	6.634147	5.919162	8.18E-11	-1.26551	down
gene30270	TMEM138	2.712322	3.898089	2.353465	1.328653	1.456767	2.092316	0.008238	-0.68174	down
gene3024	MLLT11	13.69064	3.825675	2.929549	20.38753	18.16099	17.84178	0.000184	1.502907	up
gene30123	HYLS1	4.945756	5.986974	5.868381	2.292053	2.065582	1.823581	3.35E-07	-1.38644	down
gene30115	CHEK1	1.636192	2.964463	5.085515	0.964201	1.847537	0.918371	0.000767	-0.79504	down
gene29969	HPS5	12.69137	14.91187	13.79637	27.16299	21.0394	22.33689	6.99E-06	0.829896	up
gene29952	E2F8	4.587218	5.026929	4.985952	0.805817	0.794097	1.311332	1.11E-23	-2.38304	down
gene29874	DDIAS	4.163854	4.904145	4.351096	11.07055	8.461942	8.727202	1.03E-08	1.067111	up
gene29834	CTSC	2.505414	2.619884	2.938505	5.003048	4.012636	3.686633	0.002454	0.756986	up
gene29650	PPIF	4.736325	3.980458	3.767061	6.631735	7.604751	7.322468	0.005858	0.854289	up
gene29601	DNAJC9	6.705002	4.12092	7.240485	5.518062	4.523681	3.60003	3.28E-07	-1.14793	down
gene29582	DDIT4	8.837511	7.322961	8.375907	49.51752	57.5051	49.53761	2.08E-47	2.738339	up
gene29571	SLC29A3	1.548869	1.050629	1.211141	1.07077	2.337525	1.368583	0.038234	0.656224	up
gene29569	LOC107131186	1.046484	1.279343	1.048658	1.599821	1.402237	2.05403	0.033082	0.706894	up
gene29539	HKDC1	0.040928	0.034542	0.028328	0.392915	0.234012	0.405046	3.39E-12	3.340431	up
gene29530	STOX1	0.117034	0.05948	0.081681	1.709577	1.132697	1.446677	9.50E-22	3.701033	up

gene29524	DNA2	2.981442	3.030645	2.70303	1.394915	1.046913	1.069765	1.18E-05	-1.1256	down
gene29489	LOC100139549	1.830616	2.054675	0.865331	0.880701	0.555614	0.662068	0.01032	-1.12512	down
gene29482	TMEM26	3.572306	3.140477	3.481848	2.398515	1.176325	3.330522	0.004096	-0.68541	down
gene29478	CDK1	20.83426	25.39398	20.70114	18.05173	13.25912	12.95242	0.000392	-0.6711	down
gene29422	EDARADD	0.260598	0.442656	0.153704	3.622024	3.006279	2.763375	2.26E-16	3.497189	up
gene29324	RAB4A	2.062747	2.035074	1.906981	3.646986	3.415865	2.13767	0.00868	0.59731	up
gene29302	gene29302	32.11673	33.07024	31.56152	18.01462	20.87465	17.40345	5.17E-05	-0.72749	down
gene29260	GINS4	3.746068	3.092605	3.343117	1.991063	2.048142	1.657645	0.002697	-0.77409	down
gene29206	LOC101907229	0.99507	0.876986	1.173592	0.991148	2.137372	1.420646	0.032605	0.66451	up
gene29190	RNF122	1.662358	1.63354	1.28362	4.210009	4.219841	4.399943	3.65E-08	1.524399	up
gene29118	MICU3	2.939793	6.588646	3.224789	8.088036	5.814583	4.929534	0.020325	0.628262	up
gene29059	PRIMPOL	3.599459	4.692057	6.2588	3.75654	2.868121	2.871807	0.00165	-0.66284	down
gene29029	NEIL3	3.369157	4.378097	2.81332	1.720742	1.400206	1.083629	7.94E-07	-1.46709	down
gene28961	LOC782601	0.975775	1.711192	2.097004	0.869547	0.037921	0.017495	0.011276	-2.27758	down
gene2891	LOC782145	0.807788	0.597018	0.86606	1.967894	2.56962	1.693273	6.78E-08	1.532992	up
gene28901	MKI67	19.9153	17.78229	19.19094	7.861114	12.62275	10.60784	0.000772	-0.77476	down
gene28891	FAM196A	0.048454	0.209164	0.067302	0.163252	0.201636	0.209448	6.49E-06	1.770566	up
gene2888	UBE2Q1	12.1913	12.15382	12.48524	19.32702	17.08773	16.92731	7.37E-05	0.625986	up
gene287	KIAA1524	5.367255	6.964338	6.3693	5.011893	3.128503	3.496493	0.004494	-0.60006	down
gene2869	EFNA1	2.217824	2.014223	2.014804	1.273935	0.993097	1.355586	0.030097	-0.70023	down
gene28663	TAF5	2.706837	2.921813	2.33443	1.974134	0.850255	1.54942	0.004513	-0.82261	down
gene28630	PPRC1	8.556783	7.395404	6.590784	3.092851	5.288779	5.174249	0.002001	-0.73429	down
gene28592	BLOC1S2	12.4664	13.54626	13.05716	35.74081	21.20074	26.34372	8.84E-08	1.14161	up
gene28508	HELLS	4.670081	5.840496	5.044602	2.835154	2.449046	2.020298	6.72E-12	-1.26431	down
gene28494	CEP55	11.10536	11.87603	12.34074	4.878159	3.839068	4.227211	1.54E-12	-1.41464	down

gene28484	KIF11	12.85907	15.14705	14.57155	7.664814	5.788798	6.591574	9.81E-11	-1.07988	down
gene28461	KIF20B	5.480635	6.701449	6.772538	2.710793	2.359993	1.789945	2.87E-17	-1.63736	down
gene28446	FAS	6.493851	7.109157	7.630147	10.71624	8.772499	10.69225	0.000573	0.596153	up
gene28386	FAM20C	11.4731	9.94018	10.24567	14.43686	16.58477	17.60239	0.000239	0.683321	up
gene28361	MICALL2	3.701514	4.607056	4.205836	7.168939	8.678893	9.599528	3.45E-13	1.377065	up
gene28358	MAFK	8.930553	6.842256	10.67423	6.348936	4.571312	4.999964	0.00145	-0.74779	down
gene28127	HSPB1	207.6748	196.7186	201.1738	337.553	471.4812	191.6121	0.005678	0.802768	up
gene28102	DNAJC30	4.78767	5.113268	5.039296	2.63331	3.728205	1.879824	0.036208	-0.71269	down
gene2804	MRPL24	0.89824	0.606564	0.436128	26.77833	22.14274	23.47404	7.25E-63	5.2815	up
gene2803	HDGF	63.0806	55.10922	70.16335	68.43564	25.23665	54.18284	6.57E-05	-0.7891	down
gene28028	SUMF2	8.41402	6.569264	5.40688	0.245924	1.056835	0.20765	2.81E-11	-3.61575	down
gene28026	PSPH	17.51052	20.57805	20.05739	71.46233	52.66796	51.99476	7.40E-22	1.6567	up
gene27998	PRSS53	1.128225	0.865263	1.075314	1.283835	1.746165	1.688152	0.027352	0.679504	up
gene27952	KIF22	19.34061	19.28281	19.39562	11.19135	10.1208	9.734097	8.39E-07	-0.84082	down
gene27896	SBK1	0.264165	0.343862	0.636554	1.431054	5.738899	5.361286	4.40E-10	3.399528	up
gene27891	KIAA0556	0.383512	0.560223	0.079706	0.125442	1.396961	1.138084	0.04701	1.455198	up
gene27858	PLK1	9.533493	9.58859	11.74906	3.29611	2.851108	3.599178	1.32E-16	-1.7076	down
gene27856	PALB2	2.518599	2.952145	4.116147	2.103693	1.263163	1.217432	2.82E-06	-1.15176	down
gene27755	NTAN1	24.83503	30.25531	28.37159	50.69177	35.30724	39.11531	1.53E-06	0.788335	up
gene27662	ZNF500	1.108996	0.844372	1.780888	2.296839	1.556023	1.558463	0.012657	0.7781	up
gene27644	TFAP4	2.238762	2.221134	1.08805	0.637155	0.685862	0.446109	0.00015	-1.4744	down
gene27543	CCNF	9.196697	7.403889	9.430008	1.431865	1.958604	1.897814	5.41E-22	-2.24177	down
gene27500	NUBP2	0.72741	1.233996	0.859545	1.013074	7.616508	10.41751	2.50E-05	2.195465	up
gene27452	RHBDL1	5.151034	4.562673	3.856916	11.74047	12.41582	12.50558	1.79E-11	1.509394	up
gene27417	LOC789192	1.940898	2.560692	6.161492	6.34617	16.47078	4.858244	0.001733	1.482852	up

gene27326	C24H18orf54	4.220815	3.349077	5.110571	1.835906	2.018846	2.352361	9.38E-07	-1.0172	down
gene27309	SKA1	5.567568	7.242844	6.803947	3.905211	2.53486	2.189531	0.000168	-1.07432	down
gene27249	CEP192	5.696863	5.938656	6.654707	3.937984	3.516856	3.735926	5.27E-05	-0.65953	down
gene27227	GNAL	0.203067	0.316237	0.381875	0.524516	0.397396	0.577754	0.043598	0.768251	up
gene27173	NDC80	9.424047	10.82841	11.1807	6.109742	4.5604	6.013945	4.85E-06	-0.8531	down
gene27172	METTL4	2.044733	2.054352	1.892278	1.37818	0.878343	1.006793	0.036357	-0.63882	down
gene27167	YES1	14.62983	8.149799	7.163755	19.47022	11.87823	14.16861	0.013042	0.644099	up
gene27149	ABHD3	1.580121	1.845928	2.089878	4.316287	2.689331	2.947054	0.003106	0.902489	up
gene27131	RIOK3	11.30631	6.028637	5.842034	16.6931	10.82061	12.25729	3.50E-10	1.224204	up
gene27125	LAMA3	0.41972	0.358395	0.433316	0.68626	0.600564	0.761061	0.009379	0.811343	up
gene27097	DSC3	27.63645	25.60112	27.6951	43.39152	37.00875	30.21341	0.010435	0.62195	up
gene27092	DSG3	0.079008	0.090668	0.079077	0.396862	0.325532	0.311656	3.60E-07	2.085659	up
gene26966	RBFA	13.01411	14.98994	5.879495	4.595138	4.935849	5.895624	0.009097	-0.59963	down
gene2684	VSIG8	0.279013	0.97828	0.227827	1.388415	1.165048	0.832234	9.43E-09	2.139028	up
gene26812	LOC520966	5.372468	7.543448	6.618686	5.354577	9.211221	6.429339	0.001866	0.960976	up
gene26719	GMNN	9.556956	10.51229	13.7036	8.342924	5.4265	4.28481	0.002543	-0.84122	down
gene26700	HIST1H1A	19.5982	20.40601	17.0404	6.938142	5.174496	5.828619	1.88E-12	-1.61193	down
gene26699	HIST1H3I	13.01768	13.97653	15.14338	7.456387	5.715933	6.238932	1.82E-07	-1.06035	down
gene26698	H4	40.93214	49.43129	43.78564	36.11213	18.57443	22.72253	0.000271	-0.74611	down
gene26697	HIST1H3G	44.5639	49.17888	47.36273	25.10558	17.2247	19.14376	1.09E-11	-1.14428	down
gene26696	LOC104975684	90.68086	102.8834	92.72456	48.09808	26.71175	31.3723	1.40E-11	-1.38175	down
gene26695	HIST1H2BB	6.307462	6.836445	5.302424	3.75509	1.806707	2.135522	0.000727	-1.21178	down
gene26691	LOC617905	56.52662	96.27315	60.1126	50.9213	30.87038	33.60848	0.00057	-0.8293	down
gene26690	LOC104975686	0.1915	0.206683	0.297554	0.596335	0.300058	0.307951	0.035311	0.832379	up
gene26685	HIST1H1E	100.592	108.0774	103.2883	63.79836	53.93584	56.81085	1.04E-06	-0.77835	down

gene26683	LOC787465	10.8422	12.7693	12.34165	6.974582	3.170337	0.083822	0.023676	-1.76666	down
gene26682	LOC617875	156.0333	175.5584	153.3326	117.1284	75.89127	77.8624	0.00011	-0.78748	down
gene26681	HIST1H1E	3.245654	3.248383	4.263042	0.680488	0.610858	1.042695	5.35E-10	-2.13855	down
gene26680	LOC104975683	36.27118	40.59692	41.8874	20.53336	11.36524	12.91535	9.20E-09	-1.35521	down
gene26679	LOC505183	23.91195	52.74565	46.9917	26.99669	11.97308	14.77439	0.000193	-1.15178	down
gene26678	H2B	89.81374	97.8522	93.66628	43.20693	28.32789	29.74862	2.74E-14	-1.42054	down
gene26675	HIST1H1D	28.7265	19.55099	33.48358	16.97383	13.97483	16.34025	0.000745	-0.73384	down
gene26672	LOC104968456	70.62025	77.82351	72.45001	0.918336	24.11347	29.19164	0.005735	-1.93206	down
gene26671	LOC107131750	56.69949	62.14802	54.49214	23.08224	24.57818	25.33994	2.60E-10	-1.18232	down
gene26670	HIST1H2AM	71.15081	93.19544	80.98942	40.59818	27.40306	30.62256	1.81E-11	-1.25927	down
gene26669	HIST1H2BM	10.87081	12.26503	11.38495	6.697021	3.829645	5.273175	4.35E-06	-1.07656	down
gene26668	LOC527388	113.5166	123.8574	139.1321	75.96729	51.27469	49.26622	5.96E-07	-1.03792	down
gene26640	HMGN4	6.750121	8.945187	1.380027	0.912953	1.047714	1.401812	5.17E-06	-2.2789	down
gene26604	HIST1H2BJ	16.37961	19.31117	16.95224	5.657251	3.93641	4.473042	1.76E-17	-1.84764	down
gene26601	HIST1H2BN	131.133	166.5699	149.6838	94.56644	53.82723	57.40272	1.71E-07	-1.06939	down
gene26600	HIST1H2AH	50.0587	68.39382	64.93979	28.21174	17.82164	18.70235	1.22E-11	-1.44677	down
gene26517	HIST1H2AJ	74.84727	81.04572	76.09858	43.09047	30.84284	32.36416	2.54E-10	-1.07083	down
gene26516	LOC616819	141.9202	144.2366	126.2776	64.87022	42.06446	42.24799	1.94E-13	-1.4151	down
gene26515	HIST1H2AG	167.5035	183.1862	170.4369	96.6685	66.88615	73.35454	3.04E-10	-1.083	down
gene26512	LOC616868	116.8577	130.5947	109.8315	49.00542	34.21386	36.70269	1.06E-16	-1.52012	down
gene26511	LOC614970	71.74265	84.79659	71.15036	32.73359	22.47272	24.08944	1.31E-14	-1.46691	down
gene26509	LOC104975676 、histone H2B type 1	43.14587	57.1804	43.9972	22.42329	9.552156	10.98818	5.10E-09	-1.70368	down
gene26508	LOC529277	18.19672	21.37085	18.44886	10.90684	6.650647	7.556957	1.38E-06	-1.15552	down
gene26507	HIST1H1B	194.0983	202.7133	196.2895	88.00745	72.83057	70.82536	6.95E-12	-1.29719	down

gene26506	LOC528329	56.15816	62.13531	52.96574	31.82189	20.42507	22.18669	1.70E-08	-1.15026	down
gene26505	HIST1H3C	28.28318	36.13792	15.72776	0.335476	11.28449	13.82933	0.037052	-1.56196	down
gene26503	HIST1H2BI	26.78329	31.76352	29.75257	14.26338	5.979124	8.032488	4.57E-09	-1.60042	down
gene26322	JSP.1	7.726751	6.509248	8.951711	12.86336	12.66228	12.45062	0.008102	0.605225	up
gene2632	PVRL4	0.068498	0.144663	0.064389	1.001257	1.593501	0.801488	3.42E-19	3.320317	up
gene26295	IER3	69.05473	67.45494	67.94454	151.4398	121.6266	116.4442	2.01E-07	0.987528	up
gene26276	TCF19	6.276978	5.977744	5.61374	2.264279	2.26323	2.45047	2.65E-07	-1.22324	down
gene2622	B4GALT3	6.35881	5.391539	6.174446	10.12244	9.066996	9.389729	0.000318	0.731341	up
gene26155	PAQR8	0.403418	1.020096	0.913768	0.56542	0.078556	0.0599	0.01158	-1.69825	down
gene26151	MCM3	40.28912	42.10694	43.20813	26.16435	26.55954	22.2977	0.000327	-0.67764	down
gene26133	C23H6orf141	3.04163	1.79239	2.694468	1.10185	0.972193	1.210876	0.006809	-1.00426	down
gene26080	POLH	8.819088	9.114203	9.020012	6.186154	5.404132	6.038124	7.18E-05	-0.64914	down
gene26076	LRRC73	1.297134	1.520782	1.138685	1.394398	2.524391	2.391681	0.021805	0.771725	up
gene26017	FOXP4	11.63723	8.860019	9.246821	8.579673	19.36698	18.454	0.0127	0.696275	up
gene25955	CDKN1A	95.17563	89.45563	90.67801	850.6413	916.1058	787.3771	1.10E-48	3.285273	up
gene25922	DEF6	1.729695	1.915645	1.842644	2.842496	3.116663	3.027013	0.00289	0.807113	up
gene25859	TAP1	4.984671	5.191795	5.053925	8.458832	9.422459	8.055794	2.34E-05	0.835642	up
gene25791	ABTB1	12.34024	12.75372	12.57975	14.30888	18.97886	22.09954	0.001109	0.64448	up
gene25749	XPC	9.100693	11.87519	8.356882	19.77188	21.53282	21.85602	4.84E-13	1.482003	up
gene25745	SLC6A6	9.673138	8.291994	9.383793	12.64932	15.83367	14.56615	3.11E-05	0.733369	up
gene25652	ALS2CL	6.348457	5.60136	6.7444	9.536838	10.30429	10.28826	5.73E-06	0.801869	up
gene25632	ELP6	2.088243	2.924566	2.779581	0.99868	1.743986	1.478219	0.003719	-0.7701	down
gene25625	CDC25A	5.833452	4.929343	4.454095	1.887206	2.018009	2.272975	1.24E-09	-1.48011	down
gene25557	AMIGO3	9.447263	7.252356	5.871845	5.70E-05	0.000105	6.60E-05	########	-10.8774	down
gene25549	TRAIP	1.107548	1.323542	1.369296	0.598401	0.69412	0.551258	0.002175	-0.99919	down

gene25517	CISH	0.457272	0.188713	0.38784	0.835701	0.963052	0.667399	0.002399	1.314456	up
gene25497	PCBP4	27.71099	26.58729	28.46541	40.95014	41.21177	37.83206	0.000277	0.588891	up
gene25492	DUSP7	4.174562	5.665657	4.658425	2.232067	3.343912	2.96747	0.008333	-0.58791	down
gene25434	ERC2	0.860052	1.307847	1.628068	2.140016	2.408111	2.121594	0.014785	0.735831	up
gene25409	ABHD6	2.361775	2.219712	2.244235	4.417805	3.095443	3.000058	0.013277	0.675818	up
gene25302	SETMAR	1.173671	1.018639	1.508921	2.221352	1.421704	2.010919	0.017624	0.661599	up
gene25275	RAD18	6.015958	7.834457	6.594613	6.310898	4.362691	4.240949	0.001982	-0.69231	down
gene25260	TTLL3	11.92168	2.503446	11.90442	3.90629	2.535193	2.952464	0.005204	-1.10247	down
gene25242	KIF15	3.310465	3.950058	3.580906	1.768736	1.578261	1.741151	5.93E-06	-0.94662	down
gene25231	LOC101902478	2.636949	27.55615	6.473678	31.99989	27.69853	18.18891	0.040842	1.164813	up
gene25024	CDCA4	6.929254	5.696428	5.418627	2.218583	1.882653	2.776911	3.13E-08	-1.31875	down
gene25019	CEP170B	10.07386	8.407684	8.628193	10.41871	16.71721	16.21982	0.001344	0.746987	up
gene2493	EPHA2	30.73406	24.40753	26.81408	29.64281	46.55773	44.82463	0.005595	0.623891	up
gene24769	WDR76	3.758032	3.356569	3.145713	3.472256	2.021352	2.209884	0.005113	-0.63812	down
gene24745	MIS18BP1	14.1182	15.71375	14.6267	7.035741	5.94363	5.555749	2.85E-13	-1.2671	down
gene24739	KLHL28	2.562309	2.957021	2.494082	6.864803	4.687076	6.102731	1.08E-14	1.292298	up
gene24710	LOC782598	1.063747	1.052726	1.285066	0.577773	0.430542	0.383127	3.36E-05	-1.2293	down
gene24633	G2E3	5.792909	7.285587	9.685116	6.041439	4.014494	4.663887	0.000809	-0.61562	down
gene24566	C21H15orf39	2.61151	1.965006	2.270434	0.52784	1.2983	1.35851	0.004586	-0.99612	down
gene24534	FBXO22	6.946114	8.162038	7.391536	14.10162	10.30993	10.85277	0.000228	0.73239	up
gene24513	LOC530472	2.670574	2.639684	2.352764	1.514598	1.370764	1.479154	0.014067	-0.73018	down
gene24484	KLF13	5.229181	4.456657	1.153695	2.690805	0.167289	0.713486	0.028657	-1.58157	down
gene24393	BLM	3.075057	3.734856	3.962116	1.934662	1.602533	1.747036	3.08E-06	-0.96642	down
gene24384	PRC1	21.274	22.75163	23.86842	14.08812	10.98647	11.64567	5.72E-08	-0.86053	down
gene24365	TICRR	4.192943	3.901605	4.569524	0.709501	0.838126	0.908481	2.01E-24	-2.2811	down

gene24310	AEN	21.79865	21.69245	22.38316	45.33801	40.48129	43.41544	1.53E-08	1.032267	up
gene242	SGOL1	4.454722	5.314743	4.614219	1.102584	0.911761	1.134343	2.39E-17	-2.1087	down
gene24183	CEP72	4.963348	3.912502	4.231677	2.395394	3.277272	2.452967	0.000692	-0.80466	down
gene24032	DNAJC21	9.563891	11.11547	10.20747	23.04791	18.19023	17.50485	4.43E-09	0.986031	up
gene24022	SKP2	4.091286	3.503149	4.034682	1.68681	2.251173	2.336465	0.00046	-0.7906	down
gene23945	PELO	0.608113	0.86391	0.487996	1.06008	0.632252	3.76176	0.006596	1.513772	up
gene23909	IL31RA	0.079712	0.0937	0.092755	0.241251	0.25035	0.287812	6.48E-05	1.595242	up
gene23891	PLK2	8.2296	8.79014	8.363008	59.75299	40.14448	44.5917	7.06E-35	2.49893	up
gene23878	DEPDC1B	3.604132	5.08556	4.442178	2.003377	1.535897	1.809504	5.33E-07	-1.26348	down
gene2384	TCEA3	1.346277	1.405463	1.60616	5.339473	6.033496	6.66273	4.66E-20	2.306276	up
gene2382	E2F2	1.248666	1.237303	1.249484	0.367762	0.466255	0.506385	8.60E-06	-1.35019	down
gene23818	CCNB1	30.11682	36.06537	34.36202	16.11607	11.39707	11.76337	7.95E-13	-1.29909	down
gene23791	LOC107131556	4.9937	3.523221	3.495871	2.364139	1.603608	2.302132	0.014672	-0.88642	down
gene23743	STC2	5.955386	4.502786	5.250136	2.381698	3.066339	2.845731	0.000512	-0.70481	down
gene23731	NEURL1B	0.527021	0.312247	0.286178	0.077422	0.173043	0.131117	0.002316	-1.20237	down
gene23701	SPDL1	6.747132	9.033148	8.564821	3.347906	2.895777	2.63225	1.57E-10	-1.39238	down
gene23647	KCNJ2	0.397457	0.490997	0.51155	1.158028	0.774835	1.020519	0.000209	1.144902	up
gene23630	CDC42EP4	9.776091	8.913329	8.658967	5.180791	5.371982	3.111204	0.000533	-0.79717	down
gene23619	GPRC5C	0.329683	0.51672	0.246801	0.567586	0.666357	0.605916	0.049576	0.791914	up
gene2361	IFNLR1	0.557587	0.685904	0.680237	0.890831	1.05809	1.180703	0.014706	0.754522	up
gene2359	GRHL3	0.004976	0.012851	0	0.258995	0.340931	0.257483	3.45E-17	5.233392	up
gene23520	MGAT5B	0.084488	0.013476	0.051319	1.356674	1.746399	1.620339	7.93E-19	4.539995	up
gene23510	TMC6	11.52329	10.838	11.03388	14.45761	16.54249	16.60119	0.000883	0.585399	up
gene23507	SYNGR2	23.79286	23.46943	23.13603	31.20021	35.19734	36.87993	0.000245	0.618296	up
gene2348	CBX4	0.588324	0.687435	1.002912	0.192694	0.484286	0.929474	0.037504	-1.15567	down

gene23472	SGSH	5.20705	3.410642	4.082452	6.64272	6.257755	7.606549	0.000521	0.797597	up
gene23462	BAIAP2	1.582748	1.662819	2.000525	2.43217	2.645678	2.582443	0.007108	0.749374	up
gene23364	PITPNC1	1.557198	0.959512	0.866089	2.912138	0.802946	3.039611	0.000874	1.358106	up
gene2334	AUNIP	0.846898	1.340205	1.081106	0.41409	0.283302	0.292474	1.72E-05	-1.80119	down
gene23264	KIF18B	4.485859	3.97891	4.159474	0.889675	1.272413	1.186573	3.33E-12	-1.83044	down
gene23257	GJC1	6.85514	7.735685	7.929798	4.317965	3.690712	3.110394	1.91E-06	-0.90321	down
gene23254	CCDC43	8.658254	10.41023	9.64771	23.66994	17.6228	17.93915	5.19E-10	1.101428	up
gene23236	C19H17orf53	1.243813	0.947077	1.108894	0.528283	0.758734	0.657758	0.025815	-0.70104	down
gene23218	ETV4	0.693707	0.664303	0.74303	1.087474	1.301947	1.535987	0.002659	1.039361	up
gene2317	CEP85	5.009674	4.022428	4.430795	1.627166	2.239868	2.204659	1.04E-06	-1.04938	down
gene23160	RAB5C	35.15925	32.20675	32.41003	19.99004	17.79498	16.00159	8.67E-07	-0.82107	down
gene23066	TOP2A	24.38825	30.06283	27.94782	19.85021	15.82379	15.53156	0.000217	-0.70274	down
gene23061	CDC6	5.072141	6.128374	5.021227	1.005389	1.498417	1.004581	4.83E-16	-2.18068	down
gene23010	CWC25	3.300583	1.9715	2.184075	4.312667	3.343242	3.751846	0.032491	0.62281	up
gene22895	TOB1	12.31467	14.20739	13.23149	36.87293	27.05711	27.25262	2.82E-13	1.255247	up
gene22701	CYB5D1	0.605516	0.605467	0.918354	2.997404	2.994899	1.528711	6.86E-07	1.745069	up
gene22666	KCTD11	3.898768	3.996171	4.687915	10.87367	9.674734	9.866497	7.65E-12	1.334133	up
gene2258	SESN2	3.038275	3.414593	2.897392	11.87557	18.71733	17.07593	2.45E-37	2.728997	up
gene22511	SRR	0.573172	0.614313	0.522004	0.524503	0.637593	0.988761	0.00593	1.261099	up
gene22502	RPA1	39.09995	42.86864	42.14757	83.57085	59.8357	64.82805	4.31E-05	0.801874	up
gene2242	TMEM200B	8.210153	8.258036	8.335282	4.729037	5.224408	4.012066	0.000265	-0.76511	down
gene22407	SPAG5	11.28338	10.23328	10.75762	5.18272	6.728923	5.314891	2.69E-05	-0.84352	down
gene22355	SUZ12	7.868555	11.43837	9.202668	3.06351	2.821689	2.890858	1.51E-17	-1.59987	down
gene22352	ATAD5	4.533515	5.064618	6.17161	2.047141	1.450256	1.595139	2.07E-15	-1.57806	down
gene22319	RAD51D	3.887123	3.681743	3.702254	6.030654	5.165155	5.20514	0.007432	0.606493	up

gene22251	PPM1D	2.429804	2.35736	2.226334	4.00487	7.515292	6.33688	9.95E-07	0.941356	up
gene22216	PRR11	3.599748	3.168803	3.534944	1.144065	1.553933	1.112139	9.85E-05	-1.28532	down
gene22206	TEX14	0.963549	1.548149	0.578694	0.435757	0.078178	0.791452	0.035226	-1.10259	down
gene22202	4-Sep	2.298352	2.968336	3.310035	5.226469	2.94442	3.773196	0.006233	0.759276	up
gene22116	LOC101903649	3.601175	2.276912	2.805954	2.57346	4.982295	5.760342	0.022552	0.732106	up
gene22114	LOC101903385	1.02507	0.759655	1.309081	0.651289	0.97639	1.694333	0.007401	0.820983	up
gene22089	C18H19orf18	0.3088	0.128029	0.247293	0.499867	0.190222	0.398181	1.36E-07	2.662853	up
gene22082	LOC789960	3.177207	3.248794	3.670845	2.063727	2.508527	1.688104	0.00461	-0.6718	down
gene22076	LOC100138449	2.60828	2.602019	2.634179	2.256096	1.817986	1.922926	0.000761	-0.62147	down
gene22068	LOC509810	3.001101	2.794921	2.845863	1.603474	1.512585	1.444622	0.000467	-0.82148	down
gene22066	LOC100124497	5.19742	4.844902	6.054228	8.565691	8.887846	9.27157	1.18E-07	0.909212	up
gene2205	IQCC	3.826568	3.958651	3.950822	3.036092	2.164306	2.246923	0.018095	-0.63022	down
gene22038	LOC789715	0.212474	0.19154	0.348871	0.485708	0.495604	0.459715	0.004022	1.024278	up
gene22016	LOC101908474	2.821265	2.809106	2.623836	1.692768	1.642112	1.94198	0.015769	-0.59475	down
gene21799	LOC618456	1.328386	1.179365	1.147883	2.466833	0.971546	2.368594	0.039055	0.661443	up
gene21755	LOC508131	0.338969	0.245178	0.223901	0.280624	1.151972	1.708082	6.52E-05	2.875335	up
gene21750	LOC100299712	4.137653	4.013615	2.141249	2.560821	1.477905	0.771726	0.004072	-0.93993	down
gene21702	ZNF350	2.10525	2.750642	2.660558	1.427401	1.005328	1.600858	0.006448	-0.72973	down
gene21634	C18H19orf48	25.55631	26.66337	18.20891	9.564336	10.47883	7.56389	8.45E-08	-0.89065	down
gene21604	NUP62	17.14075	13.28525	14.94642	7.641911	10.00168	9.834203	0.000931	-0.65648	down
gene21542	BAX	20.53402	20.03202	21.67761	31.45559	39.34376	39.61611	1.40E-05	0.89812	up
gene21476	BBC3	3.027613	3.240228	1.285047	6.224677	6.310096	12.96751	3.35E-05	1.692134	up
gene21462	DACT3	2.984431	2.400186	2.297882	3.50575	3.902282	4.309877	0.008809	0.673616	up
gene21437	SIX5	4.213667	3.050898	3.449385	1.716986	2.229144	2.309632	0.008182	-0.70832	down
gene21423	FOSB	0.244497	0.250811	0.356691	0.785668	0.577643	0.486546	0.029888	0.878313	up

gene21421	CD3EAP	6.334423	5.869638	6.40701	3.942367	3.465307	3.396168	0.001216	-0.7267	down
gene21403	RELB	1.998214	1.970803	1.825292	2.70416	3.112758	3.14138	0.015727	0.664758	up
gene2133	HTR2B	0.036538	0.095387	0.171038	1.184361	0.754947	1.026902	9.61E-11	3.001429	up
gene21319	DEDD2	3.790824	2.78948	2.774754	7.493525	8.808175	7.251839	3.49E-08	1.280499	up
gene21316	gene21316	9.302124	8.486563	8.857249	4.483662	5.186421	5.785173	0.00117	-0.71496	down
gene21293	B3GNT8	1.244358	1.436022	1.911419	2.539294	2.165727	2.162011	0.045434	0.653511	up
gene21260	SPTBN4	0.178735	0.593743	0.536925	1.64211	1.252609	2.489931	2.85E-10	2.415662	up
gene21141	LOC101907883	1.307699	0.569386	1.19501	2.098321	2.522313	2.467407	4.75E-09	1.482148	up
gene21133	ZNF146	4.696413	12.84378	4.132995	5.737289	2.664633	2.432657	0.018329	-0.91095	down
gene21110	ARHGAP33	0.789226	1.105166	1.273779	0.437356	0.471526	1.022289	0.041321	-0.73854	down
gene20997	CCNE1	6.755697	7.631704	6.368132	3.913155	3.039746	3.311758	2.49E-05	-0.97927	down
gene20933	ZFP90	0.909294	0.511727	1.730636	0.670466	0.370308	0.545371	0.02898	-0.93623	down
gene20919	NFATC3	6.177516	5.990443	7.610323	10.79106	10.50351	13.12849	1.94E-07	0.814589	up
gene20900	GFOD2	2.607562	3.808633	1.149929	4.263225	3.721938	4.608348	0.011856	0.848604	up
gene20896	ACD	2.843196	2.324246	2.829384	1.369853	1.594908	1.223409	0.004845	-0.78125	down
gene20857	CES2	0.438463	0.31334	0.459719	0.780784	0.473273	0.985328	0.016267	0.942222	up
gene20815	GINS3	4.672313	3.924138	5.118072	2.04003	1.407228	1.31887	3.55E-07	-1.45154	down
gene20784	FAM192A	3.356631	3.409603	3.804873	2.445102	1.504754	2.457849	0.001951	-0.67482	down
gene20736	IRX3	5.074811	3.960995	3.649374	5.686475	7.046286	6.460257	0.005552	0.672234	up
gene20730	AKTIP	3.410337	3.810306	2.799978	7.974795	6.390376	6.120464	8.46E-07	1.090463	up
gene20679	SHCBP1	8.445054	8.118303	8.591281	4.230431	4.135117	4.280159	3.56E-06	-0.92921	down
gene20666	FANCA	3.858717	2.452713	2.794833	1.206814	2.004345	1.919654	0.043076	-0.59217	down
gene20645	CDT1	8.218529	8.151621	8.28484	2.166638	2.919286	2.823727	2.63E-11	-1.56988	down
gene20537	NUDT7	5.188737	5.432172	4.946276	7.577853	7.492859	7.666368	0.001071	0.849189	up
gene20507	MLKL	1.031759	0.991638	0.934449	1.610287	1.515218	1.680763	0.026362	0.67638	up

gene20506	RFWD3	6.729091	6.999574	9.25121	4.905268	4.208343	4.553801	4.66E-05	-0.68559	down
gene2044	DNAJB2	5.655925	5.294573	4.959953	7.146113	6.274344	7.389649	0.004001	0.619692	up
gene20407	YDJC	0.909421	1.057621	1.038266	0.893252	0.255343	0.451487	0.04139	-0.87028	down
gene20401	LOC101905136	0.464252	0.561856	0.487306	0.159547	0.292032	0.129138	5.64E-05	-1.61715	down
gene20352	ZNF280B	2.583236	2.931668	3.534387	2.338219	1.924224	1.532339	0.024354	-0.66525	down
gene20319	PLA2G3	1.308729	1.177255	1.397689	1.846557	2.38732	2.096606	0.009782	0.775296	up
gene20290	LIF	14.43033	13.91511	24.28905	8.433879	8.981374	12.29858	0.001925	-0.61178	down
gene20277	NEFH	2.6915	2.062226	1.635511	3.718853	4.182572	3.41456	0.000431	0.893638	up
gene20265	ZNRF3	1.361133	1.348639	1.078228	0.682438	0.855171	0.742288	0.02172	-0.65669	down
gene20206	UNG	18.25264	18.97811	18.70733	12.58212	10.45403	10.10526	5.94E-05	-0.69522	down
gene20171	TRIAP1	4.422145	4.240237	4.642292	6.493829	5.840077	7.319774	0.015141	0.621621	up
gene20084	VSIG10	2.636892	2.336803	10.56099	1.774853	1.820688	2.031054	0.001669	-1.93806	down
gene20078	HSPB8	0.792227	0.85688	0.973703	3.368276	3.791497	3.278803	3.43E-12	2.13028	up
gene20073	LOC101908545	0.832285	0.979567	1.090525	0.243257	0.699938	0.302658	3.28E-07	-1.42251	down
gene20020	BCL7A	1.307474	1.229514	1.52609	0.486767	0.586067	0.505121	2.26E-05	-1.29564	down
gene20007	KNTC1	6.899422	7.900168	7.678625	4.684189	4.061366	4.218163	2.93E-06	-0.74893	down
gene20	CRYZL1	3.43538	3.605387	4.323873	10.85309	4.031829	7.089279	0.001293	0.92514	up
gene19964	UBC	8.852433	8.65196	11.99211	10.75085	42.57885	10.2241	0.006736	1.220703	up
gene19879	POLE	5.115905	4.597964	6.352157	2.623242	3.251493	3.205909	0.000756	-0.67522	down
gene19794	FGF2	3.091018	3.10279	2.92556	8.307098	5.47987	6.673912	2.34E-11	1.207924	up
gene19792	NUDT6	2.708562	2.795832	2.967943	3.78002	3.309347	4.024789	0.003859	0.731155	up
gene19774	HSPA4L	4.384859	5.024111	4.872476	11.28333	7.245824	7.754432	4.21E-07	0.919305	up
gene19773	PLK4	6.677828	8.147254	8.413161	3.98722	2.988088	3.88359	2.58E-09	-1.13363	down
gene1975	MREG	3.113163	2.195781	2.66208	4.221657	3.04517	3.52891	0.0048	0.616274	up
gene19725	LOC101907835	0.665159	0.630322	1.446527	1.202622	0.748371	4.530539	0.030064	1.286454	up

gene1968	BARD1	3.455352	4.874025	2.821441	1.962147	1.800011	1.766252	7.47E-09	-1.2478	down
gene19672	SMAD1	6.909619	6.971423	7.783259	5.763667	5.315356	5.705873	0.000588	-0.64855	down
gene19659	EDNRA	0.634717	0.865824	0.988015	1.923045	1.127816	1.796725	0.002276	1.035896	up
gene19652	ARHGAP10	6.144133	5.154963	5.800723	7.532685	8.057413	8.510628	0.000831	0.621102	up
gene19626	FBXW7	5.011739	4.822325	6.174576	7.646452	7.665113	7.201419	5.50E-10	0.994242	up
gene19609	TLR2	0.678481	0.715377	0.60925	1.51953	1.324097	1.298381	0.000223	1.101202	up
gene19560	KIF14	3.705741	3.734175	3.696087	1.32202	1.053629	1.067215	1.74E-12	-1.55248	down
gene19539	CRB1	0.080788	0.038006	0.22025	0.647006	0.363547	0.438211	7.10E-07	2.282778	up
gene19537	ASPM	13.69285	14.21416	16.79391	6.281917	6.675963	5.884807	2.36E-10	-1.17628	down
gene19492	SLC30A1	21.2283	22.88023	21.64601	42.01343	32.27718	35.49686	1.15E-05	0.796177	up
gene19490	NEK2	8.117999	7.631772	7.394395	3.842724	3.540539	3.692887	2.49E-06	-0.99241	down
gene1949	KANSL1L	3.313335	4.214206	3.700948	7.864679	6.653264	5.290939	8.61E-08	1.103831	up
gene19485	DTL	15.38715	17.48677	17.81738	8.116127	6.280751	7.760823	3.35E-12	-1.13623	down
gene19476	ATF3	3.574568	3.638857	2.171454	13.82038	10.43748	10.52254	2.85E-20	1.659249	up
gene19473	NSL1	5.629386	5.770704	7.01792	4.067027	2.532743	3.13111	0.000928	-0.81166	down
gene19436	CENPF	21.8817	24.40993	25.1425	10.0131	8.29496	9.207643	1.08E-11	-1.30763	down
gene19403	C16H1orf21	4.606297	6.094914	6.481355	6.134082	7.908963	4.081845	0.019376	-0.64957	down
gene19382	RGS16	0.033329	0.118754	0.007427	0.588251	0.458884	0.57116	1.22E-07	3.350058	up
gene19298	CENPL	3.338719	2.985774	3.604699	1.594489	2.006355	1.690526	0.000831	-0.95043	down
gene19227	PLEKHN1	0.078409	0.082688	0.086626	0.679435	0.96212	0.540163	4.56E-11	3.095817	up
gene1921	CREB1	2.281726	2.210298	1.73562	2.66212	3.58365	2.628514	0.003323	0.594021	up
gene19162	PLCH2	0.10811	0.035942	0.046411	0.759008	0.974688	0.81093	1.52E-12	3.338778	up
gene19097	PHF13	5.195225	5.07498	5.153017	2.449494	3.041078	2.600943	8.67E-06	-0.97353	down
gene19074	GPR157	1.858846	1.680746	2.094555	2.600034	2.105453	4.019297	5.25E-09	1.229628	up
gene19065	SLC25A33	3.628775	3.964525	4.465174	2.714746	2.050787	2.331635	0.012424	-0.70651	down

gene19045	DFFA	4.102358	5.040738	4.290641	11.0875	9.989536	10.11791	6.78E-16	1.412609	up
gene19031	FBXO44	0.946669	0.92711	0.896324	1.418836	1.302038	1.523414	0.041034	0.663648	up
gene18966	C16H1orf112	4.174647	4.558239	3.400226	3.80924	1.810237	1.820106	0.000179	-0.79894	down
gene18965	METTL18	5.100442	5.721817	4.915745	3.053303	2.07681	2.255748	0.000592	-0.97189	down
gene18931	EXO1	6.755297	6.567494	7.163746	2.548248	2.185236	2.180723	6.15E-17	-1.4798	down
gene1891	RAPH1	3.903657	3.519177	4.183123	2.463957	4.387481	3.318139	0.007063	-0.61817	down
gene18890	LIN9	2.570784	3.183066	2.968869	1.99805	1.283963	1.395167	0.002545	-0.90109	down
gene18876	LBR	10.32302	10.65235	8.556488	5.484459	6.267417	4.304043	0.00019	-0.71105	down
gene18857	SUSD4	1.194993	1.275579	1.756202	3.409364	3.625001	3.503474	8.71E-16	2.628777	up
gene18739	PFKFB2	3.560898	2.208187	2.407193	2.447229	2.004031	2.176632	0.034205	-0.63669	down
gene18725	DYRK3	11.19108	13.79144	13.02727	36.56117	27.98597	31.98253	2.31E-18	1.461446	up
gene18718	FAM72A	1.79166	2.375745	1.95093	0.591186	0.486147	0.394292	8.37E-09	-1.98402	down
gene18710	RAB7B	2.04379	1.334844	1.653632	2.97281	2.868007	3.051259	0.000257	1.031226	up
gene18660	PRELP	2.030806	2.936646	1.654753	1.085147	1.290108	1.925663	0.029032	-0.76047	down
gene18657	BTG2	5.341457	5.026702	5.555527	12.46788	12.53649	14.05047	2.07E-13	1.3564	up
gene18645	PPFIA4	1.194535	1.255153	1.495994	2.054631	2.97458	2.355083	0.000105	0.8723	up
gene18616	LOC101902204	0.788492	0.59994	0.710424	0.30085	0.327712	0.536022	0.041521	-0.78807	down
gene18555	FAM111B	3.096948	3.797341	3.416416	2.217314	1.405746	1.611178	8.79E-05	-0.92347	down
gene1848	SGOL2	5.026375	6.456511	6.231919	3.104589	2.440017	2.488683	1.20E-08	-1.08066	down
gene1841	C2H2orf69	4.414364	4.985268	4.630572	3.428261	2.575676	2.910368	0.003664	-0.59704	down
gene18241	CRY2	1.62223	1.680526	2.090433	1.517425	3.357882	2.732549	0.009494	0.634866	up
gene1821	CCDC150	1.698404	1.181599	0.966185	2.001314	1.665603	2.131218	0.0149	0.743371	up
gene18160	DEPDC7	0.869971	1.271655	1.252957	2.006151	1.089257	1.630455	0.034203	0.69154	up
gene18131	KIF18A	3.634898	3.9095	4.137762	1.789933	1.137632	1.160539	5.59E-11	-1.58741	down
gene18121	FIBIN	0.72195	0.581824	0.60542	1.499215	0.873573	1.45509	0.00135	1.047264	up

gene18109	TSKU	8.05908	7.281544	6.664574	11.91878	17.93799	17.50223	3.87E-08	1.184975	up
gene1809	SDPR	0.117468	0.257252	0.312348	3.138247	2.843172	2.346909	8.79E-22	3.650821	up
gene18062	POLD3	6.191534	6.262413	6.668317	2.728012	2.10875	2.758031	5.61E-10	-1.27051	down
gene1801	STAT4	0.800908	1.37092	1.150591	2.233957	1.691083	1.76054	0.01286	0.830492	up
gene18008	TRPC2	1.570544	2.69967	1.686566	1.40672	1.331866	0.927784	0.023646	-0.69651	down
gene179	CRYBG3	3.496921	4.034954	3.895375	6.975654	6.46037	6.456837	1.94E-08	0.884968	up
gene17753	LOC614922	0.929677	0.733682	0.347995	2.087279	0.829034	0.995991	0.035804	1.003131	up
gene17683	RRP8	3.797262	4.024932	3.681415	3.435696	2.824933	3.027132	0.000705	-0.66357	down
gene17576	MRVI1	4.091854	3.990944	3.92488	7.378047	8.081925	8.346901	3.07E-11	1.131572	up
gene17545	RRAS2	48.26947	53.60006	54.77127	47.41804	25.83736	34.76121	0.035328	-0.71788	down
gene17503	GRAMD1B	2.197425	1.719893	2.511821	1.892025	1.691387	2.059234	0.000383	-0.94349	down
gene17447	H2AFX	51.72851	55.81488	54.64703	38.85463	32.16384	31.47616	0.000221	-0.60255	down
gene17407	FXYD2	0.843416	0.776538	1.515334	0.689438	0.633884	0.486055	0.009163	-1.06561	down
gene174	LOC782966	0.417381	0.691993	0.508864	3.686057	0.804889	0.278916	0.019651	1.568531	up
gene17387	APOA4	0.939984	0.086904	0.54979	0.814305	1.233909	1.743911	0.032906	1.315047	up
gene17286	SLC35F2	1.36872	1.847396	1.893277	1.124994	1.196792	1.047578	0.008626	-0.75111	down
gene17217	MMP1	0.025427	0.024397	0.009037	0.556343	0.416966	0.712857	9.85E-17	4.756141	up
gene17130	DSCC1	2.496021	3.032355	2.750555	1.960635	1.59646	1.7975	0.03732	-0.64003	down
gene17046	GEM	16.8797	18.7381	16.89198	13.25066	7.783058	10.91905	0.000395	-0.67351	down
gene17043	RAD54B	3.527566	3.689285	4.074257	3.181161	2.11031	2.092466	0.003225	-0.66841	down
gene17037	CCNE2	5.324821	7.24885	6.879928	2.499212	1.506301	1.82809	3.06E-11	-1.6762	down
gene17034	TP53INP1	15.47267	19.00959	17.84683	80.26874	24.0779	43.40863	2.52E-06	1.531608	up
gene16866	TPD52	6.81382	8.345074	6.185941	4.244296	4.380814	4.467628	3.73E-26	-2.59679	down
gene16787	CSPP1	4.39671	4.566808	4.774042	3.044225	2.607042	3.432958	0.000235	-0.7101	down
gene16777	MYBL1	3.194277	4.124218	3.777432	2.1484	1.615972	2.163068	1.62E-06	-1.03209	down

gene16774	RRS1	11.8628	11.13937	11.345	7.691995	6.539819	7.14741	0.001512	-0.62536	down
gene16752	TTPA	0.328274	0.358118	0.285972	0.171061	0.072205	0.096596	0.001434	-1.41337	down
gene16677	MCM4	27.54259	28.59761	28.38059	16.58585	16.80121	15.10632	1.26E-05	-0.74444	down
gene16614	MYC	12.66773	11.06227	10.52924	3.694879	9.768575	4.338787	0.010031	-0.86002	down
gene16590	TMEM71	0.514419	0.560117	0.703246	0.599582	0.315562	0.337613	0.018689	-0.8654	down
gene16360	SNAI1	1.353025	1.098623	0.971825	1.307404	1.9042	2.176182	0.031811	0.777941	up
gene16328	SULF2	0.977068	1.009212	1.22105	3.116853	3.76148	3.413587	2.19E-11	1.690107	up
gene163	LOC100335242	5.494153	6.903866	8.006793	12.79796	9.198628	9.64157	0.000352	0.653863	up
gene16299	PLTP	0.589162	0.394709	0.575189	1.123093	1.236827	0.981722	2.22E-05	1.56851	up
gene1629	ARL5A	10.42945	13.52165	13.73478	15.04494	10.03719	3.495063	0.035737	-0.73604	down
gene16231	ADA	1.120887	0.871589	1.205438	2.184965	2.077872	1.841294	0.002609	1.009187	up
gene16228	TTPAL	1.812882	3.541815	2.23669	3.562877	7.910835	5.899624	0.001285	1.023829	up
gene16187	FAM83D	9.90241	9.588	9.33498	2.941303	2.497721	2.952429	4.26E-16	-1.68911	down
gene16181	LOC524176	7.854522	8.530746	7.775029	5.052355	4.304688	3.967803	0.000118	-0.79792	down
gene16162	SRC	3.469839	2.783224	3.533472	4.623029	5.368556	4.875794	0.000382	0.755387	up
gene16151	DSN1	6.711985	7.743455	8.581489	6.494299	3.588136	4.746461	2.35E-05	-0.82857	down
gene16150	RBL1	9.339654	10.81127	11.17809	5.854105	5.04906	5.676809	4.49E-07	-0.86725	down
gene16124	LOC526745	0.050628	0.197783	0.11671	0.12381	0.494182	0.129446	0.001724	1.426475	up
gene16084	E2F1	10.35519	10.54596	9.967384	5.131383	8.101642	6.115898	0.01013	-0.59665	down
gene16081	NECAB3	0.929719	1.188092	1.282076	0.570284	0.539143	0.40581	0.006704	-0.97889	down
gene16028	TPX2	20.44284	20.3201	22.1622	10.63289	9.788802	10.54207	1.11E-07	-0.96674	down
gene16026	BCL2L1	13.34765	9.036066	10.03403	16.51124	20.37893	19.79054	6.12E-06	1.330158	up
gene15974	AURKA	12.31122	13.59324	13.04114	4.499435	4.599069	4.164094	8.06E-13	-1.48818	down
gene15853	PCMTD2	6.193295	2.855291	4.357951	12.75037	4.143491	5.074006	0.016785	0.602011	up
gene15852	GINS1	12.11634	14.46593	13.70785	7.619432	7.340011	7.216853	7.27E-06	-0.78904	down

gene15799	CDC25B	7.24559	7.270322	7.530276	1.679401	4.476326	4.430019	0.002727	-0.94004	down
gene15735	PCNA	32.79423	39.94782	38.42354	24.06062	21.35518	21.23116	3.94E-05	-0.67376	down
gene15644	GZF1	6.467406	6.290882	6.341138	9.656526	10.00237	8.802823	0.000425	0.611386	up
gene15643	NXT1	18.42512	18.14752	16.04598	11.63025	9.854913	10.13248	0.001529	-0.67619	down
gene15597	MGME1	5.218426	5.170059	5.212531	4.071516	3.404576	3.644849	0.000428	-0.71216	down
gene15587	SLC6A9	1.195675	1.254088	1.151208	0.930306	0.574688	1.295104	0.042643	-0.95603	down
gene15535	PTER	4.168767	4.516801	4.635371	11.73195	9.134838	9.765589	6.38E-10	1.267657	up
gene15500	MCM10	12.53717	14.49733	13.50465	5.982879	4.962187	4.990929	1.08E-14	-1.25839	down
gene1547	SCN2A	1.128915	1.755194	1.442646	2.93455	2.859067	2.775378	9.77E-08	1.244423	up
gene15459	SKIDA1	0.289211	0.349597	0.191667	0.123468	0.173251	0.046393	0.015927	-1.18924	down
gene15429	MASTL	2.841732	2.739163	3.125654	1.354615	1.533992	1.402843	4.37E-08	-1.18752	down
gene15278	ATP4B	1.026589	0.848352	0.899636	3.025212	3.281147	3.548974	1.48E-14	2.438171	up
gene15215	TEX30	4.9684	6.609459	5.846165	5.199382	2.75828	2.904171	0.038255	-0.66776	down
gene15214	LOC101906350	0.251842	0.201716	0.145863	0.106603	0.066232	0.09629	0.010323	-1.0935	down
gene14997	RNF219	2.720801	5.384115	4.584951	2.601376	1.397729	1.702357	0.000176	-1.07623	down
gene14966	KLF5	2.472157	2.209091	2.64404	1.545428	1.573642	1.408118	0.016124	-0.62599	down
gene14963	BORA	3.900769	5.07833	4.828233	2.770204	1.765993	2.648109	3.26E-07	-1.13271	down
gene14945	CENPJ	3.146449	4.096206	3.494045	2.635538	1.640223	2.466736	0.00368	-0.62954	down
gene14905	SPATA13	0.234138	0.157819	0.324703	0.238173	0.124473	0.127528	0.048454	-0.75087	down
gene14881	LNX2	6.406114	6.193171	5.580122	8.586456	9.32963	7.883092	0.000154	0.632704	up
gene14834	BRCA2	4.399256	4.45152	4.508687	2.82278	1.696699	1.602824	9.59E-10	-1.13762	down
gene14793	FOXO1	23.55006	7.540919	10.20725	5.487592	7.909142	6.667221	0.020628	-0.59681	down
gene14785	THSD1	9.544997	6.997797	8.616221	16.04006	20.86292	20.4032	5.00E-11	1.270109	up
gene14783	CKAP2	26.19025	28.40495	29.71387	19.2435	14.63949	15.36029	4.08E-06	-0.83556	down
gene1471	RAPGEF4	0.125407	0.105182	0.06003	0.737246	0.213565	0.25158	0.014456	1.083637	up

gene1467	CDCA7	4.708713	3.974876	4.696134	0.832258	1.061058	0.964476	1.75E-15	-2.07219	down
gene14577	SAPCD2	2.927968	2.212992	2.373144	1.129704	1.15079	0.951716	2.11E-06	-1.15049	down
gene14561	LOC104973516	5.507659	3.981231	3.606431	2.146755	1.275741	3.617707	0.038508	-0.84666	down
gene14496	NOTCH1	1.602215	0.865325	1.095457	1.247199	2.612725	2.53898	0.000683	1.012608	up
gene14424	USP20	5.154782	4.088593	4.413534	5.628936	7.390716	7.427082	0.001435	0.676934	up
gene14414	C11H9orf50	0.919897	1.308885	1.202021	2.595255	2.979448	2.886455	1.87E-05	1.341106	up
gene14333	GARNL3	0.434061	0.490365	0.360603	1.445773	1.17835	0.879487	0.000985	1.092443	up
gene14184	KLF11	4.305291	3.968422	4.595568	1.909058	1.477034	2.042433	4.46E-08	-1.19329	down
gene14182	RRM2	37.01106	43.93619	42.34196	23.90864	20.38423	18.97321	1.59E-06	-0.90058	down
gene14171	LOC100847716	1.435252	2.055789	1.397886	1.175615	0.824301	0.829285	0.030884	-0.73146	down
gene14144	GEN1	3.669989	4.428763	3.136346	2.691224	2.00956	1.884437	0.000119	-0.8175	down
gene14093	CENPO	5.973127	5.911342	5.646029	3.691526	3.287029	3.444827	0.003646	-0.66667	down
gene14056	KCNK3	2.172754	2.06622	2.192186	3.024978	3.131505	3.568209	0.001123	0.764767	up
gene13940	SPRED2	2.288485	2.820686	2.141983	0.855259	1.02964	2.118082	0.027101	-0.72112	down
gene13937	CEP68	4.154979	4.294135	4.262359	6.181824	6.006859	5.733329	0.000471	0.585333	up
gene13868	MAT2A	47.83055	49.49279	50.58911	30.46561	24.57908	31.59009	0.000106	-0.71583	down
gene13852	ST3GAL5	6.600865	6.874898	6.795701	15.3197	13.31307	16.40535	2.30E-10	1.201157	up
gene13805	CKAP2L	7.697426	9.430852	10.64743	2.542178	2.311495	2.354869	9.51E-21	-1.8869	down
gene1379	LOC101905179	0.365331	0.889308	0.723828	1.938012	0.475992	2.308595	0.01053	1.287301	up
gene13775	SH3RF3	0.435283	0.296752	0.244353	0.898527	1.296269	1.192544	7.04E-06	1.655606	up
gene13772	SOWAHC	1.0698	0.688496	0.838659	0.396511	0.342802	0.612594	0.022329	-0.88215	down
gene13760	REL	0.810597	1.214573	0.936141	1.009915	1.147815	1.055997	0.029724	0.627981	up
gene1372	NUP35	7.694508	9.519086	9.117756	6.832855	5.232181	4.186904	0.007428	-0.63579	down
gene13631	ZFP36L2	9.880431	9.455631	9.564414	6.247277	4.730704	7.364853	0.001413	-0.60211	down
gene13584	CYP1B1	6.679674	6.157804	6.197275	3.640027	2.983262	3.564843	0.000117	-0.84429	down

gene13537	XDH	4.687248	4.216468	5.00513	5.964659	6.795594	7.67194	0.000226	0.666533	up
gene13533	TGFA	1.002775	1.064108	0.900526	1.591446	1.597156	1.49245	0.006055	0.721027	up
gene1352	CALCRL	0.515574	0.597619	0.489142	1.596016	0.711265	0.827312	0.020433	0.857719	up
gene13517	CYP26B1	95.83837	49.53147	80.05858	26.4419	48.78616	54.2366	0.030606	-0.67876	down
gene13510	SFXN5	0.582308	0.565612	0.708772	0.96902	0.875526	1.040199	0.024558	0.696837	up
gene13433	IL1R1	0.815685	0.789462	0.71508	1.717724	1.846443	1.018094	6.83E-08	1.466677	up
gene13427	CREG2	0.294011	0.21036	0.392956	0.71807	0.911505	0.565984	0.000706	1.350348	up
gene13418	LOC107131213	14.35827	16.3421	15.79416	27.49139	19.87715	20.05516	0.00061	0.59249	up
gene13370	NCAPH	5.258146	6.036485	5.811346	3.373603	3.833456	3.386353	0.000238	-0.77177	down
gene13369	ITPRIPL1	1.322056	1.333805	1.319669	0.825443	0.670893	0.625266	0.004803	-0.85499	down
gene13342	BUB1	12.14179	14.19008	13.64808	9.397397	8.058145	7.791198	0.000215	-0.61518	down
gene133	LOC518080	0.100466	0.288548	0.069302	2.388228	1.998444	2.05649	1.67E-41	4.807922	up
gene13197	JDP2	5.108732	3.52648	5.950449	2.764656	2.133774	2.867919	0.037517	-0.67434	down
gene13097	ZFP36L1	33.41529	33.00423	30.89515	16.96462	21.30606	21.45771	0.000336	-0.64564	down
gene13012	SIX1	1.053839	1.180257	1.132452	0.742731	0.499563	0.624475	0.014534	-0.79303	down
gene1297	HS6ST1	6.886072	6.382926	6.938914	11.58453	14.42143	13.96139	1.54E-06	1.051357	up
gene12956	ATG14	0.916965	2.436386	1.058543	2.937269	2.06109	2.516552	0.014986	0.88762	up
gene12954	DLGAP5	11.96519	13.44745	13.15897	8.981778	7.078922	5.632018	5.65E-05	-0.77993	down
gene12951	SOCS4	9.306363	10.27281	9.822961	20.05541	12.6058	14.35166	0.000152	0.728625	up
gene12950	WDHD1	9.477404	9.581742	10.01186	5.626094	5.33373	4.690077	3.61E-09	-0.99092	down
gene12949	GCH1	6.769082	8.167515	6.949799	15.37515	11.64013	12.36187	6.54E-07	0.901305	up
gene12941	CDKN3	5.849169	3.123137	3.066801	2.93214	2.014058	1.62582	0.003793	-0.779	down
gene1290	ARHGEF4	0.140804	0.14893	0.18345	0.345858	0.231118	0.356813	0.007559	1.02671	up
gene12891	CEP152	2.779011	2.96959	3.173437	1.456352	1.265826	1.023865	1.18E-07	-1.18633	down
gene12863	AP4E1	2.312345	4.93029	3.268794	5.944983	4.287912	5.00335	0.008538	0.609119	up

gene12837	FAM214A	10.10127	10.91502	10.8485	16.5066	13.28536	15.11288	7.37E-05	0.640783	up
gene12813	RFX7	6.663483	6.83117	7.193359	11.42068	10.17481	12.56474	9.15E-07	0.794484	up
gene12756	RPS27L	93.49642	129.6103	119.0948	267.0456	116.496	126.0257	0.007047	0.661084	up
gene12740	CSNK1G1	6.546786	6.889913	6.266574	8.329497	8.732225	10.75837	6.22E-07	0.925298	up
gene12730	PIF1	0.881211	0.698544	0.40028	0.124091	0.457724	0.131507	0.009588	-1.13488	down
gene12691	MGAT2	26.47636	30.83404	28.62115	54.77621	39.89479	45.05037	4.49E-05	0.757389	up
gene12689	LRR1	3.201226	3.470279	3.360597	1.350873	1.311888	1.132271	5.99E-05	-1.2051	down
gene12687	RPS29	1056.472	965.4673	778.3104	1010.485	626.867	6157.876	0.015859	1.8206	up
gene12646	SPTBN5	0.11745	0.206671	0.46923	0.209448	0.577607	1.065274	0.011123	0.979473	up
gene12633	NUSAP1	11.18771	13.68462	12.56858	8.741418	7.215959	7.153541	0.000668	-0.6351	down
gene12632	OIP5	8.429953	5.337613	4.93031	2.486492	2.216315	2.295369	1.47E-05	-0.93879	down
gene12614	CASC5	8.101521	9.014151	9.586641	4.923893	3.769908	3.723542	9.72E-10	-1.04617	down
gene12607	KNSTRN	11.02542	13.81745	12.15885	6.346249	4.409753	3.991726	2.93E-08	-1.26639	down
gene12598	BUB1B	12.49015	14.46706	14.85077	7.420808	6.129108	5.987645	2.04E-09	-1.0364	down
gene12561	ARHGAP11A	10.63082	14.17726	14.48773	4.978887	5.091626	5.662384	2.74E-13	-1.18578	down
gene12552	RYR3	0.051252	0.283942	0.048374	0.377392	0.071215	0.100555	0.004219	1.093212	up
gene12119	RABGGTA	10.80881	10.17423	8.959729	16.84207	21.07913	20.55112	1.62E-07	1.032926	up
gene12115	LTB4R2	0.055711	0.08999	0.082679	0.937413	0.734511	1.300648	1.47E-21	3.727622	up
gene1210	CHAF1B	4.21826	3.87604	3.774628	1.990521	2.401409	2.485057	2.26E-05	-0.91181	down
gene12064	KIF23	13.68034	14.55163	14.99938	5.731125	4.922182	5.048375	3.09E-18	-1.49174	down
gene11973	SCAMP1	11.20612	14.50281	12.23087	15.15644	7.71466	11.65063	0.041931	-0.73385	down
gene11946	POLK	4.963825	6.485426	5.876128	14.38064	9.996437	10.13874	3.13E-09	1.092717	up
gene11818	QKI	14.58344	14.08008	16.3571	16.44164	17.22251	16.19005	0.039222	-0.60856	down
gene11729	RGS17	2.033175	3.175581	3.023342	5.050534	4.693148	3.23999	4.15E-05	0.826073	up
gene11727	FBXO5	5.484534	5.362522	5.588175	1.292228	0.716492	1.180739	5.26E-18	-2.19671	down

gene11704	LOC104972994	2.439215	2.140917	2.249161	0.814582	0.785572	0.75831	4.74E-09	-2.15825	down
gene11702	LOC785982	5.160564	2.52129	2.426101	8.564828	10.30382	4.269674	0.00461	1.138362	up
gene117	BTG3	21.47175	24.51916	23.12166	43.11007	26.15907	30.99867	0.00192	0.585671	up
gene11684	NUP43	6.698602	8.18184	7.379866	5.175041	3.319161	3.550986	4.89E-05	-0.85608	down
gene11591	MTFR2	5.864647	6.126481	7.298534	4.076338	2.492559	2.853128	5.65E-05	-0.95271	down
gene11584	MYB	1.776997	2.230759	1.910343	1.365472	0.573633	1.16599	0.003204	-0.93876	down
gene11448	PNRC1	14.13559	16.6581	17.43789	24.62828	22.59513	24.00374	0.000202	0.626782	up
gene11337	BEND3	4.849962	3.083266	2.952597	1.5147	1.864656	1.650282	0.000553	-0.74097	down
gene11315	SESN1	3.484909	3.95896	3.755508	11.51221	6.283287	8.758991	1.46E-09	1.233004	up
gene11290	REV3L	5.083438	5.934074	5.661547	10.0894	9.391348	9.545535	7.96E-06	0.809012	up
gene11256	DSE	13.47117	27.06749	14.33327	39.51224	30.70986	33.20463	0.000108	0.952195	up
gene11215	HSF2	4.097831	4.508574	4.803456	2.844864	2.482466	2.49141	0.000213	-0.68927	down
gene11149	TTK	5.620492	6.208184	5.854046	2.81934	2.101504	2.465242	6.08E-13	-1.37564	down
gene11145	SH3BGRL2	0.104667	0.16262	0.157457	0.719907	0.481419	0.353612	5.39E-06	1.906936	up
gene11130	HTR1B	0.59722	0.817209	0.705893	0.191864	0.19422	0.111618	1.02E-07	-2.0189	down
gene11105	MB21D1	3.41902	3.453171	3.156221	1.016152	0.80948	0.791712	1.41E-13	-1.81574	down
gene11035	PHF19	10.83704	7.509481	10.25268	5.243529	6.215185	4.589706	0.000146	-0.82077	down
gene10923	TMEM245	200.0456	12.26528	100.2788	1125.739	16.21307	743.6532	0.015851	2.656505	up
gene10921	CTNNAL1	17.7217	62.90512	18.51537	19.63242	14.82612	14.01065	0.00435	-1.2344	down
gene10889	ABCA1	0.528132	0.605735	0.597162	3.462932	2.311045	3.747349	9.56E-29	2.474165	up
gene10851	TMEM246	0.161176	0.049007	0.184089	2.155455	2.047792	1.860997	4.73E-20	3.994315	up
gene10826	SHC3	1.60405	1.16884	1.336084	0.734022	0.781865	1.206504	3.77E-05	-0.91635	down
gene10824	CKS2	33.2212	28.90262	28.54104	12.32129	11.48791	11.82668	1.23E-09	-1.28688	down
gene10766	ZNF367	19.52877	20.35602	10.94633	3.640752	2.626744	3.072825	2.23E-23	-2.12652	down
gene10758	FANCC	1.30071	1.241759	1.150592	0.269962	0.555116	0.926489	0.009957	-1.12016	down

gene10742	DAPK1	8.692604	9.314421	8.284696	11.90991	13.77645	16.59159	8.30E-06	0.790248	up
gene10721	RMI1	5.858529	7.64441	7.180809	4.851188	2.708365	3.263413	5.55E-06	-0.92427	down
gene10692	DNAI1	1.125293	0.785786	0.497928	0.993296	0.987454	1.065128	0.013498	0.816077	up
gene10690	C8H9orf24	0.383844	0.586863	0.822054	1.411963	1.643398	1.419213	9.05E-06	1.366016	up
gene10688	KIF24	2.227197	1.925749	2.0744	1.011317	1.245928	1.146081	0.001573	-0.78252	down
gene10643	LOC107132709	10.581	11.62688	10.31013	2.849532	3.316125	3.100246	6.63E-12	-1.74165	down
gene10642	CDCA2	9.539541	7.310131	12.43203	3.655642	3.376072	3.628613	1.86E-13	-1.60811	down
gene10614	LOC104968444	0.82324	1.422713	1.99873	3.2169	3.983369	3.079863	1.09E-06	1.796112	up
gene10608	LOC784541	8.325968	9.939529	8.802029	39.34801	26.61129	31.58965	2.84E-24	1.825424	up
gene10584	REEP4	14.24108	12.99061	13.31045	8.054013	7.03119	7.934846	0.000128	-0.75691	down
gene10552	LOC100297540	1.169259	1.014849	2.361618	0.878795	0.551224	0.839293	0.000849	-1.16678	down
gene10523	ANP32B	5.604724	3.247674	7.029565	2.59286	4.883117	2.80128	0.011547	-0.68021	down
gene10486	FBXO10	1.983545	2.516483	2.208298	4.084143	3.543274	4.213657	4.53E-06	0.941923	up
gene10431	ARHGEF39	10.66817	10.58905	7.1807	7.390895	5.58578	5.126327	0.001255	-0.69024	down
gene10392	CEP78	6.705254	7.509421	7.20478	4.262697	4.110909	3.716554	3.79E-05	-0.85687	down
gene10367	OSTF1	30.71313	36.94578	35.24901	79.79301	55.9703	58.67031	2.88E-08	0.973106	up
gene10342	TRPM3	3.188925	3.669801	3.868565	5.020532	5.033557	6.297084	0.000189	0.776442	up
gene10341	TRPM3	3.952155	3.682149	4.270981	6.190967	6.272389	7.294363	1.22E-05	0.834986	up
gene10339	KLF9	8.085257	9.163685	8.788054	13.47946	11.9531	13.18197	0.000124	0.607755	up
gene10335	MAMDC2	22.4162	25.13779	25.79137	40.68556	34.04822	36.15727	0.000131	0.699464	up
gene10115	CDKN2B	1.16592	1.089818	1.076402	2.643665	1.938449	1.970523	0.000465	1.029636	up
gene10052	ESCO2	5.394535	6.459653	5.913459	3.400551	2.010639	1.712506	2.53E-06	-1.16904	down
gene10051	PBK	13.67608	18.58534	17.04525	13.64382	9.033505	8.540245	0.003819	-0.60425	down
gene10041	ZNF395	1.548272	1.383538	1.51579	0.668544	0.9024	0.858364	0.00586	-0.72429	down
gene10039	FZD3	2.112205	2.670512	2.018075	3.864977	2.318114	3.049526	0.000472	0.65656	up

Bovini_newGene_9954	--	0.322309	0.268866	0.300828	0.482501	0.462275	0.577388	0.030519	0.719454	up
Bovini_newGene_99430	--	0.859546	1.112507	0.770188	1.797942	0.963777	1.369001	0.023076	0.865958	up
Bovini_newGene_98604	--	0.698198	0.273226	0.479071	0.220499	0	0.230362	0.037363	-1.64491	down
Bovini_newGene_97743	--	0.592812	0.598693	0.444868	0.374508	0.30904	0.306666	0.037577	-0.70007	down
Bovini_newGene_97183	--	1.973593	1.565553	0.549362	0.398017	0.641553	0.556064	0.005844	-1.31173	down
Bovini_newGene_95762	--	0.328643	0.330651	0.80047	0.209289	0.232128	0.45079	0.047431	1.331245	up
Bovini_newGene_9573	--	0.469289	0.540992	0.602429	0.113357	0.313844	0.372434	0.038022	-0.9317	down
Bovini_newGene_9211	--	0.495443	0.303275	0.737991	1.302233	1.741138	1.762884	2.31E-06	1.469266	up
Bovini_newGene_91688	--	0.94779	1.058175	1.10581	0.528743	0.680918	0.660639	0.001014	-0.67283	down
Bovini_newGene_91648	--	3.175895	4.561165	4.466244	1.820638	3.220342	2.550707	0.019081	-0.60135	down
Bovini_newGene_91484	--	0.120295	0.119398	0.107979	1.089768	0.565221	0.675668	3.78E-16	2.39851	up
Bovini_newGene_84864	--	0.122078	0.30998	0.238845	0.681902	0.230773	0.434799	0.02098	1.008991	up
Bovini_newGene_84418	--	312.5198	525.4864	278.0946	794.6097	713.603	505.9644	2.58E-05	1.111064	up
Bovini_newGene_83998	--	0.249719	0.146151	0.125534	0.728916	1.468868	1.171122	4.47E-12	2.764872	up
Bovini_newGene_83129	--	0.817533	1.81321	0.991638	0.490143	0.707062	0.793643	0.031283	-0.71004	down
Bovini_newGene_82842	--	0.543979	0.580139	0.630805	0.608771	0.991585	1.387129	0.029208	0.643478	up
Bovini_newGene_82812	--	0.377123	0.373982	0.393949	0.389777	0.844491	0.675592	0.03276	0.8211	up
Bovini_newGene_81043	--	0.536881	0.848773	0.999427	0.900159	1.464519	1.575069	0.006318	0.94835	up
Bovini_newGene_78448	--	0.129619	0.205984	0.195354	0.067293	0.091315	0	0.043958	-1.64108	down
Bovini_newGene_7546	--	2.202418	2.434033	2.537779	4.389712	4.092214	4.488618	0.00043	0.856645	up
Bovini_newGene_75115	--	0.35183	62.93951	0.534375	226.4763	79.31671	156.953	0.018733	2.830791	up
Bovini_newGene_74878	--	192.8717	606.6092	231.8629	2449.227	799.994	764.8221	1.56E-06	1.976828	up
Bovini_newGene_72416	--	0.348885	0.372028	0.328483	0.112638	0.228427	0.118488	0.013463	-1.10083	down
Bovini_newGene_71430	--	1.884872	1.905615	1.782386	0.698873	1.061457	1.067404	0.000124	-1.07908	down
Bovini_newGene_6840	--	0.805416	1.141572	0.88961	0.485432	0.457617	0.516807	0.000732	-0.94224	down

Bovini_newGene_64645	--	0.52099	0.403517	0.392584	0.322491	1.118065	0.942409	0.023407	0.942923	up
Bovini_newGene_64641	--	1.812917	1.57206	1.945644	2.744582	5.157178	2.902914	4.48E-05	1.108605	up
Bovini_newGene_63161	--	0.445536	0.409005	0.450944	0.152039	0.171853	0.145122	0.000762	-1.40013	down
Bovini_newGene_62073	--	1.389786	1.58801	2.374494	0.276726	0	0.909823	0.036955	-2.28196	down
Bovini_newGene_62050	--	1.295581	0.762932	0.737608	1.763032	0.766532	1.902731	0.025732	1.15438	up
Bovini_newGene_61256	--	1.296424	1.300148	1.259804	0.715697	0.814136	0.640851	0.038118	-0.66655	down
Bovini_newGene_60650	--	0.735125	0.776903	0.696923	0.341931	0.295002	0.375209	0.001	-1.06542	down
Bovini_newGene_60340	--	0.49414	0.581886	0.603051	1.225789	0.656972	1.277147	0.001019	1.154658	up
Bovini_newGene_60265	--	0.863103	2.281857	1.272376	1.978175	2.932525	2.944823	0.00732	0.982327	up
Bovini_newGene_5602	--	0.141107	0.180209	0.127296	0.3899	0.421893	0.567883	7.79E-05	1.661606	up
Bovini_newGene_50138	--	4.274589	5.291449	4.902243	4.089437	2.522385	3.646156	0.011358	-0.59654	down
Bovini_newGene_48446	--	0.907986	0.762267	0.983489	0.391712	0.403935	0.553361	0.005899	-0.84095	down
Bovini_newGene_48038	--	2.009593	1.270911	8.231306	40.92234	165.9359	152.3852	5.79E-11	3.420511	up
Bovini_newGene_47780	--	0.925682	0.978864	0.937908	1.677028	1.835642	2.145786	6.57E-05	1.08548	up
Bovini_newGene_45001	--	0.465605	0.653404	0.36847	0.222133	0.18679	0.267095	0.008283	-1.06361	down
Bovini_newGene_42298	--	2.58226	3.944639	4.029566	1.731912	1.844811	2.750544	0.004839	-0.70833	down
Bovini_newGene_39791	--	0.239497	0.321444	0.297862	0.38483	0.625615	0.567312	0.013301	0.947195	up
Bovini_newGene_38904	--	0.218781	0.21918	0.311266	0.304077	0.497876	0.456251	0.011338	1.017716	up
Bovini_newGene_35872	--	2.788676	2.525671	2.736465	3.222787	4.503308	3.802734	0.017292	0.606506	up
Bovini_newGene_35871	--	1.110283	1.006074	1.18184	1.930284	1.401029	1.815156	0.019177	0.791487	up
Bovini_newGene_35868	--	3.109077	3.762593	3.445198	4.488646	5.552333	5.88106	0.000939	0.75313	up
Bovini_newGene_35757	--	12.82323	11.26127	10.14601	10.95594	20.72525	24.3	0.000916	0.979658	up
Bovini_newGene_35340	--	0.083595	0.413421	0.063112	0.844189	0.016545	0.63504	0.000737	3.696047	up
Bovini_newGene_34293	--	0.318546	0.098584	0.284999	1.172441	1.090459	1.231275	1.05E-07	2.570288	up
Bovini_newGene_33037	--	0.796465	0.601888	0.715194	0.843838	1.095116	1.234652	0.032154	0.608779	up

Bovini_newGene_31853	--	0.759374	0.765905	0.500289	0.271334	0.383866	0.566909	0.033199	-0.86854	down
Bovini_newGene_30003	--	0.770918	0.66808	0.726556	1.398513	1.553792	1.872913	0.001745	0.980858	up
Bovini_newGene_29882	--	1.412619	1.55416	1.096113	0.700484	1.006965	0.745088	0.049057	-0.72072	down
Bovini_newGene_28174	--	2.288728	2.058431	1.928388	1.094894	0.788119	0.897698	6.03E-07	-1.01866	down
Bovini_newGene_23607	--	1.893006	1.912849	2.087586	1.204932	1.20755	1.069864	3.43E-06	-1.0016	down
Bovini_newGene_23096	--	0.339062	0.235208	0.280261	0.397891	0.821521	0.54501	0.023961	1.029798	up
Bovini_newGene_18358	--	0.194123	0.08656	0.170578	0.42363	0.893362	0.852878	2.76E-08	2.335835	up
Bovini_newGene_18347	--	0.714724	0.87203	0.665771	1.826345	3.058146	3.684561	1.45E-16	2.275206	up
Bovini_newGene_1822	--	0.460276	0.323659	0.237718	0.122854	0.139873	0.175014	0.001382	-1.16157	down
Bovini_newGene_1819	--	0.480557	0.433508	0.61555	0.154538	0.323714	0.35014	0.033843	-0.63274	down
Bovini_newGene_16138	--	0.587416	0.742312	0.618802	0.222924	0.407342	0.456135	0.047652	-0.73616	down
Bovini_newGene_15413	--	0.067029	0.072251	0.084223	0.236295	0.21034	0.188369	7.10E-05	1.545026	up
Bovini_newGene_152486	--	4.090312	5.275709	5.239038	18.90722	17.44617	21.20197	1.20E-21	1.998726	up
Bovini_newGene_152484	--	7.079592	8.539748	8.974042	28.13037	32.0515	31.37261	4.52E-27	1.939465	up
Bovini_newGene_150889	--	0.380011	0.391598	0.440344	0.490043	0.798428	0.782937	0.001112	0.847695	up
Bovini_newGene_149450	--	0	0	2.212979	1148.687	0	228.385	0.000194	9.288903	up
Bovini_newGene_149326	--	0.273373	0.392169	0.356995	0.634897	0.650415	0.753926	0.001727	1.055941	up
Bovini_newGene_147732	--	1.017531	0.831518	1.438478	0.234914	0.437527	1.026782	0.014531	-0.83022	down
Bovini_newGene_147322	--	0.199706	0.299856	0.790525	0.219218	0.229021	0.109116	0.022961	-1.212	down
Bovini_newGene_146000	--	1.200868	1.214559	0.86469	0.69924	0.769785	0.660465	0.010627	-0.91833	down
Bovini_newGene_14484	--	0.229586	0.222309	0.257417	0.068036	0.113111	0.067794	0.000407	-1.42353	down
Bovini_newGene_143200	--	0.245666	0.363256	0.205929	0.691741	1.256099	1.059328	2.55E-07	1.957552	up
Bovini_newGene_139382	--	1.202327	0.329261	1.173254	0.065332	0.757644	0.345691	0.006012	-1.49528	down
Bovini_newGene_137047	--	0.57074	0.398997	0.51714	0.060195	0.214274	0.185959	0.001476	-1.59125	down
Bovini_newGene_132655	--	0.96608	0.942891	1.238574	0.460212	0.449607	0.492622	0.002398	-1.10191	down

Bovini_newGene_132652	--	1.118404	1.337684	1.569887	0.802641	0.490161	0.378085	0.002328	-1.22754	down
Bovini_newGene_131258	--	0.562596	0.52664	0.744208	1.041042	1.023754	1.82598	0.000545	1.070057	up
Bovini_newGene_130740	--	0.883124	1.057073	1.092858	0.406031	0.317736	0.343297	1.37E-05	-1.43029	down
Bovini_newGene_128623	--	132.2339	178.68	153.7882	291.8916	126.9474	323.4425	0.008742	0.724347	up
Bovini_newGene_126969	--	0.567333	0.731298	0.68748	1.225236	0.688414	1.018954	0.001442	0.890631	up
Bovini_newGene_125863	--	0.193429	0.17852	0.209066	0.929327	1.626397	1.383706	1.99E-21	2.50959	up
Bovini_newGene_124296	--	0.54061	0.641261	0.715181	1.007145	1.271357	1.210342	0.005969	0.809827	up
Bovini_newGene_124203	--	0.267943	0.184562	0.193532	0.675057	0.726516	0.821918	4.14E-11	1.841958	up
Bovini_newGene_123883	--	1.266481	0	1.025043	0.101102	0.08162	0.091331	0.01163	-3.01011	down
Bovini_newGene_121262	--	0.231562	0.276842	0.290238	0.039817	0	0.163506	0.024137	-1.93012	down
Bovini_newGene_120263	--	0.750588	0.552849	0.674594	0.267732	0.496031	0.462124	0.046638	-0.61466	down
Bovini_newGene_11991	--	0.765108	0.863258	0.83291	1.88586	1.059407	1.436509	0.00873	0.912871	up
Bovini_newGene_119692	--	1.173563	1.04944	1.172465	0.500922	0.764201	0.786227	0.020114	-0.65386	down
Bovini_newGene_119691	--	2.153658	2.229517	1.478416	0.451302	1.0394	1.292709	8.46E-16	-2.629	down
Bovini_newGene_11745	--	0	0.129942	0.347985	0.306456	0.689244	0.903908	0.017292	2.069359	up
Bovini_newGene_115573	--	0.164877	0.175749	0.197407	0.262381	0.376684	0.426562	0.003626	1.053851	up
Bovini_newGene_114435	--	1.198294	1.078487	1.032519	3.028492	1.86716	2.09292	0.000228	1.126165	up
Bovini_newGene_114424	--	0.116923	0.117055	0.145742	0.321481	0.188777	0.416504	0.013714	1.018252	up
Bovini_newGene_109865	--	0.001616	0.030442	0.087688	0.856423	0.755419	0.531494	3.99E-11	3.739408	up
Bovini_newGene_109803	--	1.671667	1.705842	1.867346	0.817895	1.094661	1.256433	0.004929	-0.65763	down
Bovini_newGene_109162	--	0.394799	0.226294	0.307836	0.820213	0.547936	0.631158	0.006281	1.20423	up
Bovini_newGene_108246	--	0.571069	0.997012	0.824839	1.789246	2.107178	1.466922	4.80E-05	1.272139	up
Bovini_newGene_106193	--	3.528673	1.458231	4.119955	6.096513	3.56222	4.082863	0.049895	0.638009	up
Bovini_newGene_101865	--	0.408393	0.427957	0.48058	0.691661	0.647679	0.621912	0.000706	1.453498	up
Bovini_newGene_101466	--	0.698043	0.447384	0.724004	0.143779	0.271081	0.719104	0.003219	-1.04676	down

Bovini_newGene_101391	--	1.005327	0.561327	0.818705	0.707533	0.073456	0.269342	0.041512	-1.14901	down
Bovini_newGene_101205	--	172.5474	489.1201	119.742	1925.679	691.6748	778.3849	4.24E-06	2.145373	up
