# Supplementary material for: DNA Double-Strand Break-Related Competitive Endogenous RNA Network of Noncoding RNA in Bovine Cumulus Cells
Source: Genes (Basel). 2023 Jan 22;14(2):290. doi: 10.3390/genes14020290 (PMC9956238; doi:10.3390/genes14020290)
Supplement: Supplementary file 1 [file genes-14-00290-s001.zip › Table S4.pdf]

**Supplementary Table S4. Differentially expressed lncRNAs**

| #ID | NC_FPKM | NC_FPKM | NC_FPKM | BLM_FPKM | BLM_FPKM | BLM_FPKM | Pvalue | log2FC | regulated |
|---|---|---|---|---|---|---|---|---|---|
| MSTRG.102941.1 | 0 | 0.015415 | 0 | 0.128232 | 0.371093 | 0.131703 | 0.002253 | 4.228254 | up |
| MSTRG.112545.1 | 0 | 0 | 0 | 0.026111 | 0.601892 | 0.136393 | 0.046921 | 5.583367 | up |
| MSTRG.117210.1 | 0 | 0 | 0 | 0.283293 | 0.665494 | 0.027811 | 0.02704 | 5.873336 | up |
| MSTRG.124291.1 | 0 | 0.030702 | 0 | 0.191248 | 0.260397 | 0.567761 | 0.040641 | 4.048418 | up |
| MSTRG.128317.1 | 0.040256 | 0.074331 | 0.065182 | 0.259485 | 0.188719 | 0.378828 | 0.036486 | 2.008578 | up |
| MSTRG.128785.1 | 0.003897 | 0.002395 | 0 | 0.044035 | 0.160239 | 0.005124 | 0.001719 | 4.517381 | up |
| MSTRG.13419.1 | 0 | 0 | 0 | 0.36713 | 0.003927 | 0.110672 | 0.049878 | 5.532244 | up |
| MSTRG.146346.1 | 0 | 0.038158 | 0.062496 | 0.164811 | 0.27213 | 0.156679 | 0.015903 | 2.331837 | up |
| MSTRG.147601.1 | 0.005686 | 0.002139 | 0.0312 | 0.155625 | 0.033512 | 0.211851 | 0.046002 | 2.586912 | up |
| MSTRG.152485.1 | 0 | 0 | 0 | 0.200877 | 0.075148 | 0.20444 | 0.048126 | 5.137357 | up |
| MSTRG.155388.1 | 0 | 0 | 0 | 0.310575 | 0.082713 | 0.043198 | 0.043862 | 5.422232 | up |
| MSTRG.155681.1 | 0.084365 | 0 | 0.020205 | 0.234483 | 0.250504 | 0.088838 | 0.013027 | 2.587821 | up |
| MSTRG.155681.4 | 0.002259 | 0.002964 | 0.004059 | 0.298349 | 0.388151 | 0.002623 | 3.47E-05 | 5.562449 | up |
| MSTRG.155787.1 | 0 | 0 | 0 | 0.576198 | 0.307937 | 0.064832 | 0.012845 | 6.059244 | up |
| MSTRG.177599.1 | 0.25334 | 0.232668 | 0.245595 | 0.676266 | 0.835413 | 0.675791 | 0.00892 | 1.644736 | up |
| MSTRG.185353.3 | 0.456342 | 0.551026 | 0.32722 | 2.164983 | 0.535922 | 1.199747 | 0.024108 | 1.692898 | up |
| MSTRG.187126.4 | 0.03828 | 0.018366 | 0.050399 | 0.153283 | 0.194136 | 0.264293 | 0.000662 | 2.351336 | up |
| MSTRG.197583.1 | 0.024172 | 0.026213 | 0.035687 | 0.210918 | 0.17384 | 0.063569 | 0.005411 | 2.396307 | up |
| MSTRG.202064.1 | 0 | 0 | 0 | 0.021146 | 0.022602 | 0.192826 | 0.013399 | 6.025181 | up |
| MSTRG.216087.1 | 0 | 0 | 0 | 0.090727 | 0.128868 | 0.123116 | 0.004875 | 6.155142 | up |
| MSTRG.22243.1 | 0.013863 | 0.010414 | 0.028726 | 0.039165 | 0.149392 | 0.11402 | 0.012132 | 2.365678 | up |
| MSTRG.225326.1 | 0.030444 | 0.063569 | 0.067239 | 0.027392 | 0.219759 | 0.32053 | 0.033498 | 1.734471 | up |
| MSTRG.233543.1 | 0.084621 | 0.218583 | 0.121014 | 0.567919 | 0.698482 | 0.148294 | 0.021032 | 1.829678 | up |

| | | | | | | | | | |
|---|---|---|---|---|---|---|---|---|---|
| MSTRG.245613.1 | 0 | 0 | 0 | 0.21519 | 0.230007 | 0.051102 | 0.018494 | 5.896555 | up |
| MSTRG.24637.1 | 0 | 0 | 0 | 0.062858 | 0.044791 | 0.085298 | 0.011981 | 5.910862 | up |
| MSTRG.252009.1 | 0 | 0 | 0 | 0.161591 | 0.172717 | 0.246686 | 0.023821 | 5.605835 | up |
| MSTRG.257787.1 | 0.07816 | 0.019036 | 0.248259 | 0.029972 | 3.399158 | 0.215148 | 0.010079 | 3.339507 | up |
| MSTRG.264056.1 | 0 | 0 | 0.027949 | 0.314827 | 0.107097 | 0.233617 | 0.002899 | 3.806405 | up |
| MSTRG.266341.1 | 0.062171 | 0.035578 | 0.054435 | 0.131198 | 0.27996 | 0.216221 | 0.002147 | 2.060967 | up |
| MSTRG.65406.1 | 0 | 0 | 0 | 0.252346 | 0.016398 | 0.019862 | 0.034145 | 5.897755 | up |
| MSTRG.65861.1 | 0 | 0 | 0.007378 | 0.253334 | 0.360235 | 0.160032 | 0.040457 | 4.101015 | up |
| MSTRG.67316.2 | 0.338352 | 0.251815 | 0.219463 | 0.710965 | 0.305246 | 3.238857 | 0.005296 | 2.396228 | up |
| MSTRG.69684.1 | 0.102196 | 0.103556 | 0.07354 | 0.170579 | 0.310409 | 0.294875 | 0.018199 | 1.517669 | up |
| MSTRG.77140.1 | 0 | 0 | 0 | 0.08152 | 0.660574 | 0.041333 | 0.044396 | 5.686679 | up |
| MSTRG.81157.2 | 0.19282 | 0.111924 | 0.053662 | 0.759811 | 0.187488 | 0.194674 | 0.028212 | 1.832348 | up |
| MSTRG.87347.1 | 0 | 0 | 0 | 0.114837 | 0.204573 | 0.034283 | 0.020637 | 5.877652 | up |
| MSTRG.93118.1 | 0.003428 | 0.000122 | 0.002502 | 0.468 | 0.064576 | 0.398449 | 3.35E-10 | 7.058812 | up |
| MSTRG.95875.1 | 0 | 0 | 0 | 0.203364 | 0.002898 | 0.275273 | 0.037547 | 5.616748 | up |
| MSTRG.112314.1 | 1.042455 | 0.026203 | 0.057003 | 0.05812 | 0.072498 | 0.077249 | 0.030064 | -2.32548 | down |
| MSTRG.114071.1 | 2.55772 | 1.424795 | 0.3231 | 0.250189 | 0.200808 | 0.615393 | 0.018371 | -1.9559 | down |
| MSTRG.122845.1 | 0.352081 | 0.070288 | 0.265424 | 0 | 0.075303 | 0 | 0.019463 | -3.13776 | down |
| MSTRG.124142.1 | 0.421874 | 0.10715 | 0.034035 | 0 | 0 | 0 | 0.030667 | -5.78538 | down |
| MSTRG.126418.1 | 0.143657 | 0.19128 | 0.241447 | 0 | 0.068338 | 0.030032 | 0.038502 | -2.25955 | down |
| MSTRG.135539.1 | 0.124758 | 0.134813 | 0.074927 | 0.000478 | 0 | 0.037001 | 0.036402 | -2.64458 | down |
| MSTRG.141129.1 | 0.301757 | 0.059449 | 0.155786 | 0 | 0 | 0 | 0.028434 | -5.59398 | down |
| MSTRG.146080.1 | 0.180469 | 0.732545 | 0.078633 | 0 | 0 | 0 | 0.025105 | -5.8489 | down |
| MSTRG.147952.1 | 0.078087 | 0.372146 | 0.206319 | 0 | 0 | 0.08426 | 0.043125 | -2.97762 | down |
| MSTRG.148522.1 | 0.195906 | 0.171849 | 0.225226 | 0 | 0 | 0.046717 | 0.046881 | -3.0825 | down |

| | | | | | | | | | |
|---|---|---|---|---|---|---|---|---|---|
| MSTRG.16158.1 | 0.936794 | 0.158137 | 0.061356 | 0.074141 | 0.091769 | 0.047924 | 0.013902 | -2.27371 | down |
| MSTRG.177123.1 | 0.085944 | 0.275354 | 0.172368 | 0.006043 | 0.018236 | 0 | 0.015679 | -3.60239 | down |
| MSTRG.193401.1 | 0.160976 | 0.35318 | 0.049685 | 0 | 0.023052 | 0 | 0.002903 | -4.22053 | down |
| MSTRG.197668.1 | 0.082993 | 0.195868 | 0.261539 | 0 | 0 | 0 | 0.039296 | -5.39123 | down |
| MSTRG.214559.1 | 0.097363 | 0.166051 | 0.24716 | 0.008182 | 0.020752 | 0.00945 | 0.001114 | -3.28154 | down |
| MSTRG.215381.1 | 0.042434 | 0.065319 | 0.587005 | 0.015922 | 0 | 0.035484 | 0.019972 | -3.25594 | down |
| MSTRG.21667.1 | 0.334703 | 0.293424 | 0.320403 | 0 | 0.068023 | 0.060522 | 0.002946 | -2.78611 | down |
| MSTRG.222809.1 | 0.046264 | 0.213053 | 0.062206 | 0 | 0 | 0 | 0.022991 | -5.74624 | down |
| MSTRG.229041.1 | 0.49096 | 0.280427 | 0.304768 | 0 | 0 | 0.027077 | 0.045753 | -3.90723 | down |
| MSTRG.231328.1 | 0.077354 | 0.088013 | 0.078006 | 0 | 0 | 0 | 0.026195 | -5.54437 | down |
| MSTRG.239680.1 | 0.140655 | 0.242625 | 0.114138 | 0 | 0 | 0 | 0.001733 | -6.41343 | down |
| MSTRG.242691.1 | 0.250038 | 0.153007 | 0.066938 | 0 | 0 | 0.042098 | 0.041375 | -2.99676 | down |
| MSTRG.25896.1 | 0.135588 | 0.104166 | 0.149267 | 0.020292 | 0 | 0.020652 | 0.017121 | -2.59951 | down |
| MSTRG.262965.1 | 0 | 0.253658 | 0.253221 | 0 | 0 | 0.007481 | 0.039238 | -4.35301 | down |
| MSTRG.268366.1 | 0.045631 | 0.066728 | 0.183449 | 0 | 0 | 0 | 0.029387 | -5.60838 | down |
| MSTRG.31915.1 | 1.168788 | 1.319658 | 1.035635 | 0.526581 | 0.410582 | 0.218279 | 0.046157 | -1.38948 | down |
| MSTRG.49414.1 | 0.079456 | 0.088832 | 0.162725 | 0.027632 | 0.041348 | 0.016873 | 0.03253 | -1.65404 | down |
| MSTRG.59150.1 | 0.091106 | 0.609457 | 0.126582 | 0 | 0 | 0 | 0.027869 | -5.73931 | down |
| MSTRG.59961.1 | 0.314307 | 0.157467 | 0.114479 | 0.031519 | 0.054952 | 0.032079 | 0.003687 | -2.10285 | down |
| MSTRG.62074.1 | 0.121489 | 0.104443 | 0.234186 | 0 | 0.018002 | 0 | 0.026942 | -3.53389 | down |
| MSTRG.63995.1 | 0.038509 | 0.207175 | 0.206597 | 0 | 0.03382 | 0 | 0.039982 | -3.17963 | down |
| MSTRG.68239.1 | 0.108327 | 0.083374 | 0.05462 | 0 | 0 | 0 | 0.040764 | -5.22963 | down |
| MSTRG.7914.1 | 0.204503 | 0.230042 | 0.105758 | 0 | 0.054801 | 0 | 0.00852 | -3.27456 | down |
| MSTRG.80408.1 | 0.308216 | 0.05225 | 0.15444 | 0 | 0 | 0 | 0.002288 | -6.43049 | down |
| MSTRG.822.1 | 0.593744 | 0.364149 | 0.580044 | 0 | 0.281086 | 0.118287 | 0.030634 | -1.89473 | down |

| | | | | | | | | | |
|---|---|---|---|---|---|---|---|---|---|
| MSTRG.86775.1 | 0.413401 | 0.060147 | 0.026269 | 0 | 0 | 0 | 0.044845 | -5.53881 | down |
| MSTRG.971.1 | 0.131705 | 0.247829 | 0.214867 | 0 | 0 | 0.055068 | 0.03319 | -2.99066 | down |
