# Supplementary material for: DNA Double-Strand Break-Related Competitive Endogenous RNA Network of Noncoding RNA in Bovine Cumulus Cells
Source: Genes (Basel). 2023 Jan 22;14(2):290. doi: 10.3390/genes14020290 (PMC9956238; doi:10.3390/genes14020290)
Supplement: Supplementary file 1 [file genes-14-00290-s001.zip › Table S5.pdf]

Supplementary Table S5. Differentially expressed circRNAs

#ID	NC_FPKM	NC_FPKM	NC_FPKM	BLM_FPKM	BLM_FPKM	BLM_FPKM	PValue	log2FC	regulated
AC_000158.1:53820984|53827241	0	3111.141	1369.37	1055.726	0	0	0.471155	-1.64003	down
AC_000158.1:69644514|69650504	857.5703	653.6287	0	199.6206	0	597.4752	0.638105	-0.90399	down
AC_000158.1:91966700|92022294	0	23.47867	0	17.92618	78.23539	0	0.404146	1.859904	up
AC_000158.1:107948998|107975652	157.7031	146.9101	0	179.4676	265.1635	149.21	0.21894	1.006838	up
AC_000158.1:151034206|151054964	5179.137	5263.293	0	6831.572	6028.788	6013.901	0.186955	0.939276	up
AC_000159.1:20992753|21047650	1242.146	1188.838	1230.333	0	0	1151.907	0.006649	-1.85212	down
AC_000159.1:20992753|21047706	0	29.69066	64.68838	25.38938	89.04145	36.18657	0.811455	0.622184	up
AC_000159.1:28012365|28017086	6085.524	4251.78	0	2597.018	0	0	0.646258	-1.46731	down
AC_000159.1:35276763|35300122	0	6011.234	0	8392.469	10015.29	0	0.282695	1.651303	up
AC_000159.1:92120815|92126362	0	2228.99	0	3812.153	0	3803.327	0.21656	1.845226	up
AC_000160.1:451877|464312	8779.398	13494.61	6074.65	4495.966	0	0	0.092511	-2.15547	down
AC_000160.1:42522336|42531072	14110.69	22675.09	11716.18	12645.75	0	0	0.281221	-1.4373	down
AC_000160.1:100359682|100360151	3975.012	0	0	4664.963	0	3384.845	0.309968	1.389571	up
AC_000161.1:57251461|57254120	351.1759	0	0	149.8655	408.7873	0	0.789711	0.807406	up
AC_000161.1:75520956|75522308	0	8701.977	5170.742	1594.569	4349.497	0	0.391346	-1.25773	down
AC_000161.1:87352684|87371198	8295.344	5039.755	7237.043	4617.478	5877.698	0	0.361247	-0.7799	down
AC_000162.1:12007564|12043849	154.4609	0	249.3744	82.39589	299.6677	241.135	0.565472	0.626637	up
AC_000162.1:65936896|65952713	10689.84	0	0	4561.928	0	9802.941	0.544768	0.608786	up
AC_000162.1:101603226|101603456	16175.38	0	0	19845.83	14121.74	0	0.300891	1.442102	up
AC_000162.1:104231667|104232045	0	0	5854.554	2166.534	13789.17	0	0.67876	1.430339	up
AC_000162.1:108308462|108309286	0	5142.077	0	4590.426	0	10123.76	0.294328	1.450464	up
AC_000163.1:46430752|46431533	15699.63	10054.81	9052.42	5583.225	7614.665	2228.12	0.375848	-1.04746	down
AC_000164.1:4209868|4211760	0	0	4039.643	5813.534	0	4971.492	0.3976	1.717602	up

AC_000164.1:31516094|31547896	4569.104	3901.702	4566.552	2816.495	0	4755.34	0.658272	-0.65213	down
AC_000164.1:39760828|39781786	9203.231	0	4775.932	4418.449	0	0	0.730652	-1.04528	down
AC_000164.1:39760828|39789878	0	0	0	870.398	1978.483	4052.453	0.00579	Inf	up
AC_000164.1:55303870|55306582	7297.874	0	10099.11	0	5663.407	0	0.308175	-1.74031	down
AC_000164.1:63854849|63858288	9613.666	16232.25	11086.5	0	0	16372.67	0.173051	-1.38424	down
AC_000165.1:40614837|40622735	5251.844	2935.45	3149.346	1045.913	0	4472.106	0.36673	-1.03751	down
AC_000165.1:40621610|40622735	4562.792	2608.275	3731.109	0	3862.786	0	0.096093	-1.66959	down
AC_000165.1:40834490|40838780	6843.428	0	8286.446	3650.571	0	0	0.546402	-1.44371	down
AC_000165.1:73730636|73731745	0	0	29847.04	16504.3	32898.26	32932.17	0.127756	1.506483	up
AC_000165.1:78506771|78524362	710.5435	0	2130.44	0	0	1815.149	0.678278	-0.76469	down
AC_000166.1:34348787|34358839	5730.846	2001.989	991.3233	2139.951	0	0	0.557429	-1.46083	down
AC_000166.1:45777122|45786741	0	7549.916	0	4611.543	0	12268.98	0.473703	1.064182	up
AC_000166.1:81347964|81348317	10555.12	4609.098	0	0	4607.518	0	0.257912	-1.89346	down
AC_000166.1:88955812|88961729	3156.904	2095.356	0	1044.095	0	2217.766	0.796529	-0.60537	down
AC_000167.1:10556787|10560879	0	8450.625	5150.142	5558.757	8664.336	7130.426	0.640266	0.613443	up
AC_000167.1:29803745|29810994	8158.322	18524.95	0	3916.791	0	0	0.312155	-2.31098	down
AC_000168.1:10136061|10136478	0	0	3450.228	5674.623	11608.98	2830.743	0.186359	2.650356	up
AC_000169.1:81157119|81162748	2073.996	0	2181.263	0	2221.102	0	0.335338	-1.05627	down
AC_000170.1:34860214|34944186	44.49658	19.4303	0	14.2418	97.1182	66.30787	0.196056	1.377309	up
AC_000170.1:51740448|51746695	5486.801	0	11863.85	2926.889	0	0	0.317767	-1.96589	down
AC_000171.1:38926837|38986125	0	27.51979	0	23.533	55.0207	67.08147	0.157941	2.212745	up
AC_000171.1:63954121|64038908	424.1629	153.9483	0	235.0818	391.151	333.0433	0.156492	0.783235	up
AC_000172.1:64524924|64529858	7771.447	0	4301.782	4643.088	0	0	0.850654	-0.7629	down
AC_000172.1:64704077|64705175	13174.69	0	12056.44	6891.905	0	0	0.519923	-1.26172	down
AC_000173.1:42732861|42735244	0	2510.186	7457.802	6899.578	14637.73	15296.9	0.232144	1.863949	up

AC_000175.1:46155528|46202870	157.8485	0	218.4375	84.20301	229.68	420.0403	0.296098	0.952581	up
AC_000175.1:48819153|48819571	0	13456.67	0	14384	11210.04	0	0.510092	1.039457	up
AC_000176.1:39742140|39768649	0	0	365.7164	218.0428	574.2451	465.0811	0.059254	1.801181	up
AC_000176.1:46774559|46775930	5787.236	5708.294	7301.978	3196.112	0	7536.955	0.448101	-0.73605	down
AC_000177.1:6690071|6692376	0	10877.47	0	6644.038	0	10846.89	0.627737	0.670529	up
AC_000178.1:8020127|8020672	26518.28	27492.51	30186.34	17887.8	0	31322.22	0.462158	-0.65651	down
AC_000178.1:42937592|42966175	0	1473.051	2660.2	1799.501	0	0	0.755074	-0.68655	down
AC_000178.1:45366129|45368903	4753.832	9964.096	9045.51	5578.963	0	0	0.32745	-1.58239	down
AC_000178.1:58507060|58508811	5156.858	4117.655	7391.144	2986.673	3001.427	3450.247	0.404881	-0.72411	down
AC_000181.1:934078|947669	1786.884	0	832.1806	703.9005	0	0	0.590497	-1.27819	down
AC_000181.1:41588500|41590938	0	0	8415.922	3633.458	18406.07	4142.91	0.392061	1.625367	up
AC_000182.1:2062843|2071436	9504.208	5217.392	0	6373.641	10273.16	6936.965	0.324193	0.730181	up
AC_000182.1:10461217|10480061	6932.304	9081.377	18586.85	0	0	10330.37	0.050021	-1.92038	down
AC_000182.1:10832424|10838285	0	3521.484	10462.38	2867.93	0	0	0.409618	-1.75093	down
AC_000182.1:38287754|38329596	0	0	84.95771	61.92613	155.922	133.0705	0.111337	2.068562	up
AC_000187.1:74265660|74273915	0	0	0	1871.56	3190.652	2800.84	0.01447	Inf	up
AC_000187.1:79761001|79786582	9717.003	0	7171.626	6634.823	0	0	0.823069	-0.73512	down
AC_000187.1:97207129|97209131	0	0	4683.644	1805.445	5909.642	2161.518	0.658804	1.102523	up
