# Supplementary material for: DNA Double-Strand Break-Related Competitive Endogenous RNA Network of Noncoding RNA in Bovine Cumulus Cells
Source: Genes (Basel). 2023 Jan 22;14(2):290. doi: 10.3390/genes14020290 (PMC9956238; doi:10.3390/genes14020290)
Supplement: Supplementary file 1 [file genes-14-00290-s001.zip › Table S6.pdf]

Supplementary Table S6. Differentially expressed miRNAs

#ID	NC_ FPKM	NC_ FPKM	NC_ FPKM	BLM_ FPKM	BLM_ FPKM	BLM_ FPKM	PValue	Log2FC	regulated
unconservative_AC_000169.1_288145	0	0	0	0	0.45	1.38	0.000744	6.207248	up
unconservative_AC_000183.1_527362	0	0	0	1	0.18	0.13	0.003331	5.922996	up
unconservative_AC_000175.1_385872	0	0	0	0.55	0.27	0.5	0.000477	5.828651	up
unconservative_AC_000175.1_387857	0	0	0	0.18	0.36	0.69	0.000985	5.686655	up
unconservative_AC_000180.1_482694	0	0	0	0.18	0.8	0.13	0.006973	5.402332	up
bta-miR-380-3p	0	0	0	0.09	0.45	0.5	0.004013	5.398977	up
unconservative_AC_000179.1_459601	0	0.05	0	0.45	1.61	0.94	3.17E-08	5.150795	up
unconservative_AC_000182.1_502603	0	0	0.09	1.09	0.98	2.13	1.05E-09	4.995241	up
bta-miR-1298	0	0	0	0	0.27	0.5	0.03765	4.971976	up
unconservative_AC_000175.1_384914	0	0	0	0.18	0.18	0.31	0.025724	4.859309	up
bta-miR-486	0.6	0.21	0.09	0.27	32.31	1.26	4.19E-05	4.835705	up
unconservative_AC_000187.1_569828	0	0	0	0.09	0.27	0.25	0.036691	4.659082	up
unconservative_AC_000159.1_48437	0	0.11	0	1.36	0.45	0.88	3.26E-05	4.372626	up
unconservative_AC_000175.1_399428	0	0.43	0.66	2.36	5.27	7.91	4.24E-11	3.628053	up
unconservative_AC_000166.1_217448	0.06	0	0	0.36	0.45	0.13	0.034497	3.515859	up
unconservative_AC_000161.1_106583	0.72	0.53	0.75	2.91	7.41	10.04	4.45E-13	3.193355	up
unconservative_AC_000176.1_409242	0	0	0.09	0.18	0.36	0.63	0.006023	3.163992	up
unconservative_AC_000167.1_234337	0.12	0	0	0	0.45	0.82	0.043171	3.134644	up
unconservative_AC_000162.1_126365	0.06	0.21	0.09	0.82	1.52	1.13	9.86E-07	3.019437	up
bta-miR-451	4.07	2.57	1.6	1.27	75.41	2.95	0.002296	2.898241	up
unconservative_AC_000163.1_141219	0	0.11	0.05	0.09	0.27	1	0.01383	2.852016	up
bta-miR-2378	0	0.11	0.05	0.27	0.62	0.44	0.004598	2.769625	up

unconservative_AC_000173.1_350375	0.06	0.05	0.05	0.55	0.09	0.38	0.031425	2.509653	up
unconservative_AC_000184.1_528794	0.18	0	0.52	0	1.61	2.76	0.012298	2.432254	up
unconservative_AC_000163.1_144851	0.3	0.27	0.05	0.45	1.16	1.26	0.001271	2.109263	up
unconservative_AC_000176.1_413881	0.06	0.05	0.28	0.82	0.27	0.44	0.043364	1.833872	up
bta-miR-2368-3p	0.3	0.11	0.09	0.82	0.62	0.44	0.014768	1.822995	up
unconservative_AC_000158.1_2047	0.06	0.11	0.28	0.55	0.71	0.31	0.046309	1.582439	up
unconservative_AC_000173.1_359748	0.3	0.11	0.28	1.27	0.45	0.31	0.049876	1.530098	up
unconservative_AC_000161.1_105334	0.18	0.27	0.09	0.45	0.71	0.5	0.038181	1.474688	up
bta-miR-2284p	0.6	0.21	0.09	0.64	1.07	0.88	0.031231	1.428508	up
bta-miR-10a	1.98	2.03	2.49	5.64	8.84	4.2	4.51E-05	1.393471	up
unconservative_AC_000178.1_450303	0.12	0.43	0.42	0.73	0.8	1.26	0.016911	1.390449	up
bta-miR-302b	0.54	0.32	0.24	0.82	1.25	0.94	0.013871	1.351826	up
unconservative_AC_000161.1_107164	0.12	0.27	0.38	0.55	1.07	0.5	0.048941	1.261094	up
unconservative_AC_000178.1_443766	0.54	0.43	0.47	0.55	1.96	1.13	0.021118	1.175837	up
unconservative_AC_000164.1_169729	0.66	0.59	0.52	0.64	2.14	1.19	0.036115	1.008007	up
bta-miR-122	25.21	14.96	19.84	22.09	80.05	28.05	0.011493	0.920855	up
bta-miR-2461-3p	0.66	0.53	1.13	1.55	1.52	1.57	0.034909	0.891709	up
unconservative_AC_000158.1_13387	3.23	3.63	3.71	5.64	7.85	5.21	0.017488	0.704887	up
bta-miR-145	17.25	20.36	18.75	10.46	14.37	15.37	0.026931	-0.59608	down
bta-miR-133a	2.99	2.03	2.54	0.91	2.05	1.88	0.035594	-0.74901	down
bta-miR-2339	2.57	2.19	3.48	1.27	2.05	1.82	0.024032	-0.7879	down
unconservative_AC_000178.1_442694	1.86	1.23	1.83	0.73	0.98	1.07	0.029386	-0.90796	down
bta-miR-2403	2.28	3.1	3.67	1	2.14	1.88	0.005728	-0.97198	down
unconservative_AC_000172.1_343848	1.5	1.07	1.46	0.55	0.45	1.07	0.031352	-0.99572	down
unconservative_AC_000171.1_312038	23.29	15.39	20.92	9.18	11.51	8.85	8.78E-05	-1.10004	down

unconservative_AC_000176.1_403475	0.9	0.8	0.8	0.27	0.45	0.44	0.030047	-1.18799	down
unconservative_AC_000173.1_357367	1.92	0.91	1.74	0.45	0.71	0.88	0.007399	-1.21819	down
unconservative_AC_000163.1_149041	0.54	0.48	0.71	0	0.36	0.38	0.042663	-1.28902	down
unconservative_AC_000168.1_263586	0.9	0.69	0.99	0.64	0.18	0.25	0.048858	-1.3487	down
unconservative_AC_000170.1_301545	1.62	1.07	0.52	0.45	0.36	0.44	0.019844	-1.41409	down
unconservative_AC_000171.1_311957	0.96	0.64	0.89	0.36	0.45	0.19	0.012626	-1.45983	down
bta-miR-2338	0.9	0.53	0.56	0	0.54	0.25	0.029852	-1.46608	down
unconservative_AC_000159.1_32276	125.93	89.89	126.96	44.64	49.44	33.01	3.88E-07	-1.49776	down
unconservative_AC_000173.1_360722	1.98	1.28	1.13	0.55	0.54	0.38	0.00082	-1.69136	down
unconservative_AC_000162.1_112908	1.92	1.18	1.27	0.55	0.45	0.38	0.000583	-1.76325	down
unconservative_AC_000177.1_440668	1.44	0.53	0.94	0.18	0.45	0.25	0.002478	-1.82081	down
unconservative_AC_000182.1_510741	1.92	1.92	1.69	0.45	0.54	0.56	1.58E-05	-1.91461	down
unconservative_AC_000182.1_503109	0.36	0.21	0.47	0.09	0	0.13	0.045084	-2.14484	down
unconservative_AC_000182.1_503110	0.36	0.21	0.47	0.09	0	0.13	0.045081	-2.14484	down
unconservative_AC_000159.1_44566	0.24	0.21	0.38	0	0.09	0.06	0.038654	-2.37351	down
unconservative_AC_000169.1_283348	0.06	0.27	0.47	0	0.09	0	0.034728	-3.12759	down
unconservative_AC_000165.1_188071	0.36	0.21	0.24	0	0	0.06	0.018508	-3.14083	down
unconservative_AC_000159.1_36513	0.66	0.11	0.33	0.09	0	0	0.022762	-3.4434	down
unconservative_AC_000183.1_527038	0.36	0.48	0.24	0	0.09	0	0.003763	-3.53423	down
unconservative_AC_000161.1_89964	0.6	0.32	0.42	0	0	0.06	0.000546	-3.84352	down
unconservative_AC_000175.1_395122	0.24	0.05	0.33	0	0	0	0.030768	-4.79325	down
unconservative_AC_000169.1_281373	0.3	0.16	0.33	0	0	0	0.005205	-5.13637	down
unconservative_AC_000168.1_251602	0.18	0.43	0.19	0	0	0	0.007187	-5.18172	down
unconservative_AC_000185.1_544675	0.66	0	0.28	0	0	0	0.01247	-5.34524	down
