# Supplementary material for: DNA Double-Strand Break-Related Competitive Endogenous RNA Network of Noncoding RNA in Bovine Cumulus Cells
Source: Genes (Basel). 2023 Jan 22;14(2):290. doi: 10.3390/genes14020290 (PMC9956238; doi:10.3390/genes14020290)
Supplement: Supplementary file 1 [file genes-14-00290-s001.zip › Table S7.pdf]

Supplementary Table S7. The relationship between differentially expressed RNAs and DSB/DDR

Differentially expressed RNAs			Reported
RNAs	Up/down	KEGG	DSB/DDR function
UHRF1	down	-	depletion causes a G2/M arrest, activation of DNA damage response
REV3L	up	Fanconi anemia pathway (ko03460)	Knockdown caused DDR disorder
PIF1	down	-	Pif1 is recruited to DNA repair foci after induction of DSBs, as a key player in genome maintenance
FOXO1	down	FoxO signaling pathway (ko04068); AMPK signaling pathway (ko04152); Insulin signaling pathway (ko04910); Thyroid hormone signaling pathway (ko04919); Pathways in cancer (ko05200); Transcriptional misregulation in cancer (ko05202); Prostate cancer (ko05215)	DNA damage can induce FOXO1 expression and promotes the transcriptional activity, target p27Kip1, GADD45, Bim and induced cell cycle arrest responds to DNA damage
MASTL	down	-	MASTL controls the timing of mitotic entry after DNA damage
E2F1	down	Cell cycle (ko04110); Pathways in cancer (ko05200); MicroRNAs in cancer (ko05206); Pancreatic cancer (ko05212); lik Glioma (ko05214); Prostate cancer (ko05215); Melanoma (ko05218); Bladder cancer (ko05219); Chronic myeloid leukemia (ko05220); Small cell lung cancer (ko05222); Non-small cell lung cancer (ko05223)	Ectopic expression of these E2Fs in cells reduces the level of DNA damage following genotoxic treatment, while ablation of E2F1 and E2F2 leads to the accumulation of DNA lesions and increased apoptotic response.
CRY2	up	Circadian rhythm (ko04710)	recognition and repair of DNA damage

FBXW7	up	Ubiquitin mediated proteolysis (ko04120)	FBXW7 is recruited to DNA damage sites to facilitate non-homologous end joining
POLE	down	Purine metabolism (ko00230);<br>Pyrimidine metabolism (ko00240);<br>DNA replication (ko03030);<br>Base excision repair (ko03410);<br>Nucleotide excision repair (ko03420);<br>HTLV-I infection (ko05166)	POLE mutation affects DSB and DDR
RFWD3	down	-	RFWD3 is an E3 ligase that affects DDR via ubiquitination RPA and RAD51
XPC	up	Nucleotide excision repair (ko03420)	USP11 positively regulates NER by deubiquitinating XPC and promotes its retention at the site of DNA damage
CDKN1A	up	ErbB signaling pathway (ko04012);<br>HIF-1 signaling pathway (ko04066);<br>FOXO signaling pathway (ko04068);<br>Cell cycle (ko04110);<br>p53 signaling pathway (ko04115);<br>PI3K-Akt signaling pathway (ko04151);<br>Oxytocin signaling pathway (ko04921);;<br>Hepatitis C (ko05160);Hepatitis B (ko05161);<br>HTLV-I infection (ko05166);<br>Epstein-Barr virus infection (ko05169);<br>Pathways in cancer (ko05200);<br>Transcriptional misregulation in cancer (ko05202);<br>Viral carcinogenesis (ko05203);<br>Proteoglycans in cancer (ko05205);<br>MicroRNAs in cancer (ko05206);	P21 participates in the DSB repair pathway and promotes HR by inhibiting CDK

		Glioma (ko05214); Prostate cancer (ko05215);<br>Melanoma (ko05218);<br>Bladder cancer (ko05219);<br>Chronic myeloid leukemia (ko05220)	
DNA2	down	DNA replication (ko03030)	DNA2 is recruited to the DSB site and its excision to participate in DSB repair,
PIDD1	up	NF-kappa B signaling pathway (ko04064);<br>p53 signaling pathway (ko04115)	PIDD activates NF-κB to participate in DDR
CASP2	down	-	DNA damage depends on PIDD activation of CASP2
PAXIP1	down	-	PAXIP1 plays a role in DSB and promotes non-homologous end joining (NHEJ) repair.
RAD51AP1	down	-	RAD51AP1 mediates DDR
CHAF1A	down	-	CHAF1A suppresses DDR
miR-486	up	metabolism (ko00052);<br>Starch and sucrose metabolism (ko00500); Amino sugar and nucleotide sugar metabolism (ko00520);<br>Butirosin and neomycin biosynthesis (ko00524);<br>Carbon metabolism (ko01200);<br>HIF-1 signaling pathway (ko04066);<br>Insulin signaling pathway (ko04910);<br>Type II diabetes mellitus (ko04930);<br>Carbohydrate digestion and absorption (ko04973);	Overexpression of miR-486 inhibits JARID1B protein accumulation and up-regulates BRCA1 expression affects DSB

		Central carbon metabolism in cancer (ko05230)<br>NF-kappa B signaling pathway (ko04064);<br>p53 signaling pathway (ko04115);<br>Base excision repair (ko03410)	
miR-451	up	-	AntogomiR-451 attenuates DNA damage by activating AMPK signaling
miR-145	down	Hippo signaling pathway (ko04390)<br>NF-kappa B signaling pathway (ko04064);<br>Cell adhesion molecules (CAMs) (ko04514);<br>Natural killer cell mediated cytotoxicity (ko04650);<br>TNF signaling pathway (ko04668);<br>Leukocyte transendothelial migration (ko04670);<br>African trypanosomiasis (ko05143);<br>Staphylococcus aureus infection (ko05150);<br>Influenza A (ko05164); Malaria (ko05144);<br>HTLV-I infection (ko05166);<br>Epstein-Barr virus infection (ko05169);<br>Rheumatoid arthritis (ko05323);<br>Viral myocarditis (ko05416)	Arsenite promotes DNA damage by targeting ERCC2 to induce overexpression of miR-145 and inhibit DNA repair
