# Supplementary figures and images for: DNA Double-Strand Break-Related Competitive Endogenous RNA Network of Noncoding RNA in Bovine Cumulus Cells
Source: Genes (Basel). 2023 Jan 22;14(2):290. doi: 10.3390/genes14020290 (PMC9956238; doi:10.3390/genes14020290)

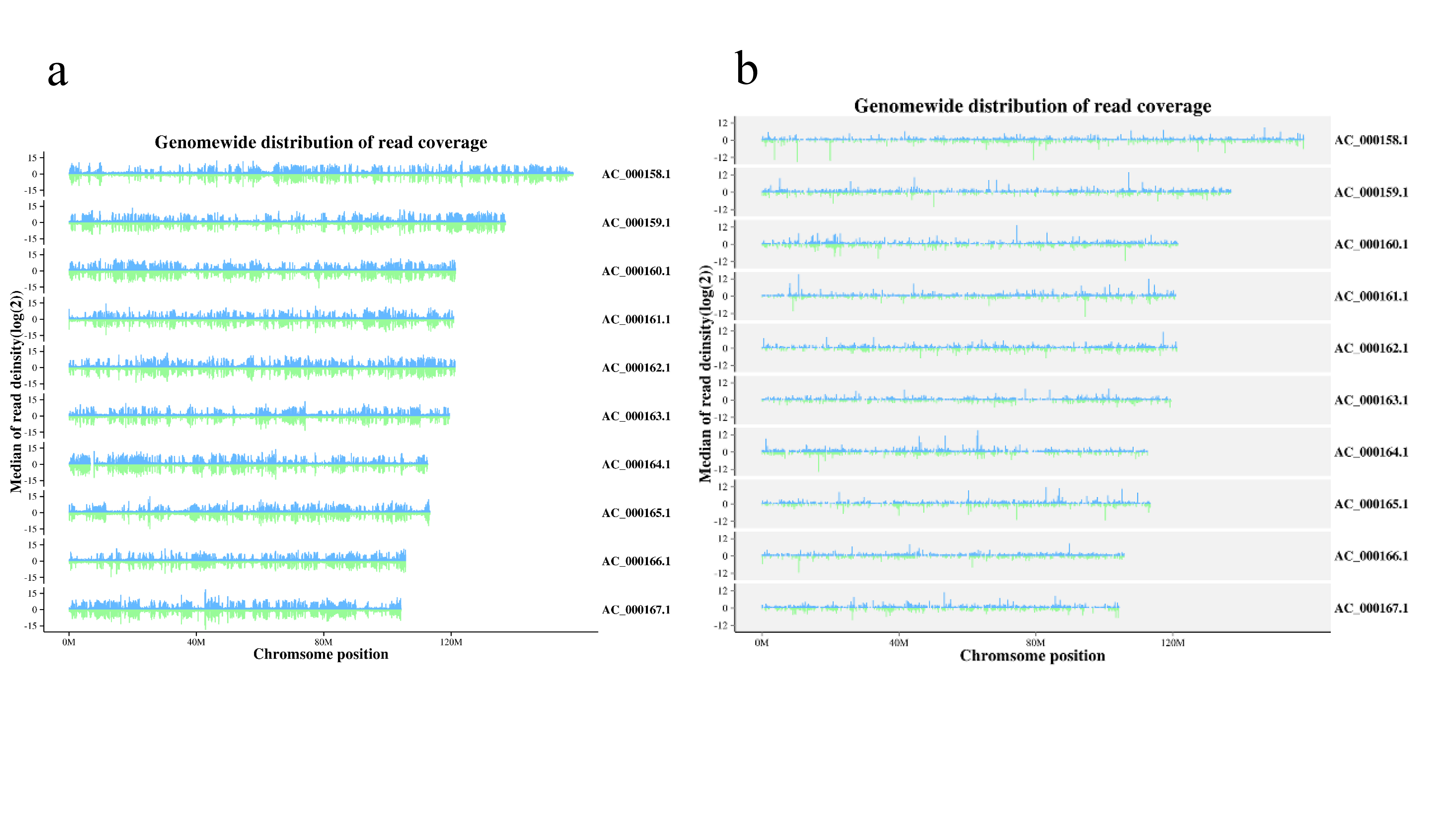

Supplement: Supplementary file 1 [file genes-14-00290-s001.zip › Figure S1.tif]

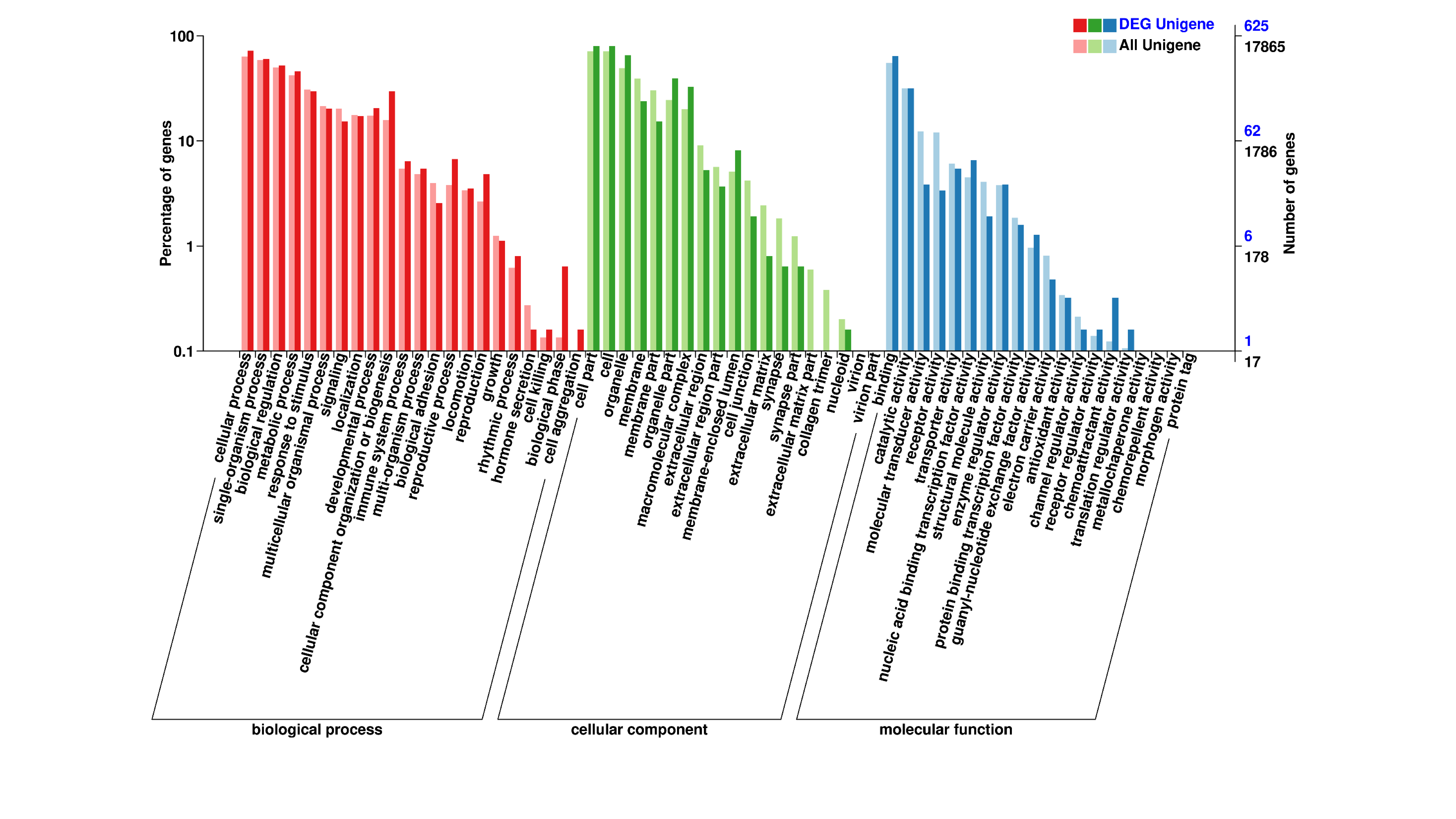

Supplement: Supplementary file 1 [file genes-14-00290-s001.zip › Figure S2.tif]

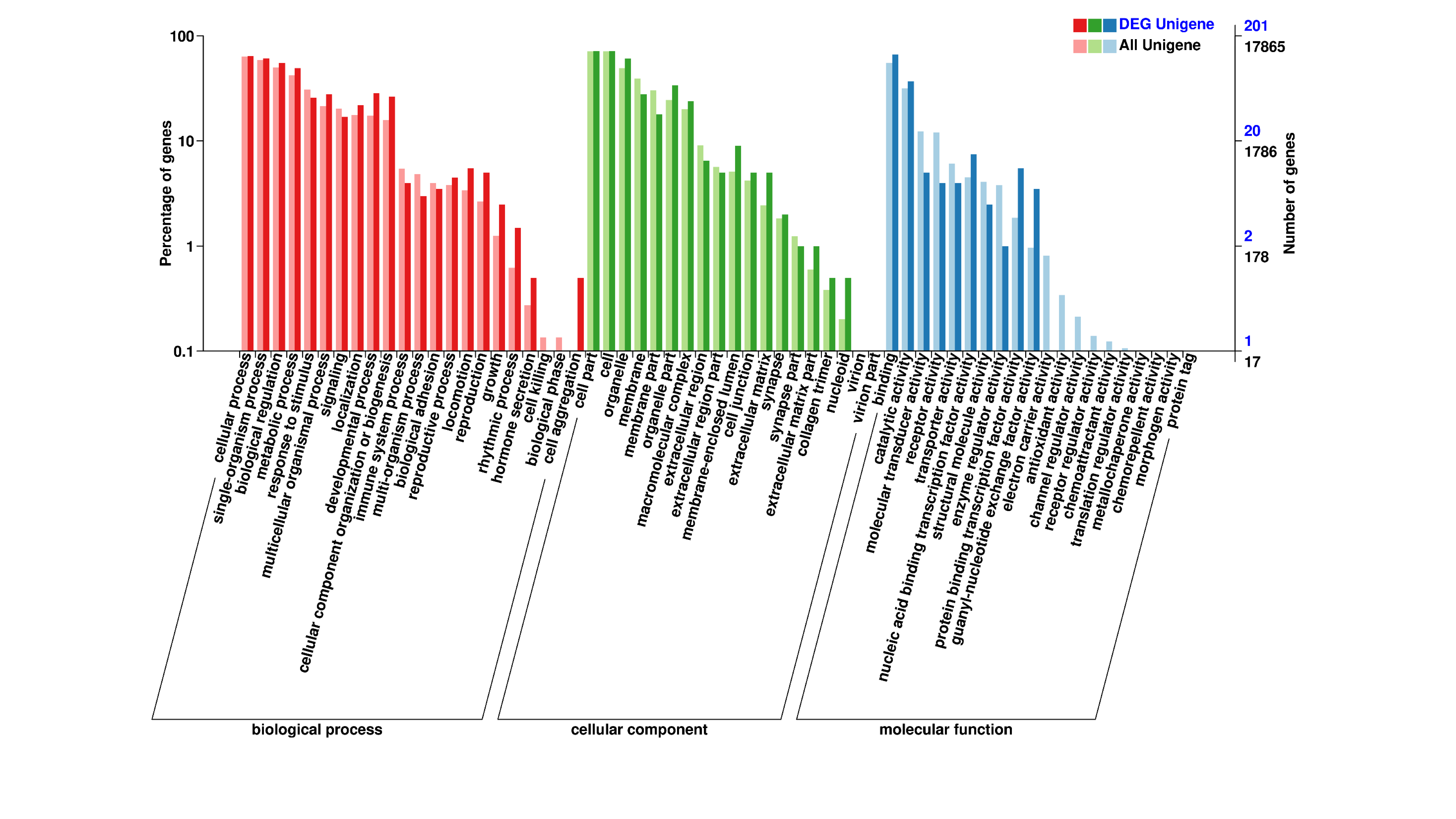

Supplement: Supplementary file 1 [file genes-14-00290-s001.zip › Figure S3.tif]

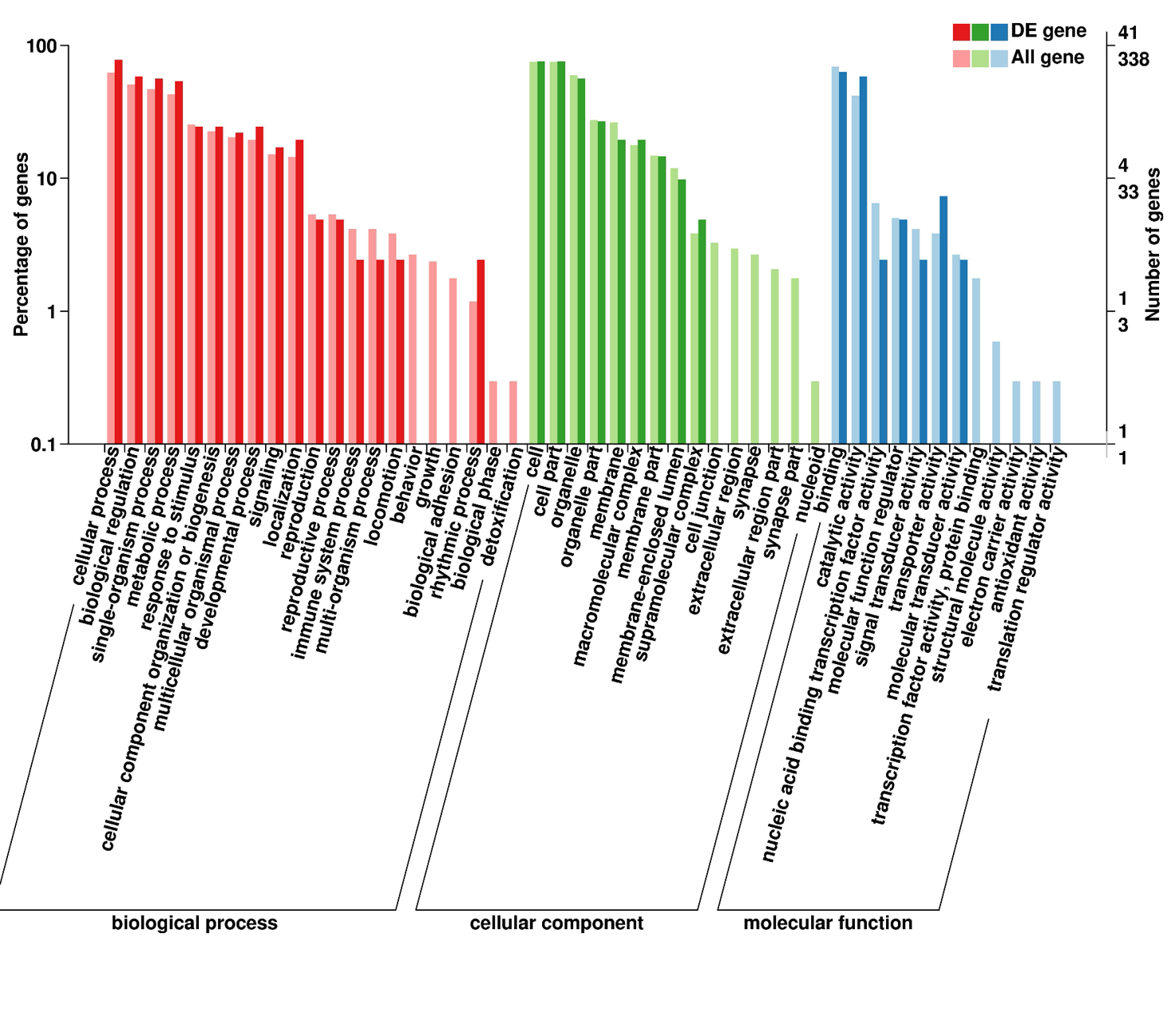

Supplement: Supplementary file 1 [file genes-14-00290-s001.zip › Figure S4.tif]

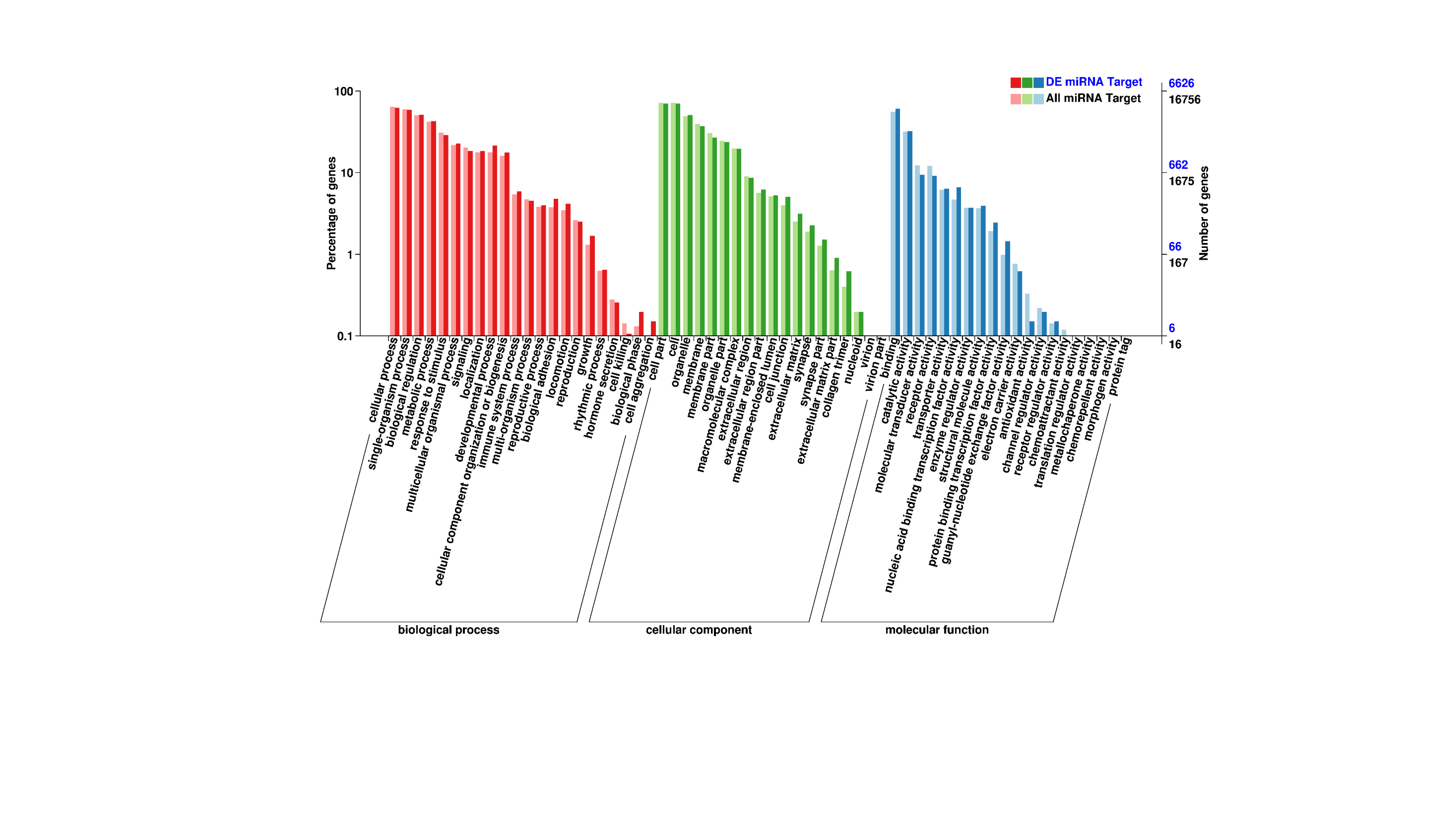

Supplement: Supplementary file 1 [file genes-14-00290-s001.zip › Figure S5.tif]

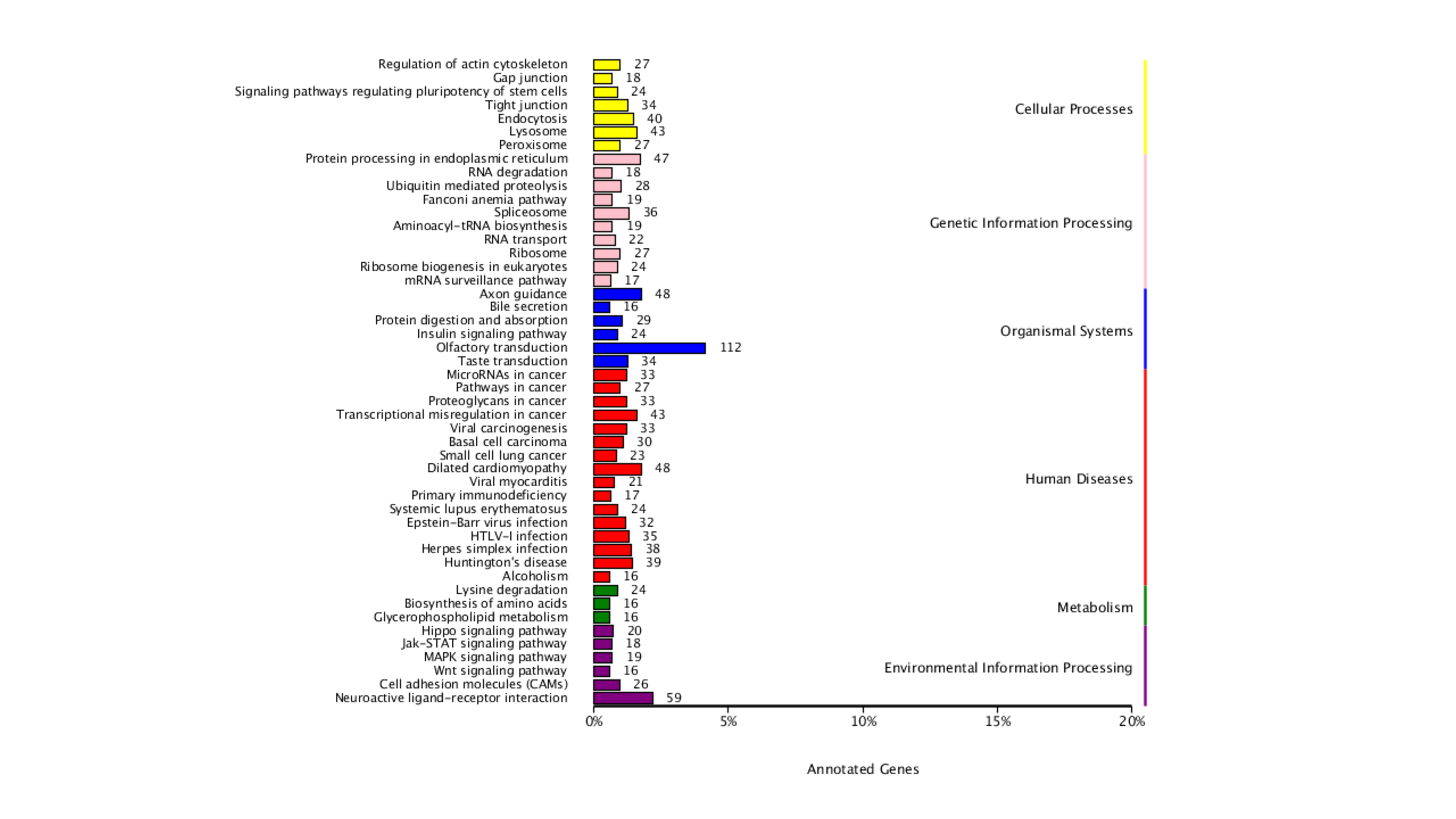

Supplement: Supplementary file 1 [file genes-14-00290-s001.zip › Figure S6.tif]

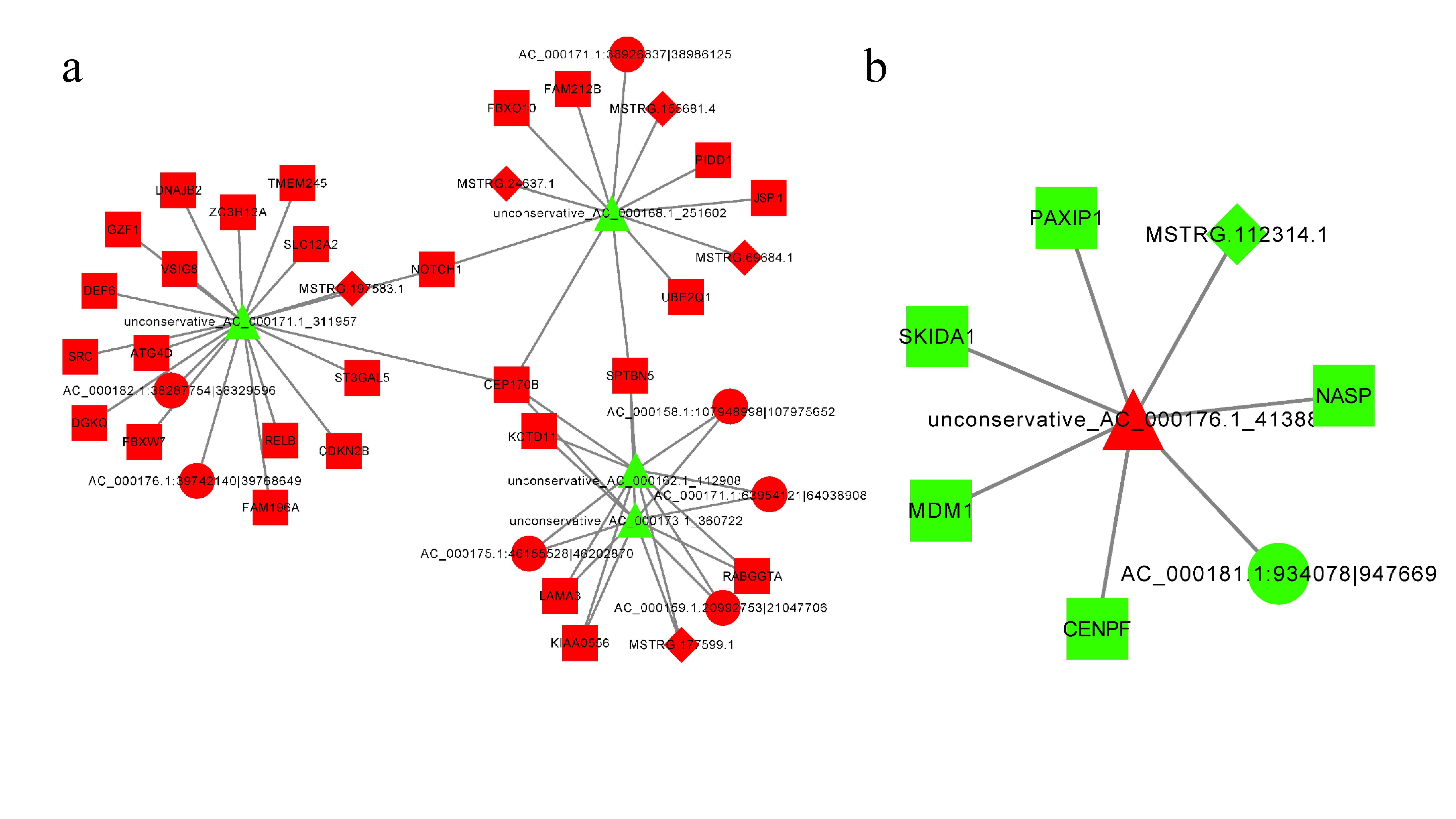

Supplement: Supplementary file 1 [file genes-14-00290-s001.zip › Figure S7.tif]
